# Comparison of Mg–Yb and Mg–Ca bonding: structures, electronic analyses and reactivities

**DOI:** 10.1039/d6sc05592c

**Published:** 2026-09-21

**Authors:** Sandeep Kumar Thakur, Tanuja Tewari, Nil Roig, Jens Langer, Mercedes Alonso, Sjoerd Harder

**Affiliations:** a Inorganic and Organometallic Chemistry, Universität Erlangen-Nürnberg Egerlandstrasse 1 Erlangen 91058 Germany sjoerd.harder@fau.de; b Institut de Química Computacional i Catàlisi (IQCC) and Departament de Química, Facultat de Ciències, Universitat de Girona (UdG) C/Maria Aurèlia Capmany 69 Girona 17003 Catalonia Spain; c Eenheid Algemene Chemie (ALGC), Vrije Universiteit Brussel (VUB) Brussels 1050 Belgium

## Abstract

Reaction of [(BDI*)MgNa]_2_ with (Am*YbI)_2_ in methylcyclohexane led to the precipitation of NaI and quantitative formation of (BDI*)Mg–Yb(Am*) (1-Yb); BDI* = HC[C(*t*Bu)N(DIPeP)]_2_, DIPeP = 2,6-Et_2_CH-phenyl; Am* = *t*BuC(N-DIPP)_2_, DIPP = 2,6-*i*Pr-phenyl. Complex 1-Yb features a long unsupported 2c–2e Mg–Yb bond of 3.302 Å and crystallizes isomorphous to (BDI*)Mg–Ca(Am*) (1-Ca). The formal Mg^0^–Yb^II^ oxidation states in 1-Yb are confirmed by effective-oxidation-state analysis but the low reliability index of 68.0% indicates that this description is not fully representative of the electron distribution. Analysis of the electron density shows significant Mg^0^ → Yb^II^ electron transfer and therefore the complex has partial Mg^I^–Yb^I^ character. EDA-NOCV analysis suggests that bonding in 1-Yb is best pictured as a combination of neutral open-shell radicals (BDI*)Mg^˙^/(Am*)Yb^˙^. Accordingly, (BDI*)Mg acts as an X-type ligand for Yb(Am*). The long, polarized Mg^*δ*−^–Yb^*δ*+^ bond in 1-Yb is highly reactive. The thermal decomposition and reactivity of 1-Yb with N_2_O, NH_3_, 1,1-diphenylethylene, *trans*-stilbene and anthracene have been studied. Comparison with the reactivity of 1-Ca shows strong similarities, but also important differences. Most reactants oxidize the Mg^0^ centre in 1-Yb and 1-Ca and produce similar complexes that generally crystallize isomorphous. However, N_2_O can also oxidize the Yb^II^ centre, resulting in paramagnetic Yb^III^ products. In all cases, the Mg–Yb and Mg–Ca bonds in 1-Yb and 1-Ca are considerably more reactive than the unpolarized Mg–Mg bond in complexes of type (BDI)Mg–Mg(BDI).

## Abstract 2

A complex featuring an unsupported 2c–2e Mg–Yb bond and formal oxidation states Mg^0^–Yb^II^ was isolated. The properties and very high reactivity of the polarized Mg^*δ*−^–Yb^*δ*+^ bond are compared with those of a related Mg–Ca complex.

## Graphical abstract

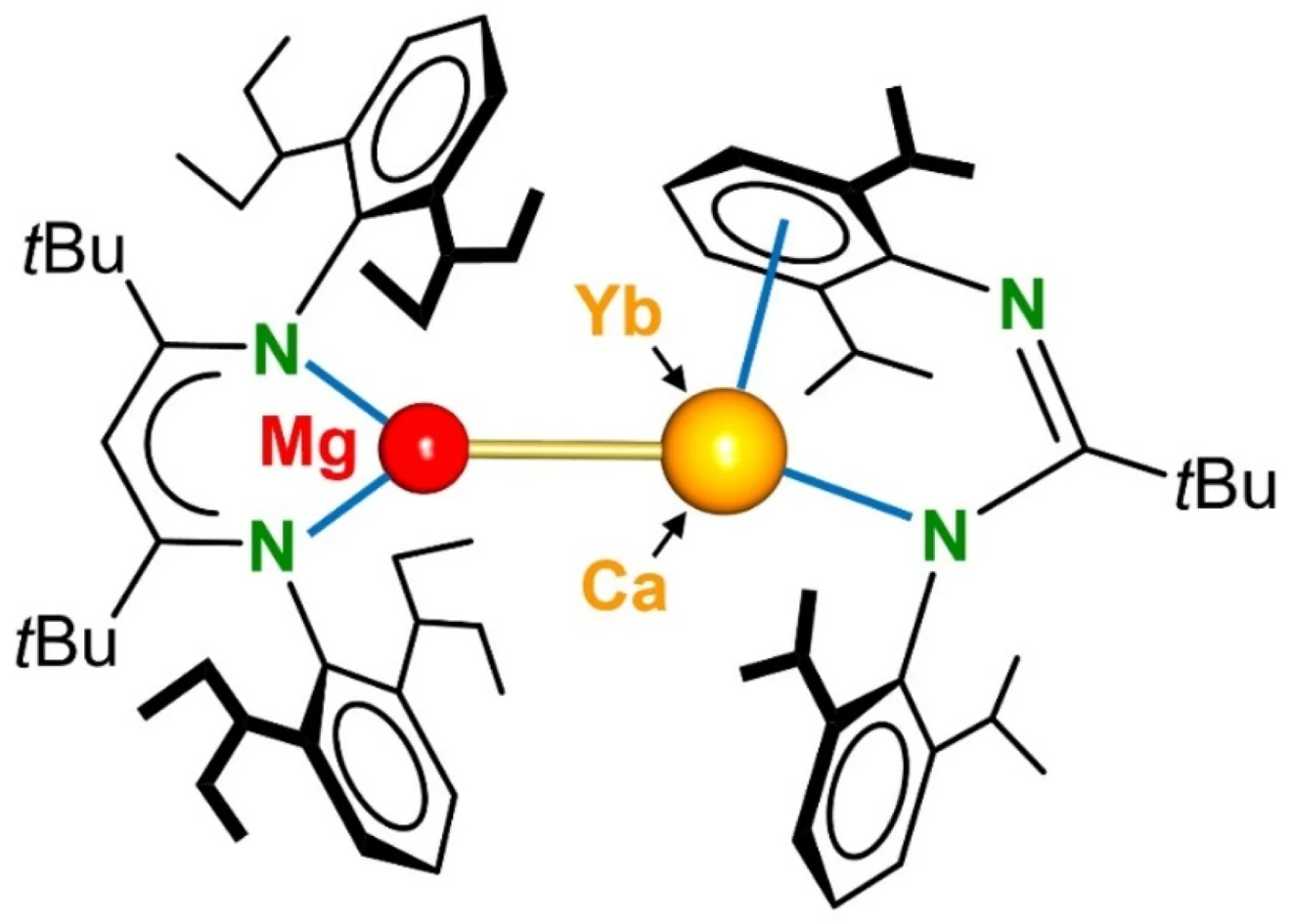

## Introduction

Cotton's first examples of metal–metal bonding are now more than half a century old.^1^ What began as investigations of homometallic multiple bonding between transition metals has developed into a large research field covering homo- and heterometallic bonding in all varieties throughout the periodic table.^2^ The vast majority of these bonds involve p- and d-block metals whereas f- and especially s-block metal–metal bonding remain clearly underrepresented. This is undoubtedly due to their highly electropositive character, which results in facile loss of the electrons required for metal–metal bonding. There are rare examples of unusual, supported metal–metal interactions between f-block elements.^3–6^ Stable complexes with bonding between group 1 metals are unknown although long, weak M^+^⋯M^−^ or M^−^⋯M^−^ metal–metal interactions in alkalides have been discussed.^7,8^ The only homometallic metal–metal bonds established in the s-block involve the most electronegative metals, Be and Mg. Whereas stable (BDI)Mg–Mg(BDI), (Gua)Mg–Mg(Gua) or CpBe–BeCp complexes have been reported (BDI = β-diketiminate ligand, Gua = guanidinate ligand, and Cp = cyclopentadienyl ligand),^9,10^ attempts to isolate similar complexes for the larger, more electropositive alkaline-earth (Ae) metals Ca and Sr resulted in electron loss and reduction of either the solvent or N_2_ used as a protecting gas.^11–13^

Aiming to further develop the rapidly growing field of low-oxidation-state group 2 metal chemistry,^14–17^ we recently isolated a Mg^0^/Na^I^ complex: [(BDI*)Mg^−^Na^+^]_2_ (I, Scheme 1, BDI* = HC[C(*t*Bu)N(DIPeP)]_2_, DIPeP = 2,6-Et_2_CH-phenyl).^18^ This unusual magnesyl sodium dimer, for which a second example has been recently reported,^19^ displays unique Mg–Na bonding that is best described as an electrostatic interaction between a magnesyl anion, (BDI*)Mg^−^, and a Na^+^ cation. However, charge distribution calculations show slight electron transfer from the electron rich (BDI*)Mg^−^ anion to Na^+^, indicating that this is a simplified bonding model.

**Scheme 1:**
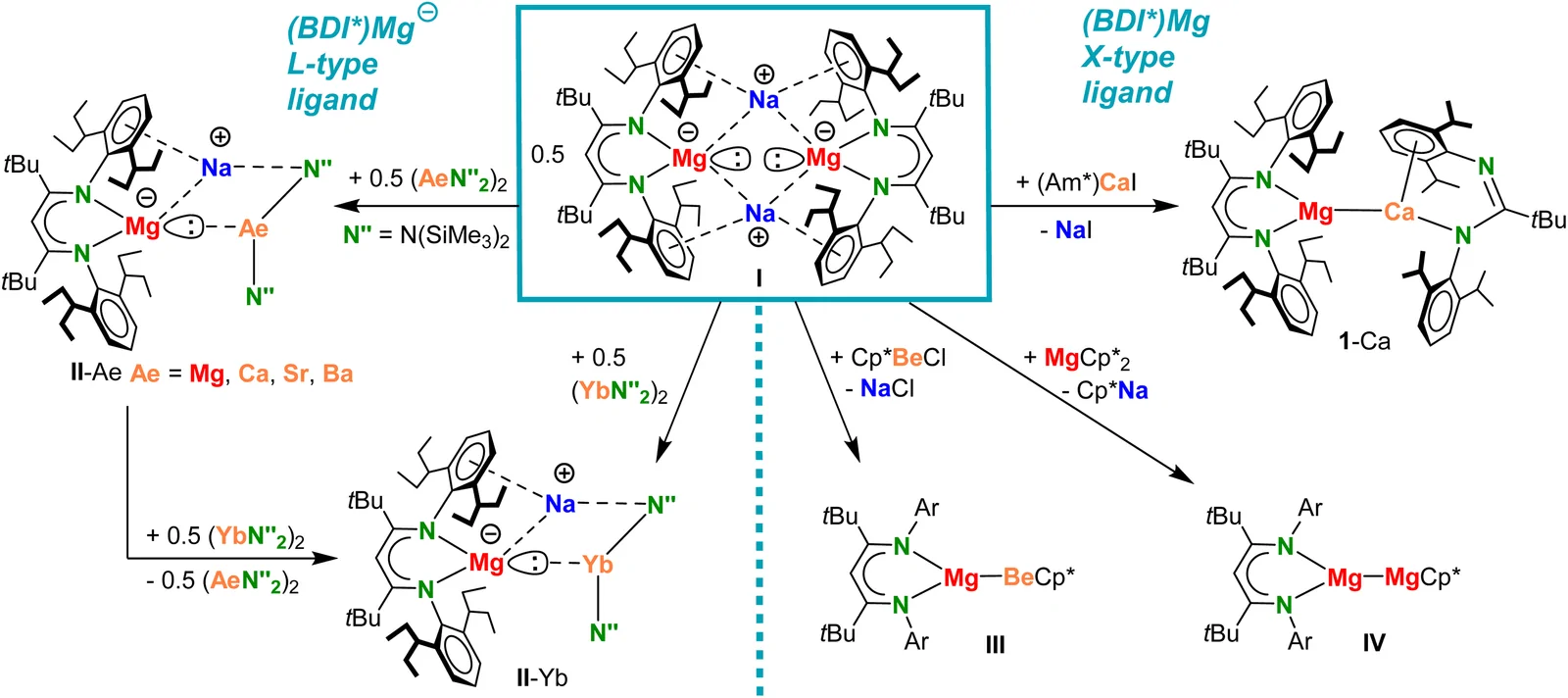
The magnesyl sodium complex [(BDI*)Mg^−^Na^+^]_2_ (I) as a key precursor to create new Mg–metal bonds either by ligand–metal exchange (left) or salt-metathesis (right) (Ar = 2,6-Et_2_CH-phenyl).

Mixing dimeric I with (AeN″_2_)_2_ (N″ = N(SiMe_3_)_2_) gave access to a range of mixed aggregates with unique Mg–Ae bonds (II-Ae, Ae = Mg, Ca, Sr, Ba).^20^ These aggregates could be regarded as (BDI*)Mg–AeN″ complexes stabilized by aggregation with NaN″. Also, these complexes feature slight electron transfer from the electron rich (BDI*)Mg^−^ anion to Ae^2+^ and Na^+^. Charge distributions show that (BDI*)Mg^−^ → Ae^2+^ electron transfer increases with increasing Lewis acidity of the Ae^2+^ cation (Ba^2+^ < Sr^2+^ < Ca^2+^ < Mg^2+^). The large, electropositive Ae^2+^ cations form very long and rather labile Mg–Ae bonds, whereas the small, more electronegative Mg^2+^ cation forms the strongest bond. The Mg–Mg bond in II-Mg is so strong that NaN″ elimination to give (BDI*)Mg–MgN″ is facile. However, in the heavier complexes of type II-Ae (Ae = Ca, Sr, Ba) the Mg–Ae bond is weak. Therefore, they are in dynamic equilibrium with (BDI*)Mg^−^Na^+^ and AeN″_2_. This allows facile exchange of Ae for another metal M by simple addition of MN″_2_.

Based on the close similarity between Ca^II^ and Yb^II^ complexes,^21^ we could also isolate II-Yb which shows the same structure and properties as II-Ca.^22^ The Mg–Ca and Mg–Yb bonds in II-Ca and II-Yb are supported by interaction with a Na–N″ moiety. According to Green's MLX covalent-bond-classification,^23,24^ the (BDI*)Mg^−^ anion in II-Ca or II-Yb could be considered as a L-type ligand, *i.e.* an anionic 2e^−^ Lewis base coordinating to AeN″_2_ or YbN″_2_ in a similar manner to neutral Cp*Ga or THF coordinating to AeCp_2_*.^25–27^

In a different approach, the magnesyl sodium complex I has been used as a reactant in salt-metathesis reactions (Scheme 1). Thus, we have been able to prepare complexes in which (BDI*)Mg is an X-type ligand for BeCp* (III),^28^ MgCp* (IV)^29^ or Ca(Am*) (1-Ca);^30^ Am* = *t*BuC(N-DIPP)_2_ and DIPP = 2,6-*i*Pr-phenyl. These complexes feature unsupported Mg–Ae bonds in which both metals contribute one electron each. Based on the decreasing electronegativities descending group 2 (Be: 1.56, Mg: 1.31, and Ca: 1.00),^31^ the formal oxidation state assignments in these types of complexes are Mg^II^/Be^0^ (III), Mg^I^/Mg^I^ (IV) and Mg^0^/Ca^II^ (1-Ca). Although formal oxidation states are not equivalent to metal charges, the calculated NPA charges (III: Mg +1.21/Be +0.62; 1-Ca: Mg +0.66/Ca +1.28) rationalize the observed switch in metal–metal bond polarization from Mg^*δ*+^–Be^*δ*−^ to Mg^*δ*−^–Ca^*δ*+^. Indeed, the Mg centre in (BDI*)Mg-Ca(Am*) (1-Ca) was shown to react as a nucleophile, whereas Ca functions as an electrophile.^30^ Despite these roles and formal oxidation states Mg^0^/Ca^II^, there is considerable transfer of electron density from Mg^0^ to Ca^II^. This is evident from the NPA charge of +1.28 on Ca which is significantly lower than typical values calculated for Ca^II^ (*ca.* +1.75). It is also considerably lower than the +1.52 charge on Ca in II-Ca in which (BDI*)Mg^−^ is an L-type ligand. In fact, the +1.28 charge on Ca in 1-Ca is the lowest calculated charge for any reported Ca complex. Hence, this complex could be considered to have partial Mg^I^/Ca^I^ character.

As there are striking similarities, but also important differences in Ca^+II^ and Yb^+II^ chemistry,^21^ we extended this chemistry through the isolation and full characterization of (BDI*)Mg–Yb(Am*) (1-Yb). This complex represents the first lanthanide complex with an unsupported s-block metal–Yb bond. In addition, we expect an unusually low charge on Yb and a significant contribution of Mg^I^/Yb^I^ character.

## Results and discussion

### Synthesis

The synthesis of (BDI*)Mg–Yb(Am*) (1-Yb) was achieved following a similar protocol to that reported for (BDI*)Mg–Ca(Am*) (1-Ca, Scheme 2). Salt-metathesis between Am*K and YbI_2_ gave the precursor (Am*YbI)_2_ (2-Yb) in the form of an orange powder in 70% yield. It crystallized from pentane solution as an ether-free dimer (Fig. S57). ^1^H NMR analysis (Fig. S1) revealed two different DIPP-substituents indicating that the *N-*aryl-chelation of the Am* ligand is maintained in solution.

**Scheme 2:**
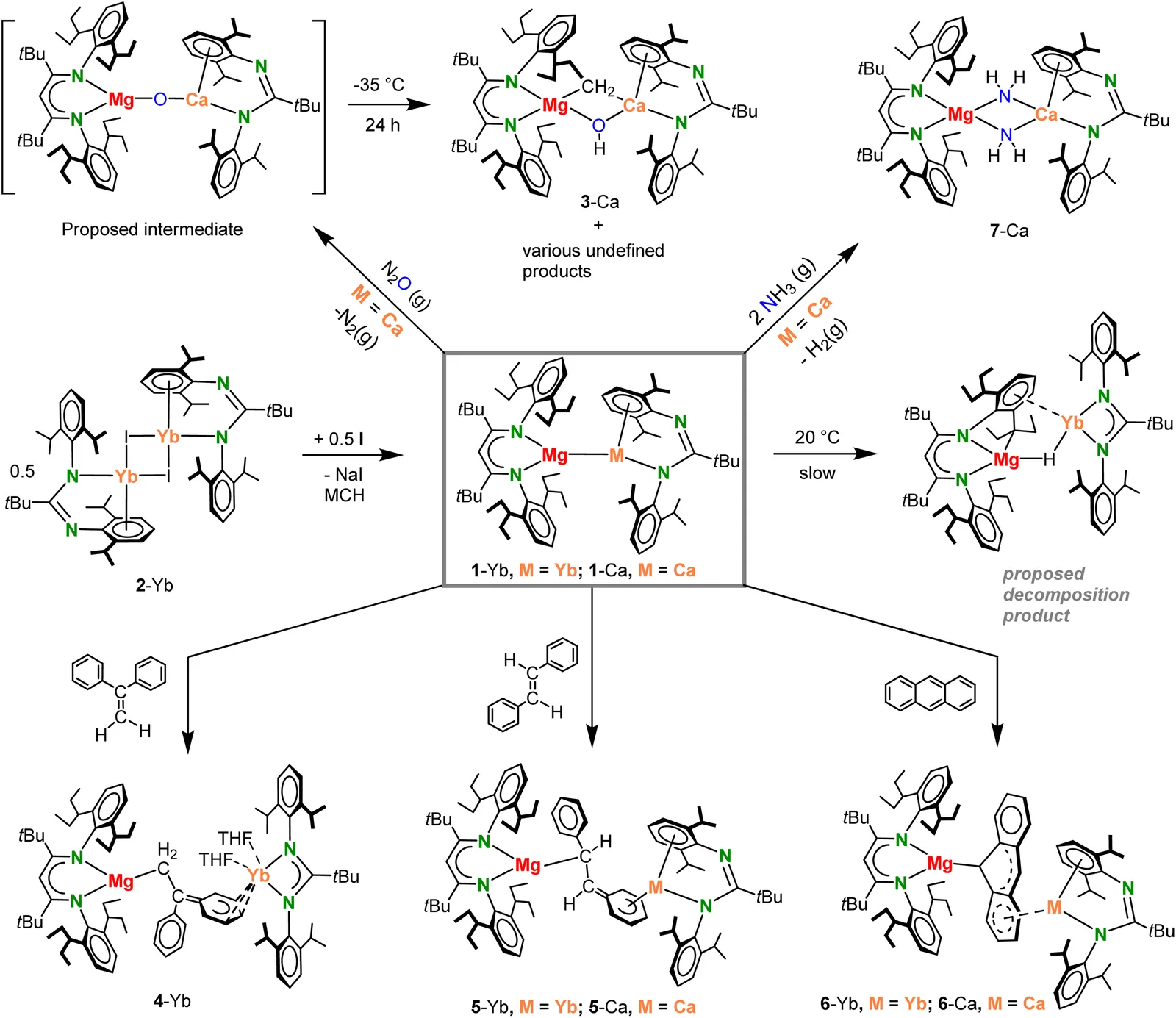
Synthesis of (BDI*)Mg–Yb(Am*) (1-Yb), and reactivity of 1-Yb and the previously reported 1-Ca complex.

Reaction of (Am*YbI)_2_ (2-Yb) with the magnesyl sodium dimer (I) in methylcyclohexane led to immediate precipitation of NaI and quantitative formation of the expected (BDI*)Mg–Yb(Am*) complex (1-Yb). The crude product obtained after solvent removal is essentially pure, but can also be crystalized overnight from a concentrated pentane solution at −35 °C in 94% yield. Like 1-Ca, it crystallized in the triclinic space group *P*1̄ with very similar cell parameters (Fig. 1a).^30^ There are two independent molecules in the asymmetric unit with nearly equal Mg–Yb bond lengths (3.299(2) Å and 3.305(2) Å). The average Mg–Yb distance of 3.302 Å is close to the sum of the covalent radii of 3.28 Å (Mg 1.41 and Yb 1.87)^32^ and very similar to the Mg–Ca bond length in 1-Ca (3.298 Å). However, it is considerably shorter than the Mg–Yb bond in II-Yb (3.466(1) Å).^22^ Fitting the structure of 1-Yb on that of 1-Ca shows their close similarity (Fig. 1b).

**Fig. 1:**
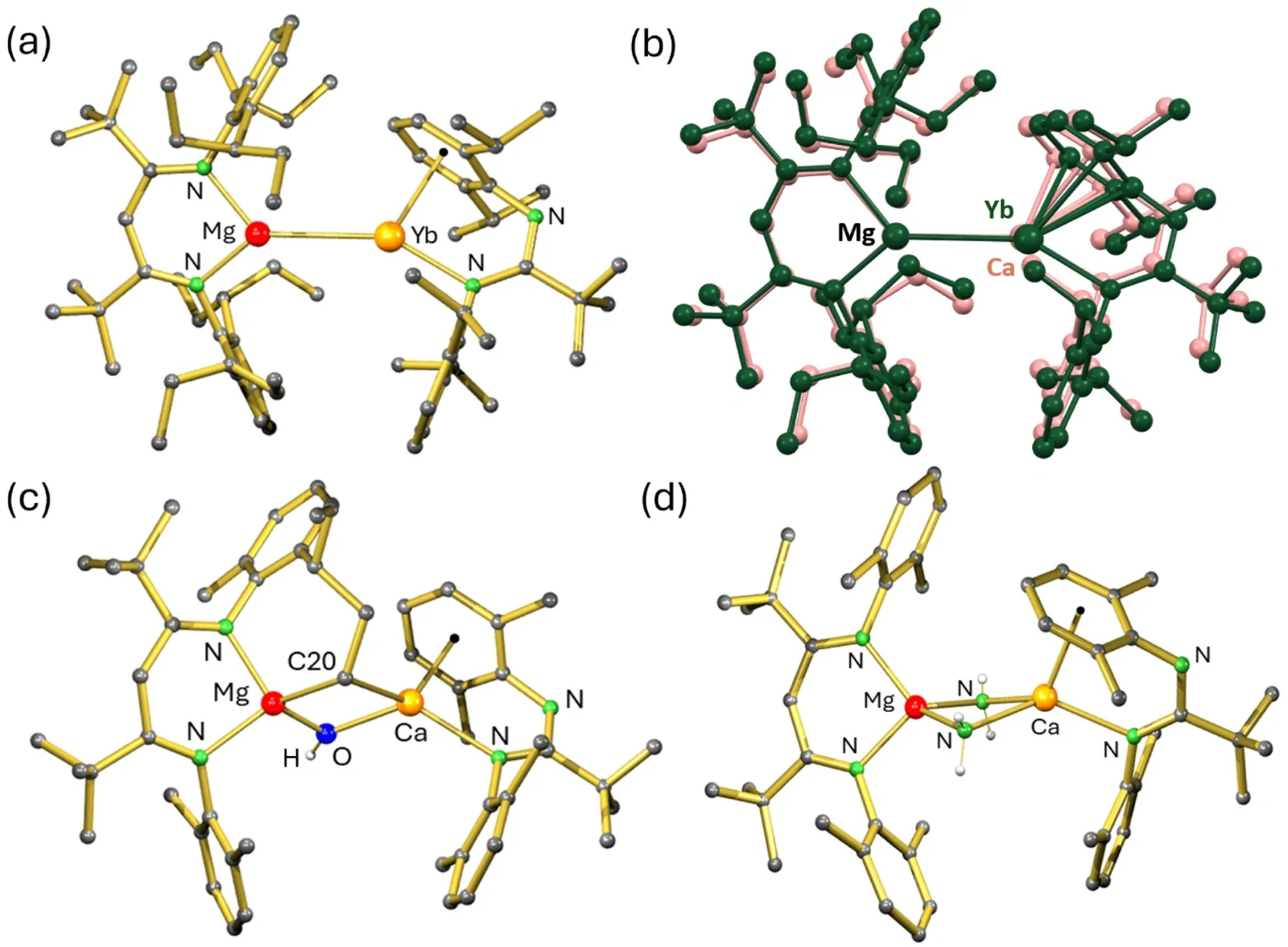
(a) Crystal structure of 1-Yb. (b) Superimposed structures of 1-Yb (dark-green) and 1-Ca (pink). (c) Crystal structure of 3-Ca. (d) Crystal structure of 7-Ca. All H atoms (except OH and NH hydrogen) are omitted for clarity. In 3-Ca and 7-Ca, the Et and Me groups from BDI* and Am*, respectively, have also been omitted for clarity.

The Mg–N bond lengths in 1-Yb range from 2.086(4) to 2.107(5) Å. The average Mg–N bond (2.094 Å) is longer as expected for BDI*–Mg^II^ coordination. For example, the average Mg–N bond distance in (BDI*)MgI, which features a similar tricoordinate Mg centre, is only 1.998 Å.^33^ As we noticed earlier, the Mg–N bond distances are very sensitive to the oxidation state of the Mg centre. Complex 1-Yb is formally a Mg^0^/Yb^II^ complex and the electron rich Mg^0^ centre results in Mg–N bond elongation. However, the average Mg–N bond distance in 1-Yb (2.094 Å) is smaller than the Mg^0^–N bond in I (2.117 Å)^18^ but larger than the Mg^I^–N bond in (BDI*)Mg–Mg(BDI*)^33^ (2.064 Å). This is a strong indication for partial transfer of electron density from the electron rich Mg^0^ centre to Yb^II^. Such transfer of electron density to Yb is also in agreement with the rather long Yb–N bonds in 1-Yb (2.392(4)–2.421(5) Å, average 2.407 Å) when compared to that in precursor 2-Yb (2.369 Å). Also, the Yb⋯C(Ar) contacts in 1-Yb (average: 2.485 Å) are longer than in 2-Yb (average: 2.429 Å). Charge calculations confirm the electron-rich nature of both, Mg and Yb (*vide infra*).

Complexes 1-Ca and 1-Yb are not stable in benzene solution and immediately lose their intense dark-brown colour. ^1^H NMR analyses suggest reductive cleavage of the benzene C–H bond and formation of previously reported (BDI*)MgPh^18^ and (Am*)MH complexes (M = Ca, Yb)^34,35^ (Fig. S43). The ^1^H NMR spectrum of 1-Yb in C_6_D_12_ (Fig. S6) shows close similarities to that of 1-Ca, revealing sharp resonances with clear spin coupling. This confirms its diamagnetic nature and the Yb^II^ oxidation state. The only broadened signals are assigned to the DIPP-substituents of the Am* ligand. Two sets of resonances are observed showing that the Am* ligand in 1-Yb is also *N-*aryl-chelating in solution. Broadening is due to slow exchange, but because of thermal instability of 1-Yb, we were unable to reach coalescence by heating. In C_6_D_12_, 1-Yb decomposes over time (Fig. S37; 24 h: 50% decomposition and 4 days: full decomposition). The complex is significantly less stable than 1-Ca, which required 10 days for full decomposition. Its decomposition is also less selective and at least 3–4 different products are formed. Although we could not crystallize the major decomposition product, we propose a similar decomposition route to that for 1-Ca. This involves insertion of Mg^0^ in a benzylic C–H bond and formation of a complex with a bridging hydride ligand (Scheme 2). Strong evidence for this complex is provided by comparison of the ^1^H NMR data of the decomposition products of 1-Yb with those reported for 1-Ca. These spectra show close similarities except for the resonance of the bridging hydride (Fig. S38). This is a singlet at 2.57 ppm for the Mg–H–Ca unit but is considerably downfield shifted for Mg–H–Yb (5.16 ppm). A similar shift of the hydride resonance to higher ppm values has previously been observed for Yb hydride complexes and is due to the close vicinity of the heavy metal Yb nucleus.^36^ As the ^171^Yb nucleus (14% abundance, *I* = 1/2) is NMR active, the hydride signal also shows weak satellites with ^1^*J*_Yb–H_ coupling of 252 Hz (Fig. S37). However, we have not been able to detect the ^171^Yb NMR signal which is often a problem for ytterbium hydride complexes.^36,37^

### Computational analysis

Complex 1-Yb was optimized at the same level of theory as previously reported for 1-Ca (B3PW91-D3BJ/def2-TZVP//B3PW91-D3BJ/def2-SVP), using a Stuttgart–Dresden (SDD) effective core potential for the heavy Yb atom. The calculated bond distances are generally in good agreement with the experimental values (Fig. S66), except for the Mg–Yb bond, which is calculated to be somewhat shorter (3.241 Å *versus* 3.302 Å). Such discrepancies of 0.05–0.10 Å are commonly observed for metal–metal bonds.^38^

Atoms-In-Molecules (AIM) analysis of 1-Yb (Fig. 2a) shows a weak Mg–Yb bond path with a bond-critical-point (bcp). Similar to 1-Ca, a Non-Nuclear-Attractor (NNA) could not be located and the electron density in the Mg–Yb bond is polarized towards the Mg centre. The electron density and Laplacian in the bcp are similar to that calculated for the Mg–Ca complex (1-Yb: *ρ*(*r*) = 0.023, ∇^2^*ρ*(*r*) = −0.002; 1-Ca: *ρ*(*r*) = 0.022, ∇^2^*ρ*(*r*) = −0.001; values in a.u.). These low values indicate weak covalent interactions.

**Fig. 2:**
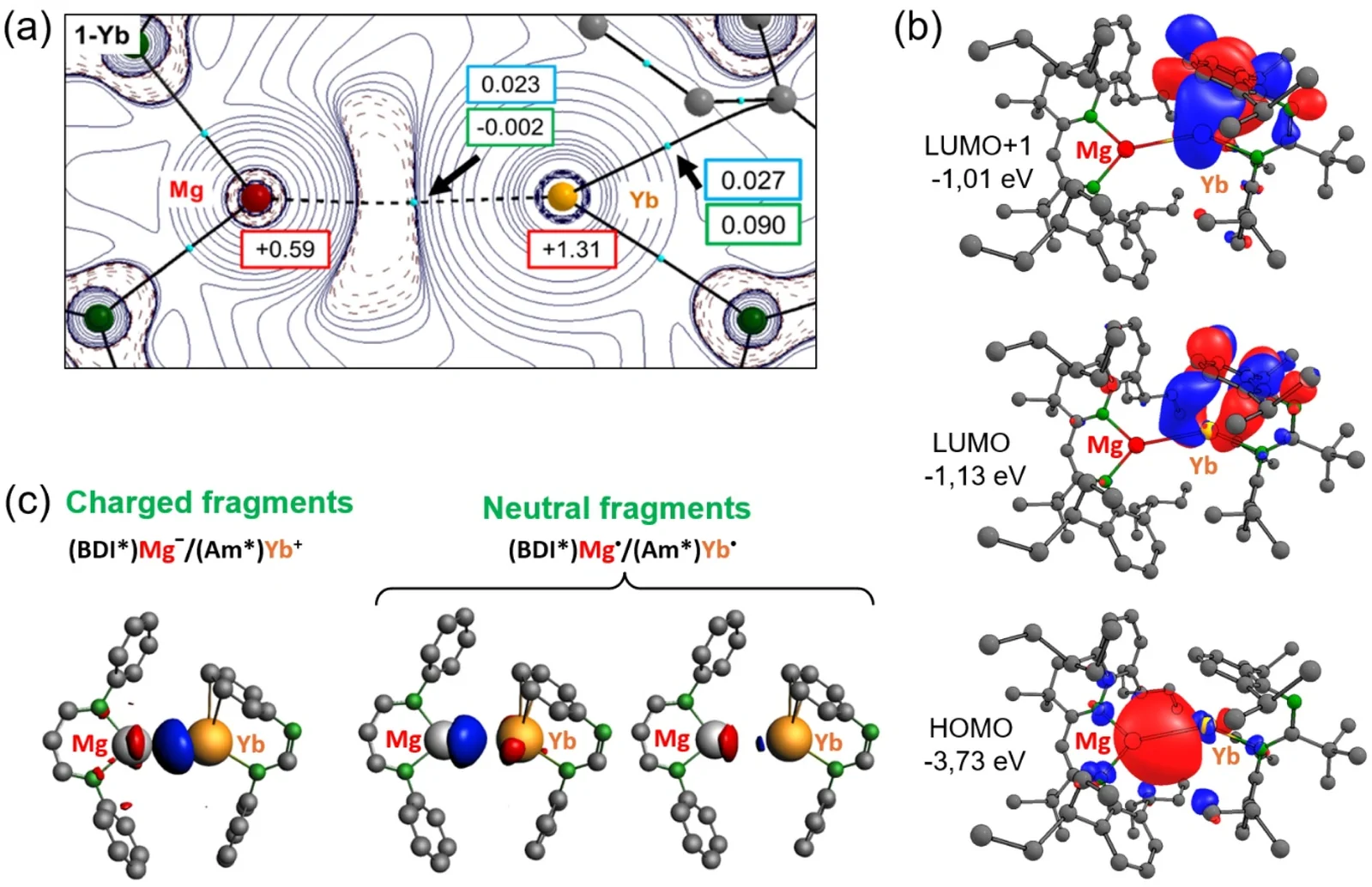
(a) Atoms-In-Molecules (AIM) analysis. Laplacian distribution for 1-Yb. Light-blue dots: bond-critical-points (bcps). (b) HOMO, LUMO and LUMO+1 in 1-Yb. (c) EDA-NOCV analysis for a truncated version of (BDI*)Mg–Yb(Am*) (1-Yb) using either charged closed-shell fragments or neutral open-shell fragments. The direction of the charge flow is red → blue. For charged fragments, the major NOCV deformation densities are characterized by a flow of electron density from Mg^0^ to Yb^II^ (Δ*E*_orb_ = −21.8 kcal mol^−1^, |v1| = 0.694). For neutral fragments, electron density flows primarily from Yb^I^ to Mg^I^ (Δ*E*_orb_ = −20.1 kcal mol^−1^, |*v*1| = 0.517) and there is a smaller flow from Mg^I^ to Yb^I^ (Δ*E*_orb_ = −8.9 kcal mol^−1^, |*v*2| = 0.294).

As pointed out previously,^22,39^ NPA charge analysis for the metal in Yb^II^ complexes consistently shows considerably smaller values than for analogous Ca^II^ complexes. This is also the case for 1-Ca and 1-Yb (Table S4). This deficiency is likely related to the use of relativistic effective core potentials in the calculations of Yb complexes. The atomic charges calculated using AIM, which are based on a partitioning of the electron density, provide a much better comparison between 1-Yb (Mg +0.59 and Yb +1.31) and 1-Ca (Mg +0.49 and Ca +1.41). The AIM charge of +1.31 on Yb in 1-Yb is the lowest value calculated for any existing Yb^II^ complex. For comparison, the AIM charges in II-Yb are Mg +0.47 and Yb +1.46. This indicates that the transfer of electron density from the electron-rich Mg^0^ centre to Yb^II^ in 1-Yb is even larger than in II-Yb. Although 1-Yb is formally a Mg^0^/Yb^II^ complex, its electron density suggests partial Mg^I^/Yb^I^ character. This is in agreement with the experimentally observed geometry (*vide supra*).

The HOMO in 1-Yb (Fig. 2b) corresponds to a σ-bond along the Mg–Yb axis with a major contribution from s-orbitals on Mg and Yb (Mg: 3s 33%, 3p 20%; Yb: s 19%, p 11%, d 5%). The HOMO−1 to HOMO−7 are mainly represented by the seven f-orbitals located on Yb (Yb: f > 95%). The LUMO and LUMO+1 are represented by a combination of a Yb d-orbital, contributing 12% and 13%, respectively, and the π*-system of the coordinating aryl ring (45% and 57%, respectively), with additional minor contributions from a Mg p-orbital in the LUMO (5%) and from a Yb s-orbital in the LUMO+1 (6%).

Effective-Oxidation-State (EOS) analysis derives oxidation states from first principles.^40^ This method indicates that 1-Yb can be best described as a Mg^0^/Yb^II^ complex, in agreement with the formal oxidation states (Table S6 and Fig. S67). However, a low reliability index of 68.0% indicates that these assigned oxidation states are not fully representative of the electron distribution described by the wavefunction. This is consistent with the previously discussed transfer of electron density from Mg^0^ to Yb^II^ and closely resembles the EOS analysis on 1-Ca.^30^

Energy Decomposition Analysis combined with Natural Orbitals for Chemical Valence (EDA-NOCV) calculations (Fig. 2c) on a truncated model system for 1-Yb showed similar results to those for 1-Ca. Two different fragmentations were evaluated: charged closed-shell fragments (BDI*)Mg^−^/(Am*)Yb^+^ and neutral open-shell radical ions (BDI*)Mg˙/(Am*)Yb˙ (Table S8). The total interaction energy is significantly larger for the charged fragments (Δ*E*_int_ = −155.5 kcal mol^−1^) than for the neutral fragments (Δ*E*_int_ = −61.6 kcal mol^−1^). This is mainly due to a larger electrostatic contribution for the charged fragments (Δ*E*_elstat_ = −178.9 kcal mol^−1^) *versus* that for neutral fragments (Δ*E*_elstat_ = −79.1 kcal mol^−1^). The orbital interaction energy is also considerably larger for the charged fragments (Δ*E*_orb_ = −53.7 kcal mol^−1^) than for the neutral fragments (Δ*E*_orb_ = −32.8 kcal mol^−1^). According to EDA convention, the partitioning giving the smallest absolute Δ*E*_orb_ represents the most realistic description for the bonding interaction. This means that bonding in 1-Yb is best pictured as a combination of neutral open-shell radicals (BDI*)Mg˙/(Am*)Yb˙. However, as there are exceptions to this rule,^41^ this conclusion should be treated with caution. A similar situation was found in the EDA-NOCV analysis for the Ca analogue 1-Ca.^30^

### Reactivity

We recently reported preliminary results on the reactivity of 1-Ca.^30^ Herein, we extend these reactivity studies and provide a direct comparison with the reactivity of 1-Yb (Scheme 2).

Solutions of both complexes in methylcyclohexane-*d*_14_ react rapidly with H_2_ already at −80 °C, underscoring the extreme reactivity of these mixed-metal low-valent complexes. For comparison, the well-known (BDI)Mg–Mg(BDI) complexes only react with H_2_ at increased temperatures.^9^ Unfortunately, the reactions of 1-Yb and 1-Ca with H_2_ are fully unselective and even at low temperatures we could not isolate any defined products.

Both complexes 1-Yb and 1-Ca also reacted fast, but unselectively, with CO and CO_2_ to yield a complex mixture of products. In contrast, Mg^I^ complexes reacted selectively with CO and CO_2_.^42,43^ Similarly, (BDI)Ca^I^ synthons also reacted selectively with CO_2_ at low temperatures, yielding well-defined carbonates as the major product.^44^

While reaction of 1-Ca with I_2_ selectively produced a mixture of BDI*MgI and Am*CaI,^30^ the oxidation of 1-Yb with I_2_ was unselective. The formation of paramagnetic species showed that complex 1-Yb was overoxidized to undefined Yb^III^ species. The possibility of additional oxidation of the lanthanide metal complicates reactivity studies of this Mg^0^/Yb^II^ complex. This represents the most significant difference in the reactivity of 1-Yb and 1-Ca.

Stasch and coworkers showed that (BDI)Mg–Mg(BDI) complexes can be selectively oxidized with N_2_O to give a soluble form magnesium oxide: (BDI)Mg–O–Mg(BDI).^45^ The ability to deprotonate H_2_ supports the very high reactivity of such molecular oxide complexes.^45^ We showed that a similar isolation of a molecular Ca oxide complex could only be achieved by the solid-state conversion of a Ca^I^ synthon with N_2_O gas.^46^ This afforded a powder of a (BDI)Ca–O–Ca(BDI) complex which despite rapid decomposition in solution could be crystallized fast in a low yield of 27%. The relative stability of a (BDI)Mg–O–Mg(BDI) complex compared with the instability of an analogous (BDI)Ca–O–Ca(BDI) complex, motivated us to attempt the synthesis of the mixed metal (BDI*)Mg–O–Ca(Am*) complex.

Reaction of a solution of 1-Ca in methylcyclohexane with excess N_2_O at −70 °C led to an immediate change of colour from dark-brown to yellow. ^1^H NMR of the reaction mixture indicated the formation of several products (Fig. S51). Also, the reaction of solid crystalline 1-Ca with excess N_2_O gas gave a similar mixture of products. Cooling the crude mixture of products to −35 °C led to formation of a few colourless crystals (circa 5%) suitable for X-ray diffraction. This showed the formation of 3-Ca (Scheme 2) in which one of the terminal CH_3_ substituents in the BDI* ligand has been deprotonated by the oxide dianion (Fig. 1c), demonstrating the very high reactivity of soluble complexes with low-coordinate O^2−^ anions. A similar reaction of 1-Yb with excess N_2_O led to further oxidation of Yb^II^ and formation of multiple unidentified paramagnetic Yb^III^ products (Fig. S52). Reaction with exactly one equivalent of N_2_O gas led to partial conversion of 1-Yb and formation of the same paramagnetic product(s). This shows that the oxidations of Yb^II^ and Mg^0^ by N_2_O are of similar rates.

It was found that the Yb metal in 1-Yb can be replaced with Ca by the reaction of 1-Yb with 0.5 equivalents of (Am*CaI)_2_ (2-Ca). However, the reaction gave an equilibrium mixture of 1-Yb, 1-Ca, 2-Yb and 2-Ca (Fig. S50). This underscores the weak nature of the Mg–Ca and Mg–Yb bonds.

As reported for 1-Ca,^30^ a solution of 1-Yb in methylcyclohexane reacted at room temperature quantitatively within two hours with 1,1-diphenylethylene (DPE) giving essentially pure (BDI*)Mg(CH_2_CPh_2_)Yb (Am*) in 94% isolated yield. Single crystals of 4-Yb were grown from a mixture of pentane and THF. The crystal structure of 4-Yb (Fig. 3a) shows the formation of a product with a (BDI*)Mg–CH_2_CPh_2_ bond. This confirms the nucleophilic nature of Mg. The (Am*)Yb fragment is not bound to carbon but instead coordinates to one of the phenyl rings in CH_2_CPh_2_^2−^ (Yb–C: range = 2.712(4)–2.480(4) Å; shortest contact is Yb–C_para_ = 2.712(4) Å). The C–C bond in CH_2_CPh_2_^2−^ is elongated to 1.514(5) Å and shows a single-bond character whereas one of the C–Ph bonds is very short (1.393(5) Å) and displays a double-bond character. The phenyl ring connected to Yb shows characteristics similar to a “Meisenheimer anion” in which the negative charge is delocalized over C_ortho_ and C_para_ positions. The ^1^H NMR spectrum of 4-Yb in cyclohexane-*d*_12_ shows three very broad signals in a 2 : 2 : 1 ratio (5.15–5.85 ppm) which are assigned to the phenyl rings (Fig. S11). This pronounced high-field shift is indicative of loss of aromaticity. As both phenyl rings give one set of signals, there is fast exchange between the coordinating (non-aromatic) Ph ring and the non-coordinated (aromatic) Ph ring. It should also be noted that the presence of THF has a pronounced effect on the coordination mode of the Am* ligand. Without THF the ligand binds *N-*aryl-chelating, *e.g.* in 1-Yb or in the recently reported THF-free Ca analogue of 4-Yb.^30^ With two additional THF ligands, the Am* ligand switches to *N,N*-coordination, *e.g.* in 4-Yb. Note that the reaction of a (BDI)Mg–Mg(BDI) complex with DPE led to formation of (BDI)Mg–H_2_CC(Ph_2_)–Mg(BDI) in which both Mg atoms form Mg–C single bonds.^47^ Preference of the larger Ca^2+^ or Yb^2+^ cations for Ph-coordination can be explained by the hard–soft acid–base (HSAB) principle.^48^

**Fig. 3:**
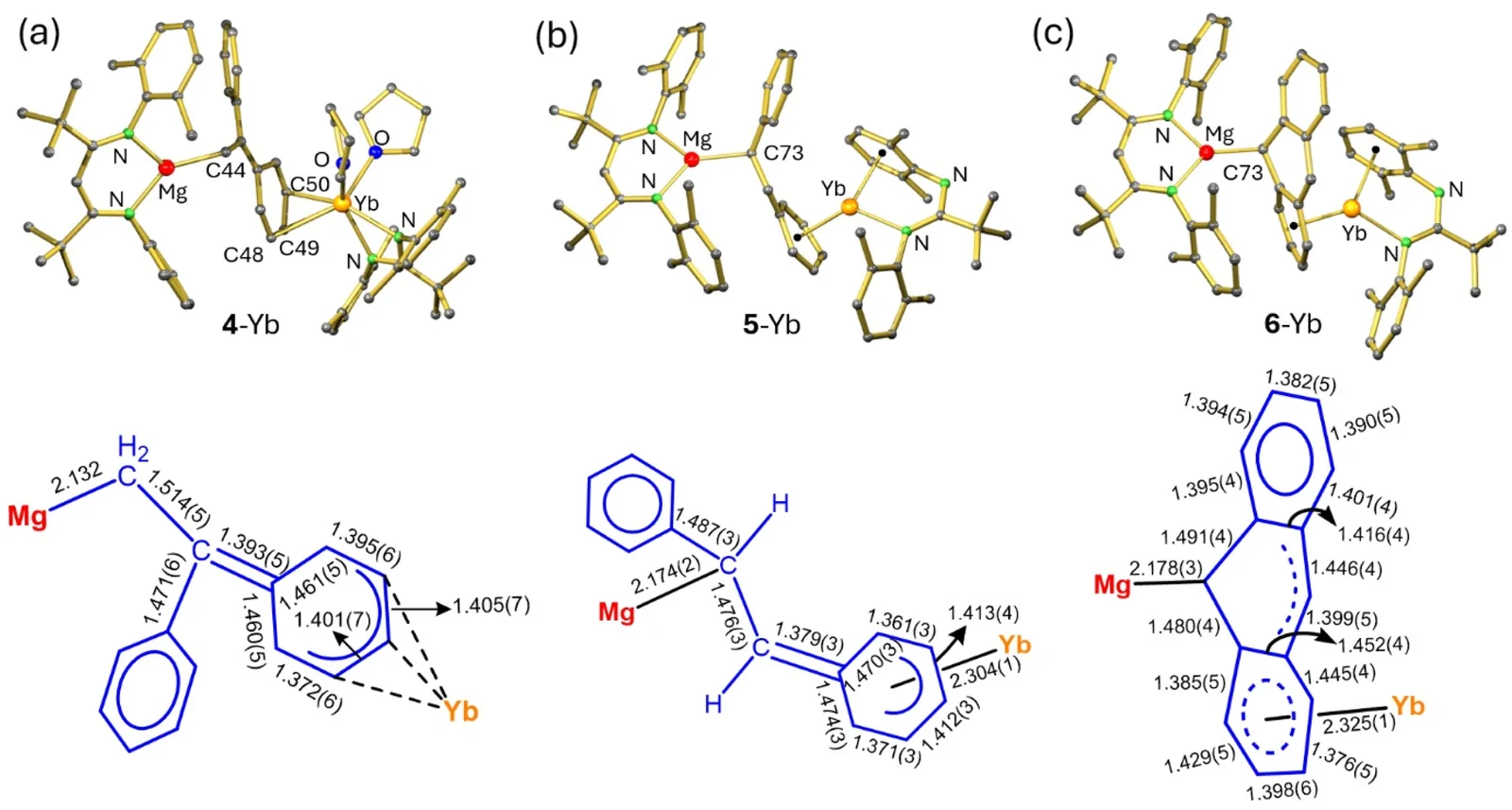
(a) Crystal structure of 4-Yb and selected bond distances in the Mg–CH_2_CPh_2_–Yb unit. (b) Crystal structure of 5-Yb and selected bond distances in the Mg–CH(Ph)C(H)Ph–Yb unit. (c) Crystal structure of 6-Yb and selected bond distances in the Mg–(anthracene)–Yb unit. All H atoms, Et groups on BDI* and Me groups on Am* have been omitted for clarity.

Reaction of 1-Yb with *trans*-stilbene afforded a similar product (5-Yb) but with slightly different connectivity (Fig. 3b). Again, the smaller and harder Mg^2+^ prefers Mg–CH(Ph)CHPh coordination, whereas the softer Yb^2+^ shows (*η*^6^)Ph⋯Yb coordination (Yb–Ph_centroid_ = 2.304(1) Å). The analogous complex 5-Ca could also be isolated and crystallized isostructural with Mg–C and (*η*^6^)Ph⋯Ca coordination (Ca–Ph_centroid_ = 2.312(1) Å). The contact between a formally negatively charged Ph ring and Yb^2+^ (or Ca^2+^) in 5-Yb/Ca (Yb–Ph_centroid_ = 2.304(1) Å; Ca–Ph_centroid_ = 2.312(1) Å) is considerably shorter than the Yb⋯Ar_centroid_ contact in 2-Yb (average: 2.429 Å) which involves a neutral aryl ring.

The bridging mode of the *trans*-stilbene^2−^ dianion in 5-Yb/Ca differs from that in (BDI)Mg–(stilbene)–Mg(BDI),^49^ (Am*)Ca–(stilbene)–Ca(Am*)^50^ or (Am*)Yb–(stilbene)–Yb(Am*).^34^ As pointed out previously,^50^ the bridging mode in the M–(stilbene)–M moiety depends on the nature of the metal (Scheme 3). Electropositive metals (Li, Na, K, and Ca) produce highly ionic M–stilbene bonds which prefer a planar delocalized Ph(H)CC(H)Ph^2−^ dianion with short C=C and Ph–C bonds and sp^2^-hybridization of the central C atoms. The negative charge is mainly on the Ph rings which also coordinate to the metals. Less electropositive metals (Mg, Be, and Al) results in the formation of more covalent M–stilbene bonds which prefer a Ph(H)C–C(H)Ph fragment with long C–C and Ph–C bonds and sp^3^-hybridization of the central C atoms. Due to significant covalency, there is only a small negative charge on the stilbene unit which is mainly on the central C atoms that are bound to the metals. The bonding situation in the heterobimetallic complexes 5-Yb/Ca is new and represents a hybrid of these two situations (Fig. 3b).

**Scheme 3:**
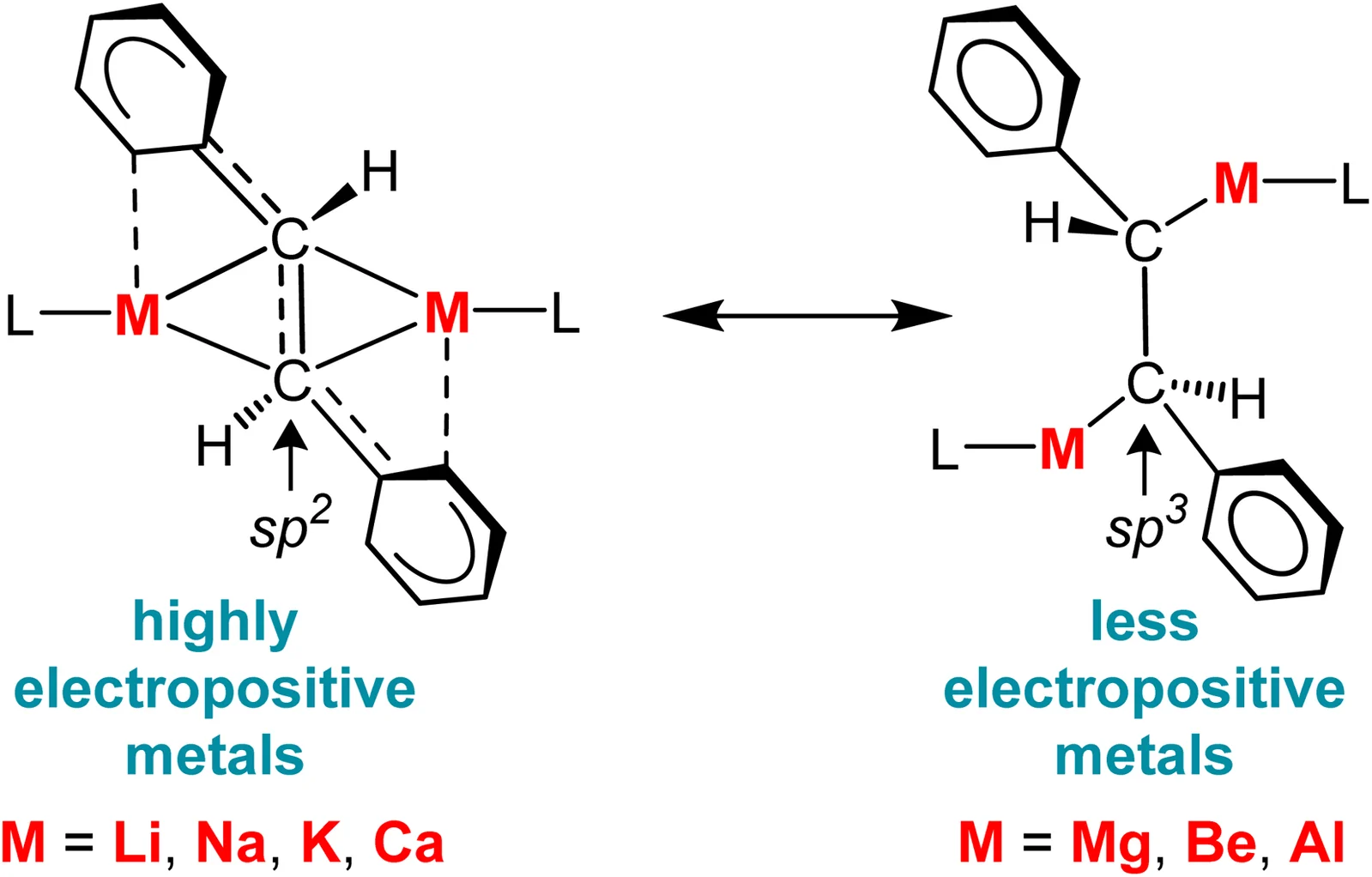
Metal complexes with a bridging stilbene^2−^ dianion. Dependence of geometry and hybridization on the nature of the metal.

The ^1^H NMR spectra of 5-Yb and 5-Ca show very similar features, but slightly different dynamics for exchange of the phenyl rings in the stilbene^2−^ dianion (Fig. S16 and S22). The ^1^H NMR signals for the Ph groups are in both cases broadened. Heating a solution of 5-Yb in methylcyclohexane-*d*_14_ to +80 °C results in fast exchange and appearance of three signals in a 2 : 2 : 1 ratio between 4.81 and 5.67 ppm (Fig. S21). This also confirms the remarkable thermodynamic stability of 5-Yb up to 80 °C without any decomposition or reversible formation of starting materials 1-Yb and *trans*-stilbene. Such a reductive elimination process was observed for (BDI)Mg–(stilbene)–Mg(BDI).^49^ This underscores the very high reducing power of heterobimetallic Mg–Ca/Yb complexes.

Cyclohexane solutions of 1-Yb and 1-Ca also reacted smoothly with anthracene and overnight selectively afforded 6-Yb and 6-Ca, respectively. The isostructural crystal structures show that the small Mg^2+^ cation is connected covalently with the C in the anthracene 9-position whereas the larger, more electropositive Yb^2+^/Ca^2+^ cations bind ionically to one of the terminal C_6_-rings *via* (*η*^6^)ring-Yb/Ca interactions (Fig. 3c and S63). This binding mode of reduced anthracene is somewhat similar to that in a (BDI*)Mg–(anthracene)–Ca(BDI) complex in which Mg bridges the 9,10-positions and Ca binds to one of the terminal C_6_-rings.^51^ The ^1^H NMR spectra of 6-Yb and 6-Ca are quite similar and show one broadened signal for the bridgehead 9,10-anthracene positions and one set of two broadened signals (1 : 1 ratio) for the two flanking C_6_-rings (Fig. S27–S32). This indicates that there is in solution fast exchange between the 9, 10-positions and the flanking rings.

Ammonia activation is well known with transition metal and p-block metal complexes,^52–56^ but not explored for low-oxidation-state s-block metal complexes. A dark-red solution of 1-Ca in methylcyclohexane reacts at −80 °C rapidly with NH_3_(g) to give a light-yellow solution from which the heterobimetallic Mg–Ca amide complex 7-Ca was crystallized in 43% yield. The presence of two bridging NH_2_^−^ anions in the crystal structure (Fig. 1d) is consistent with activation of two equivalents of NH_3_. We assume a mechanism in which Mg^0^ inserts in the N–H bond, a formal oxidative addition which forms the intermediate complex (BDI*)Mg–(µ_2_-H)(µ_2_-NH_2_)–Ca(Am*). Similar intermediates with M–(µ_2_-H)(µ_2_-NH_2_)–M cores (M = Ca, Al, Ru, Ir) have been reported.^56–58^ The reactive hydride ligand in this intermediate deprotonates a second NH_3_ molecule, resulting in H_2_ release and formation of 7-Ca. Following the reaction at low temperatures indeed suggests the intermediate formation of (BDI*)Mg–(µ_2_-H)(µ_2_-NH_2_)–Ca(Am*) (Fig. S45).

The Mg–N and Ca–N bond lengths in 7-Ca are in the normal range for Mg^II^–N and Ca^II^–N bond distances. *N-*aryl-coordination of the amidinate ligand is maintained in solution. The two bridging NH_2_^−^ anions show ^1^H NMR signals at high field (−1.90 ppm, Fig. S44). This is in accordance with previously reported polar Al–NH_2_ (−1.79 ppm)^56^ and Ca–NH_2_ (−1.73 ppm)^57^ amide complexes.

A solution of complex 1-Yb in methylcyclohexane also reacted rapidly with NH_3_(g) at −80 °C. The immediate colour change from dark-brown to pale yellow indicated the oxidation of 1-Yb. This was confirmed by ^1^H NMR (Fig. S53–S56) which showed full consumption of 1-Yb. However, all signals are considerably broadened suggesting further conversion to Yb^III^ species. This was followed by rapid precipitation of an off-white product that is fully insoluble in THF but can be quenched with methanol under release of ammonia. Since the precipitate does not contain BDI* or Am* ligands, we propose the formation of insoluble Yb(NH_2_)_3_. As the synthesis of YbI_2_ can be carried out in liquid NH_3_,^59^ the oxidation of 1-Yb to an Yb^III^ product by reaction with NH_3_ may be unexpected. However, there is precedence for such redox reactivity^60^ and it is also known that Yb metal can react in liquid ammonia to Yb(NH_2_)_3_.^61^

## Conclusion

It was recently demonstrated that the magnesyl anion (BDI*)Mg^−^ in [(BDI*)Mg^−^Na^+^]_2_ can function as an electron-donating L-type ligand for Yb (II-Yb). In the present work, a salt-metathesis procedure involving the reaction of the sodium salt [(BDI*)Mg^−^Na^+^]_2_ and (Am*YbI)_2_ resulted in formation of (BDI*)Mg–Yb(Am*) (1-Yb) and NaI. Complex 1-Yb, in which (BDI*)Mg˙ acts as an X-type ligand for Yb (Am*), crystallizes isomorphous to (BDI*)Mg–Ca(Am*) (1-Ca). The crystal structures of 1-Yb and 1-Ca fit quite well on each other.

The formal oxidation states in 1-Yb are Mg^0^–Yb^II^. This is confirmed by effective-oxidation-state analysis. However, a low reliability index of 68.0% indicates that this assignment is not fully representative of the electron distribution. Charge analysis shows significant transfer of electron density from the electron rich Mg^0^ centre to Yb^II^. Therefore, the complex has partial Mg^I^–Yb^I^ character. According to energy decomposition analysis, bonding in 1-Yb is best understood as a combination of neutral open-shell radicals (BDI*)Mg˙/(Am*)Yb˙.

Due to electronegativity differences, the metal–metal bond in 1-Yb is polarized: Mg^*δ*−^–Yb^*δ*+^. AIM charges for the metal atoms in 1-Yb (Mg +0.59 and Yb +1.31) compare well to those in 1-Ca (Mg +0.49 and Ca +1.41). This bond polarization makes the complexes highly reactive and unstable. In solution, 1-Yb slowly decomposes by insertion of the Mg^0^ centre into a benzylic C–H bond of one of the Et_2_CH-substituents. Such C–H bond activation by oxidative addition is normally associated with transition metals, but is now also well established for main group elements in low oxidation states. In comparison to unpolarized Mg–Mg bonds, the polarized Mg^*δ*−^–Yb^*δ*+^ bond is highly reactive.

Whereas the structures of 1-Yb and 1-Ca and their electron distributions are comparable, their reactivities partially differ. Oxidation of 1-Ca with N_2_O gave an unstable Mg–O–Ca complex which decomposed by deprotonation of the terminal CH_3_ group in the Et_2_CH-substituent by the O^2−^ dianion (3-Ca). In contrast, the same reaction with 1-Yb led to overoxidation and formation of an unknown paramagnetic Yb^III^ species. This highlights an important difference between the Mg–Ca and Mg–Yb systems: the additional redox accessibility of the lanthanide centre can complicate the reactivity of 1-Yb.

This is also the case for reactions of 1-Yb with gaseous NH_3_. Whereas 1-Ca reacted already at −80 °C to form a dimeric complex with bridging NH_2_^−^ anions (7-Ca), the reaction of 1-Yb under similar conditions resulted in overoxidation to Yb^III^ species and precipitation of an insoluble salt which is likely Yb(NH_2_)_3_.

Reactions of 1-Ca and 1-Yb with extended π-systems (1,1-diphenylethylene, *trans*-stilbene or anthracene) gave the expected products with dianionic bridging ligands [Ph_2_CCH_2_^2−^], [Ph(H)CC(H)Ph^2−^] and [anthracene^2−^], respectively, which have been fully characterized by X-ray diffraction. In these complexes, the small and hard Mg^2+^ generally forms covalent Mg–C σ-bonds, whereas the larger and softer Ca^2+^ (or Yb^2+^) cations preferentially bind to the softer π-system of one of the six-membered rings. Overall, these studies show that unsupported and polarized Mg–Ca and Mg–Yb bonds are exceptionally reactive platforms for small-molecule activation and reduction of unsaturated organic substrates.

Although challenging, we continue our research with attempts to isolate complexes with even more labile and reactive Mg–Sr or Mg–Ba bonds.

## Author contributions

S. K. Thakur: conceptualization, investigation, validation, formal analysis, writing – original draft, visualization. T. Tewari: investigation, validation, formal analysis. N. Roig: investigation, validation, formal analysis. J. Langer: formal analysis, validation. M. Alonso: investigation, validation, formal analysis, supervision, project administration. S. Harder: conceptualization, writing – original draft – review and editing, visualization, validation, supervision, project administration.

## Conflicts of interest

There are no conflicts to declare.

## Supplementary Material

SC-OLF-D6SC05592C-s001

SC-OLF-D6SC05592C-s002

## Data Availability

CCDC 2566191–2566199 contain the supplementary crystallographic data for the complexes.^62*a*–*i*^ Supplementary information (SI): synthetic procedures, selected NMR spectra, details of crystal structure determination, details of computational work, experimental details of reactivity studies, ^1^H and ^13^C NMR spectra, crystallographic details including ORTEP presentations, and details of the DFT calculations including XYZ-files. See DOI: https://doi.org/10.1039/d6sc05592c.
