# Supplementary material for: Comparison of Mg–Yb and Mg–Ca bonding: structures, electronic analyses and reactivities
Source: Chem Sci. 2026 Sep 21. Online ahead of print. doi: 10.1039/d6sc05592c (PMC13636282; doi:10.1039/d6sc05592c)
Supplement: Supplementary file 1 [file SC-OLF-D6SC05592C-s001.pdf]

Supplementary Information (SI) for Chemical Science.
This journal is © The Royal Society of Chemistry 2026

Supporting Information

Table of Contents

1. Materials and methods S2

2. Complex syntheses and reactivity S3

3. Spectroscopic data S13

4. Crystal structure determination S41

5. DFT Calculations S50

6. References S64

1. Materials and methods

All experiments were conducted in dry glassware under an inert nitrogen atmosphere by applying standard Schlenk techniques or gloveboxes (MBraun) using freshly dried and degassed solvents. All solvents were degassed with nitrogen, dried over activated aluminum oxide (Innovative Technology, Pure Solv 400-4-MD, Solvent Purification System), and then stored under inert atmosphere over molecular sieves (3 Å) unless noted otherwise. Deuterated benzene (C_6D_6), cyclohexane-d_{12} and methylcyclohexane-d_{14} were purchased from Sigma Aldrich or Deutero GmbH, degassed and dried over molecular sieves (3 Å). 1,1-diphenylethylene, *trans*-1,2-diphenylethylene and anthracene were purchased from Sigma-Aldrich and used as received. The following compounds were synthesized according to literature procedures: $[(BDI^*)MgNa]_2$[1] and Am*K[2] (BDI* = HC[C(*t*Bu)N-DIPeP]$_2$; DIPeP = 2,6-CHEt$_2$-phenyl), Am* = *t*BuC(N-DIPP)$_2$, DIPP = 2,6-*i*Pr-phenyl.

NMR spectra were measured on Bruker Avance III HD 400 MHz and Bruker Avance III HD 600 MHz spectrometers. Chemical shifts (δ) are denoted in ppm (parts per million) and coupling constants in Hz (Hertz). For describing signal multiplicities common abbreviations are used: s (singlet), d (doublet), t (triplet), q (quartet), p (quintet), sept (septet), m (multiplet) and br (broad). Spectra were referenced to the solvent residual signal. Assignments of resonance signals in the ^1H and $^{13}C\{^1H\}$ NMR spectra were made based on two-dimensional NMR correlation experiments (HSQC, HMBC, COSY). Elemental analyses were performed with an Hekatech Eurovector EA3000 analyzer.

Note: In most of the compounds, the obtained elemental analyses values are not within the 0.4% error range. This holds especially for the C-content values which are often too low because of formation of metal carbide (despite the addition of strong oxidizing agents). Another factor is that the DIPeP substituents make the complexes extremely soluble, even in alkanes. Hence, it is very difficult to crystalize them in good yields and wash out the impurities from crystals. The elemental analyses data is provided to illustrate the best values obtained. The recent publication also shows that elemental analyses method has a larger error range than the journal guidelines.[3]

The crystal structure data has been deposited with the Cambridge Crystallographic Data Centre. CCDC 2566191-2566199 contain the supplementary crystallographic data for the complexes. This data can be obtained free of charge from The Cambridge Crystallographic Data Centre via www.ccdc.cam.ac.uk/data_request/cif.

2. Complex syntheses and reactivity

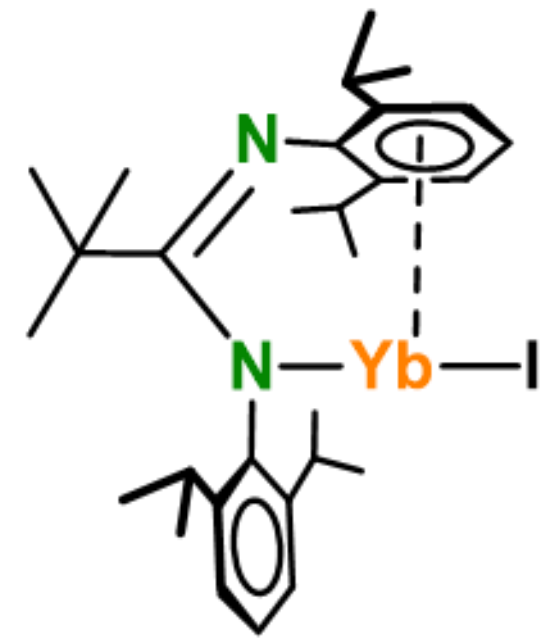

Synthesis of [Am*YbI]$_2$ (**2**-Yb). Am*K (459 mg, 1.00 mmol, 1.0 eq.) was dissolved in diethyl ether (5.0 mL) and cooled to -78°C. The cooled solution was then added dropwise to a suspension of YbI_2 (512 mg, 1.20 mmol, 1.2 eq.) in diethyl ether (5.0 mL) at -78 °C. The reaction mixture was slowly warmed to room temperature and stirred for 2 h. The resulting suspension was filtered to remove KI and excess of YbI_2 and solvent was removed in *vacuo* to afford an orange solid. The ^{1}H NMR spectrum of the crude sample showed contamination of the product with 5-10 % protonated ligand Am*H. The crude powder was washed with pentane (3 x 2 mL). This gave essentially pure and diethyl ether free [Am*YbI]$_2$ (**2**-Yb) (503 mg, 0.35 mmol, 70 %). Crystallization of the raw product from diethyl ether at -35 °C gave crystals of [Am*YbI]$_2$ (**2**-Yb) that are suitable for X-ray diffraction.

^{1}H NMR (C_6D_6, 600 MHz, 298 K): δ = 7.24 (br s, 2**H**, CH_{arom}), 7.09 (br s, 2H, C**H**$_{arom}$), 7.02 (br s, 1H, C**H**$_{arom}$), 6.25 (br s, 1H, C**H**$_{arom}$), 3.34 (br s, 2H, C**H**-*i*Pr), 3.15 (br s, 2H, C**H**-*i*Pr), 1.46 (s, 9H, C**H**$_3$, *t*Bu), 1.33-1.24(m, 24H, C**H**$_3$ *i*Pr) ppm.

^{13}C{^{1}H} NMR (C_6D_6, 600 MHz, 298 K): δ = 172.0 (**C**, *t*Bu), 160.0 (C, **C**$_{arom}$), 143.8 (C, **C**$_{arom}$), 142.6 (C, **C**$_{arom}$), 126.2 (C, **C**$_{arom}$), 124.4(C, **C**$_{arom}$), 123.4 (C, **C**$_{arom}$), 120.4 (C, **C**$_{arom}$), 42.04 (C, **C**Me$_3$), 31.6 (C, **C**H$_3$, *t*Bu), 29.0 (C, **C**H *i*Pr), 28.8 (C, **C**H *i*Pr), 27.7 (C, **C**H$_3$ *i*Pr), 23.9 (C, **C**H$_3$ *i*Pr), 20.5 (C, **C**H$_3$ *i*Pr) ppm.

Elemental analysis for $C_{58}H_{86}Yb_2I_2N_4$ (M = 1439.25 g mol^{-1}): Calc.: C 48.40%, H 6.02%, N 3.89%. Found: C 48.42%, H 6.10%, N 3.95%

Synthesis of (BDI*)Mg-Yb(Am*) (**1**-Yb). [(BDI*)MgNa]$_2$ (100 mg, 0.076 mmol, 1.0 eq.) and [Am*YbI]$_2$ (**2**-Yb) (109 mg, 0.076 mmol, 1.0 eq.) were dissolved in methylcyclohexane (5.0 mL) and the reaction mixture was stirred for 20 minutes at room temperature. The resulting red-brown suspension was filtered and the solvent was removed *in vacuo* giving an essentially pure brown powder. Crystals suitable for single crystal X-ray diffraction, were grown from a concentrated solution of pentane at -35 °C. Crystal yield: (176 mg, 0.143 mmol, 94%).

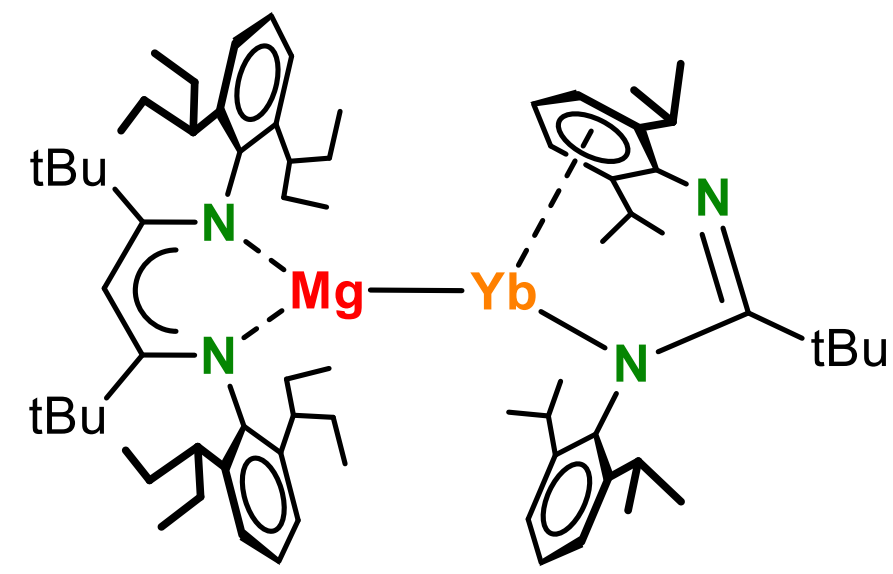

^{1}H NMR (Cyclohexane-*d$_{12}$*, 600 MHz, 298 K): δ = 6.96-6.93 (m, 2H, C**H**$_{arom}$), 6.91–6.89 (m, 4H, C**H**$_{arom}$), 6.81 (br, s, 3H, C**H**$_{arom}$), 6.71 (br s, 2H, C**H**$_{arom}$), 6.28 (br s, 1H, C**H**$_{arom}$), 5.08 (s, 1H, C**H**-backbone), 3.11 (br s, C**H**-*i*Pr), 2.95 (br s, C**H**-*i*Pr) 2.84 (br, s, 4H, C**H** *i*Pen), 1.80-1.74 (m, 4H, C**H**$_2$ *i*Pen), 1.67-1.58 (m, 12H, C**H**$_2$ *i*Pen), 1.18-1.12 (m, 12H, C**H**$_3$ *i*Pr), 1.10 (s, 9H, C**H**$_3$ *t*Bu Am*), 1.06 (s, 18H, C**H**$_3$ *t*Bu BDI*), 0.92 (t, $^3J_{HH}$ = 6 Hz, 12H, C**H**$_3$ *i*Pen), 0.87-0.86 (m, 6H, C**H**$_3$ *i*Pr), 0.82 (t, $^3J_{HH}$ = 6 Hz, 12H, C**H**$_3$ *i*Pen), 0.78-0.75 (br s, 6H, C**H**$_3$ *i*Pr) ppm.

^{13}C{^{1}H} NMR (Cyclohexane-*d$_{12}$*, 600 MHz, 298 K): δ = 173.9 (C, **C**N-backbone), 149.1(C, **C**$_{arom}$), 145.2(C, **C**$_{arom}$), 142.3 (C, **C**$_{arom}$), 140.2 (C, **C**$_{arom}$), 125.6 (C, **C**$_{arom}$), 124.0(C, **C**$_{arom}$), 123.2 (C, **C**$_{arom}$), 96.4 (C, **C**H-backbone), 44.2 (C, **C**Me$_3$, BDI*), 40.8 (C, **C**H *i*Pen), 36.3 (C, **C**Me$_3$, Am*), 33.7 (C, **C**H$_3$, *t*Bu BDI*), 31.7 (C, **C**H$_3$, *t*Bu Am*), 30.7 (C, **C**H$_3$ *i*Pr), 29.4 (C, **C**H *i*Pr), 28.2 (C, **C**H *i*Pr), 26.7 (C, **C**H$_2$ *i*Pen), 23.8 (C, **C**H$_2$ *i*Pen), 23.1 (C, **C**H$_3$ *i*Pr), 23.1 (C, **C**H$_3$ *i*Pr), 21.7 (C, **C**H$_3$ *i*Pr), 11.6 (C, **C**H$_3$ *i*Pen), 11.2 (C, **C**H$_3$ *i*Pen) ppm.

Elemental analysis for $C_{72}H_{112}MgYbN_4$ (M = 1231.07 g mol^{-1}): Calc.: C 70.25%, H 9.17%, N 4.55%. Found: C 68.41%, H 8.76%, N 4.22%. Although the C value is likely low due to metal carbide formation, it is provided to illustrate the best values obtained.

Synthesis of (BDI*)Mg-[H_2CCPh_2]-Yb(Am*)$(THF)_2$ (**4**-Yb). (BDI*)Mg-Yb(Am*) (**1**-Yb) (100.0 mg, 0.081 mmol, 1.0 eq.) and 1,1-diphenylethylene (DPE) (14.6 mg, 0.081 mmol, 1.0 eq.) were dissolved in cyclohexane (5 mL). After stirring the reaction mixture for 1 h at room temperature, the colour changed from red-brown to light brown. Analysis of the reaction mixture by NMR spectroscopy showed quantitative and clean conversion. Removal of the solvent in *vacuo* yielded essentially pure (BDI*)Mg-[H_2CCPh_2]-Yb(Am*) (107.4 mg, 0.076 mmol, 94%) as a light brown powder. Crystals suitable for single crystal X-ray diffraction, were grown by adding a few drops of THF to the concentrated pentane solution at -35 °C leading to the formation of THF adduct (BDI*)Mg-[H_2CCPh_2]-Yb(Am*)$(THF)_2$ (**4**-Yb).

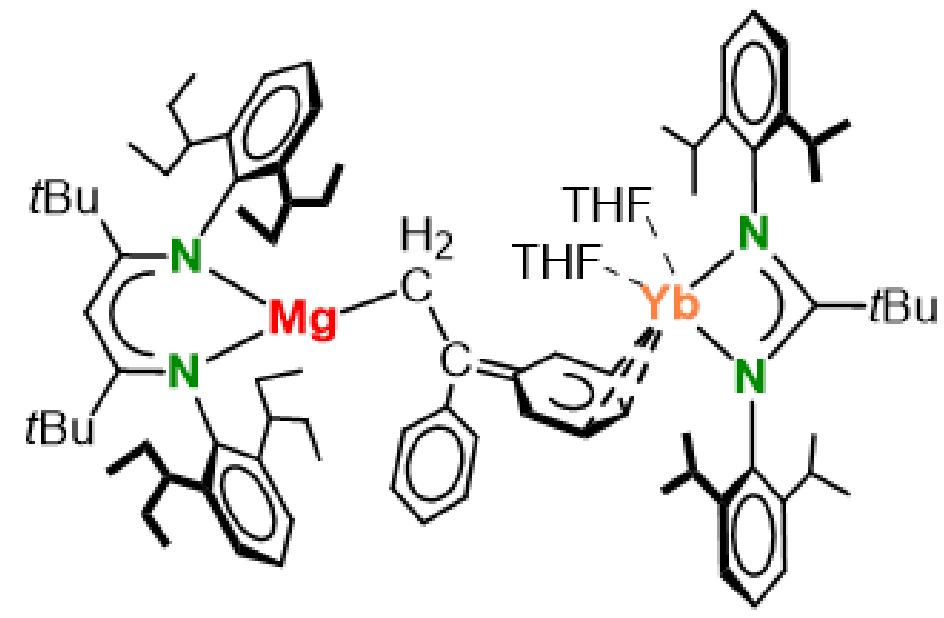

^{1}H NMR (Cyclohexane-d_{12}, 600 MHz, 298 K): δ = 6.99-6.97 (m, 2H, C**H**$_{arom}$), 6.92–6.91 (m, 4H, C**H**$_{arom}$), 6.71–6.69 (m, 2H, C**H**$_{arom}$), 6.63–6.61 (m, 4H, C**H**$_{arom}$), 5.83-5.61 (br s, 4H, C**H**$_{arom}$-DPE), 5.56-5.34 (br s, 4H, C**H**$_{arom}$-DPE), 5.30 (s, 1H, C**H**-backbone), 5.25-4.96 (br s, 2H, C**H**$_{arom}$-DPE), 3.21-3.14 (m, C**H**-*i*Pr), 2.98-2.91 (m, C**H**-*i*Pr) 2.82-2.75 (m, 4H, C**H** *i*Pen), 1.86-1.80 (m, 4H, C**H$_2$** *i*Pen), 1.61-1.55 (m, 12H, C**H$_2$** *i*Pen), 1.23-1.20 (m, 12H, C**H$_3$** *i*Pr), 1.17 (s, 9H, C**H$_3$** *t*Bu-Am*), 1.10 (s, 18H, C**H$_3$** *t*Bu BDI*), 1.08 (m, 6H, C**H$_3$** *i*Pr), 0.97-0.91 (m, 24H, C**H$_3$** *i*Pen), 0.48 (br, s, 6H, C**H$_3$** *i*Pr), 0.28 (br s, 2H, C**H$_2$**-DPE) ppm.

^{13}C{^{1}H} NMR (Cyclohexane-d_{12}, 600 MHz, 298 K): δ = 176.8 (C, **C**N-backbone-BDI*), 171.2 (C, **C**N-backbone-Am*) 158.3 (C, **C**$_{arom}$) 147.6 (C, **C**$_{arom}$), 147.0 (C, **C**$_{arom}$), 143.5 (C, **C**$_{arom}$), 141.9 (C, **C**$_{arom}$), 139.8 (C, **C**$_{arom}$), 128.9 (C, **C**$_{arom}$), 126.0 (C, **C**$_{arom}$), 124.1 (C, **C**$_{arom}$),123.2 (C, **C**$_{arom}$), 119.9(C, **C**$_{arom}$), 115.0 (C, **C**$_{arom}$), 96.7 (C, **C**H-backbone), 44.5 (C, **C**Me$_3$, BDI*), 42.7 (C, **C**Me$_3$, Am*) 41.2 (C, **C**H *i*Pen), 33.4 (C, **C**H$_3$ *t*Bu, BDI*), 32.0 (C, **C**H$_3$, *t*Bu Am*), 29.3 (C, **C**H *i*Pr), 28.7 (C, **C**H *i*Pr), 27.2 (C, **C**H$_2$ *i*Pen), 26.2 (C, **C**H$_2$ *i*Pen), 24.6 (C, **C**H$_2$ *i*Pen), 23.9 (C, **C**H$_3$ *i*Pr), 23.8 (C, **C**H$_3$ *i*Pr), 21.5 (C, **C**H$_3$ *i*Pr), 15.3 (**C**H$_2$-DPE), 12.0 (C, **C**H$_3$ *i*Pen), 10.9 (C, **C**H$_3$ *i*Pen) ppm.

Elemental analysis for $C_{86}H_{124}MgYbN_4$ (M = 1411.32 g mol^{-1}): Calc.: C 73.19%, H 8.86%, N 3.97%. Found: C 72.62%, H 8.78%, N 4.05.

Synthesis of (BDI*)Mg-[CH(Ph)CH(Ph)]-Yb(Am*) (**5**-Yb). In a J-Young NMR tube, (BDI*)Mg-Yb(Am*) (**1**-Yb) (40.0 mg, 0.032 mmol, 1.0 eq.) and trans-stilbene (5.8 mg, 0.032 mmol, 1.0 eq.) were dissolved in cyclohexane (1 mL). After stirring the reaction mixture overnight at room temperature, the colour changed from red-brown to light brown. Analysis of the reaction mixture by ^{1}H NMR spectroscopy showed full consumption of the starting material and formation of a new product. ^{1}H NMR spectrum also indicate the formation of circa 10% decomposition product of **1**-Yb overnight. After the reaction, the solvent was removed and the crude product was washed with cold pentane (2 x 200 µL). This led to the formation of essentially pure (BDI*)Mg-[CH(Ph)CH(Ph)]-Yb(Am*) (**5**-Yb) (37.0 mg, 0.026 mmol, 82%) as a light brown powder. Crystals suitable for single crystal X-ray diffraction were grown overnight from the concentrated pentane solution at -35 °C.

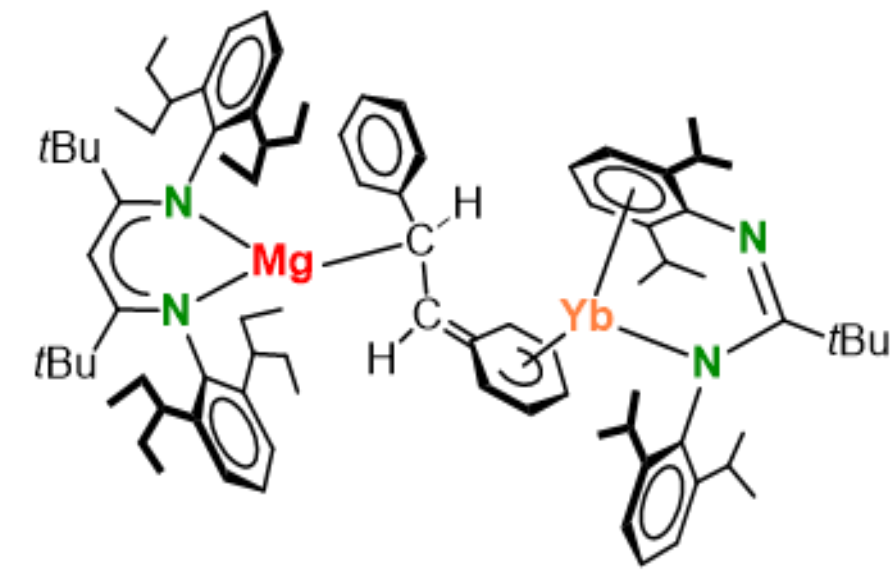

^{1}H NMR (Methylcyclohexane-*d₁₄*, 600 MHz, 298 K): 7.00-6.94 (m, 6H, C**H**$_{arom}$), 6.77–6.73 (m, 4H, C**H**$_{arom}$), 6.45–6.42 (m, 2H, C**H**$_{arom}$), 5.92–5.47 (br, s, 4H, C**H**$_{arom}$-stilbene), 5.35 (s, 1H, C**H**-backbone), 5.15-4.20 (br s, 6H, C**H**$_{arom}$- stilbene), 3.17-3.14 (m, 2H C**H**-*i*Pr), 3.00-2.96 (m, 1H, C**H**-*i*Pr) 2.84-2.77 (m, 5H, C**H** *i*Pen, C**H**-*i*Pr), 1.90-1.84 (m, 3H, C**H**$_2$ *i*Pen), 1.58-1.52 (m, 3H, C**H**$_2$ *i*Pen), 1.44 (br, s, 2H, stilbene), 1.35-1.25 (m, 10H, C**H**$_2$ *i*Pen), 1.20-1.17 (m, 19H, C**H**$_3$ *i*Pr, C**H**$_3$ *t*Bu-Am*), 1.10 (s, 18H, C**H**$_3$ *t*Bu BDI*), 0.98-0.94 (m, 12H, C**H**$_3$ *i*Pen), 0.90-0.88 (m, 12H, C**H**$_3$ *i*Pen), 0.0.84 (br, s, 3H, C**H**$_3$ *i*Pr), 0.82 (br, s, 3H, C**H**$_3$ *i*Pr), 0.71-0.69 (m, 3H, C**H**$_3$ *i*Pr), 0.63-0.61 (m, 3H, C**H**$_3$ *i*Pr) ppm.

^{13}C{^{1}H} NMR (Methylcyclohexane-*d₁₄*, 600 MHz, 298 K): δ = 177.2 (C, **C**N-backbone-BDI*), 170.5 (C, **C**N-backbone-Am*) 159.2 (C, **C**$_{arom}$) 147.4 (C, **C**$_{arom}$), 147.2 (C, **C**$_{arom}$), 143.3 (C, **C**$_{arom}$), 142.8 (C, **C**$_{arom}$), 142.2 (C, **C**$_{arom}$), 140.3 (C, **C**$_{arom}$), 139.1 (C, **C**$_{arom}$), 126.1 (C, **C**$_{arom}$), 126.0 (C, **C**$_{arom}$), 123.7 (C, **C**$_{arom}$), 123.5 (C, **C**$_{arom}$), 122.6 (C, **C**$_{arom}$), 122.5 (C, **C**$_{arom}$), 120.2 (C, **C**$_{arom}$), 120.0 (C, **C**$_{arom}$), 117.0 (C, **C**$_{arom}$), 97.2 (C, **C**H-backbone), 44.5 (C, **C**Me$_3$, BDI*), 42.4 (C, **C**Me$_3$, Am*) 41.1 (C, **C**H *i*Pen), 33.2 (C, **C**H$_3$ *t*Bu, BDI*), 31.7 (C, **C**H$_3$, *t*Bu Am*), 28.8 (C, **C**H *i*Pr), 28.2 (C, **C**H *i*Pr), 27.4 (C, **C**H$_2$ *i*Pen), 27.1 (C, **C**H$_2$ *i*Pen), 26.2 (C, **C**H$_2$ *i*Pen), 24.3 (C, **C**H$_3$ *i*Pr), 23.9 (C, **C**H$_3$ *i*Pr), 21.7 (C, **C**H$_3$ *i*Pr), 20.2 (C, **C**H$_3$ *i*Pr), 14.2 (C, **C**H$_3$ *i*Pr), 11.3 (C, **C**H$_3$ *i*Pen), 11.2 (C, **C**H$_3$ *i*Pen), 10.8 (C, **C**H$_3$ *i*Pen) ppm.

Elemental analysis for $C_{86}H_{124}MgYbN_4$ (M = 1555.53 g mol^{-1}): Calc.: C 73.19%, H 8.86%, N 3.97%. Found: C 72.08%, H 8.11%, N 4.53%. Although some measured results deviate by more than 0.4%, they are provided to illustrate the best values obtained.

Synthesis of (BDI*)Mg-[CH(Ph)CH(Ph)]-Ca(Am*) (**5**-Ca). In a J-Young NMR tube, (BDI*)Mg-Ca(Am*) (**1**-Ca) (40.0 mg, 0.036 mmol, 1.0 eq.) and trans-stilbene (6.5 mg, 0.036 mmol, 1.0 eq.) were dissolved in cyclohexane (1 mL). After stirring the reaction mixture overnight at room temperature, the colour changed from red-brown to light brown. Analysis of the reaction mixture by ^{1}H NMR spectroscopy showed full consumption of the starting material and formation of a new product. ^{1}H NMR spectrum also indicate the formation of circa 5% decomposition product of **1**-Ca. After the reaction, the solvent was removed and the crude product was washed with cold pentane (2 x 200 µL). This led to the formation of essentially pure (BDI*)Mg-[CH(Ph)CH(Ph)]-Ca(Am*) (**5**-Ca) (34.7 mg, 0.027 mmol, 85%) as a light brown powder. Crystals suitable for single crystal X-ray diffraction were grown overnight from the concentrated pentane solution at -35 °C.

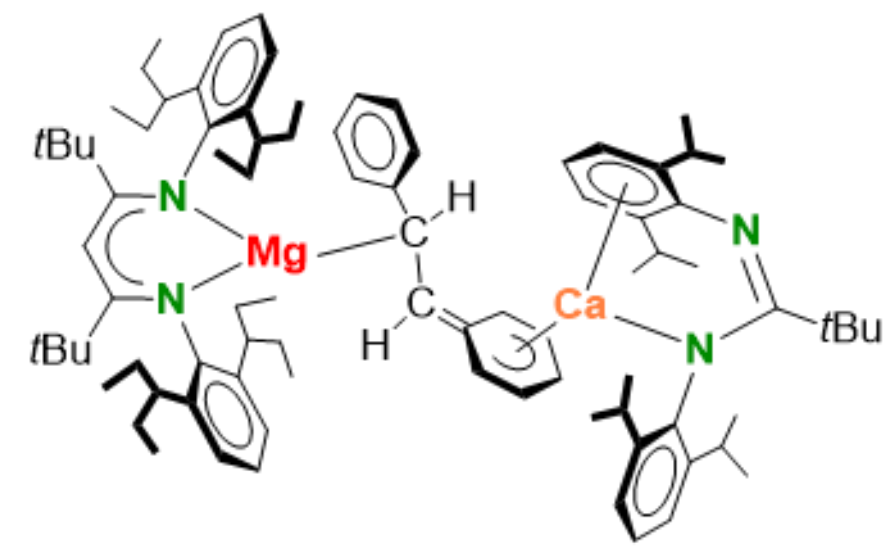

^{1}H NMR (Cyclohexane-d_{12}, 600 MHz, 298 K): 6.98-6.93 (m, 6H, C**H**$_{arom}$), 6.86 (br, s, 1H, C**H**$_{arom}$) 6.77 (br, s, 3H, C**H**$_{arom}$), 6.67–6.54 (m, 2H, C**H**$_{arom}$), 5.64 (br, s, 4H, C**H**$_{arom}$-stilbene), 5.34 (s, 1H, C**H**-backbone), 5.02-4.64 (m, 6H, C**H**$_{arom}$- stilbene), 3.18 (m, 1H C**H**-*i*Pr), 3.10-3.07 (m, 2H, C**H**-*i*Pr), 2.90 (m, 1H, C**H**-*i*Pr), 2.83-2.77 (m, 4H, C**H** *i*Pen), 1.90-1.84 (m, 4H, C**H**$_2$ *i*Pen), 1.64-1.52 (m, 12H, C**H**$_2$ *i*Pen), 1.18 (s, 9H, C**H**$_3$, *t*Bu Am*), 1.15 (d, $^3J_{HH}$ = 6 Hz, 6H, C**H**$_3$ *i*Pen), 1.10 (s, 18H, C**H**$_3$ *t*Bu BDI*), 1.00-0.85 (m, 35H, C**H**$_3$ *i*Pr, C**H**$_3$ *i*Pen), 0.77-0.69 (m, 6H, C**H**$_3$ *i*Pr) ppm.

^{13}C{^{1}H} NMR (Cyclohexane-d_{12}, 600 MHz, 298 K): δ = 177.5 (C, **C**N-backbone-BDI*), 169.8 (C, **C**N-backbone-Am*), 147.6 (C, **C**$_{arom}$), 139.9 (C, **C**$_{arom}$), 139.6 (C, **C**$_{arom}$), 130.36 (C, **C**$_{arom}$), 126.4 (C, **C**$_{arom}$), 126.3 (C, **C**$_{arom}$), 123.9 (C, **C**$_{arom}$), 121.4 (C, **C**$_{arom}$), 96.5 (C, **C**H-backbone), 44.7 (C, **C**Me$_3$, BDI*), 42.6 (C, **C**Me$_3$, Am*) 41.5 (C, **C**H *i*Pen), 33.4 (C, **C**H$_3$ *t*Bu, BDI*), 31.9 (C, **C**H$_3$, *t*Bu Am*), 29.2 (C, **C**H *i*Pr), 27.2 (C, **C**H$_2$ *i*Pen), 24.0 (C, **C**H$_2$ *i*Pen), 11.4 (C, **C**H$_3$ *i*Pen), 11.2 (C, **C**H$_3$ *i*Pen) ppm.

Elemental analysis for $C_{86}H_{124}MgCaN_4$ (M = 1278.35 g mol^{-1}): Calc.: C 80.80%, H 9.78%, N 4.38%. Found: C 79.46%, H 8.77%, N 4.04%. Although some measured results deviate by more than 0.4%, they are provided to illustrate the best values obtained.

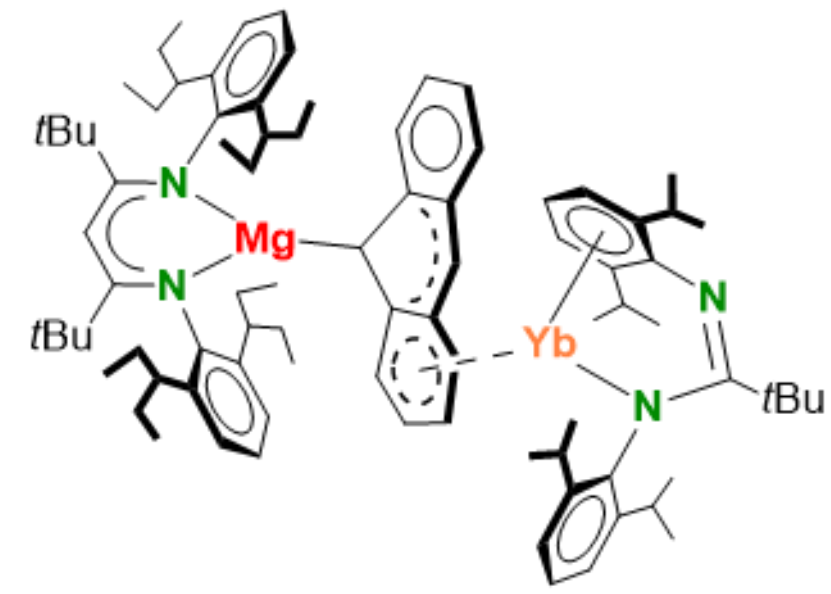

Synthesis of (BDI*)Mg-[anthracene]-Yb(Am*) (**6**-Yb). In a J-Young NMR tube (BDI*)Mg-Yb(Am*) (**1**-Yb) (50.0 mg, 0.041 mmol, 1.0 eq.) and anthracene (7.2 mg, 0.041 mmol, 1.0 eq.) were dissolved in cyclohexane (1 mL). After stirring the reaction mixture overnight at room temperature, the colour changed from red-brown to light brown. Analysis of the reaction mixture by ^{1}H NMR spectroscopy showed quantitative and clean conversion. However, it also showed the formation of circa 10% decomposition product of **1**-Yb. After the reaction, the solvent was removed and the crude product was washed with cold pentane (2 x 200 µL). This led to the isolation of essentially pure (BDI*)Mg-[anthracene]-Yb(Am*) (**6**-Yb) (45.1 mg, 0.032 mmol, 78%) as a brown powder. Crystals suitable for single crystal X-ray diffraction were grown overnight from a concentrated pentane solution at -35 °C.

^{1}H NMR (Cyclohexane-d_{12}, 600 MHz, 298 K): δ = 6.87-6.82 (m, 7H, C**H**$_{arom}$), 6.78 (br, s, 3H, C**H**$_{arom}$), 6.63–6.62 (m, 2H, C**H**$_{arom}$), 5.33 (s, 1H, C**H**-backbone), 5.09 (br s, 4H, C**H**$_{arom}$-anthracene), 4.42 (br, s, 4H, C**H**$_{arom}$-anthracene), 3.13 (s, 2H, C**H**-anthracene), 3.11-3.09 (m, 2H, C**H**-*i*Pr), 2.90-2.83 (m, 6H, C**H**-*i*Pr, C**H** *i*Pen), 1.89-1.84 (m, 4H, C**H**$_2$ *i*Pen), 1.64-1.60 (m, 8H, C**H**$_2$ *i*Pen), 1.57-1.51 (m, 4H, C**H**$_2$ *i*Pen), 1.20-1.19 (m, 6H, C**H**$_3$ *i*Pr), 1.19 (s, 9H, C**H**$_3$ *t*Bu-Am*), 1.13-1.12 (m, 6H, C**H**$_3$ *i*Pr), 1.12 (s, 18H, C**H**$_3$ *t*Bu-BDI*), 1.02 (t, $^{3}J_{HH}$ = 6 Hz, 12H, C**H**$_3$ *i*Pen), 0.91-0.88 (m, 24H, C**H**$_3$ *i*Pen, C**H**$_3$ *i*Pr) ppm.

^{13}C{^{1}H} NMR (Cyclohexane-d_{12}, 600 MHz, 298 K): δ = 177.2 (C, **C**N-backbone-BDI*), 171.4 (C, **C**N-backbone-Am*) 158.7 (C, **C**$_{arom}$) 147.7 (C, **C**$_{arom}$), 147.2 (C, **C**$_{arom}$), 143.6 (C, **C**$_{arom}$), 141.7 (C, **C**$_{arom}$), 139.5 (C, **C**$_{arom}$), 136.6 (C, **C**$_{arom}$), 126.1 (C, **C**$_{arom}$), 124.2 (C, **C**$_{arom}$), 123.4 (C, **C**$_{arom}$), 123.3 (C, **C**$_{arom}$), 122.8 (C, **C**$_{arom}$), 118.6 (C, **C**$_{arom}$), 114.3 (C, **C**$_{arom}$), 97.5 (C, **C**H-backbone), 70.2 (**C**H-anthracene) 44.6 (C, **C**Me$_3$ *t*Bu, BDI*), 42.7 (C, **C**Me$_3$ *t*Bu, Am*) 41.5 (C, **C**H *i*Pen), 33.5 (C, **C**H$_3$ *t*Bu, BDI*), 31.9 (C, **C**H$_3$, *t*Bu Am*), 29.3 (C, **C**H *i*Pr), 28.8 (C, **C**H *i*Pr), 27.4 (C, **C**H$_2$ *i*Pen), 24.1 (C, **C**H$_2$ *i*Pen), 24.0, 23.6 (C, **C**H$_3$ *i*Pr), 21.5 (C, **C**H$_3$ *i*Pr), 12.1 (C, **C**H$_3$ *i*Pen), 11.1 (C, **C**H$_3$ *i*Pen) ppm.

Elemental analysis for $C_{86}H_{122}MgYbN_4$ (M = 1409.30 g mol^{-1}): Calc.: C 73.29%, H 8.73%, N 3.98%. Found: C 72.66%, H 8.05%, N 3.15%. Although some measured results deviate by more than 0.4%, they are provided to illustrate the best values obtained.

Synthesis of (BDI*)Mg-[anthracene]-Yb(Am*) (**6**-Ca). In a J-Young NMR tube (BDI*)Mg-Ca(Am*) (**1**-Ca) (50.0 mg, 0.046 mmol, 1.0 eq.) and anthracene (8.1 mg, 0.046 mmol, 1.0 eq.) were dissolved in cyclohexane (1 mL). After stirring the reaction mixture overnight at room temperature, the colour changed from red-brown to light brown. Analysis of the reaction mixture by ^{1}H NMR spectroscopy showed quantitative and clean conversion. However, it also showed the formation of circa 8% decomposition product of **1**-Ca. After the reaction, the solvent was removed and the crude product was washed with cold pentane (2 x 200 µL). This led to the isolation of essentially pure (BDI*)Mg-[anthracene]-Yb(Am*) (**5**-Ca) (42.8 mg, 0.034 mmol, 73%) as a brown powder. Crystals suitable for single crystal X-ray diffraction were grown overnight from a concentrated pentane solution at -35 °C.

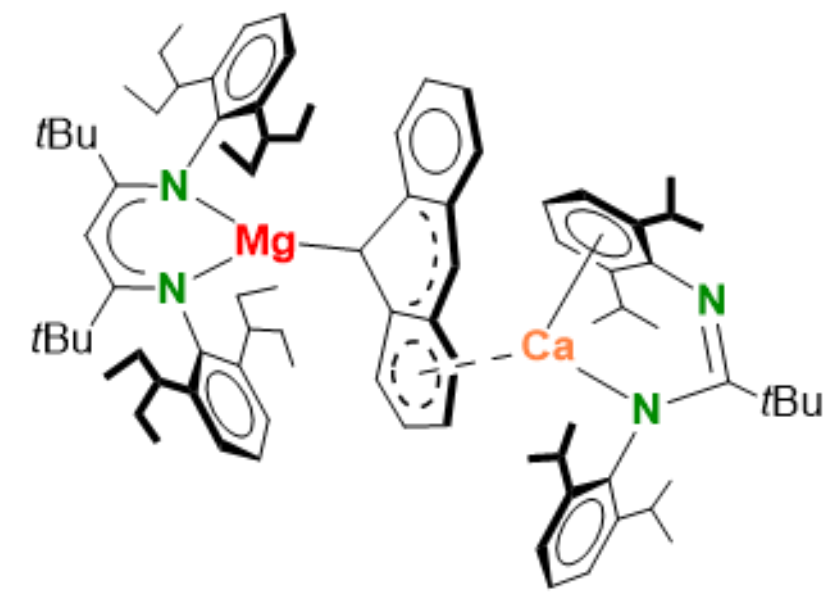

^{1}H NMR (Methylcyclohexane-*d$_{14}$*, 600 MHz, 298 K): δ = 6.88-6.85 (m, 10H, C**H**$_{arom}$), 6.64-6.63 (m, 2H, C**H**$_{arom}$), 5.28-5.26 (m, 4H, C**H**-anthracene), 5.24 (s, 1H, C**H**-backbone), 4.83-4.81 (m, 4H, C**H**$_{arom}$-anthracene), 3.31 (s, 2H, C**H**$_{arom}$-anthracene), 3.06-3.02 (m, 2H, C**H**-*i*Pr), 2.94-2.91 (m, 2H, C**H**-*i*Pr), 2.87-2.85 (m, 4H, C**H** *i*Pen), 1.87-1.80 (m, 4H, C**H**$_2$ *i*Pen), 1.34-1.27 (m, 12H, C**H**$_2$ *i*Pen), 1.20 (d, $^{3}J_{HH}$ = 6 Hz, 6H, C**H**$_3$ *i*Pr), 1.17 (s, 9H, C**H**$_3$ *t*Bu-Am*), 1.09 (s, 18H, C**H**$_3$ *t*Bu-BDI*),1.13-1.12 (m, 6H, C**H**$_3$ *i*Pr), 1.04-1.02 (m, 12H, C**H**$_3$ *i*Pen), 1.02-1.01 (m, 6H, C**H**$_3$ *i*Pr), 0.93-0.91 (m, 6H, C**H**$_3$ *i*Pr), 0.88-0.85 (m, 12H, C**H**$_3$ *i*Pen), 0.84-0.82 (m, 6H, C**H**$_3$ *i*Pr) ppm.

^{13}C{^{1}H} NMR (Methylcyclohexane-*d$_{14}$*, 600 MHz, 298 K): δ = 176.2 (C, **C**N-backbone-BDI*), 169.9 (C, **C**N-backbone-Am*), 148.0 (C, **C**$_{arom}$), 147.2 (C, **C**$_{arom}$), 146.8(C, **C**$_{arom}$), 144.3 (C, **C**$_{arom}$), 141.8 (C, **C**$_{arom}$), 140.5(C, **C**$_{arom}$), 139.5 (C, **C**$_{arom}$), 128.4 (C, **C**$_{arom}$), 126.4 (C, **C**$_{arom}$), 125.9 (C, **C**$_{arom}$),125.6 (C, **C**$_{arom}$), 125.3 (C, **C**$_{arom}$), 123.5 (C, **C**$_{arom}$), 123.3 (C, **C**$_{arom}$), 119.0 (C, **C**$_{arom}$), 114.5 (C, **C**$_{arom}$), 97.0 (C, **C**H-backbone), 68.6 (**C**H-anthracene) 44.4 (C, **C**Me$_3$, BDI*), 42.2 (C, **C**Me$_3$, Am*) 41.0 (C, **C**H *i*Pen), 33.5 (C, **C**H$_3$ *t*Bu, BDI*), 31.6 (C, **C**H$_3$, *t*Bu Am*), 29.0 (C, **C**H *i*Pr), 28.3 (C, **C**H *i*Pr), 26.6 (C, **C**H$_2$ *i*Pen), 23.8 (C, **C**H$_2$ *i*Pen), 23.7(C, **C**H$_3$ *i*Pr), 21.3(C, **C**H$_3$ *i*Pr), 23.7 (C, **C**H$_3$ *i*Pr), 21.3 (C, **C**H$_3$ *i*Pr), 12.1 (C, **C**H$_3$ *i*Pen), 10.7 (C, **C**H$_3$ *i*Pen) ppm.

Elemental analysis for $C_{86}H_{122}MgCaN_4$ (M = 1276.33 g mol^{-1}): Calc.: C 80.93%, H 9.64%, N 4.39%. Found: C 79.93%, H 9.43%, N 4.68%. Although some measured results deviate by more than 0.4%, they are provided to illustrate the best values obtained.

Synthesis of (BDI*)Mg-(µ-NH_2)$_2$-Ca(Am*) (**7**-Ca). (BDI*)Mg-Ca(Am*) (**1**-Ca) (50.0 mg, 0.046 mmol, 1.0 eq.) was dissolved in methylcyclohexane-*d_{14}* (600 µL) and the solution was cooled to −80 °C and degassed by freeze–pump–thawing (2 times). After evacuation of the head space, NH_3(g) was added at 1 bar pressure. To achieve a slow diffusion-controlled reaction, the NMR tube was kept at −80 °C without stirring for 5 minutes, until the whole solution changed from dark red to light yellow. Then the reaction mixture was stirred for 5 minutes at room temperature. The ^{1}H NMR of the reaction mixture showed full consumption of (BDI*)Mg-Ca(Am*) (**1**-Ca) and formation one major product along with some minor product; circa 10-15 % based on BDI* backbone signals. Th reaction mixture was then filtered and the solvent was removed in *vacuo* to afford a light yellow powder. The crude sample was purified by crystallization in pentane. Colourless crystals were grown in pentane at -35 °C (22.1 mg, 0.019 mmol, 43%) in two days.

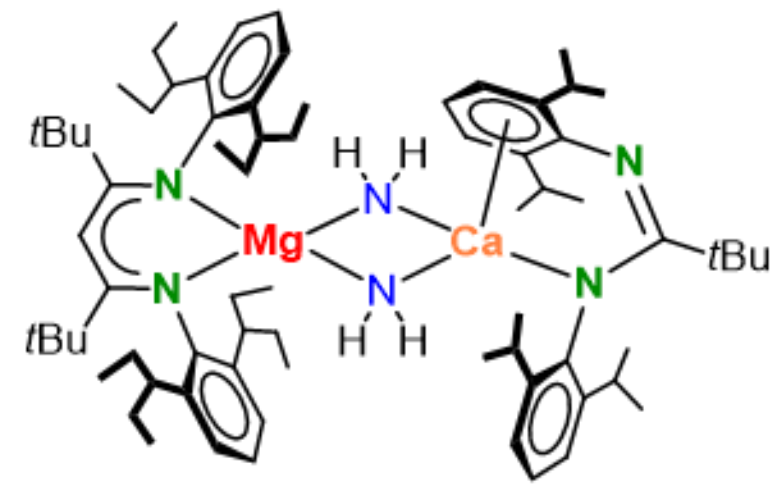

^{1}H NMR (Cyclohexane-*d_{12}*, 600 MHz, 298 K): δ = 6.97-6.92 (m, 6H, C**H**$_{arom}$), 6.88-6.85 (m, 5H, C**H**$_{arom}$), 5.26 (br, s, 1H, C**H**$_{arom}$), 5.02 (s, 1H, C**H**-backbone), 3.15-3.10 (m, 2H, C**H**-*i*Pr), 3.07-2.98 (m, 6H, C**H**-*i*Pr, C**H** *i*Pen), 1.82-1.78 (m, 4H, C**H**$_2$ *i*Pen), 1.74-1.72 (m, 4H, C**H**$_2$ *i*Pen), 1.66-1.62 (m, 4H, C**H**$_2$ *i*Pen), 1.33-1.26 (m, 4H, C**H**$_2$ *i*Pen), 1.16 (m, 12H, C**H**$_3$ *i*Pr), 1.10 (s, 9H, C**H**$_3$ *t*Bu-Am*), 1.08 (s, 18H, C**H**$_3$ *t*Bu-BDI*), 0.90-0.87 (m, 24H, C**H**$_3$ *i*Pen), 0.86-0.84 (m, 6H, C**H**$_3$ *i*Pr), 0.74-0.72 (m, 6H, C**H**$_3$ *i*Pr, -1.90 (s, 4H, NH_2) ppm.

^{13}C{^{1}H} NMR (Cyclohexane-*d_{12}*, 600 MHz, 298 K): δ = 176.0 (C, **C**N-backbone-BDI*), 169.6 (C, **C**N-backbone-Am*), 148.9 (C, **C**$_{arom}$), 147.3 (C, **C**$_{arom}$), 142.5(C, **C**$_{arom}$), 144.4 (C, **C**$_{arom}$), 126.8 (C, **C**$_{arom}$), 125.5(C, **C**$_{arom}$), 123.9 (C, **C**$_{arom}$), 119.2 (C, **C**$_{arom}$), 95.7 (C, **C**H-backbone), 39.7 (C, **C**Me$_3$, BDI*), 35.0 (C, **C**H *i*Pen), 33.9 (C, **C**Me$_3$, Am*) 31.9 (C, **C**H$_3$ *t*Bu, BDI*), 29.1 (C, **C**H$_3$, *t*Bu Am*), 29.1 (C, **C**H *i*Pr), 28.3 (C, **C**H *i*Pr), 25.7 (C, **C**H$_2$ *i*Pen), 24.1 (C, **C**H$_2$ *i*Pen), 23.7 (C, **C**H$_3$ *i*Pr), 21.7 (C, **C**H$_3$ *i*Pr), 14.4 (C, **C**H$_3$ *i*Pr), 10.9 (C, **C**H$_3$ *i*Pen), 10.2 (C, **C**H$_3$ *i*Pen) ppm.

Elemental analysis for $C_{72}H_{116}MgCaN_6$ (M = 1130.14 g mol^{-1}): Calc.: C 76.52%, H 10.35%, N 7.44%. Found: C 75.19%, H 9.85%, N 6.32%. Although some measured results deviate by more than 0.4%, they are provided to illustrate the best values obtained.

Reaction of (BDI*)Mg-Yb(Am*) (**1-**Yb) **with NH_3**

(BDI*)Mg-Yb(Am*) (**1**-Yb) (28.0 mg, 0.023 mmol, 1.0 eq.) was dissolved in methylcyclohexane-d_{14} (600 µL) and the solution was cooled to −80 °C and degassed by freeze–pump–thawing (2 times). After evacuation of the head space, NH_3(g) was added at 1 bar pressure. To achieve a slow diffusion-controlled reaction, the NMR tube was kept at −80 °C without stirring for 5 minutes, until the whole solution changed from dark red to light brown. At this moment, a ^{1}H NMR indicated the full consumption of starting material and formation of a new compound (Fig. S53). Then, the reaction mixture was stirred for 5 minutes at room temperature. This led to a change of colour to light yellow along with the formation of a light yellow precipitate. The ^{1}H NMR of the reaction mixture showed only one very broad signal indicating the formation of paramagnetic Yb(III) species(Fig S54). The reaction mixture was filtered and a ^{1}H NMR of filtrate was remeasured which showed well resolved signals corresponding to the BDI* and Am* ligands (Fig. S55). The residue was collected, washed with pentane and dried under high vacuum. ^{1}H NMR of the light yellow residue in THF-d_8 was NMR silent and only showed few very broad signals around 1 ppm (Fig. S56, we presume the formation of $Yb(NH_2)_3$). All attempts to crystalize the compounds from the filtrate were unsuccessful.

Reaction of (BDI*)Mg-M(Am*) (M = Ca, 1-Ca and M = Yb, 1-Yb) with H_2, CO and CO_2

(BDI*)Mg-M(Am*) (**1-**M) (20.0 mg) was dissolved in methylcyclohexane-d_{14} (550 µL) and the solution was cooled to −80 °C and degassed by freeze–pump–thawing (2 times). After evacuation of the head space, the reactant gas H_2, CO and CO_2 (1.0 bar) was added. To achieve a slow diffusion-controlled reaction, the NMR tube was kept at −80 °C without stirring for 5 minutes, until the whole solution changed from dark red to yellow. Then the reaction mixture was stirred for 5 minutes at room temperature. The ^{1}H NMR of the reaction mixture showed in all cases full consumption of **1-**Ca and, based on numerous BDI* backbone CH-signals, the formation of multiple products. We were not able to isolate any of the reaction products from these mixtures. In case of **1**-Yb, we obtained in all cases very broad ^{1}H NMR resonances indicating the formation of paramagnetic Yb(III) species.

Reaction of (BDI*)Mg-Yb(Am*) (**1**-Yb) **with I_2**

(BDI*)Mg-Yb(Am*) (**1**-Yb) (20.0 mg, 0.016 mmol, 1.0 eq.) and I_2 (4.1 mg, 0.016 mmol, 1.0 eq.) were dissolved in cyclohexane-d_{12} (550 µL). The reaction mixture was stirred for 10 minutes and the colour changed from red-brown to light yellow. ^{1}H NMR spectrum indicated the formation of paramagnetic compounds. We were not able to isolate any of the reaction products from these mixtures.

Decomposition of (BDI*)Mg-Yb(Am*) (**1**-Yb)

In a J-Young NMR tube, (BDI*)Mg-Yb(Am*) (**1**-Yb) (20.0 mg, 0.016 mmol, 1.0 eq.) was dissolved in cyclohexane-d_{12} (550 µL). The reaction mixture was stirred at room temperature for 4 days and the colour changed from red-brown to light yellow. Complex decomposition was monitored by ^{1}H NMR spectroscopy. After 4 days, the full consumption of starting material and formation of at least 3 new compounds was observed (Fig. S37). Numerous attempts to crystallize these products failed. However, the major product in the ^{1}H NMR spectrum of decomposed product resembles the proposed decomposition product of **1**-Ca (Fig. S38). It is most likely that the formally Mg^{0} centre inserted in a benzylic C-H bond of the BDI* ligand. The presence of an Yb-H hydride signal is proven by ^{1}H NMR (Fig. S37).

Decomposition of (BDI*)Mg-M(Am*) (M = Ca (**1**-Ca), Yb(**1**-Yb)) **in benzene**: In separate J-Young NMR tubes, (BDI*)Mg-Ca(Am*) (**1**-Ca) (15.0 mg) and (BDI*)Mg-Yb(Am*) (**1**-Yb) (15.0 mg) were dissolved in benzene (550 µL). The reaction mixtures were stirred at room temperature for 10 minutes and the colour of reaction mixture in both cases changed from red-brown to yellow. After the reaction, solvent was removed in *vacuo* to afford a light yellow powder. ^{1}H NMR spectra of crude compounds shows reductive cleavage of C-H bond of benzene and formation of previously reported (BDI*)MgPh[1] and (Am*)MH (M = Ca, Yb) complexes.[4,5] Due to poor solubility of (Am*)CaH and (Am*)YbH in benzene, corresponding signals are of very low intensity due to formation of precipitates (Fig. S43).

Reaction of (BDI*)Mg-Ca(Am*) (**1**-Ca) with N_2O

(BDI*)Mg-Ca(Am*) (20.0 mg) was dissolved in methylcyclohexane-d_{14} (600 µL) and the solution was cooled to −80 °C and degassed by freeze–pump–thawing (2 times). After evacuation of the head space, N_2O (1.0 bar) was added. To achieve a slow diffusion-controlled reaction, the NMR tube was kept at −80 °C without stirring for 5 minutes, until the whole solution changed from dark red to yellow. ^{1}H NMR showed full consumption of starting material and formation of at least 3 new products (Fig. S51). After the reaction, the reaction mixture was filtered and left it in the freezer for crystallization. Colourless needle-shaped crystals in minor quantity were obtained by storing a concentrated pentane solution at −35 °C. The crystal structure showed deprotonation of a terminal methyl group of an iso-pentyl side arm by the in-situ formed oxide dianion (Fig. S64).

Reaction of (BDI*)Mg-Yb(Am*) (1-Yb) with N_2O

(BDI*)Mg-Yb(Am*) (**1**-Yb) (20.0 mg) was dissolved in methylcyclohexane-*d*$_{14}$ (600 µL) and the solution was cooled to −80 °C and degassed by freeze–pump–thawing (2 times). After evacuation of the head space, N_2O (1.0 bar) was added. To achieve a slow diffusion-controlled reaction, the NMR tube was kept at −80 °C without stirring for 5 minutes, until the whole solution changed from dark red to yellow. ^{1}H NMR showed broad signals in the range of +13 to -3 ppm. This indicates the possible oxidation of Yb^{II} to Yb^{III} (Fig. S52). We could not isolate any well-defined compound from this complex reaction mixture.

3. Spectroscopic data

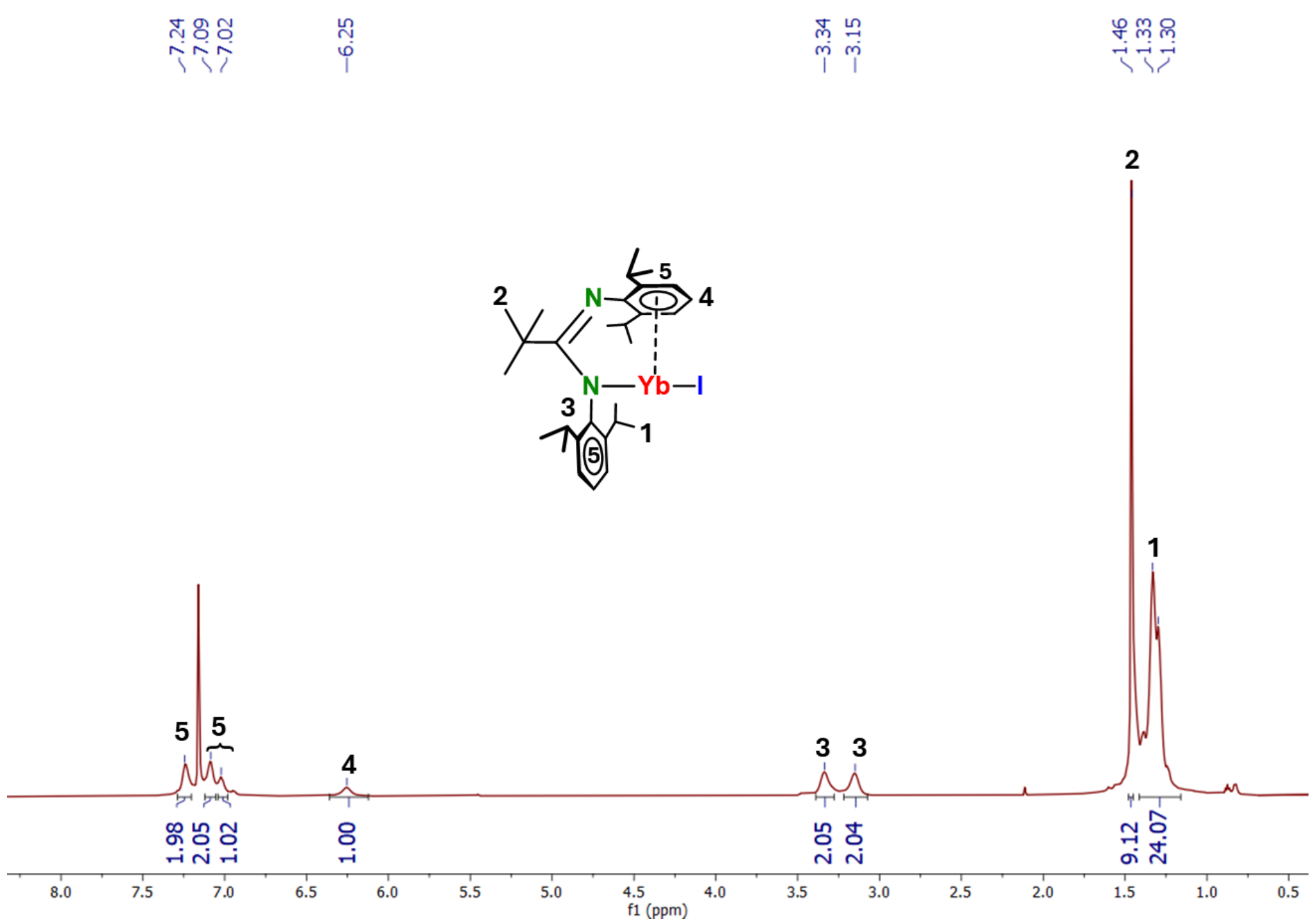

Fig. S1. ^{1}H NMR (600 MHz, 298 K, C_6D_6) spectrum of $[Am^*YbI]_2$ (**2**-Yb).

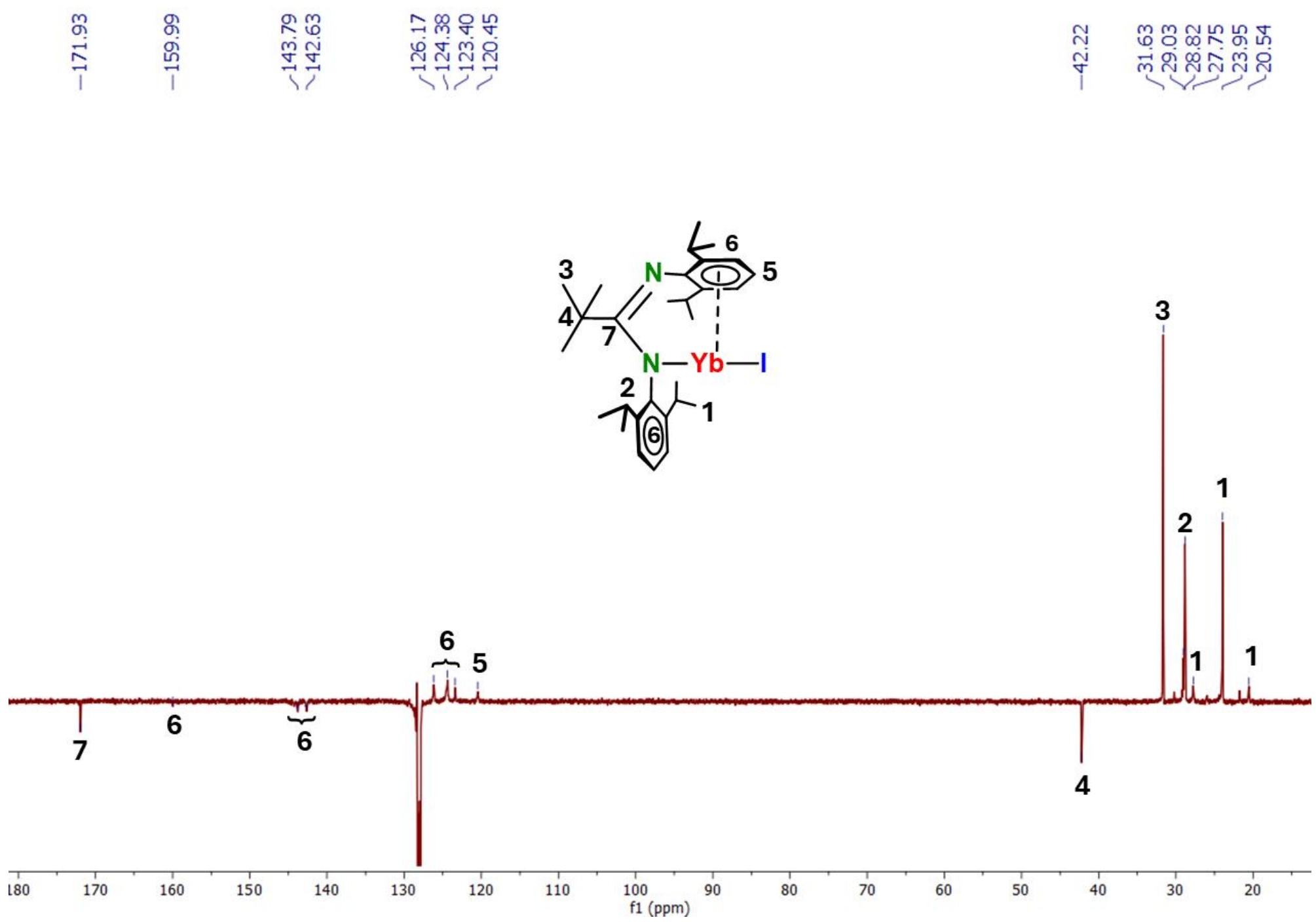

Fig. S2. ^{13}C-APT NMR (151 MHz, 298 K, C_6D_6) spectrum of [Am*YbI]$_2$ (**2**-Yb).

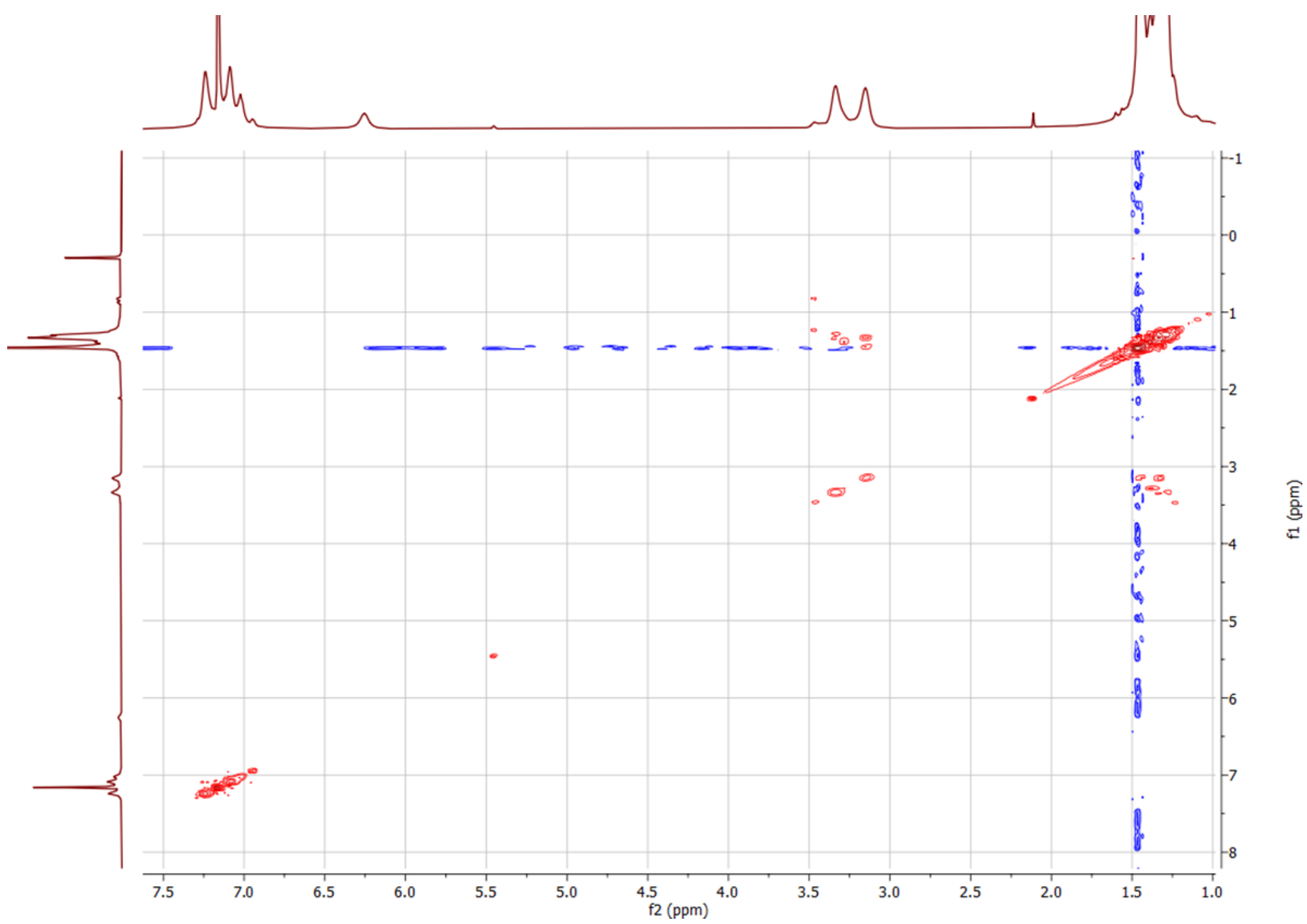

Fig. S3. 2D-COSY NMR (600 MHz, 298 K, C_6D_6) spectrum of [Am*YbI]$_2$ (**2**-Yb).

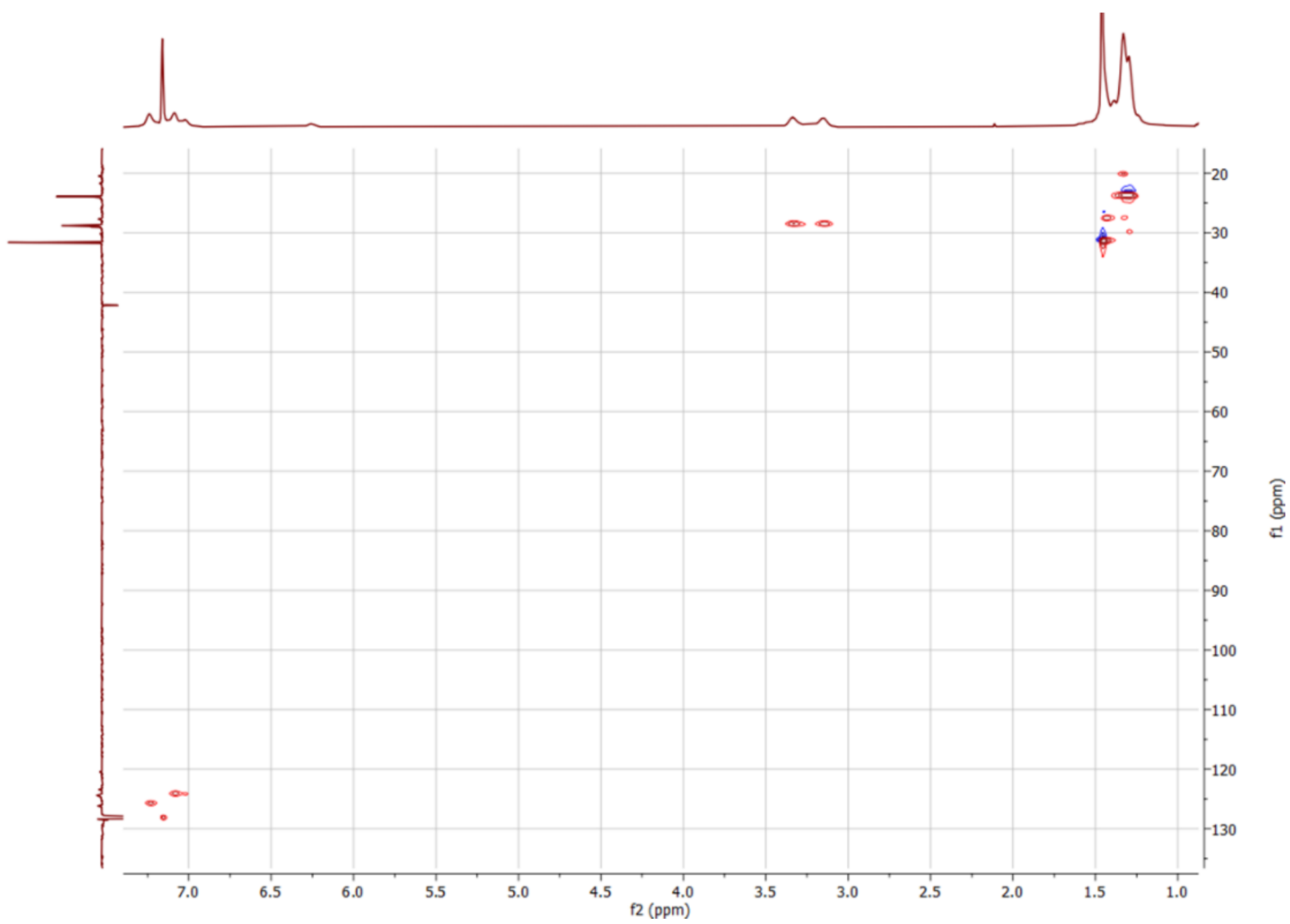

Fig. S4. 2D-HSQC NMR (600/151 MHz, 298 K, C_6D_6) spectrum of $[Am^*YbI]_2$ (**2**-Yb).

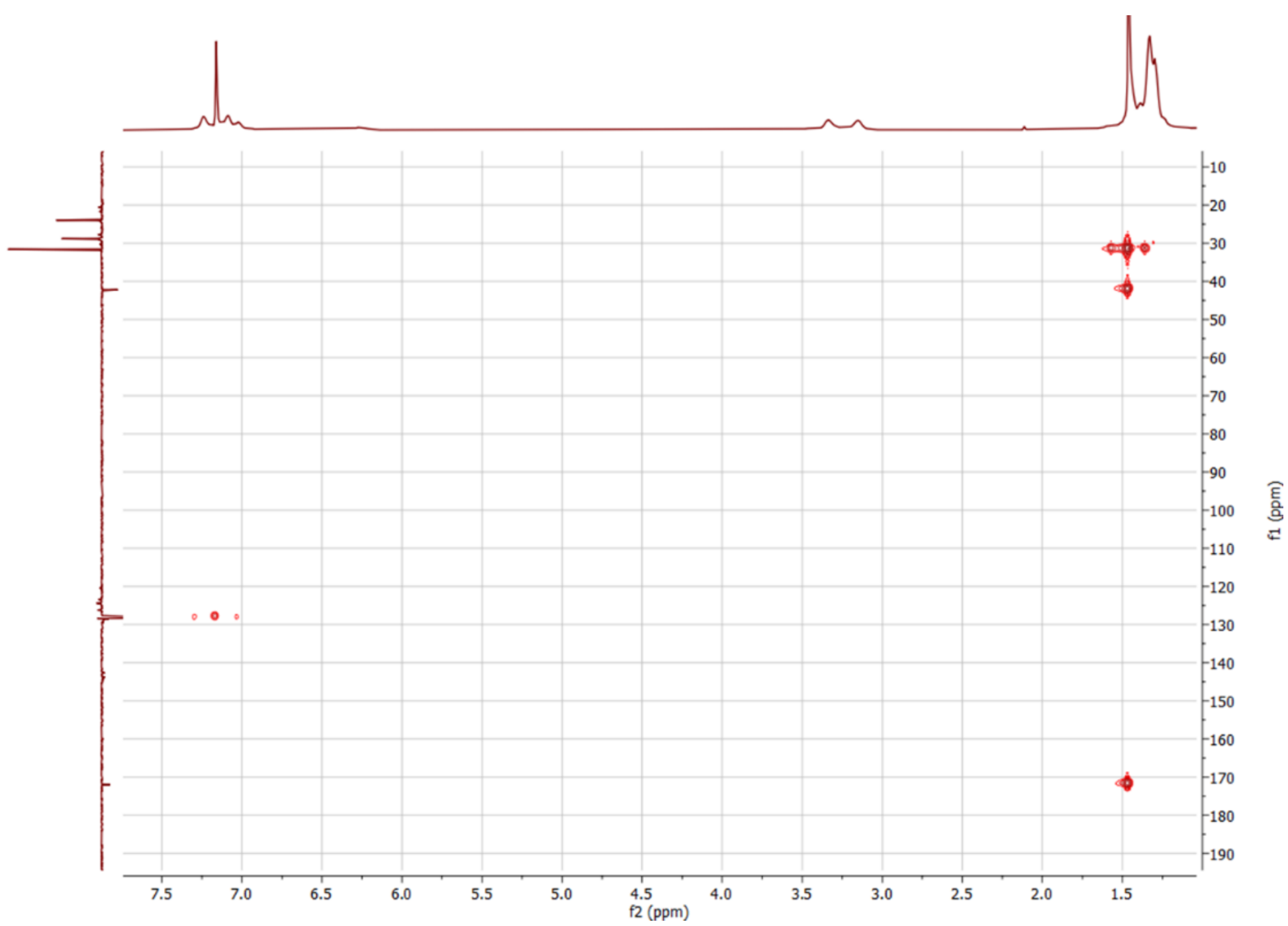

Fig. S5. 2D-HMBC NMR (600/151 MHz, 298 K, C_6D_6) spectrum of $[Am^*YbI]_2$ (**2**-Yb).

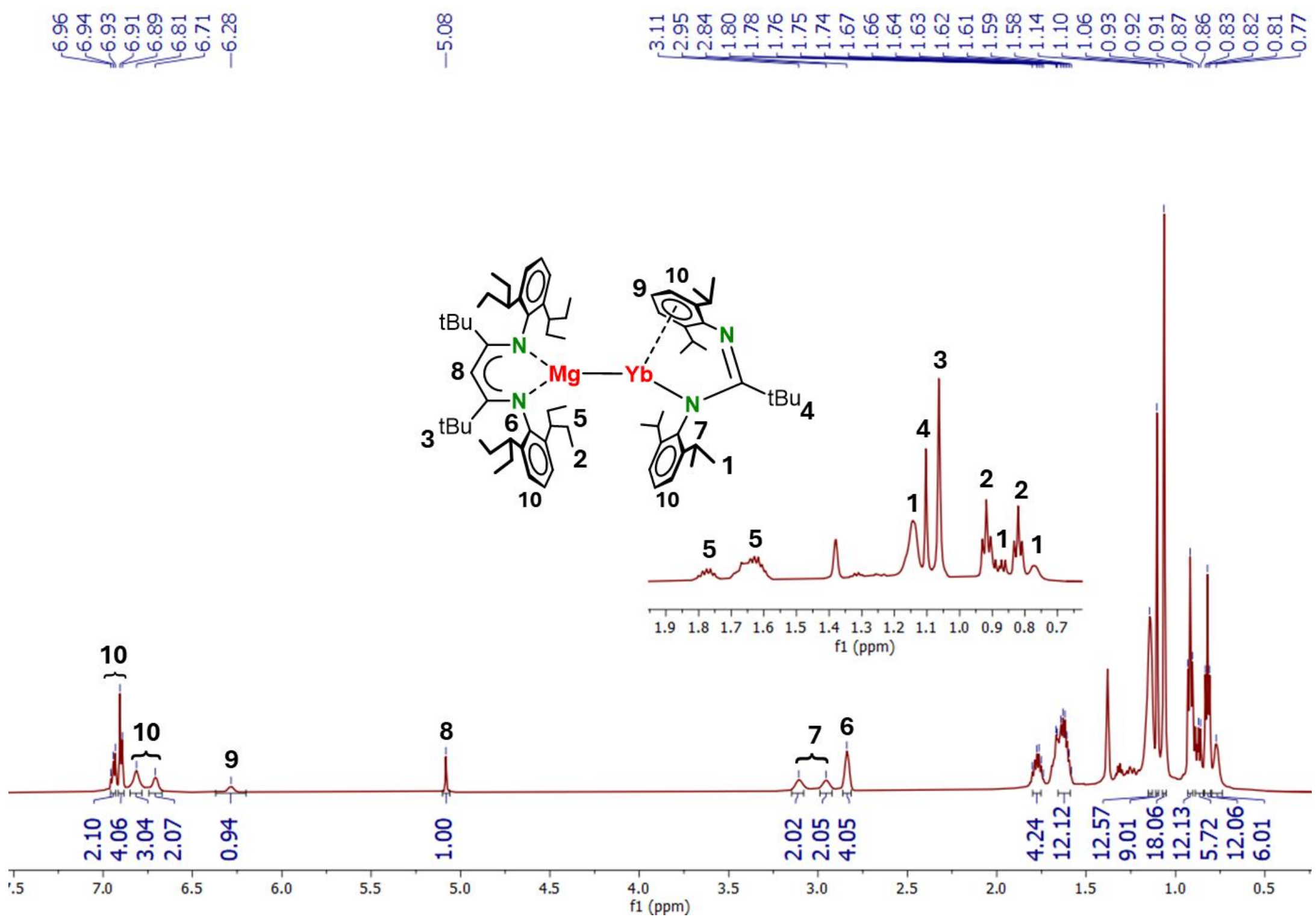

Fig. S6. ^{1}H NMR (600 MHz, 298 K, C_6D_{12}) spectrum of (BDI*)Mg-Yb(Am*) (**1**-Yb).

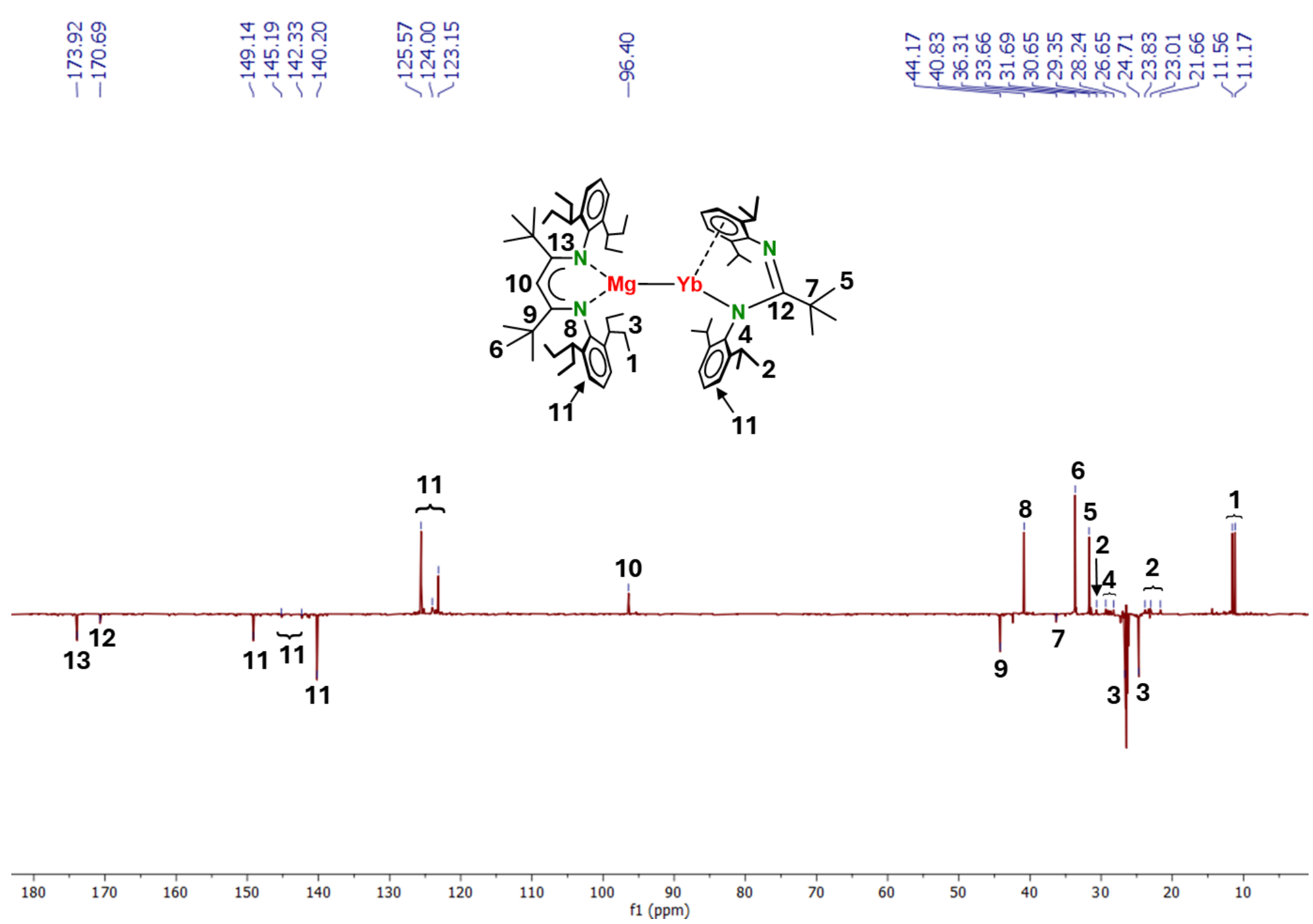

Fig. S7. ^{13}C-APT NMR (151 MHz, 298 K, C_6D_{12}) spectrum of (BDI*)Mg-Yb(Am*) (**1**-Yb).

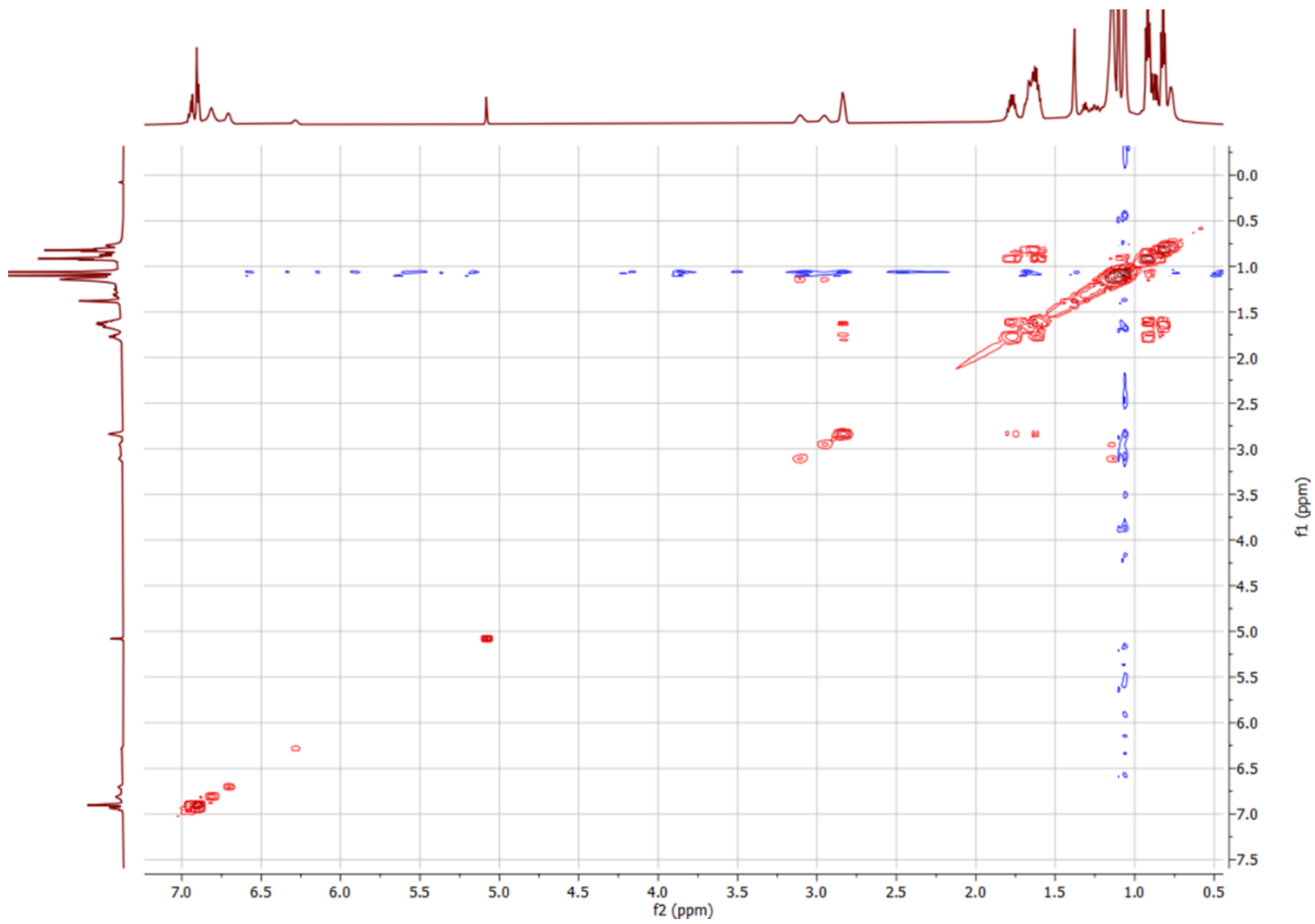

Fig. S8. 2D-COSY NMR (600 MHz, 298 K, C_6D_{12}) spectrum of (BDI*)Mg-Yb(Am*) (**1**-Yb).

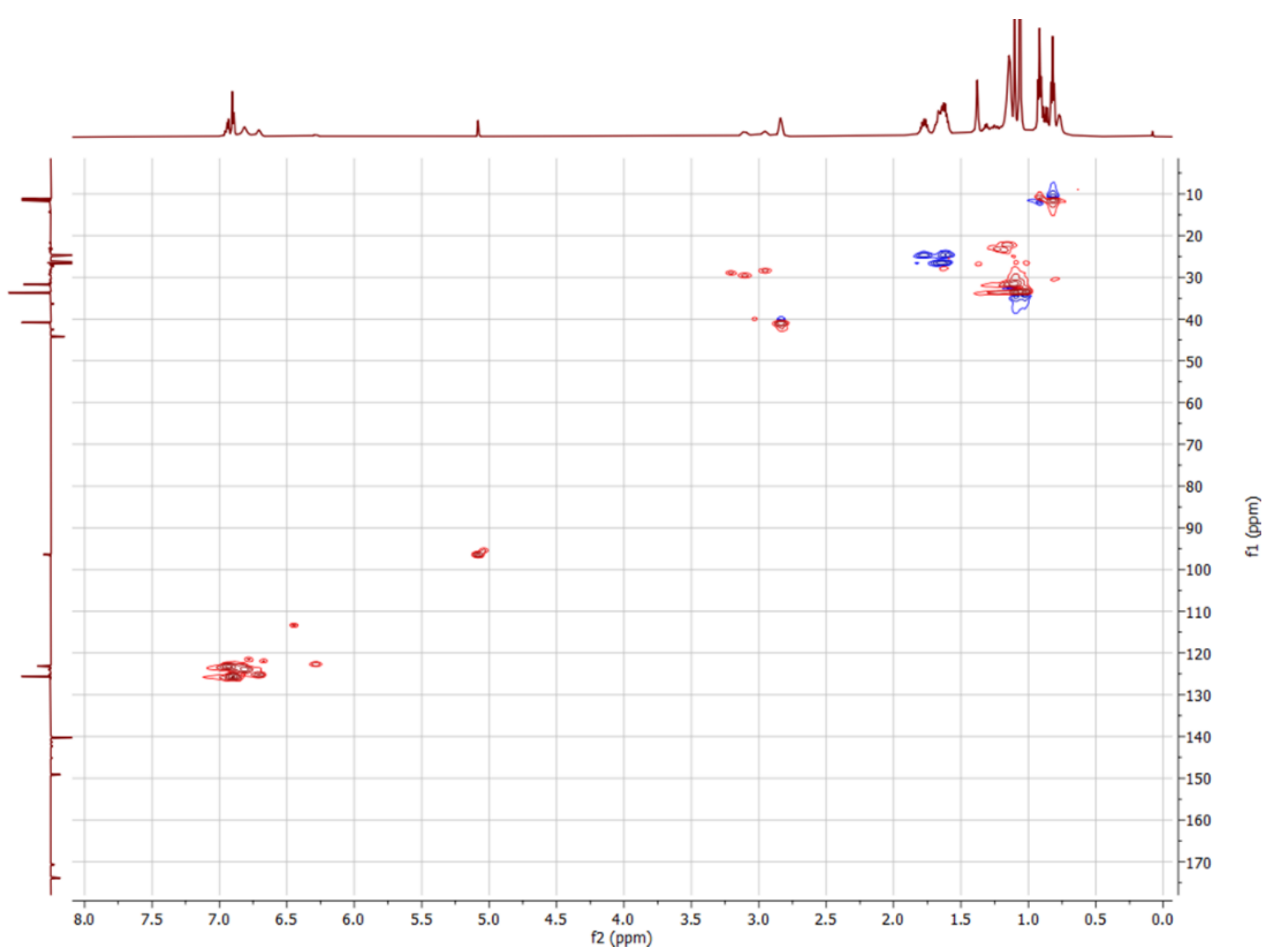

Fig. S9. 2D-HSQC NMR (600/151 MHz, 298 K, C_6D_{12}) spectrum of (BDI*)Mg-Yb(Am*) (**1**-Yb).

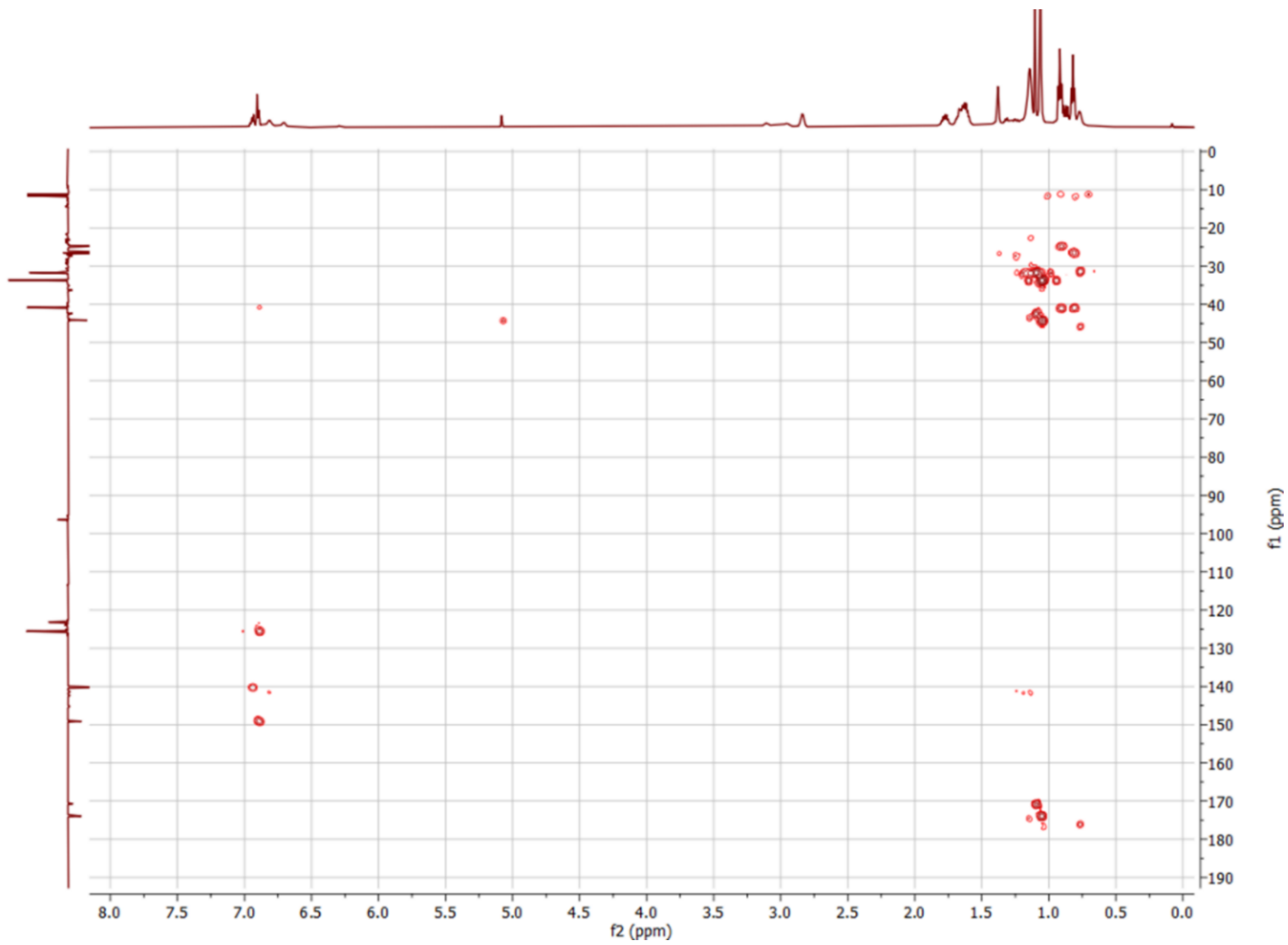

Fig. S10. 2D-HMBC NMR (600/151 MHz, 298 K, C_6D_{12}) spectrum of (BDI*)Mg-Yb(Am*) (**1**-Yb).

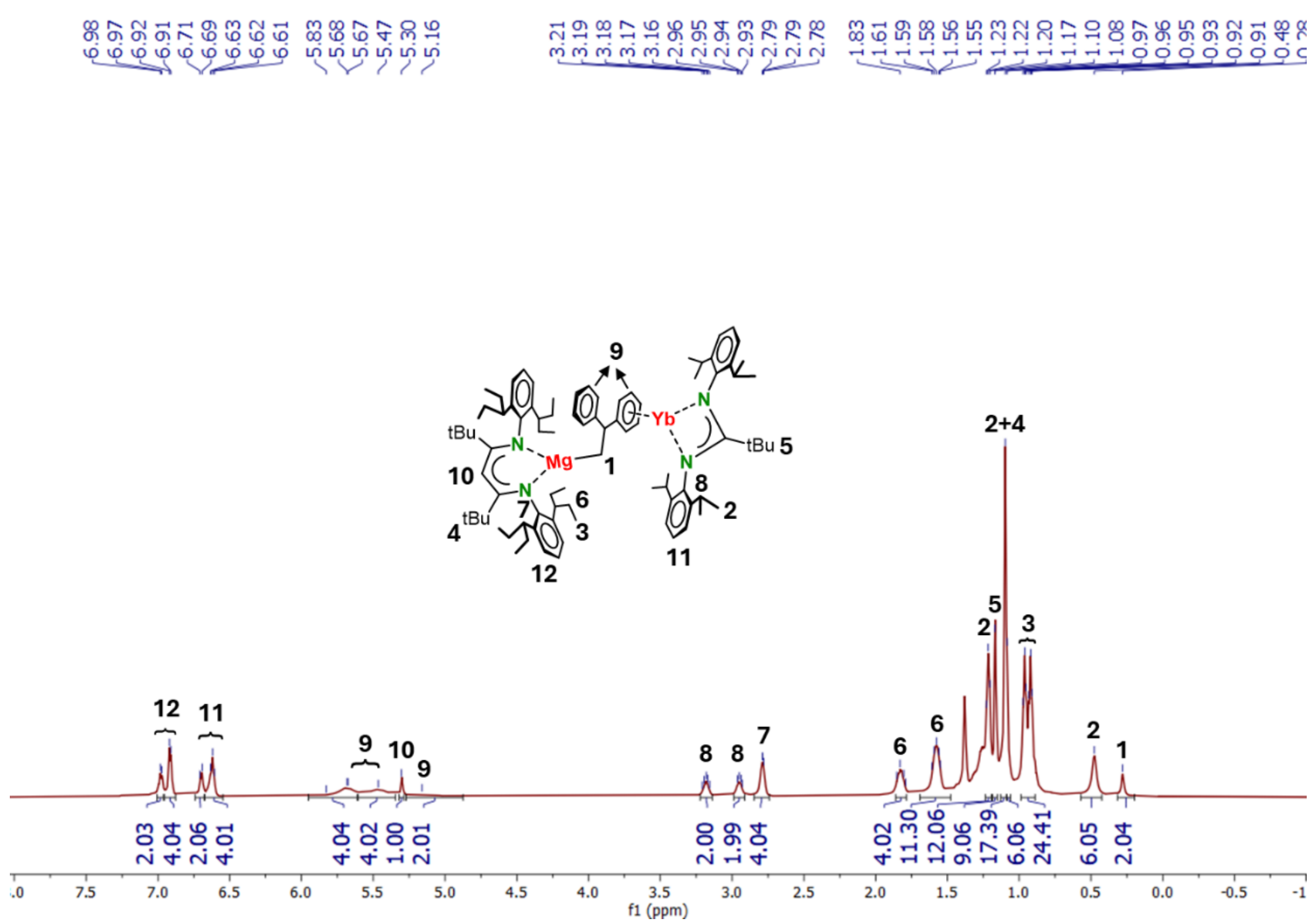

Fig. S11. ^{1}H NMR (600 MHz, 298 K, C_6D_{12}) spectrum of (BDI*)Mg-[H_2CCPh_2]-Yb(Am*) (**4**-Yb).

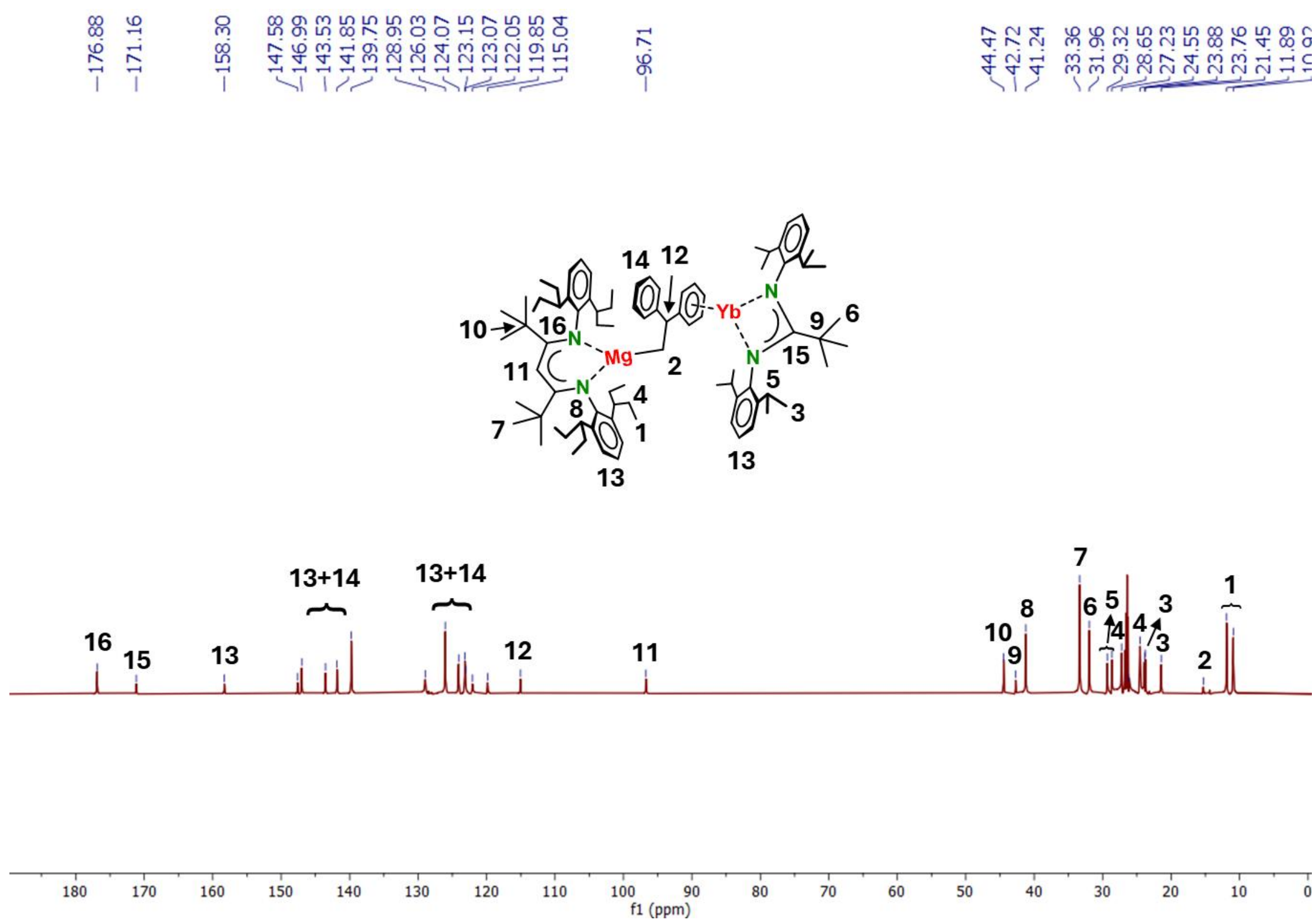

Fig. S12. ^{13}C-APT NMR (151 MHz, 298 K, C_6D_{12}) spectrum of (BDI*)Mg-[H_2CCPh_2]-Yb(Am*) (**4**-Yb).

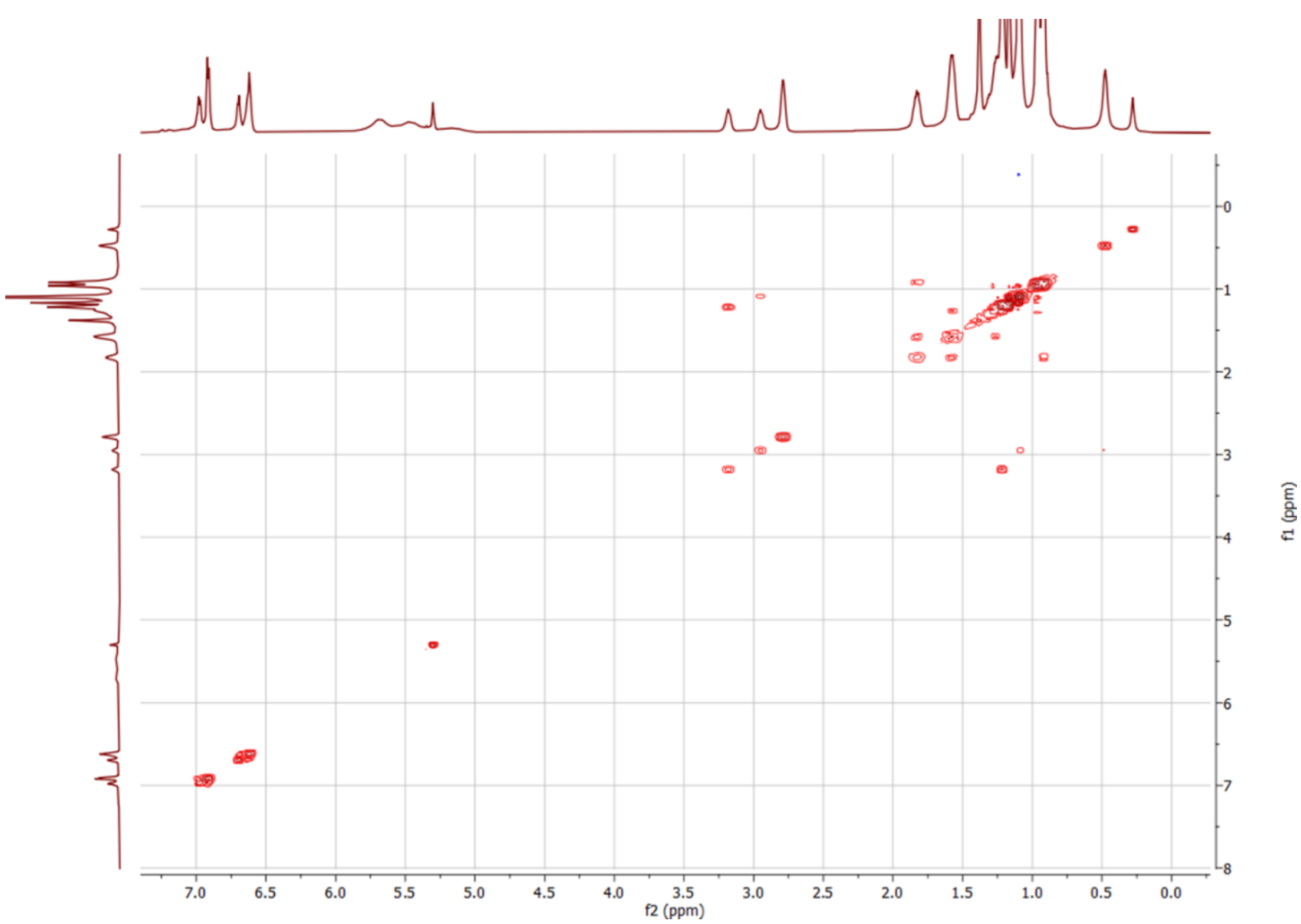

Fig. S13. 2D-COSY NMR (600 MHz, 298 K, C_6D_{12}) spectrum of (BDI*)Mg-[H_2CCPh_2]-Yb(Am*) (**4**-Yb).

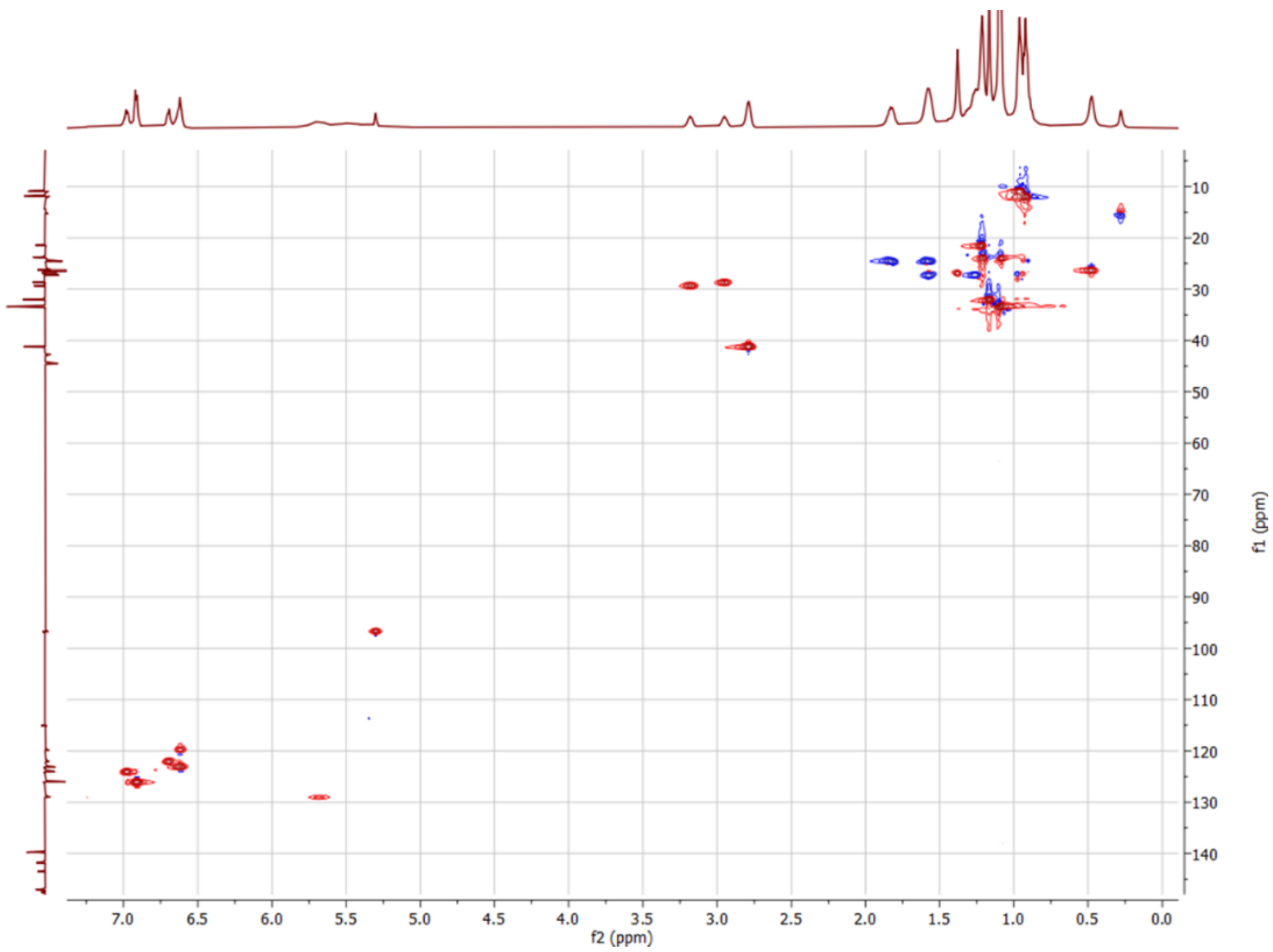

Fig. S14. 2D-HSQC NMR (600/151 MHz, 298 K, C_6D_{12}) spectrum of (BDI*)Mg-[H_2CCPh_2]-Yb(Am*) (**4**-Yb).

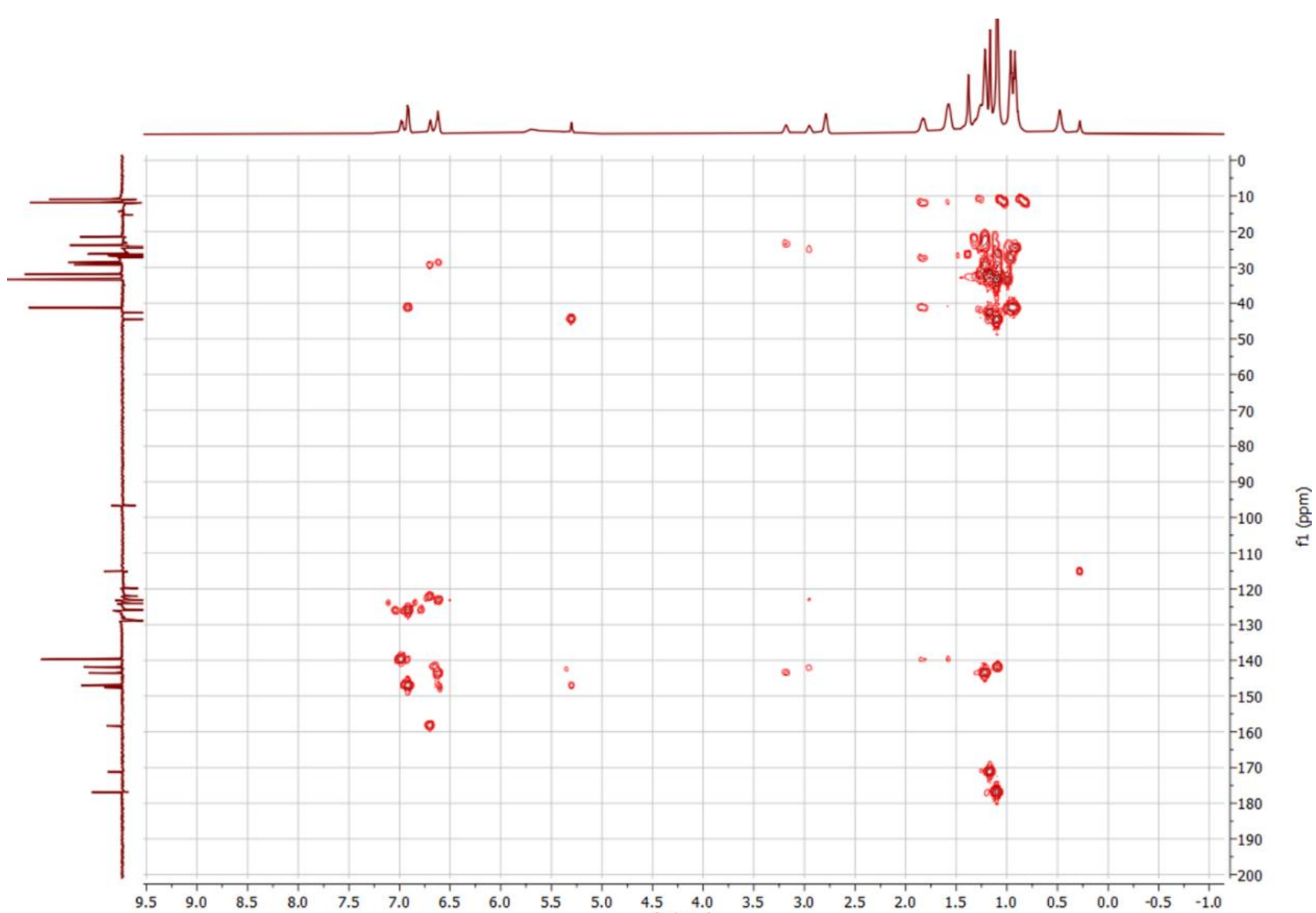

Fig. S15. 2D-HMBC NMR (600/151 MHz, 298 K, C_6D_{12}) spectrum of (BDI*)Mg-[H_2CCPh_2]-Yb(Am*) (**4**-Yb).

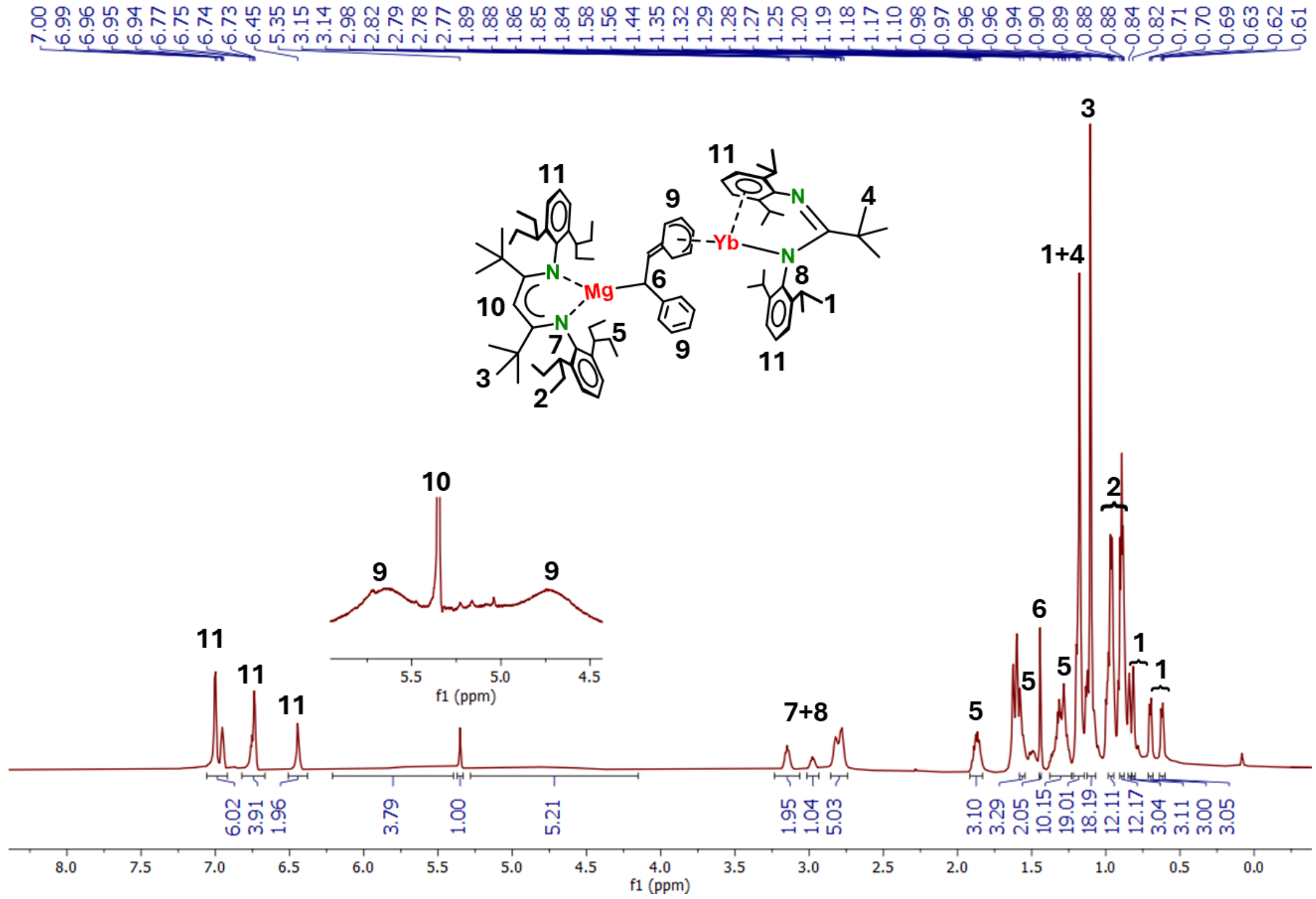

Fig. S16. ^{1}H NMR (600 MHz, 298 K, C_7D_{14}) spectrum of (BDI*)Mg-CH(Ph)(H)C(Ph)-Yb(Am*) (**5**-Yb).

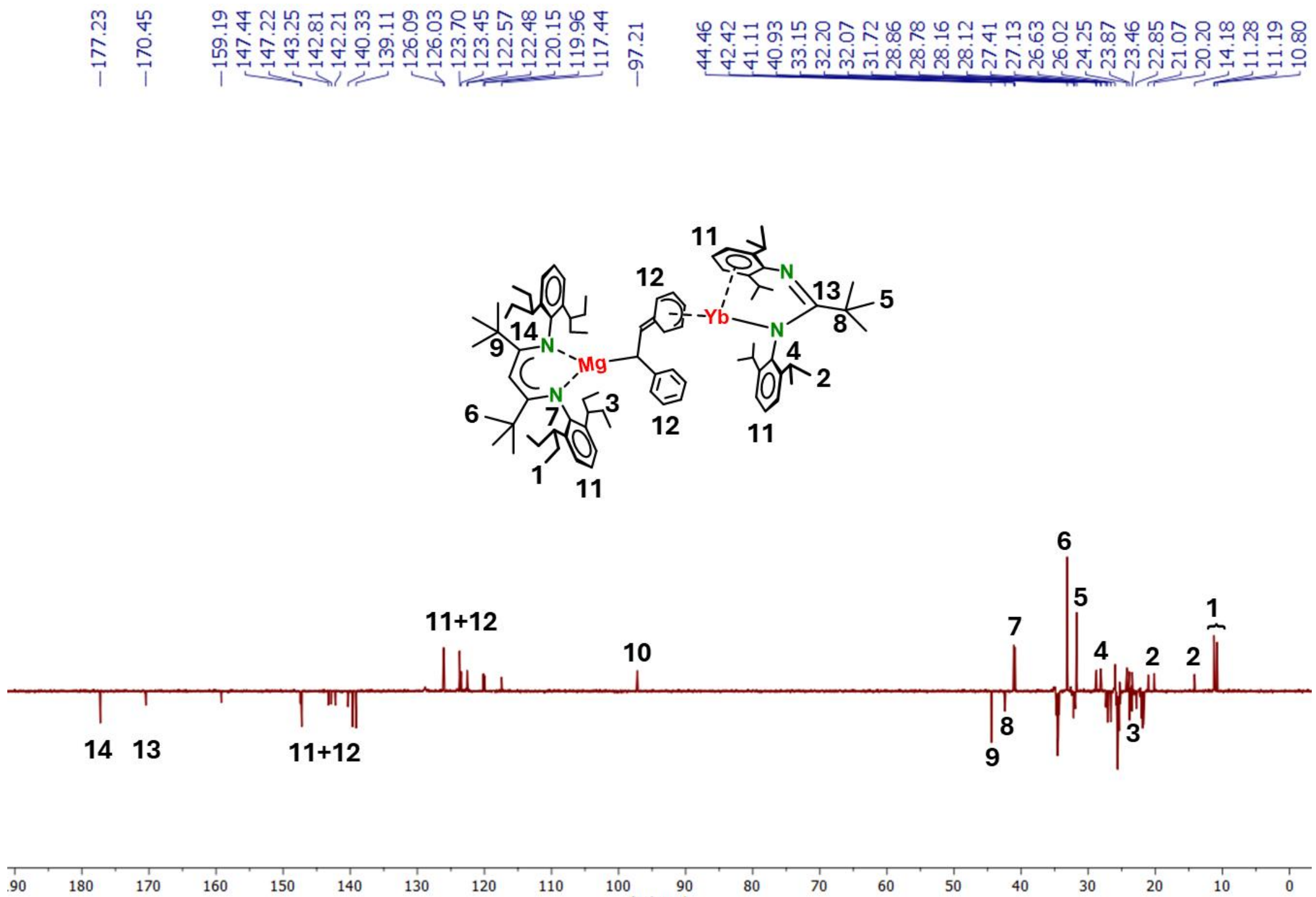

Fig. S17. ^{13}C-APT NMR (151 MHz, 298 K, C_7D_{14}) spectrum of (BDI*)Mg-CH(Ph)(H)C(Ph)-Yb(Am*) (**5**-Yb).

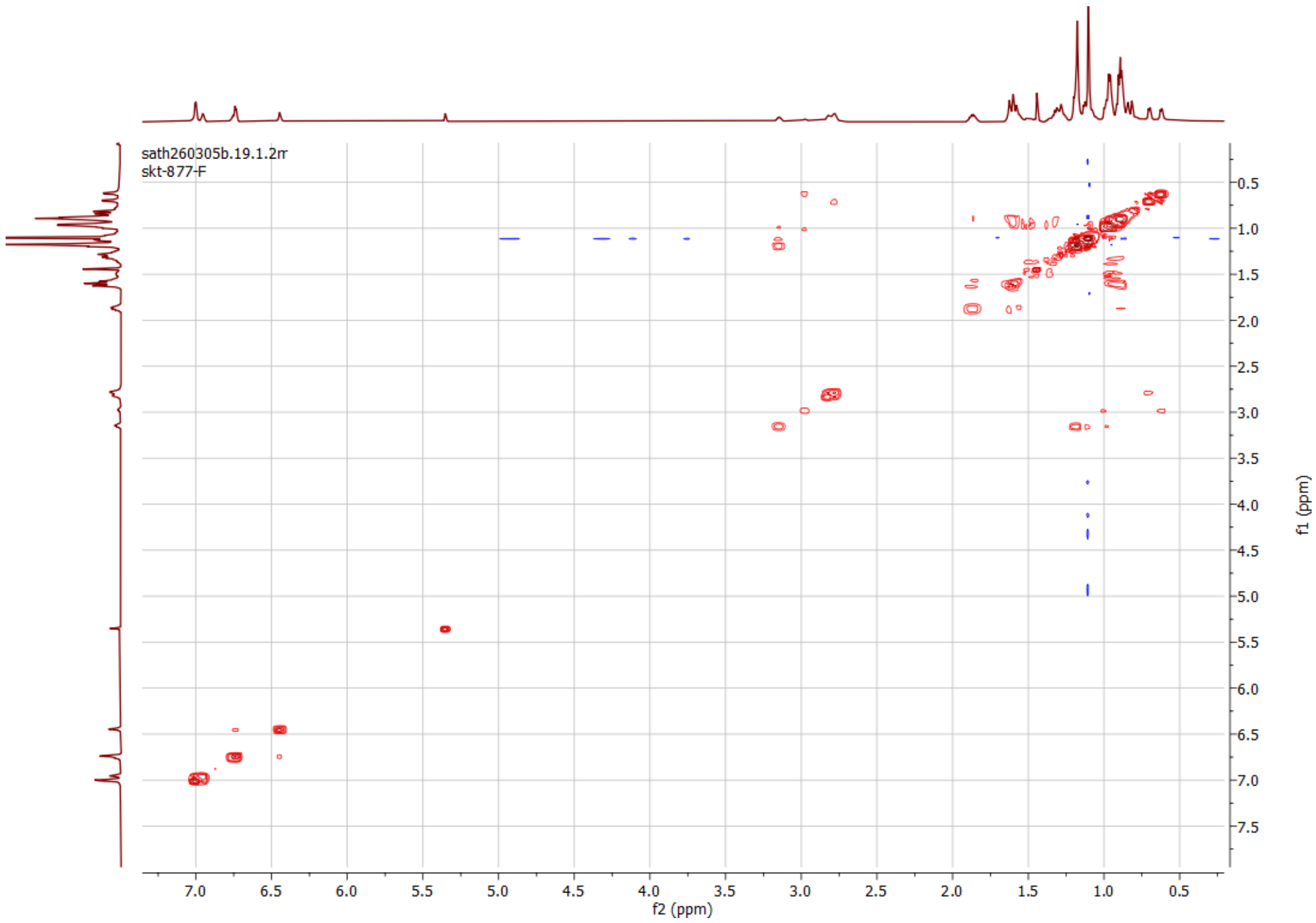

Fig. S18. 2D-COSY NMR (600 MHz, 298 K, C_7D_{14}) spectrum of (BDI*)Mg-CH(Ph)(H)C(Ph)-Yb(Am*) (**5**-Yb).

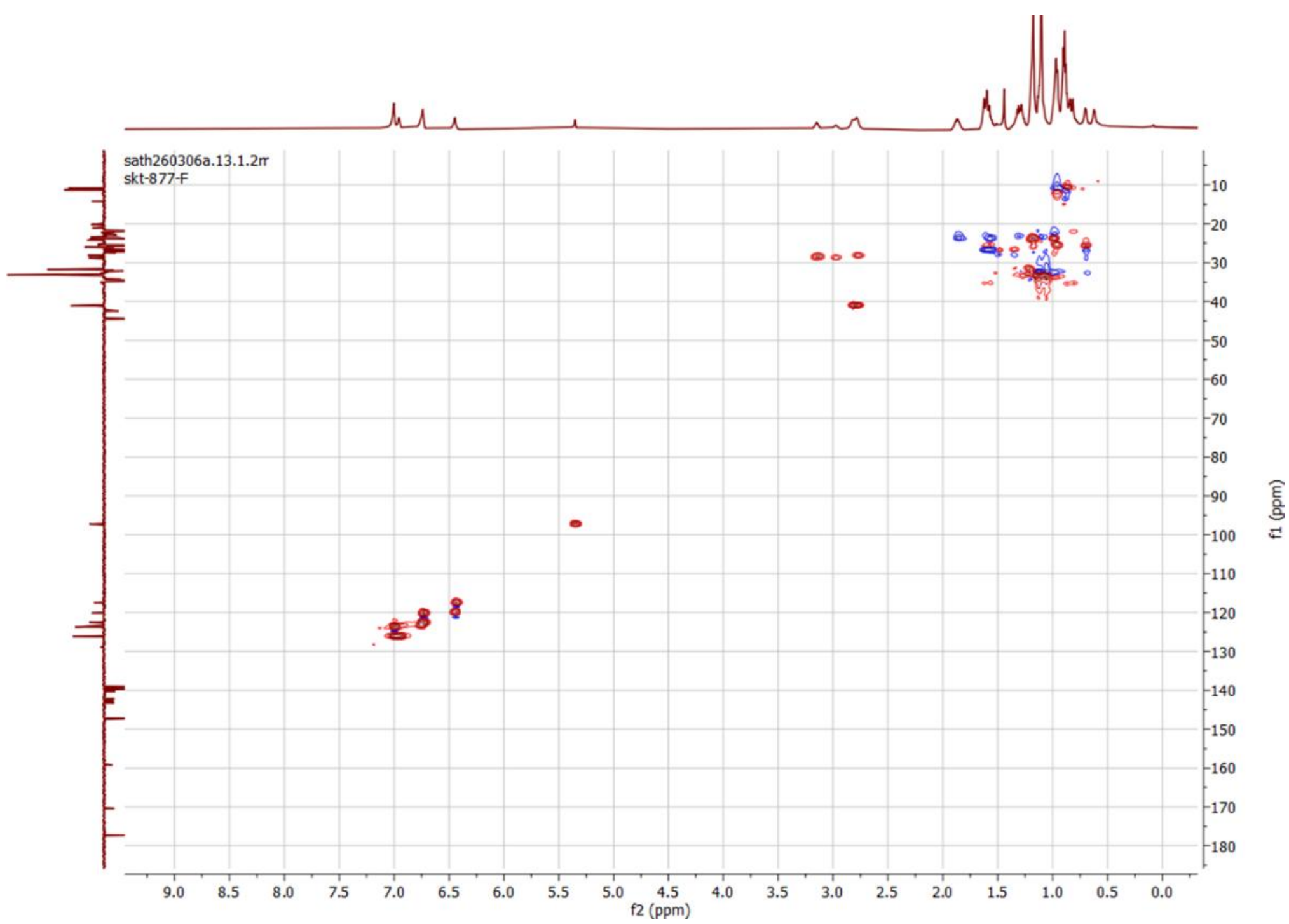

Fig. S19. 2D-HSQC NMR (600/151 MHz, 298 K, C_7D_{14}) spectrum of (BDI*)Mg-CH(Ph)(H)C(Ph)-Yb(Am*) (**5**-Yb).

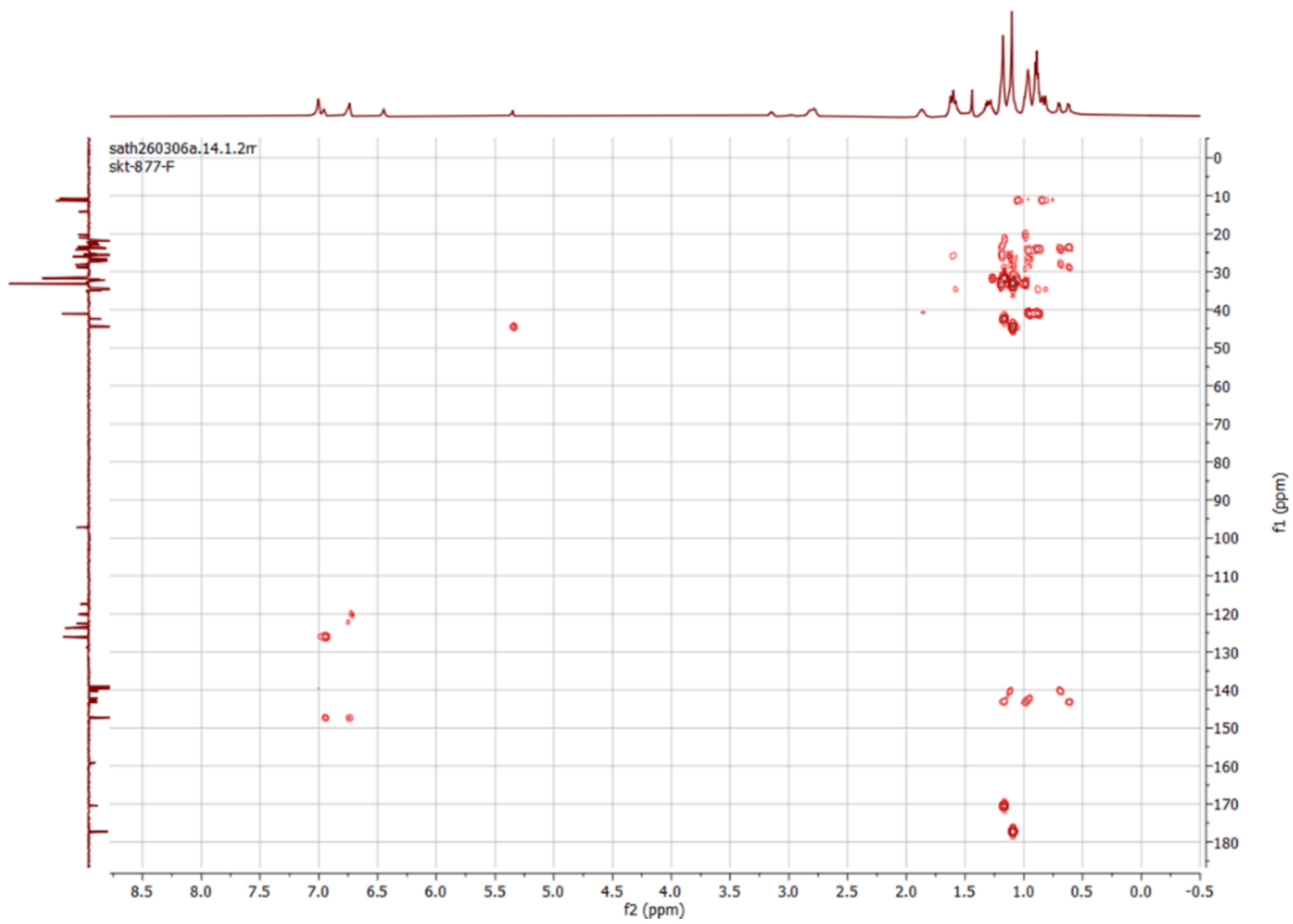

Fig. S20. 2D-HMBC NMR (600/151 MHz, 298 K, C_7D_{14}) spectrum of (BDI*)Mg-CH(Ph)(H)C(Ph)-Yb(Am*) (**5**-Yb).

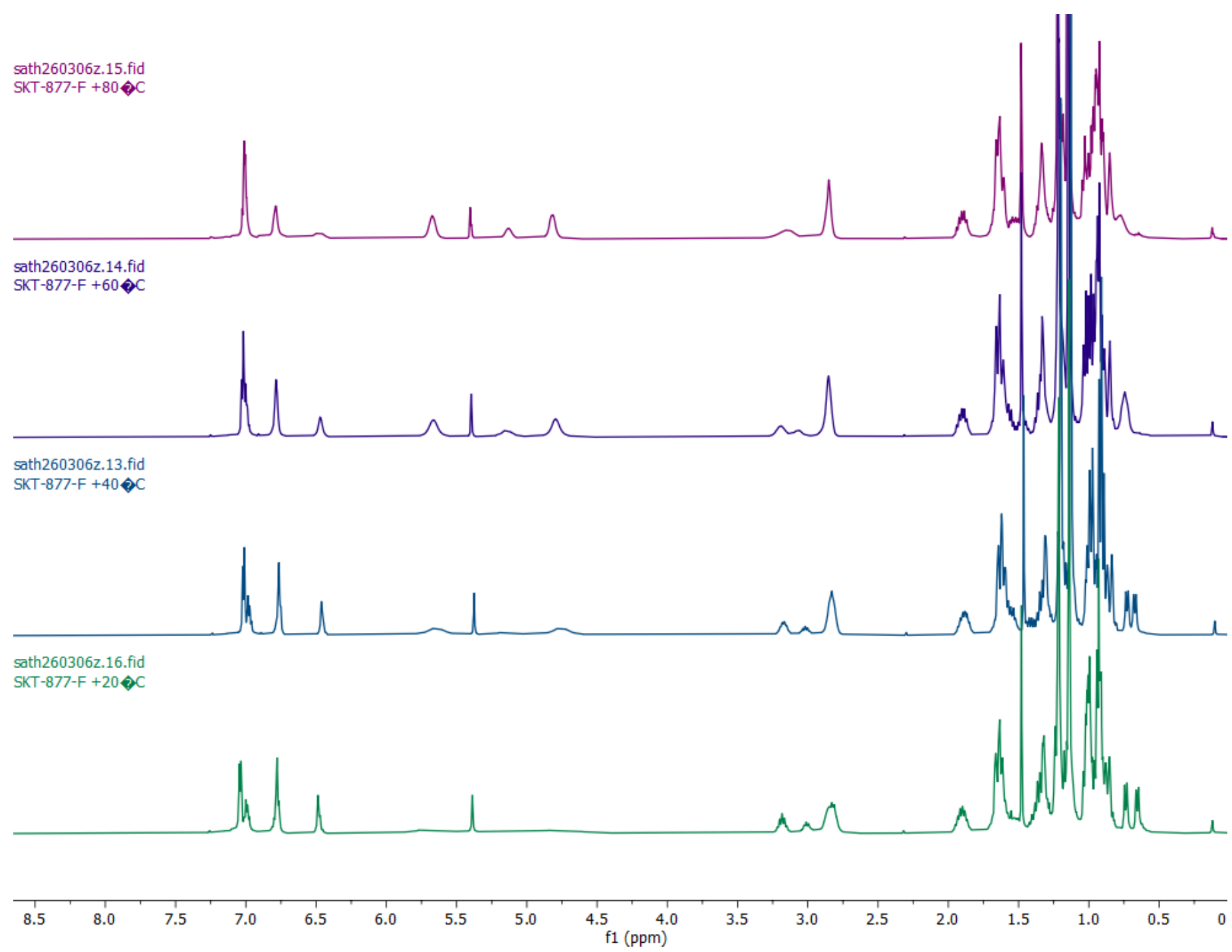

Fig. S21. High-temperature ^{1}H NMR (600 MHz, C_7D_{14}) spectrum of (BDI*)Mg-CH(Ph)(H)C(Ph)-Yb(Am*) (**5**-Yb).

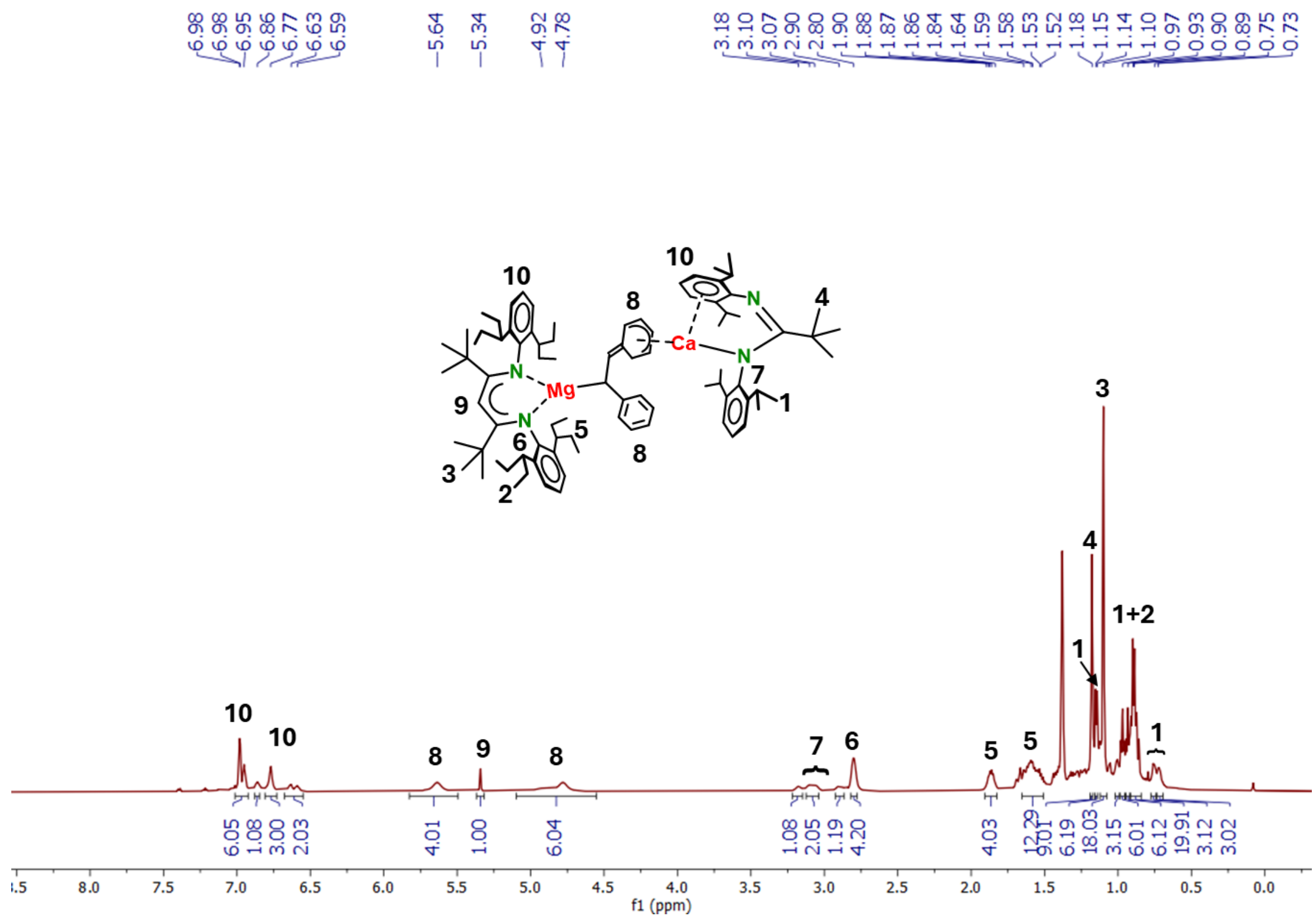

Fig. S22. ^{1}H NMR (600 MHz, 298 K, C_6D_{12}) spectrum of (BDI*)Mg-CH(Ph)(H)C(Ph)-Ca(Am*) (**5**-Ca).

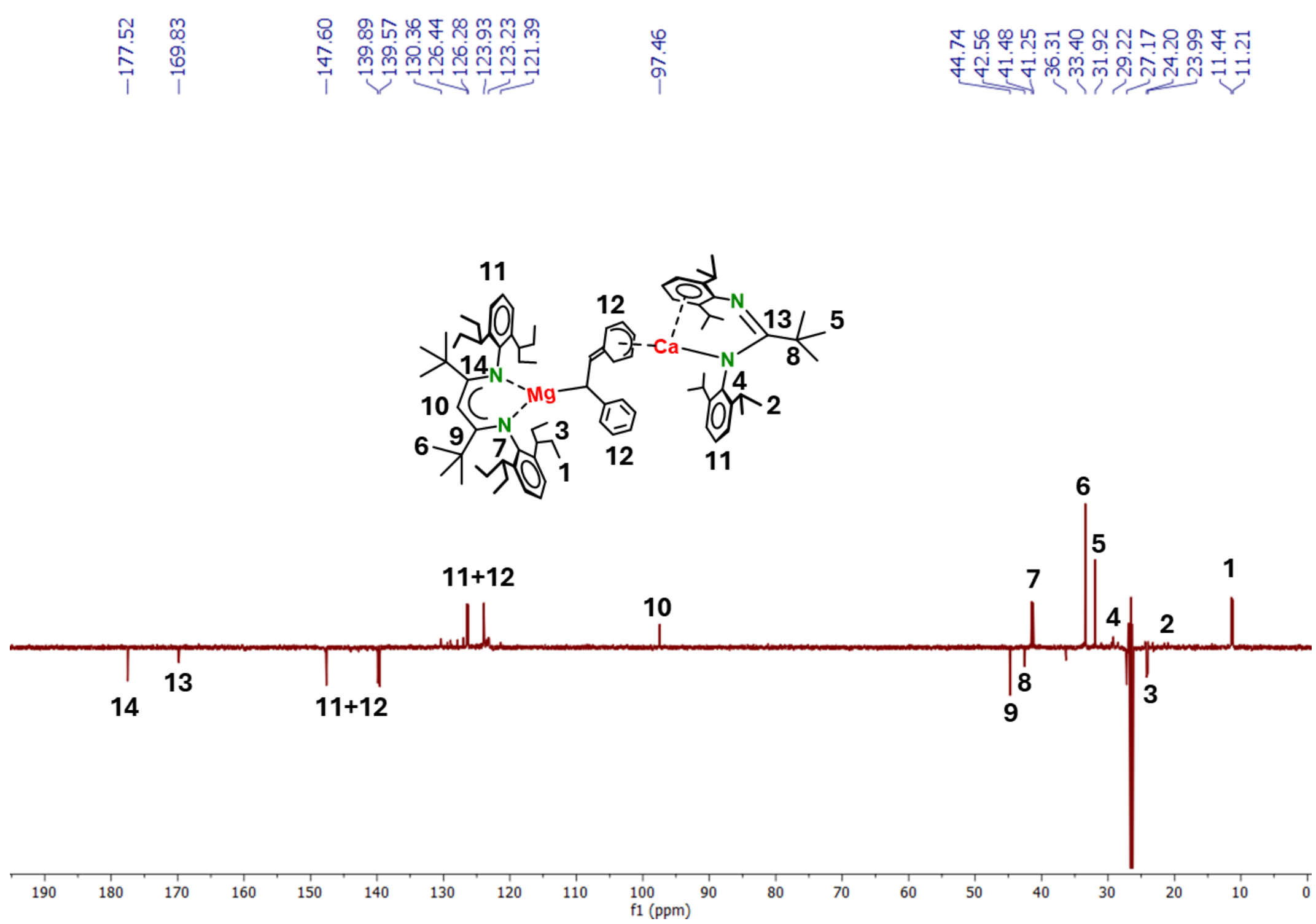

Fig. S23. ^{13}C-APT NMR (151 MHz, 298 K, C_6D_{12}) spectrum of (BDI*)Mg-CH(Ph)(H)C(Ph)-Ca(Am*) (**5**-Ca).

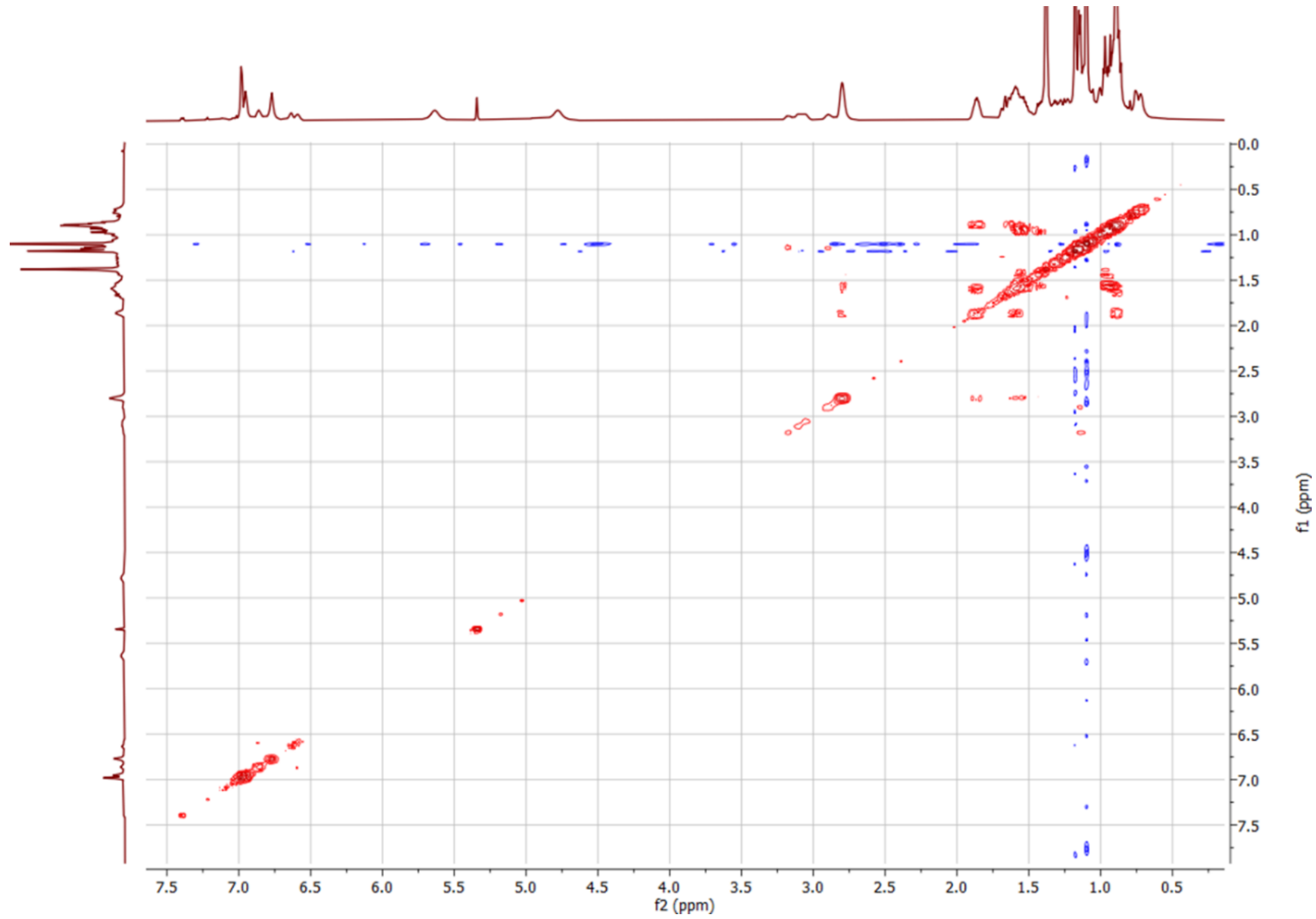

Fig. S24. 2D-COSY NMR (600 MHz, 298 K, C_6D_{12}) spectrum of (BDI*)Mg-CH(Ph)(H)C(Ph)-Ca(Am*) (**5**-Ca).

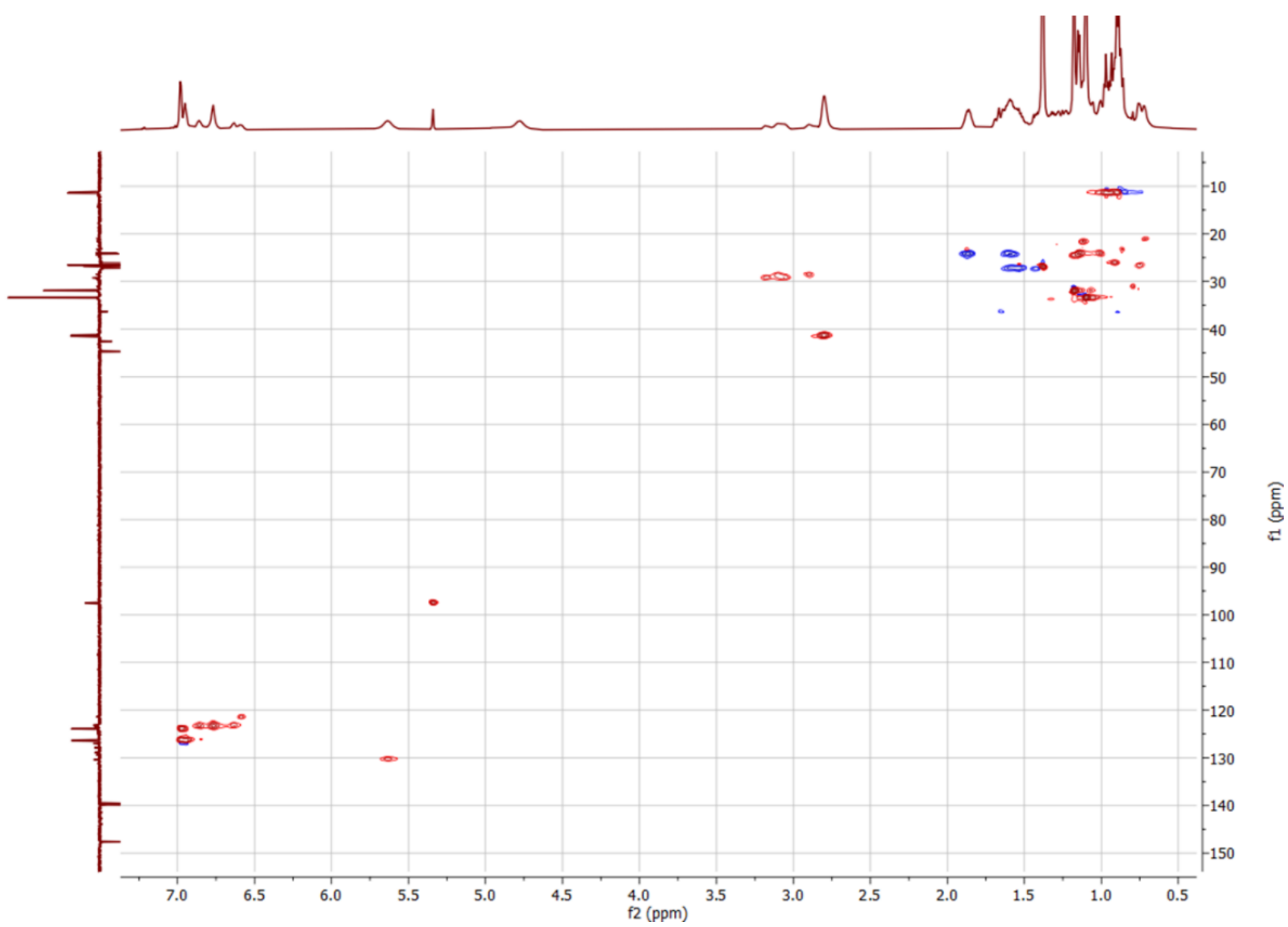

Fig. S25. 2D-HSQC NMR (600/151 MHz, 298 K, C_6D_{12}) spectrum of (BDI*)Mg-CH(Ph)(H)C(Ph)-Ca(Am*) (**5**-Ca).

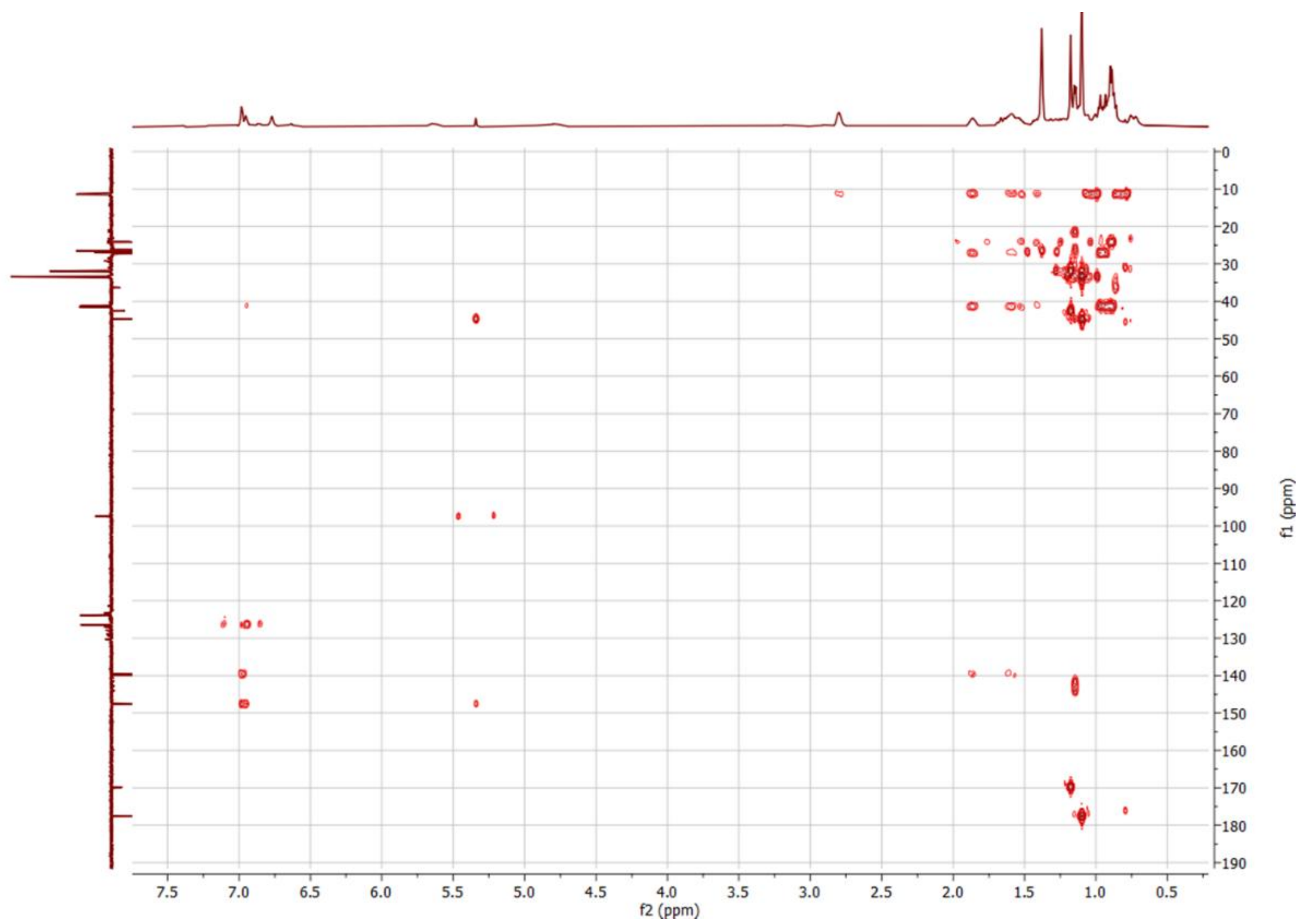

Fig. S26. 2D-HMBC NMR (600/151 MHz, 298 K, C_6D_{12}) spectrum of (BDI*)Mg-CH(Ph)(H)C(Ph)-Ca(Am*) (**5**-Ca).

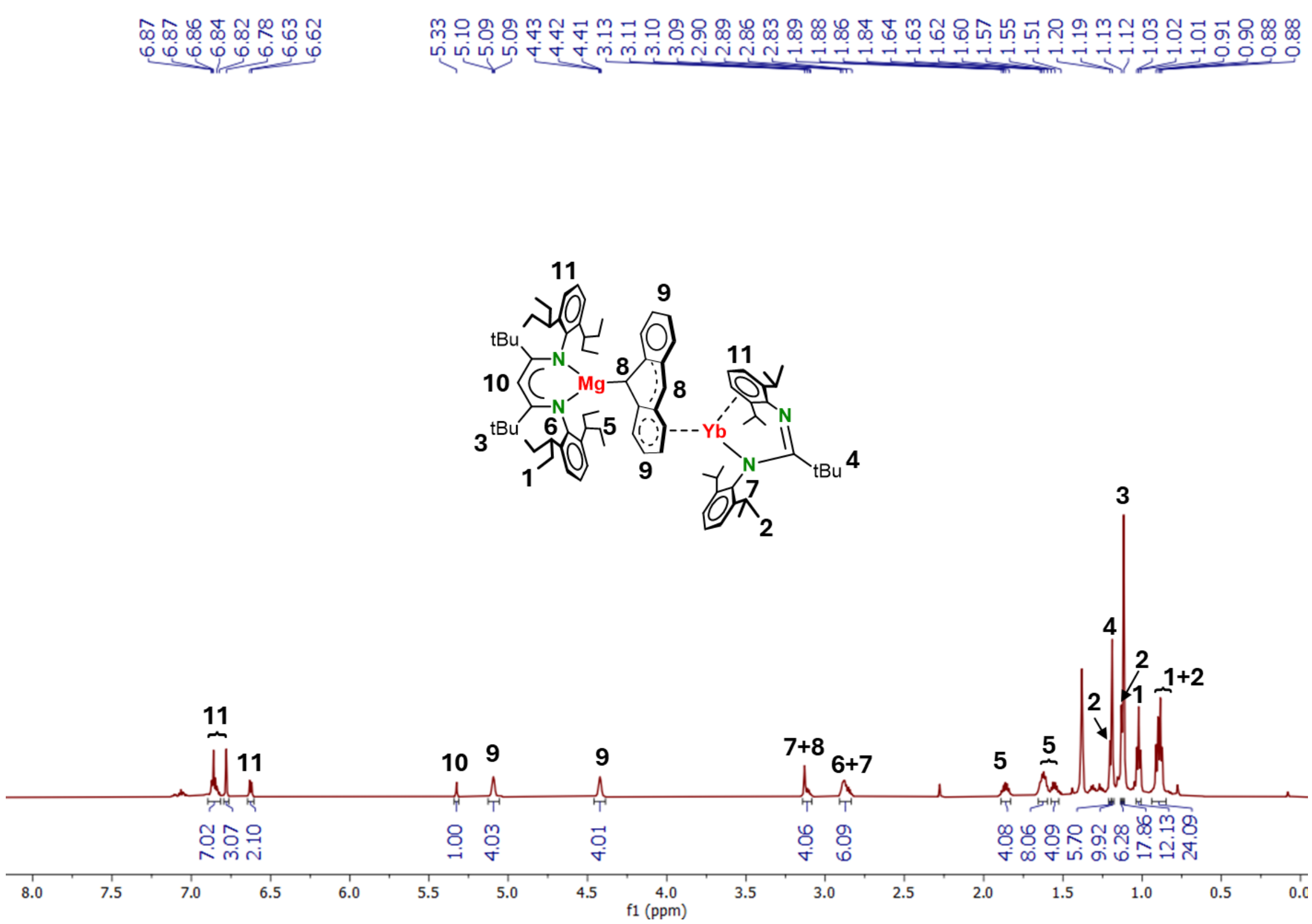

Fig. S27. ^{1}H NMR (600 MHz, 298 K, C_6D_{12}) spectrum of (BDI*)Mg-anthracene-Yb(Am*) (**6**-Yb).

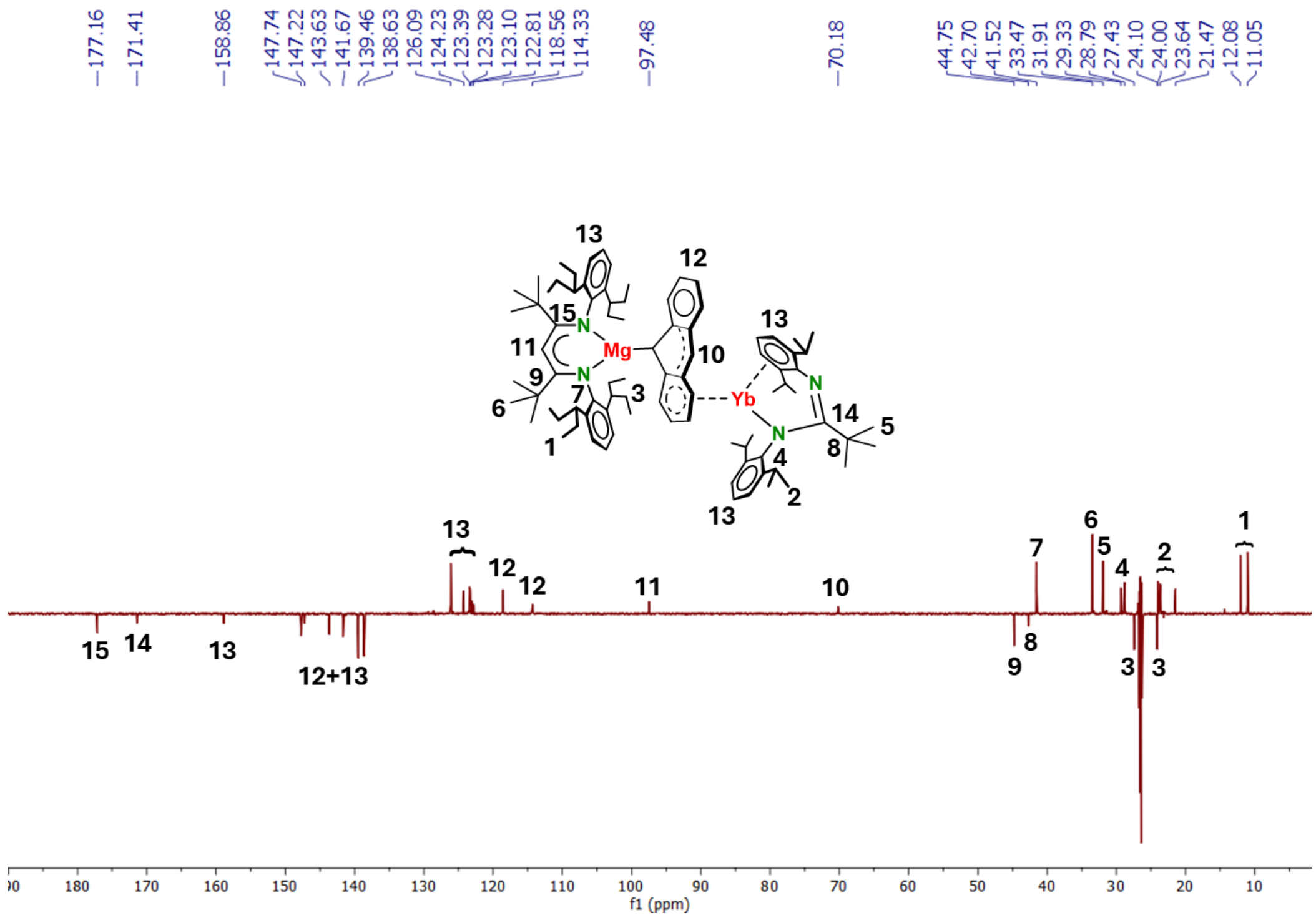

Fig. S28. ^{13}C-APT NMR (151 MHz, 298 K, C_6D_{12}) spectrum of (BDI*)Mg-anthracene-Yb(Am*) (**6**-Yb).

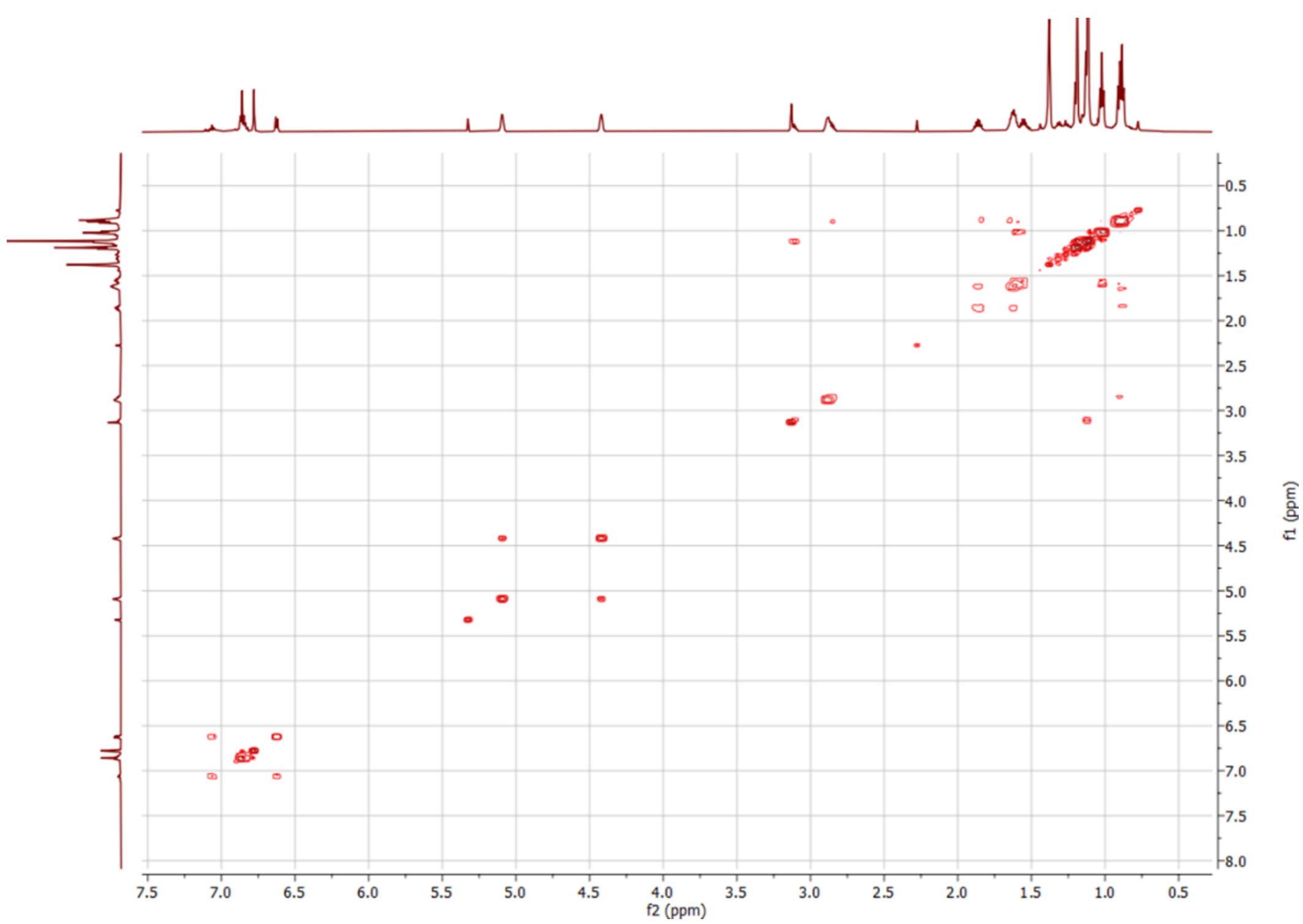

Fig. S29. 2D-COSY NMR (600 MHz, 298 K, C_6D_{12}) spectrum of (BDI*)Mg-anthracene-Yb(Am*) (**6**-Yb).

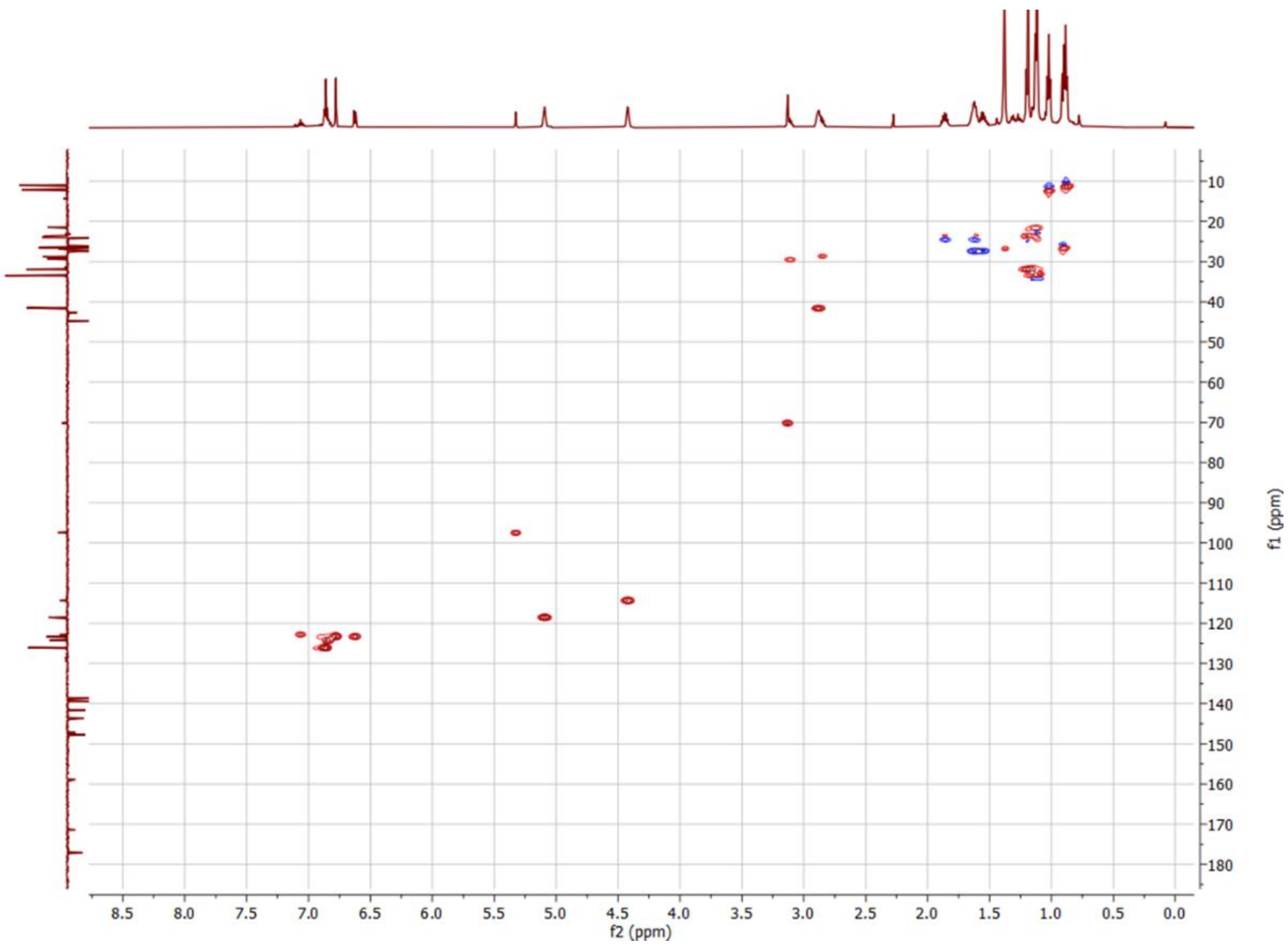

Fig. S30. 2D-HSQC NMR (600/151 MHz, 298 K, C_6D_{12}) spectrum of (BDI*)Mg-anthracene-Yb(Am*) (**6**-Yb).

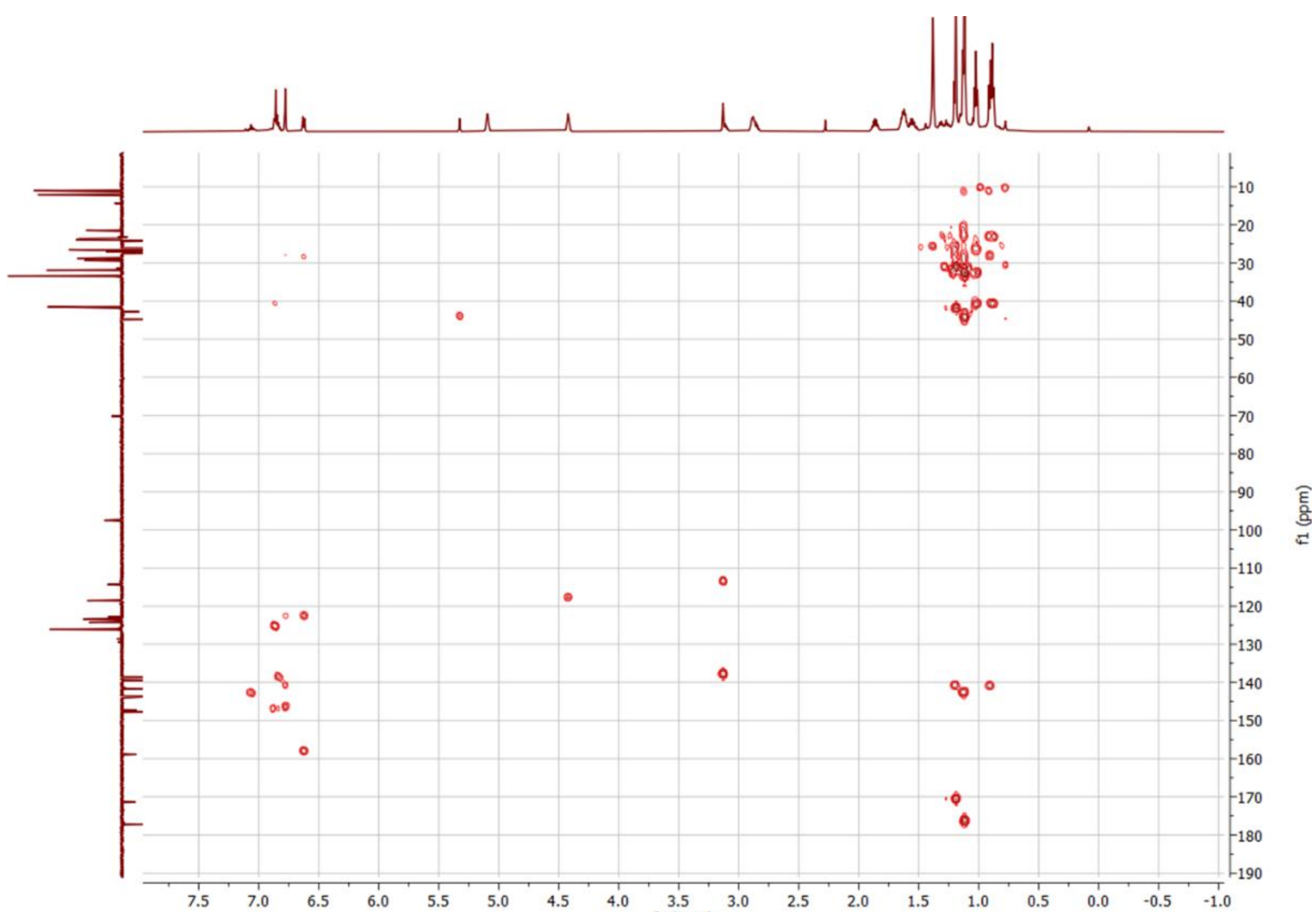

Fig. S31. 2D-HMBC NMR (600/151 MHz, 298 K, C_6D_{12}) spectrum of (BDI*)Mg-anthracene-Yb(Am*) (**6**-Yb).

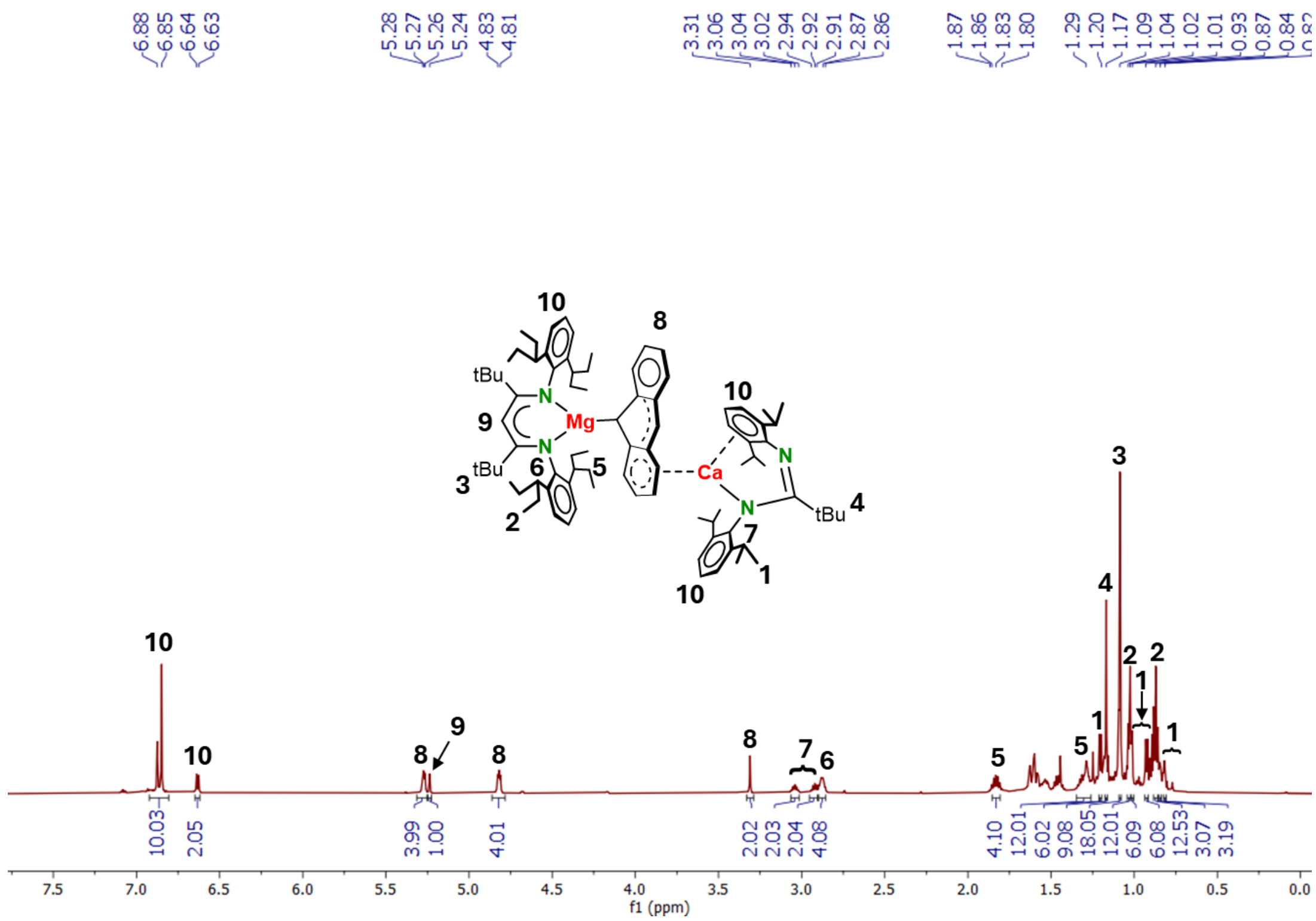

Fig. S32. ^{1}H NMR (600 MHz, 298 K, C_7D_{14}) spectrum of (BDI*)Mg-anthracene-Ca(Am*) (**6**-Ca).

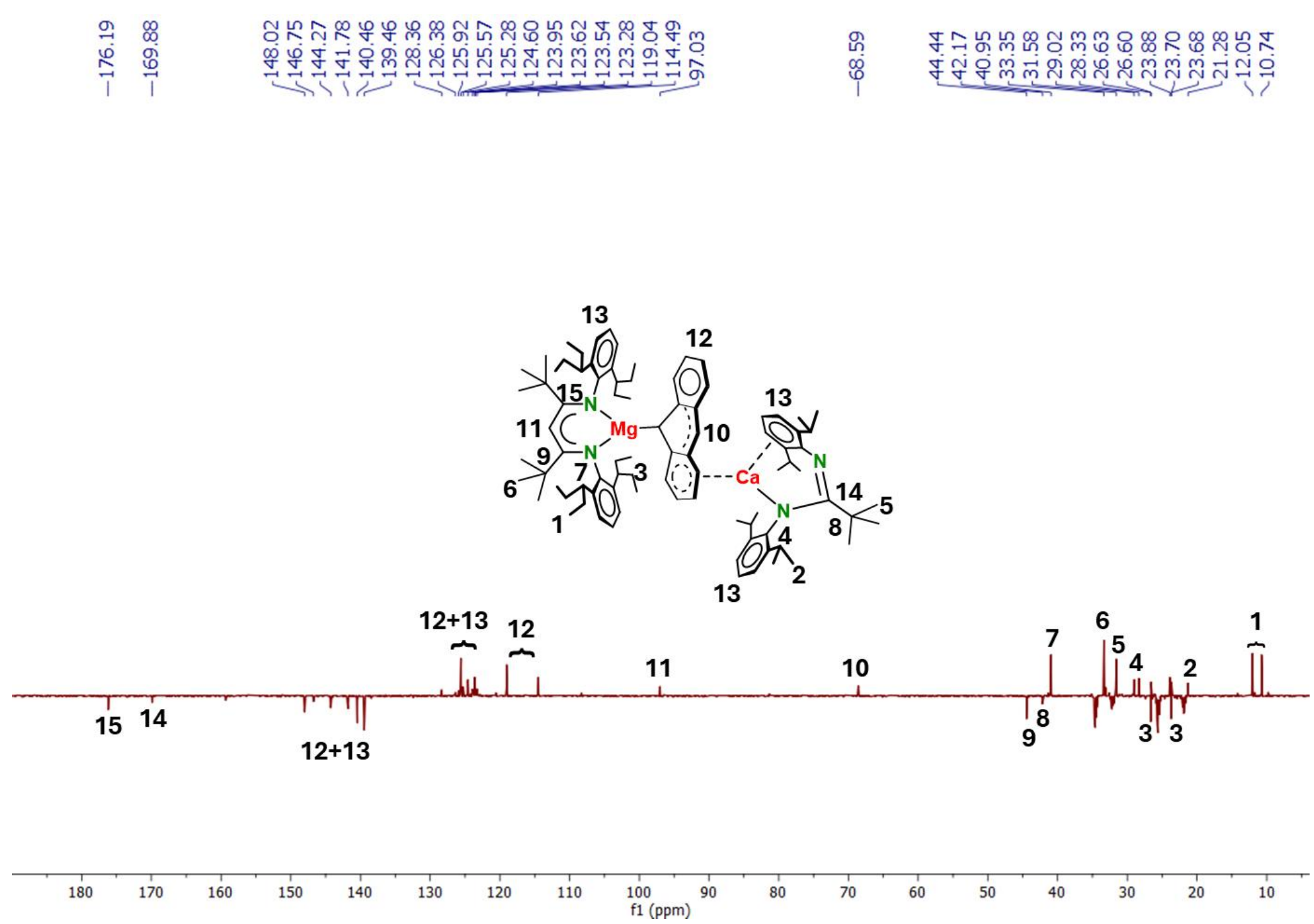

Fig. S33. ^{13}C-APT NMR (151 MHz, 298 K, C_7D_{14}) spectrum of (BDI*)Mg-anthracene-Ca(Am*) (**6**-Ca).

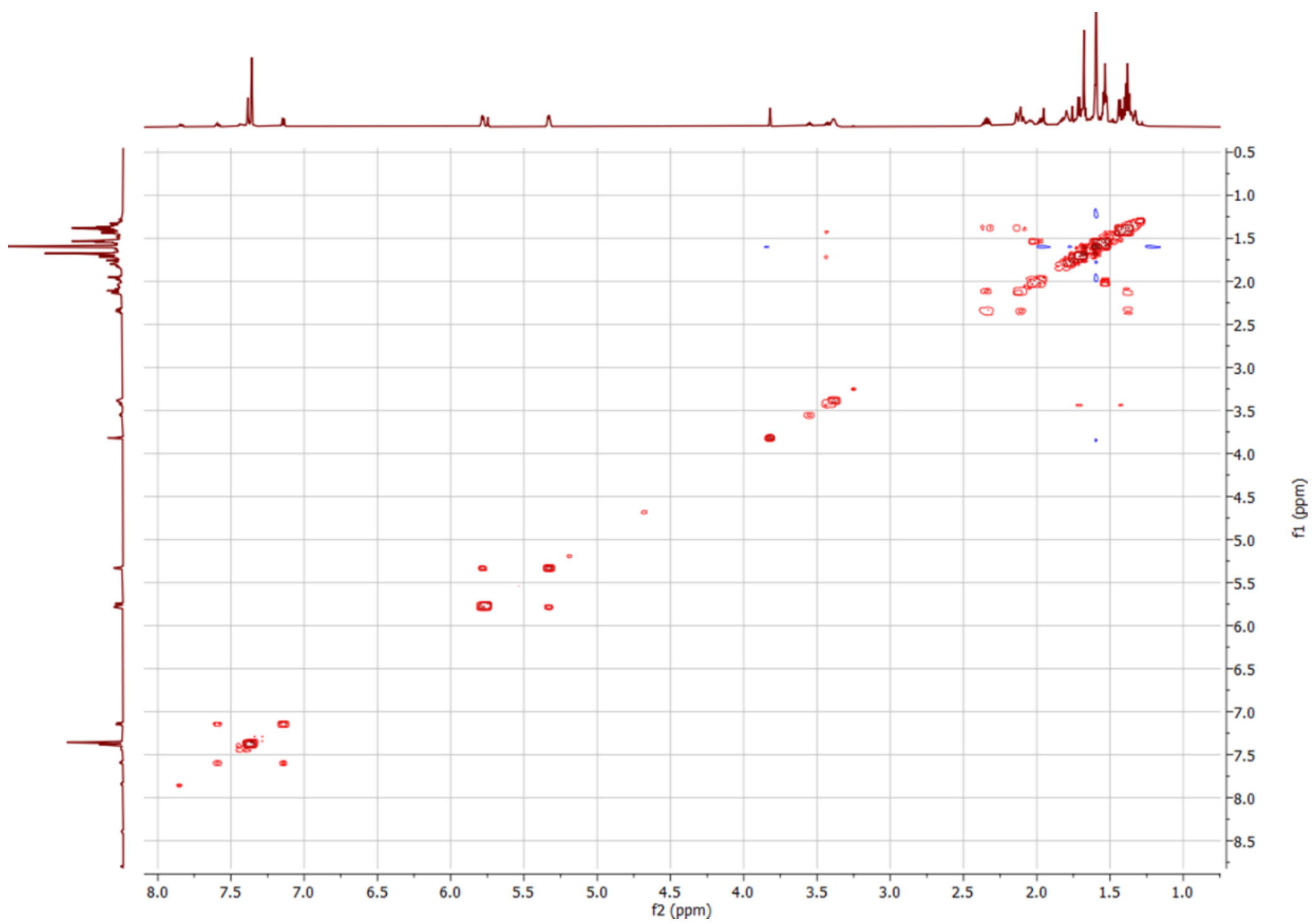

Fig. S34. 2D-COSY NMR (600 MHz, 298 K, C_6D_{12}) spectrum of (BDI*)Mg-anthracene-Ca(Am*) (**6**-Ca).

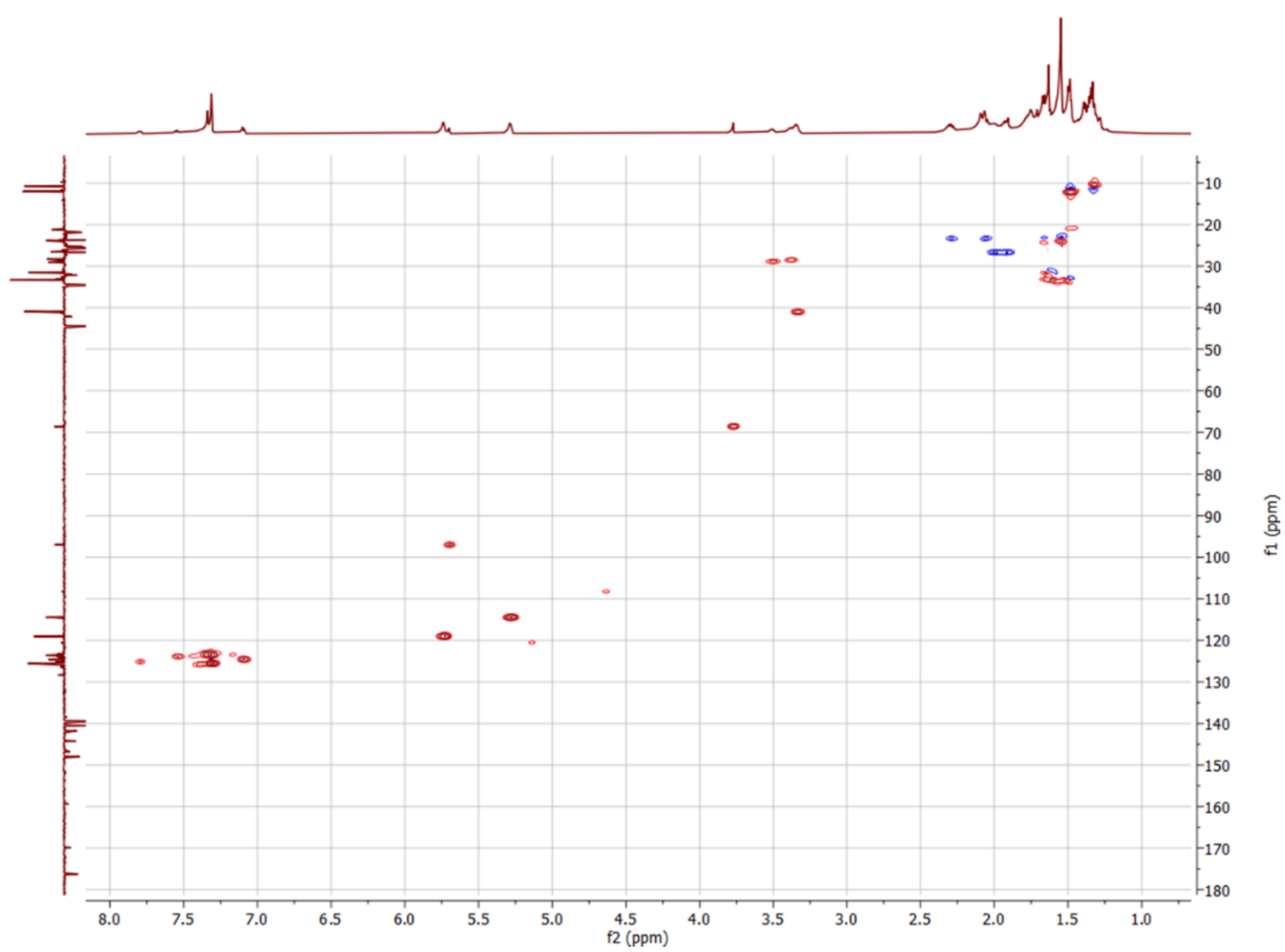

Fig. S35. 2D-HSQC NMR (600/151 MHz, 298 K, C_6D_{12}) spectrum of (BDI*)Mg-anthracene-Ca(Am*) (**6**-Ca).

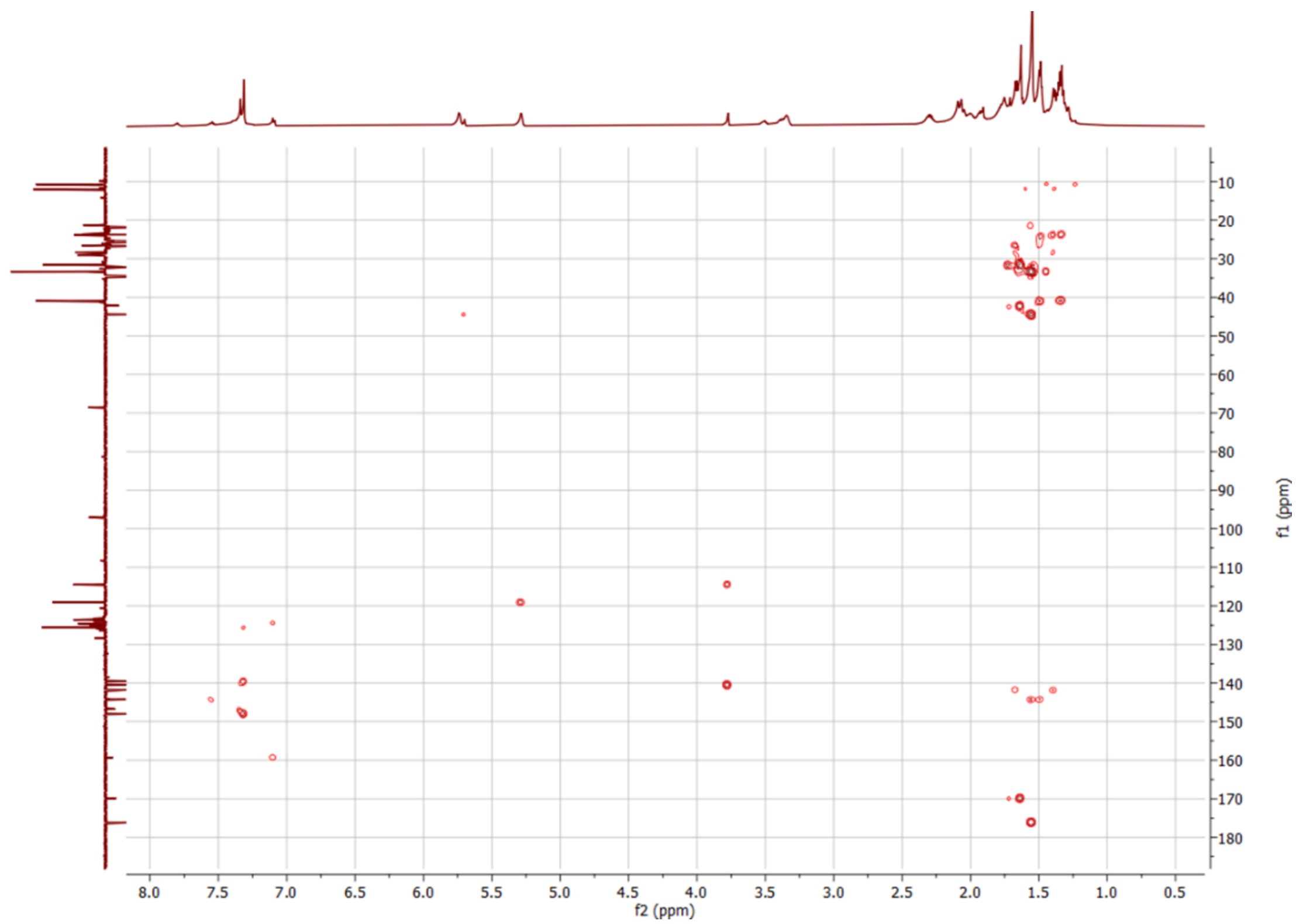

Fig. S36. 2D-HMBC NMR (600/151 MHz, 298 K, C_6D_{12}) spectrum of (BDI*)Mg-anthracene-Ca(Am*) (**6**-Ca).

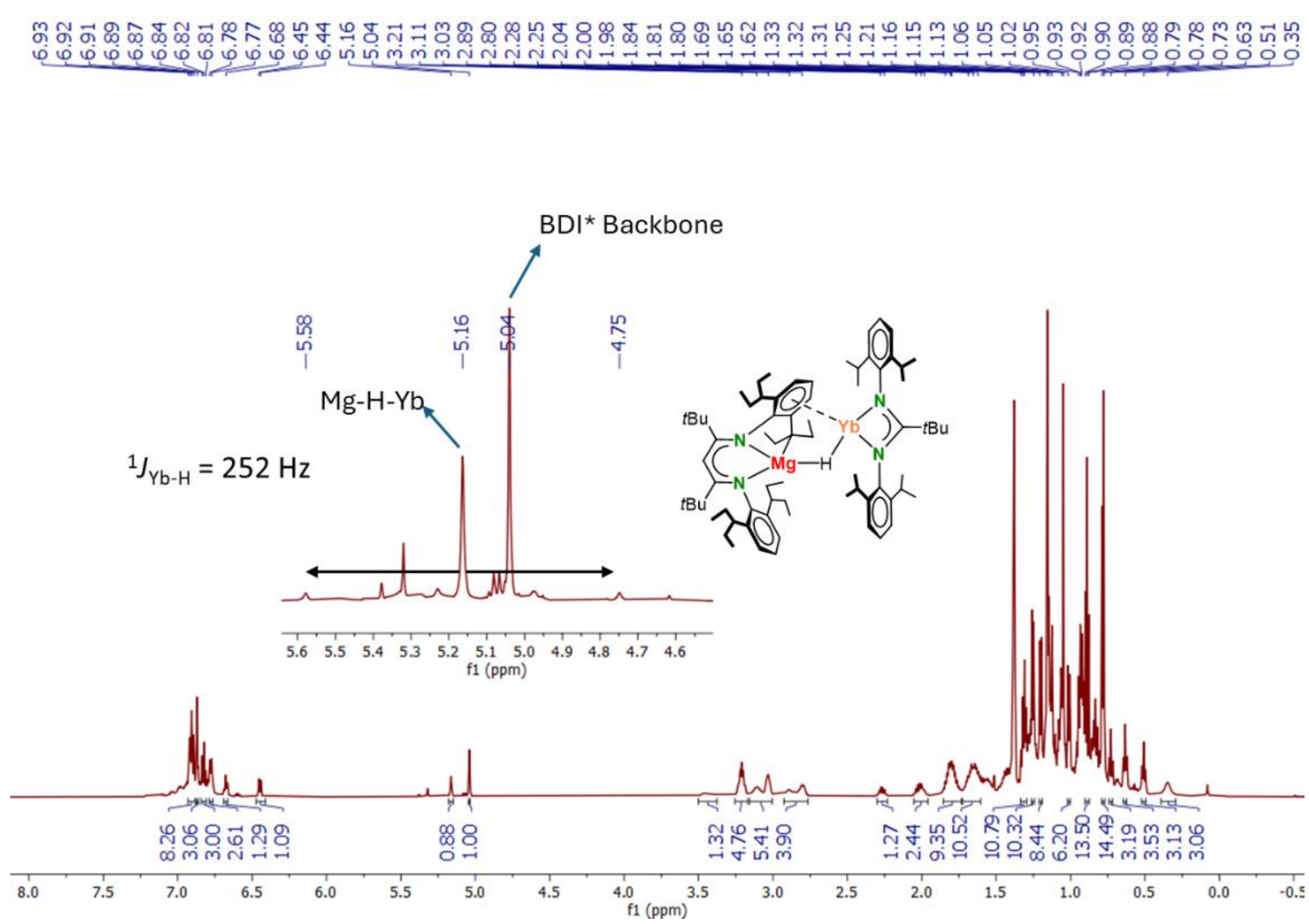

Fig. S37. ^{1}H NMR (600 MHz, 298 K, C_6D_{12}) spectrum of decomposed (BDI*)Mg-Yb(Am*) (**1**-Yb) including a tentative peak assignment.

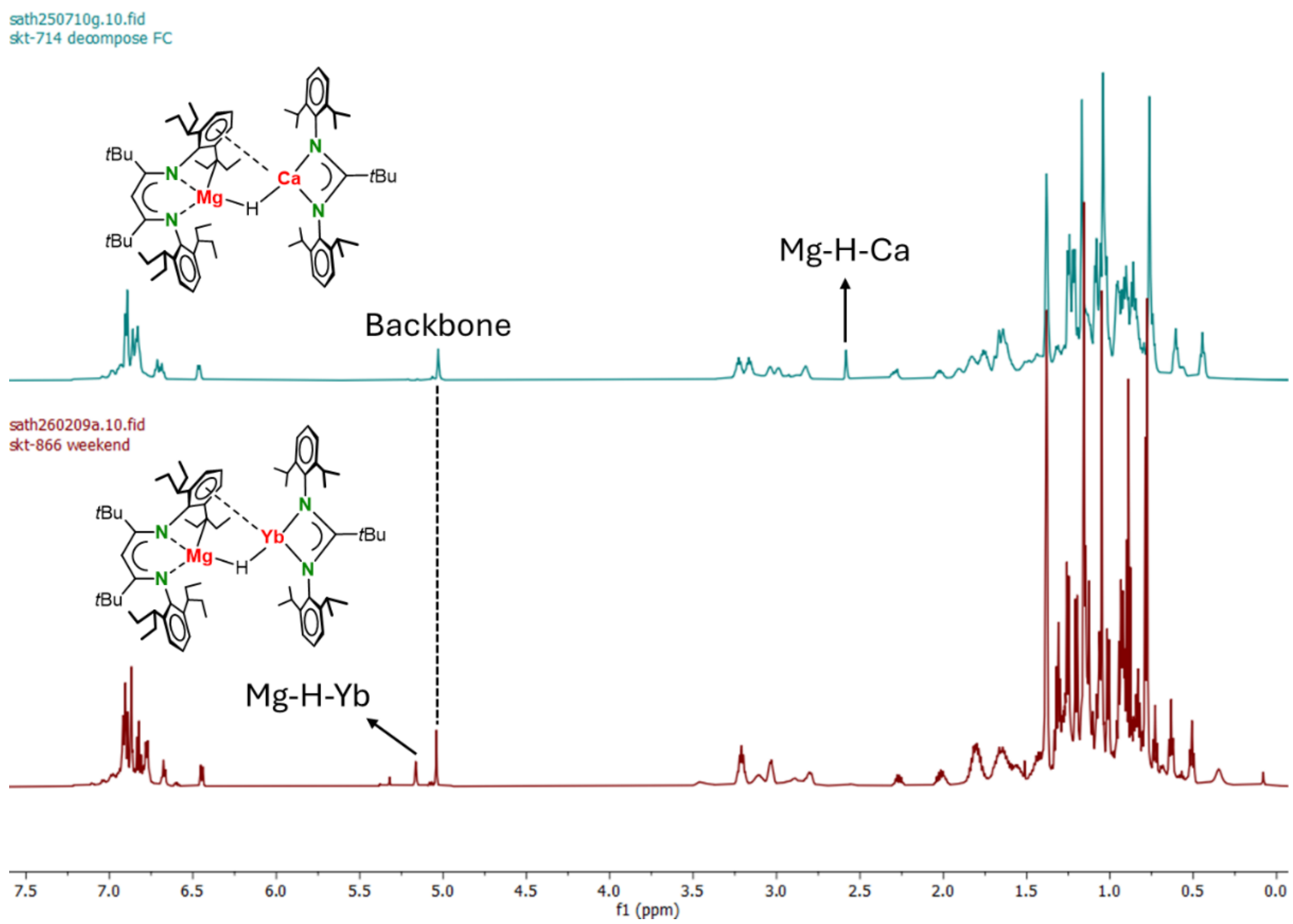

Fig. S38. Comparison of ^{1}H NMR (600 MHz, 298 K, C_6D_{12}) spectrum of the decomposition products of (BDI*)Mg-Yb(Am*) (**1**-Yb) (bottom) and (BDI*)Mg-Ca(Am*) (**1**-Ca) (top) including a tentative peak assignment.

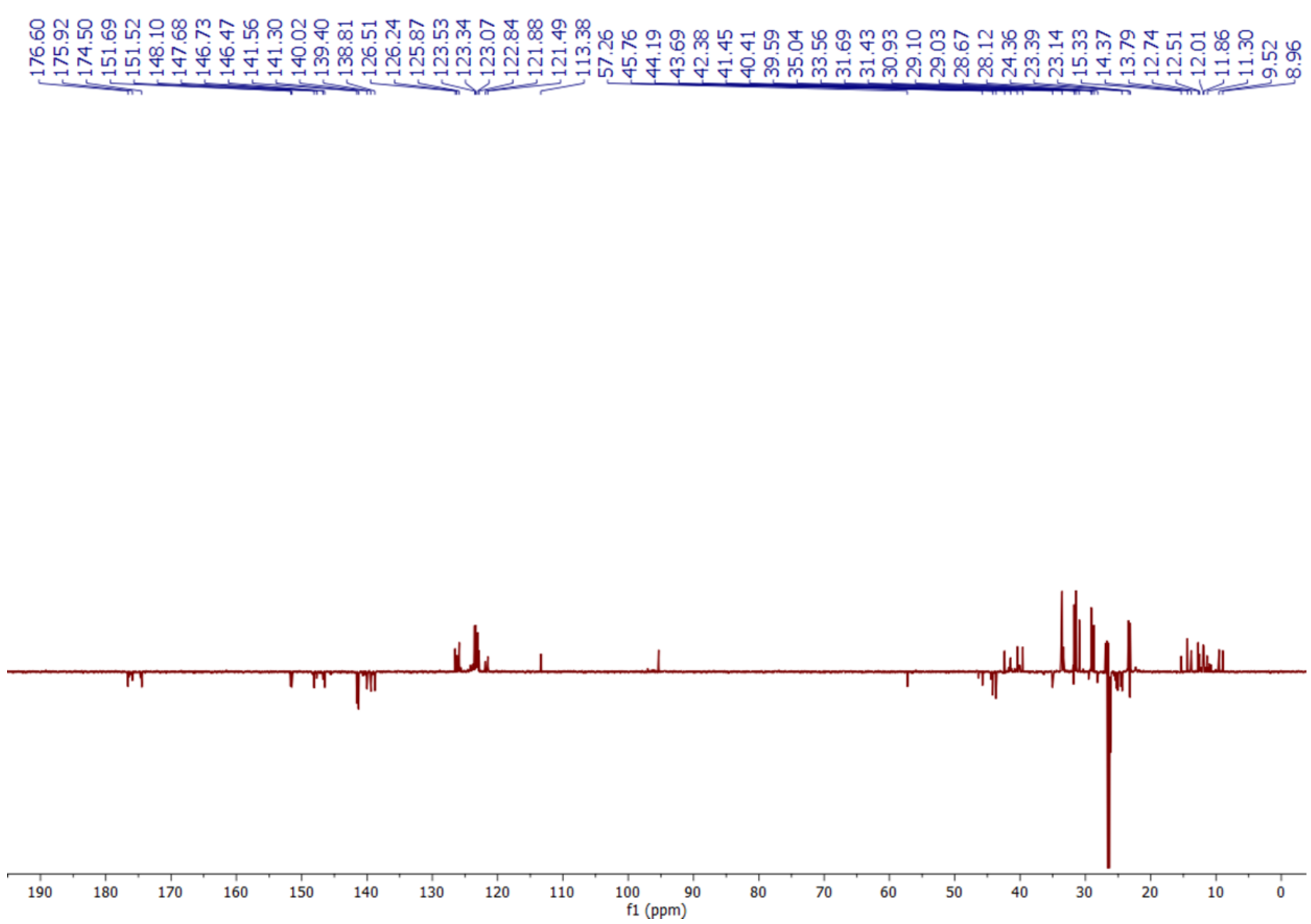

Fig. S39. ^{13}C-APT NMR (151 MHz, 298 K, C_6D_{12}) spectrum of decomposition product of (BDI*)Mg-Yb(Am*) (**1**-Yb).

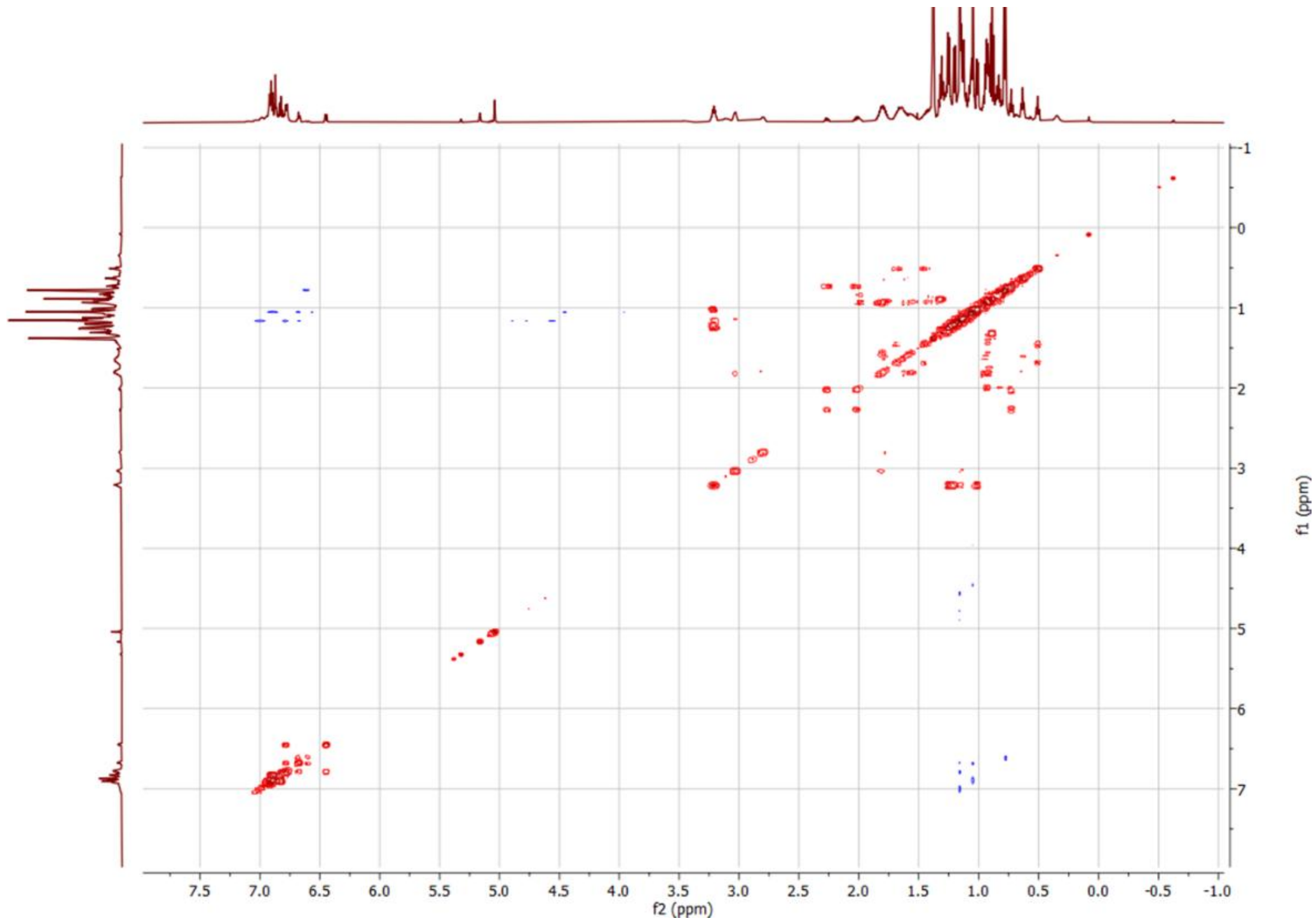

Fig. S40. 2D-COSY NMR (600 MHz, 298 K, C_6D_{12}) spectrum of the decomposition product of (BDI*)Mg-Yb(Am*) (**1**-Yb).

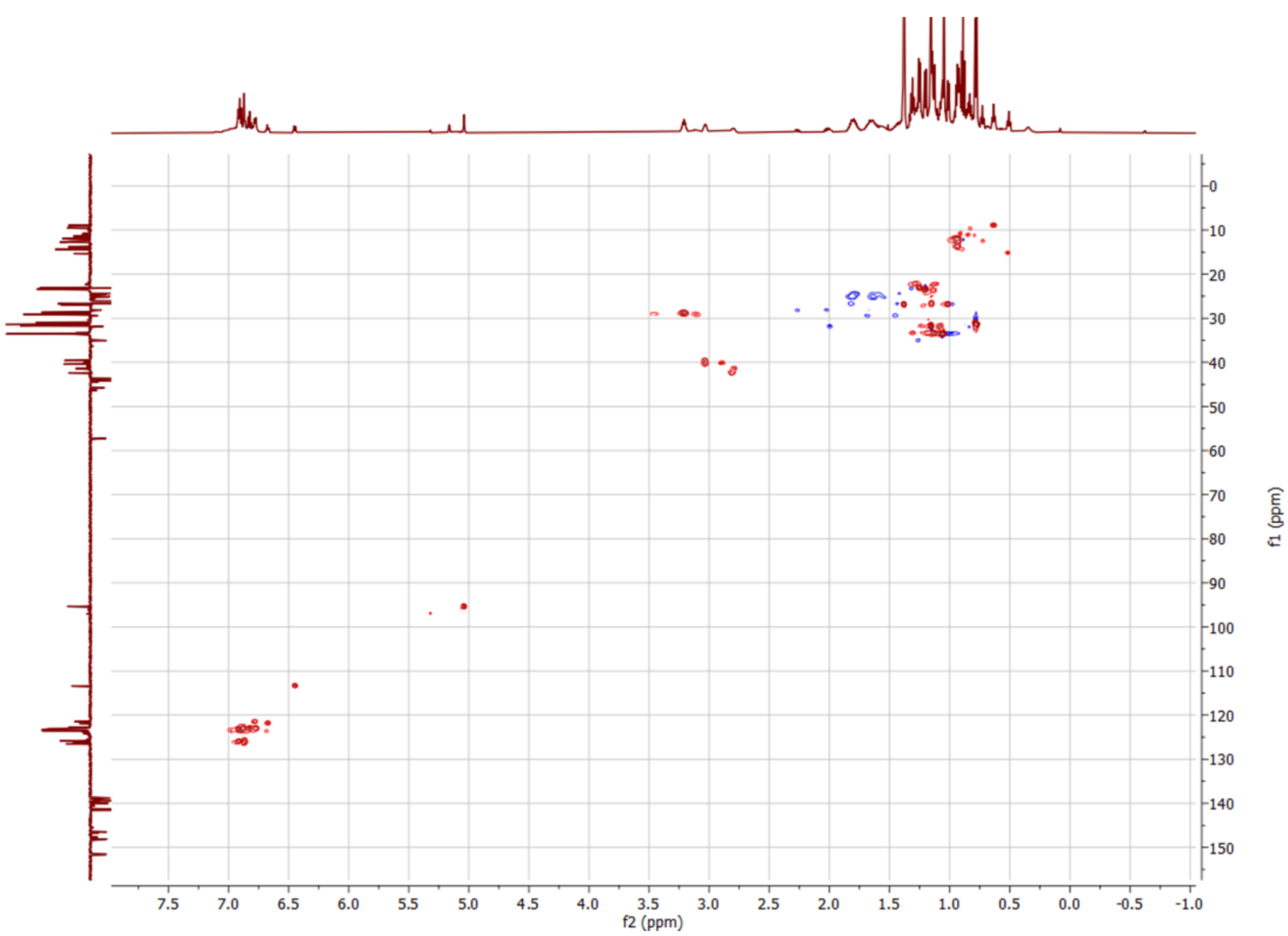

Fig. S41. 2D-HSQC NMR (600/151 MHz, 298 K, C_6D_{12}) spectrum of the decomposition product of (BDI*)Mg-Yb(Am*) (**1**-Yb).

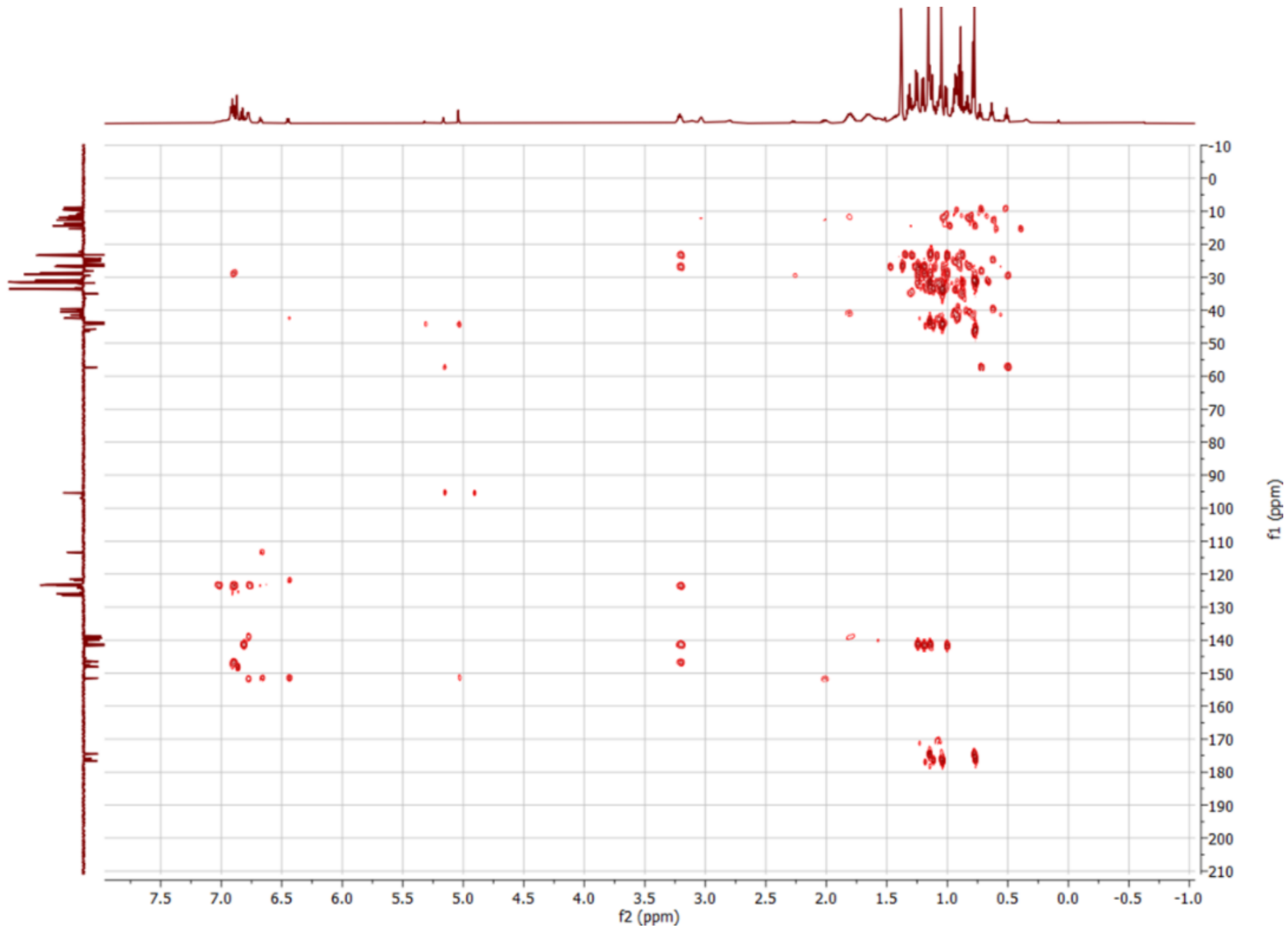

Fig. S42. 2D-HMBC NMR (600/151 MHz, 298 K, C_6D_{12}) spectrum of the decomposition product of (BDI*)Mg-Yb(Am*) (**1**-Yb).

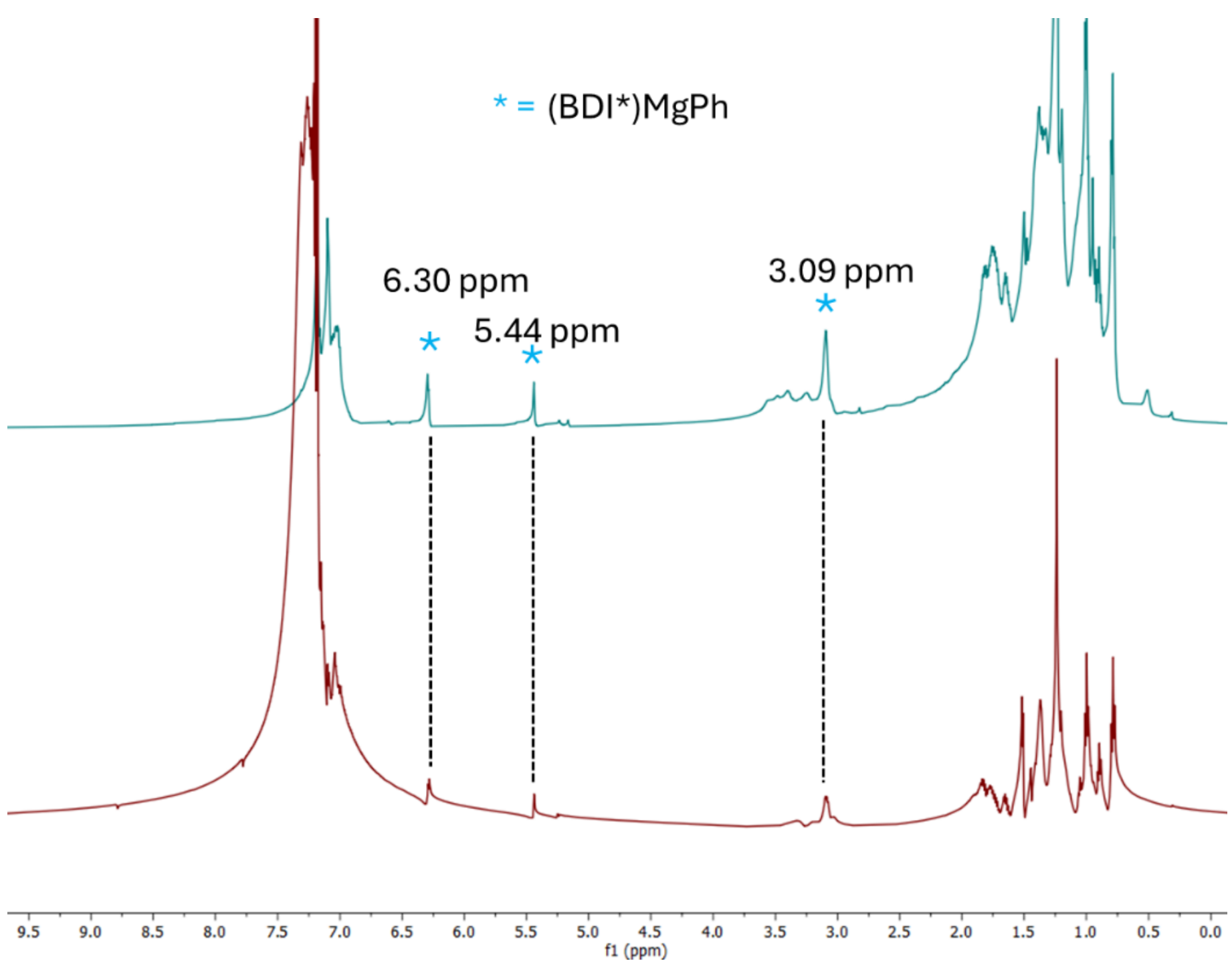

Fig. S43. ^{1}H NMR (600 MHz, 298 K, C_6D_6) spectrum of reaction product of (BDI*)Mg-Ca(Am*) (**1**-Ca) (Top) and (BDI*)Mg-Yb(Am*) (**1**-Yb) (Bottom) with C_6H_6. The reaction mixture in both cases shows reductive cleavage of C-H bond of benzene and formation of previously reported (BDI*)MgPh[1] and (Am*)MH (M = Ca, Yb).[4,5] Due to poor solubility of (Am*)CaH and (Am*)YbH in benzene, corresponding signals could not be identified.

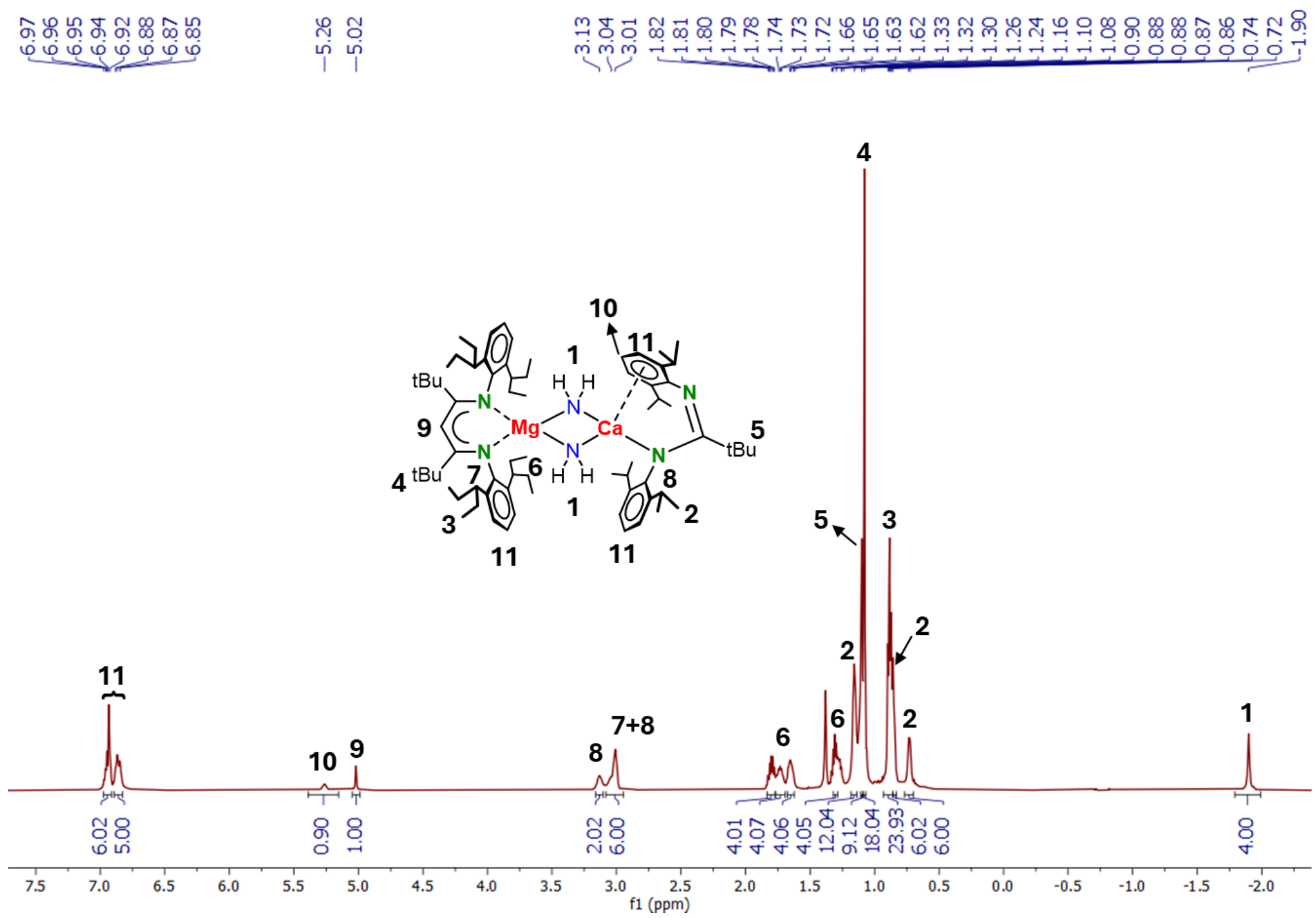

Fig. S44. ^{1}H NMR (600 MHz, 298 K, C_6D_{12}) spectrum of (BDI*)Mg-$(NH_2)_2$-Ca(Am*) (**7**-Ca).

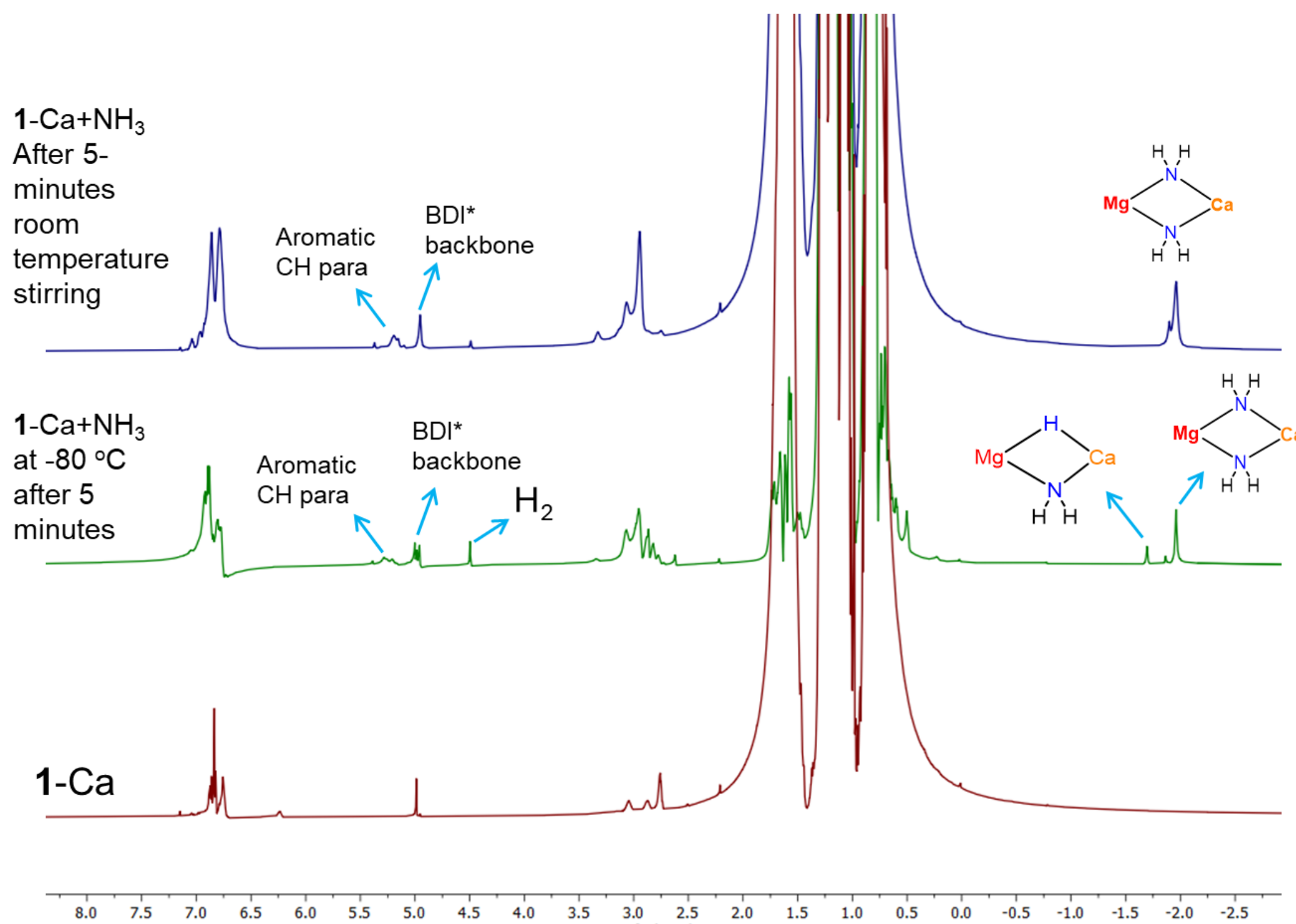

Fig. S45. No-D spectrum (600 MHz, 298 K, C_6H_{14}) of the reaction of (BDI*)Mg-Ca(Am*) (**1**-Ca) with NH_3(g). Top: ^{1}H NMR spectrum of reaction mixture after 5 minutes of stirring. Middle: ^{1}H NMR spectrum of reaction mixture kept at -80 °C for 5 minutes without stirring after the addition of NH_3, showing the proposed intermediate. Bottom: reference ^{1}H NMR spectrum of (BDI*)Mg-Ca(Am*) (**1**-Ca).

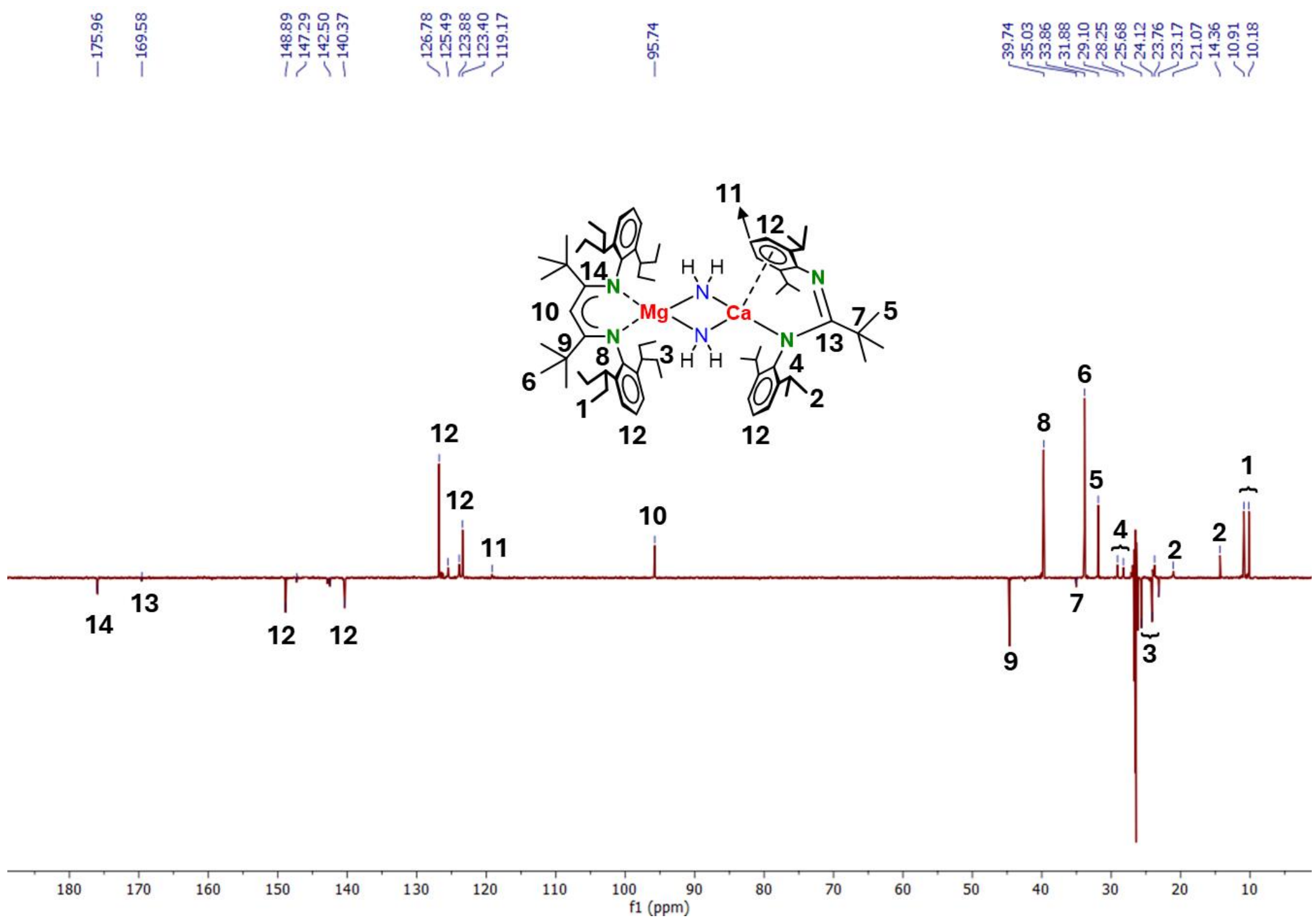

Fig. S46. ^{13}C-APT NMR (151 MHz, 298 K, C_6D_{12}) spectrum of (BDI*)Mg-$(NH_2)_2$-Ca(Am*) (**7**-Ca).

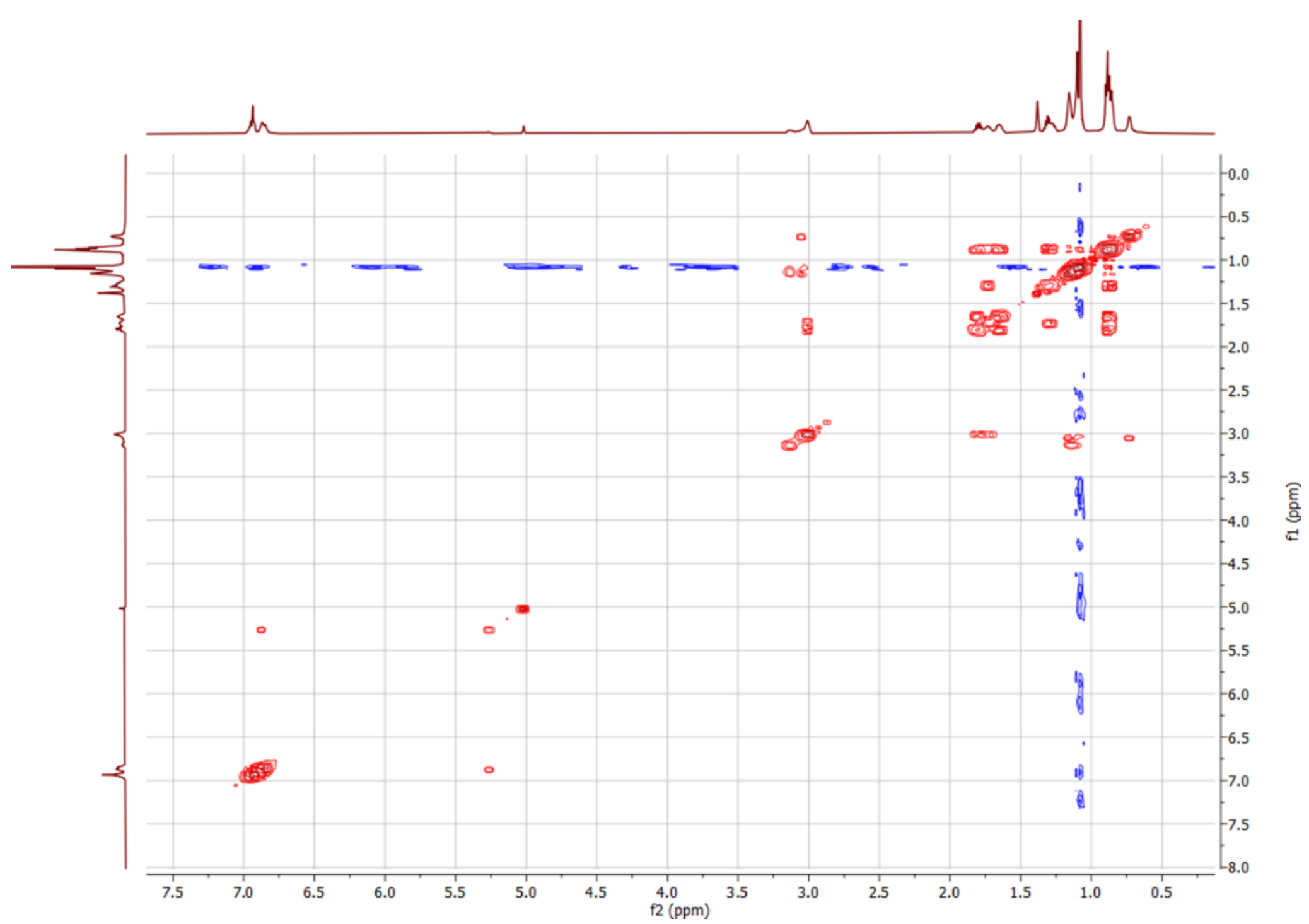

Fig. S47. 2D-COSY NMR (600 MHz, 298 K, C_6D_{12}) spectrum of (BDI*)Mg-$(NH_2)_2$-Ca(Am*) (**7**-Ca).

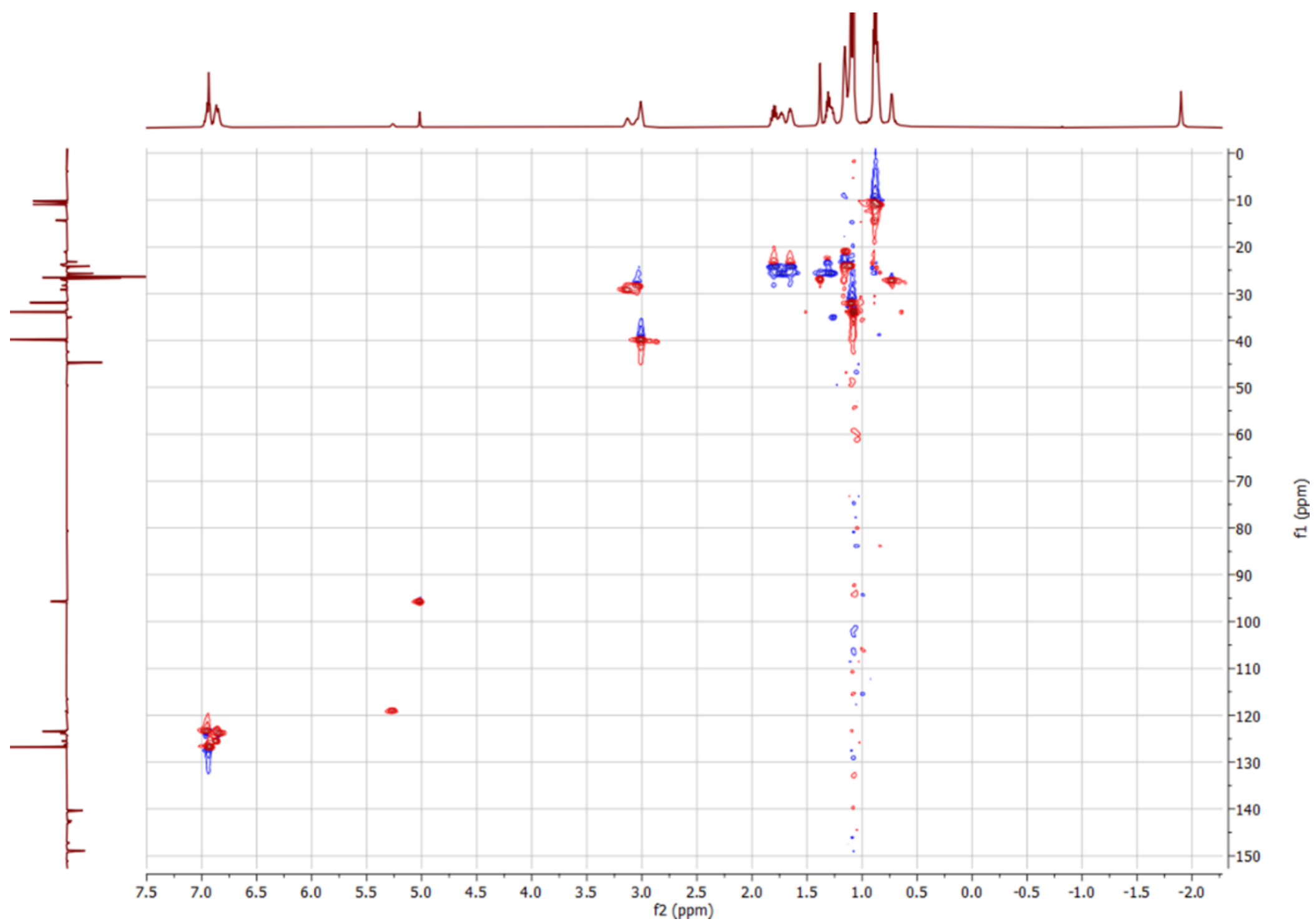

Fig. S48. 2D-HSQC NMR (600/151 MHz, 298 K, C_6D_{12}) spectrum of (BDI*)Mg-$(NH_2)_2$-Ca(Am*) (**7**-Ca).

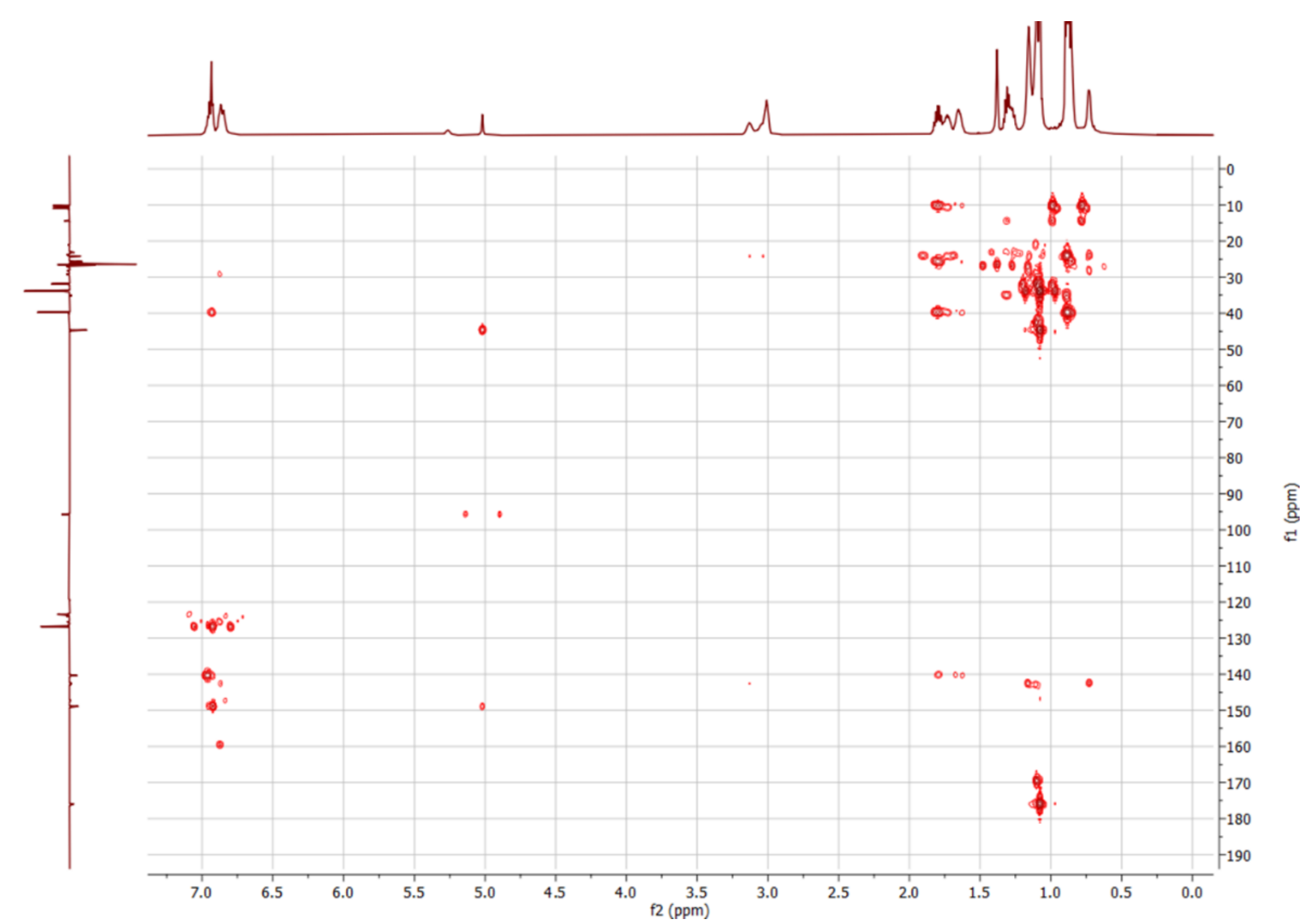

Fig. S49. 2D-HMBC NMR (600/151 MHz, 298 K, C_6D_{12}) spectrum of (BDI*)Mg-$(NH_2)_2$-Ca(Am*) (**7**-Ca).

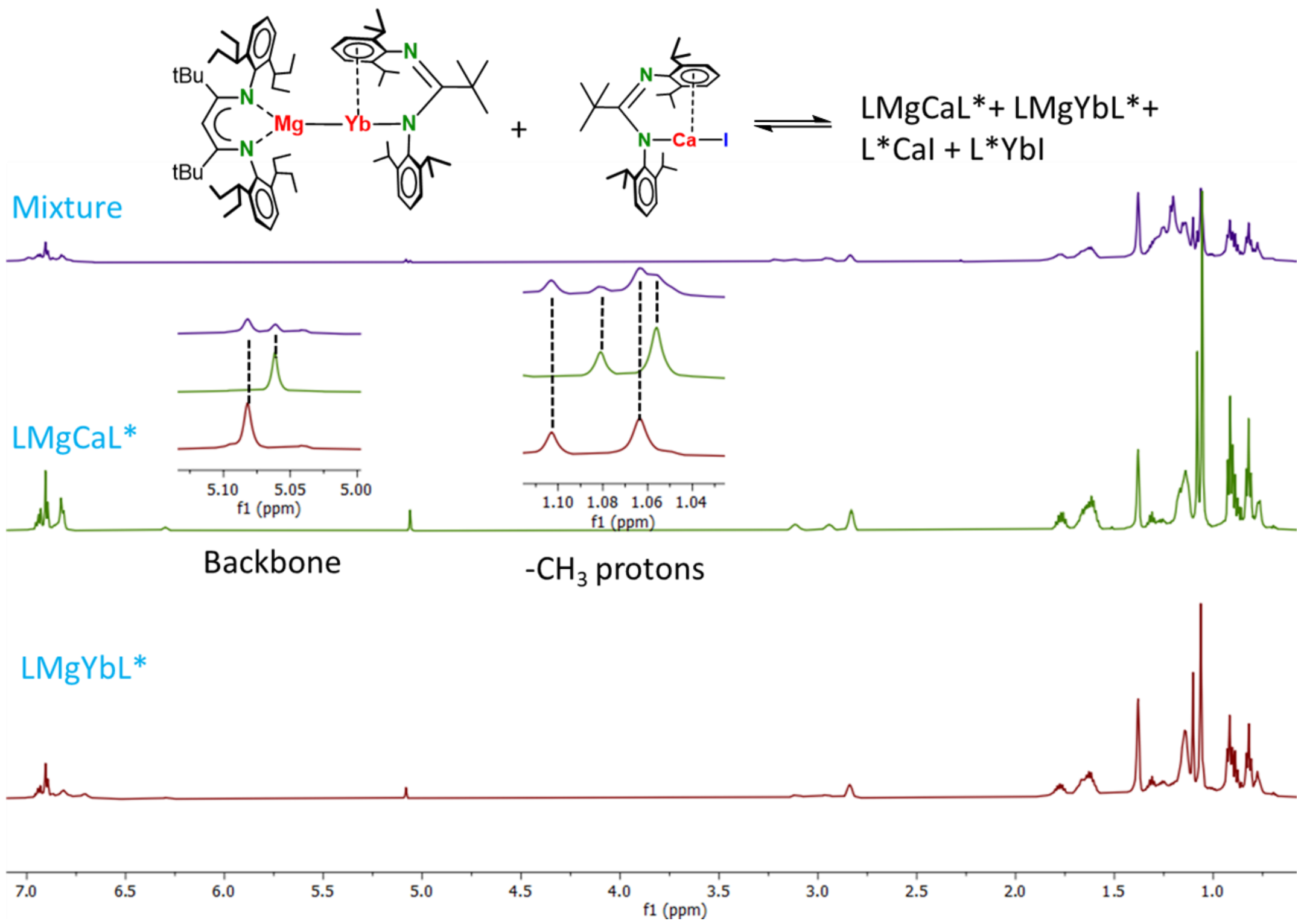

Fig. S50. ^{1}H NMR spectrum (600 MHz, 298 K, C_6D_{12}) of the reaction of 1 eq (BDI*)Mg-Yb(Am*) (**1**-Yb) with 0.5 eq $(Am^*CaI)_2$ (**2**-Ca). Top: ^{1}H NMR spectrum of reaction mixture. Middle: reference ^{1}H NMR spectrum of (BDI*)Mg-Ca(Am*) (**1**-Ca), bottom: reference ^{1}H NMR spectrum of (BDI*)Mg-Yb(Am*) (**1**-Yb).

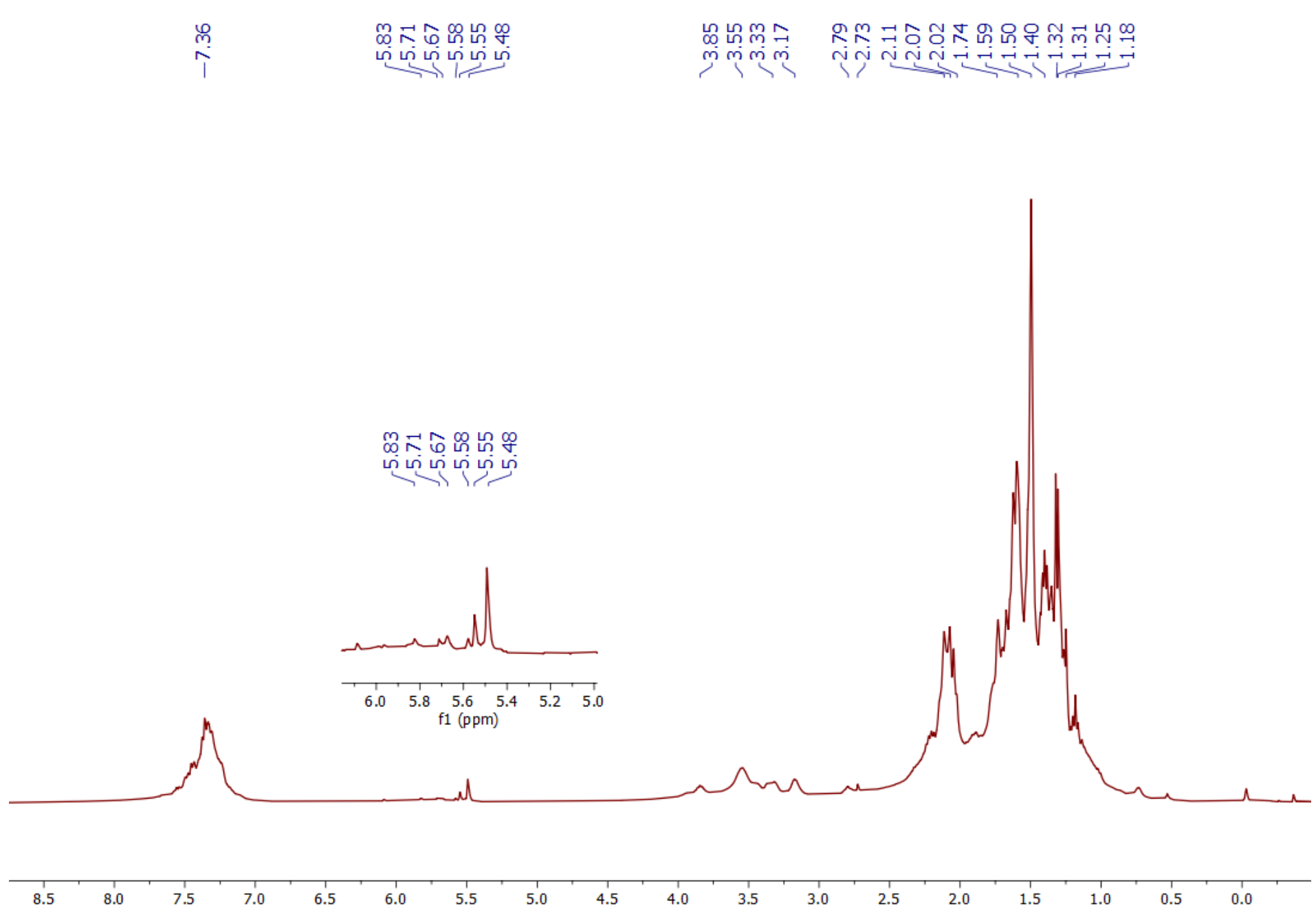

Fig. S51. ^{1}H NMR (600 MHz, 298 K, C_7D_{14}) spectrum of reaction of (BDI*)Mg-Ca(Am*) (**1**-Ca) with N_2O. At least 3 different unidentified products can be seen based on BDI* backbone proton.

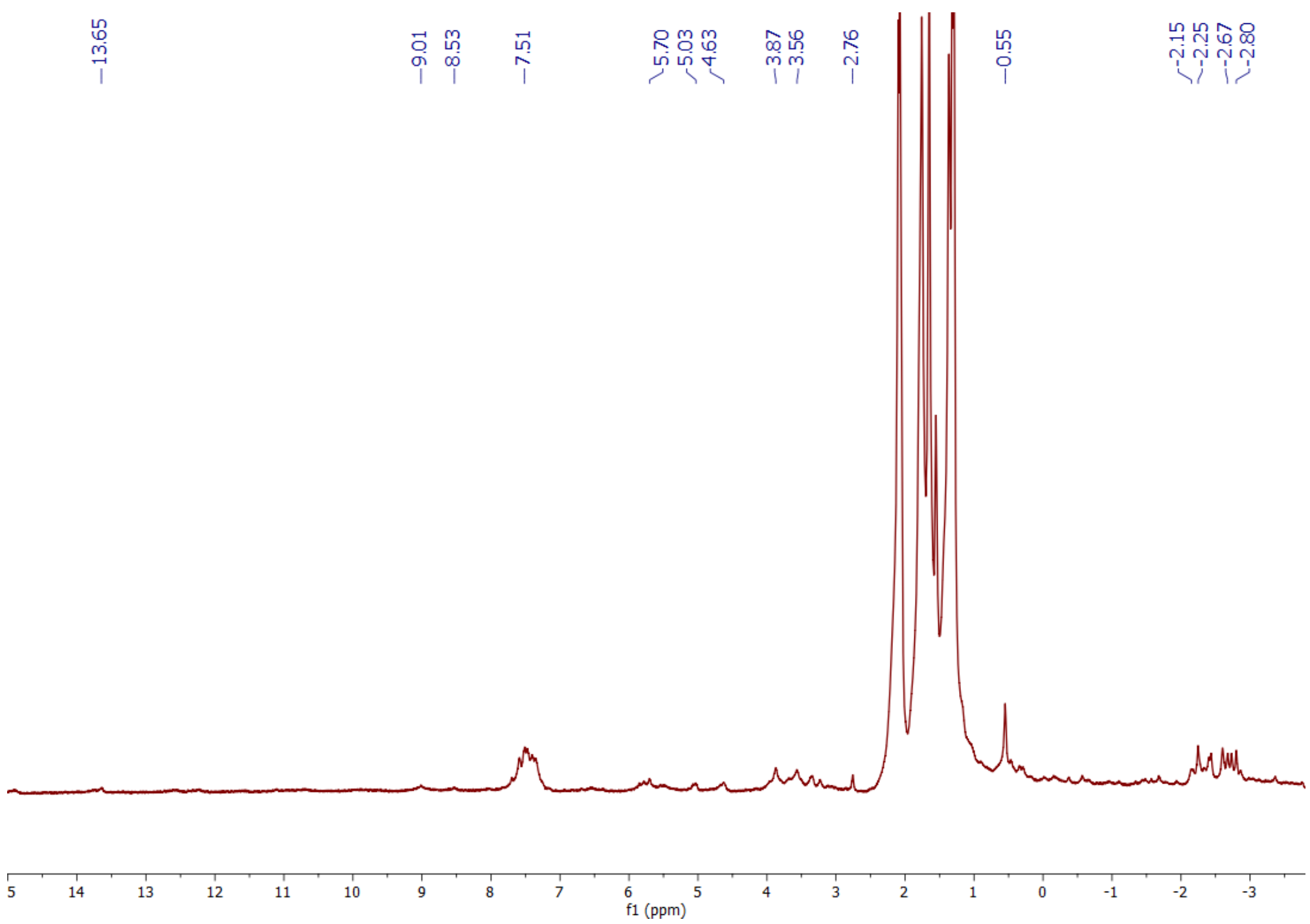

Fig. S52. ^{1}H NMR (600 MHz, 298 K, C_7D_{14}) spectrum of reaction of (BDI*)Mg-Yb(Am*) (**1**-Yb) with N_2O. The reaction is highly unselective and the wide range of broad signals indicate the formation of paramagnetic Yb^{III} species.

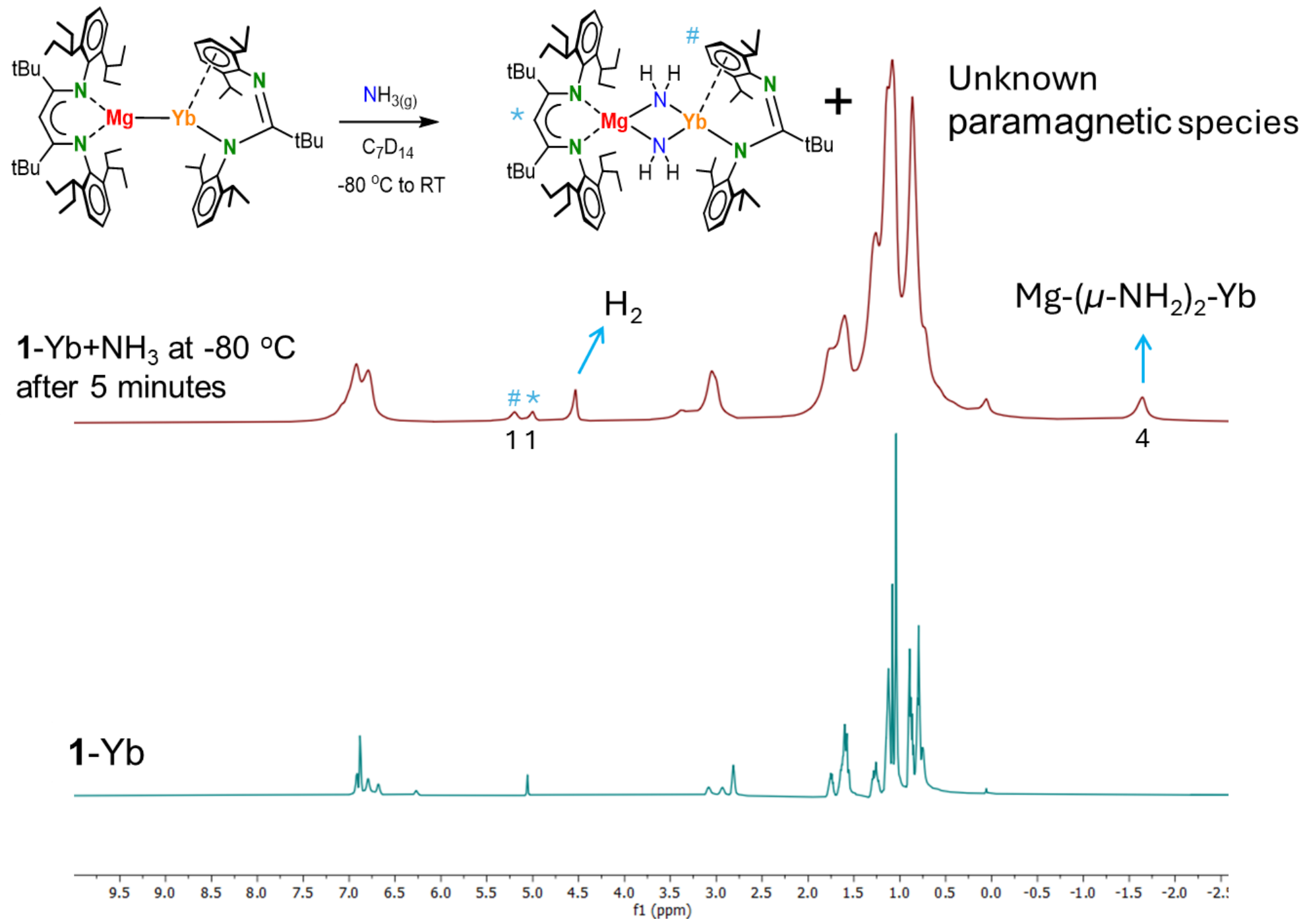

Fig. S53. ^{1}H NMR spectrum (600 MHz, 298 K, C_6D_{14}) of the reaction of (BDI*)Mg-Yb(Am*) (**1**-Yb) with NH_3(g). Top: ^{1}H NMR spectrum of the reaction mixture after the addition of NH_3 kept at -80 °C for 5 minutes without stirring. Bottom: reference ^{1}H NMR spectrum of (BDI*)Mg-Yb(Am*) (**1**-Yb).

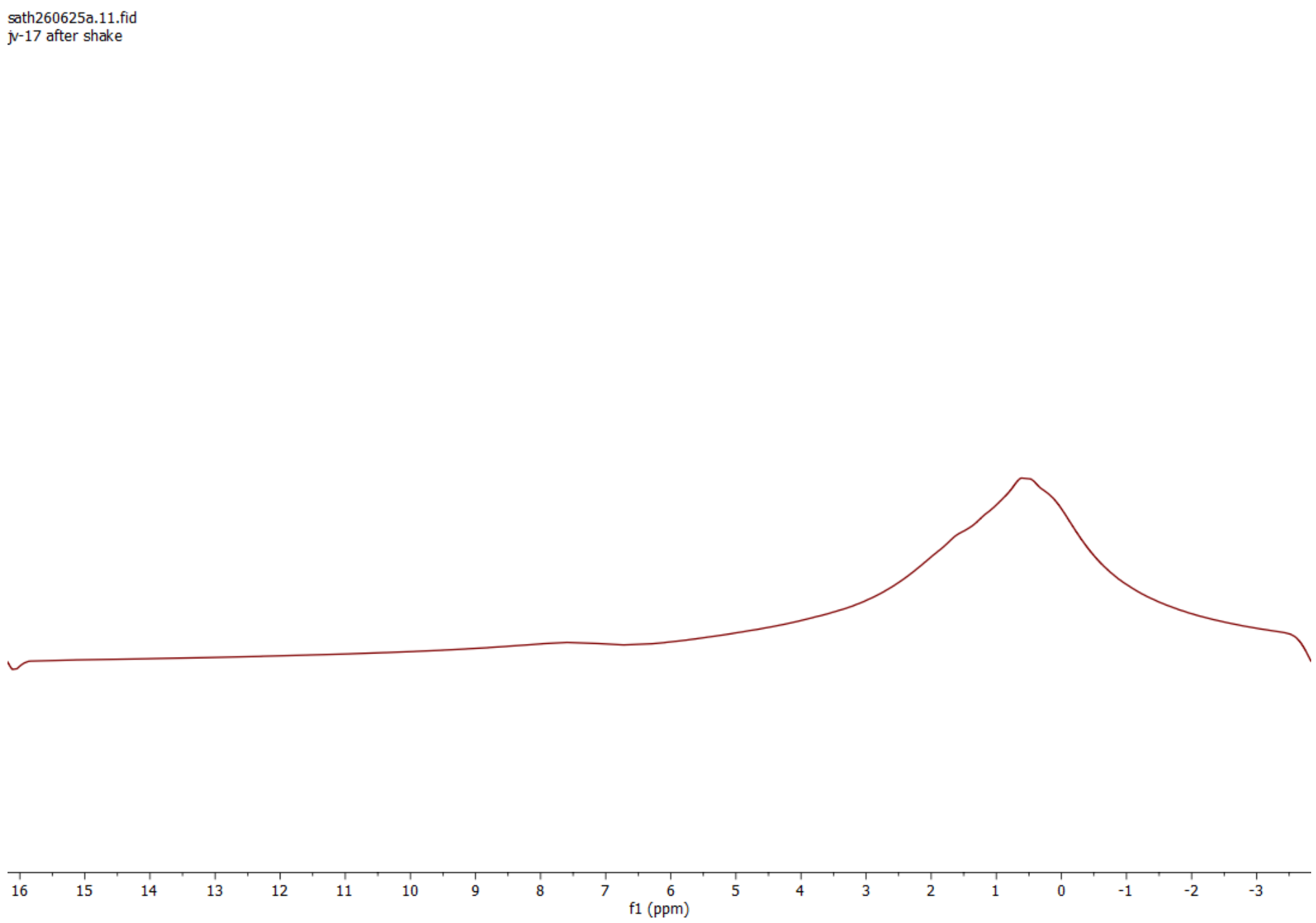

Fig. S54. ^{1}H NMR spectrum (600 MHz, 298 K, C_6D_{14}) of reaction (BDI*)Mg-Yb(Am*) (**1**-Yb) with NH_3(g) after 5 minutes of room temperature stirring.

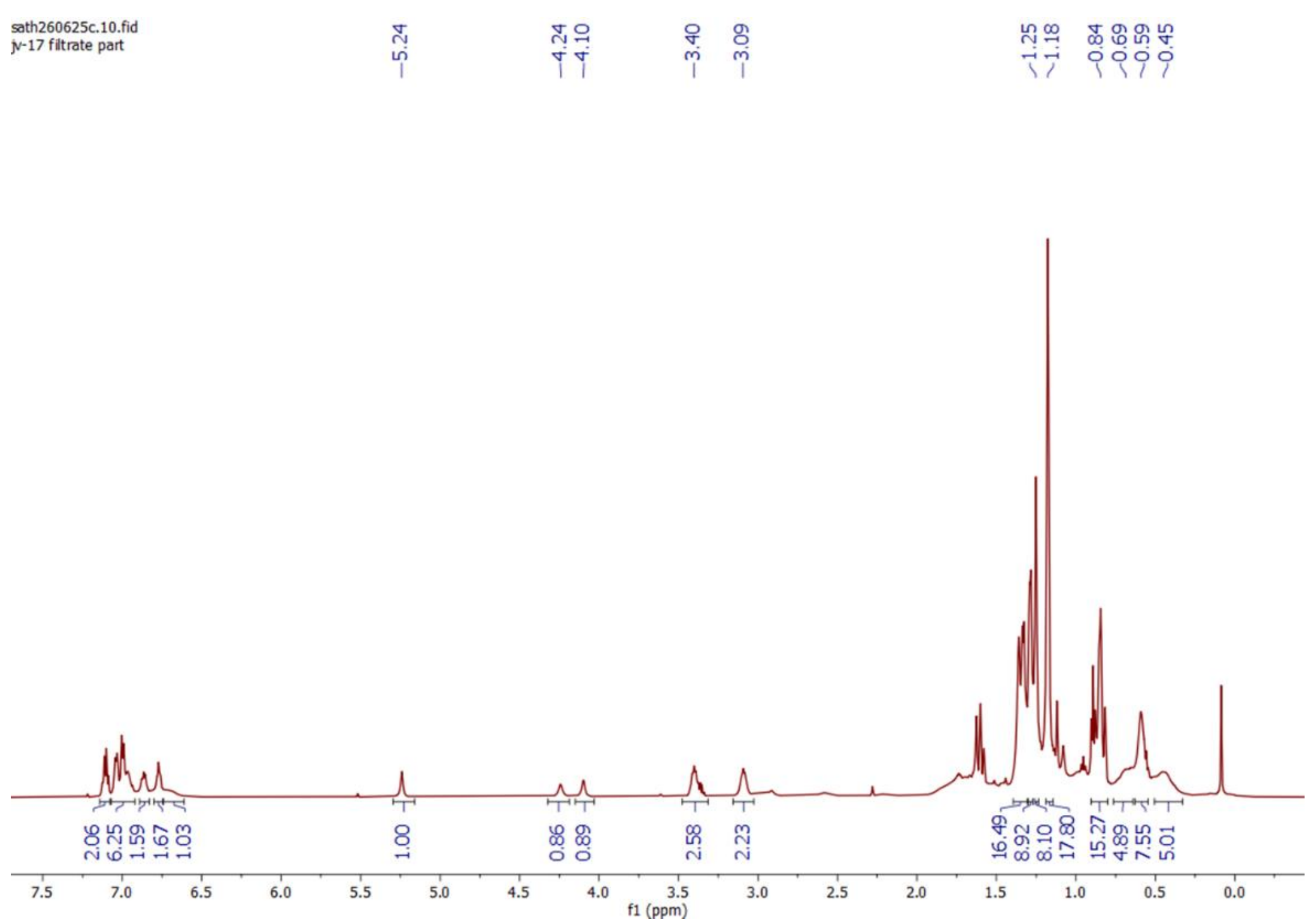

Fig. S55. ^{1}H NMR spectrum (600 MHz, 298 K, C_6D_{14}) of the filtrate part from the reaction of (BDI*)Mg-Yb(Am*) (**1**-Yb) with NH_3(g) after 5 minutes of room temperature stirring.

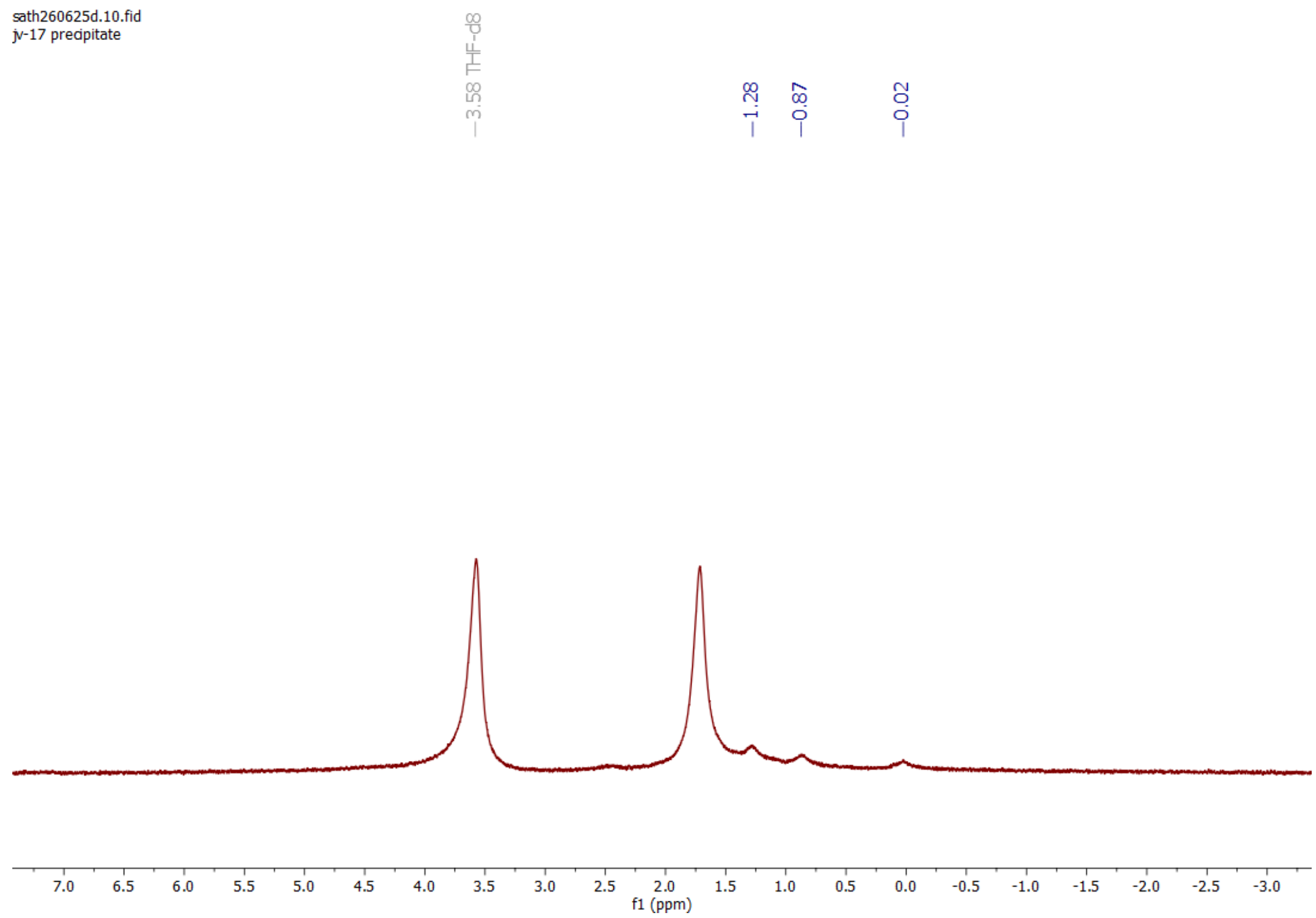

Fig. S56. ^{1}H NMR spectrum (600 MHz, 298 K, THF-d$_8$) of precipitate formed in the reaction of (BDI*)Mg-Yb(Am*) (**1**-Yb) with NH_3(g) after 5 minutes of room temperature stirring.

4. Crystal structure determination

Suitable single crystals of all the compounds were embedded in protective perfluoropolyalkylether oil (viscosity 1800 cSt; ABCR GmbH) on a microscope slide and a single specimen was selected and subsequently transferred to the cold nitrogen gas stream of the diffractometer.

The intensity data was collected at 100 K using MoK_α radiation (λ = 0.71073 Å; compound **2-Yb**) or CuK_α radiation (λ = 1.54184 Å, all other compounds) on an Agilent SuperNova dual radiation diffractometer (compounds **1-Yb**, **2-Yb** and **4-Yb**) or a Rigaku XtaLAB Synergy-S diffractometer (all other compounds) with microfocus X-ray sources and mirror optics. The measured data were processed with the CrysAlisPro software package.[6] Data was corrected for Lorentz and polarization effects, and an empirical absorption correction using spherical harmonics was applied. In all cases except of compounds **1-Yb**, **4-Yb** and **6-Ca**, an additional numerical absorption correction based on gaussian integration over a multifaceted crystal model was performed. For **1-Yb** and **6-Ca**, an additional analytical absorption correction[7] was performed instead. Using Olex2,[8] the structures were solved by dual-space methods (SHELXT)[9] and refined by full-matrix least-squares procedures on F^2 using SHELXL.[10] All non-hydrogen atoms were refined with

anisotropic displacement parameters. Most H-atoms were placed in geometrically calculated positions and refined by using a riding model where each H-atom was assigned a fixed isotropic displacement parameter with a value equal to 1.2U_{eq} (CH or CH_2) or 1.5U_{eq} (CH_3) of its parent C-atom.

The crystal of compound **1-Yb** was twinned by pseudo-merohedry (twin law 1, 0, 0 / 0, -1, 0 / 0, 0, -1). The contributions of the two twin domains were later on refined to 0.7448(12) and 0.2552(12). The asymmetric unit contained two molecules of the compound and two *n*-pentane molecules. Some disorder was observed that affected two 3-pentyl moieties and one of the co-crystallized *n*-pentanes. Similarity restraints (SADI, SIMU) and rigid bond restraints (RIGU)[11] were used to build a chemically reasonable model of the disorder. The relative occupancies of the two alternative orientations of the disordered moieties were refined to 0.51(7)/0.49(7) (3-pentyl 1), 0.63(9)/0.37(9) (3-pentyl 2) and 0.821(18)/0.179(18) (*n*-pentane), respectively.

Substantial disorder was also observed in case of **3-Ca**. Here, three 3-pentyl moieties, two *t*-Bu groups, one *i*-Pr group and the co-crystallized *n*-pentane were affected. The disorder was modeled with the help of similarity restraints (SADI, SIMU) and rigid bond restraints (RIGU).[11] The relative occupancies of the two alternative orientations of the disordered parts of the compound were refined to 0.529(5)/0.471(5) (3-pentyl 1), 0.745(4)/0.255(4) (3-pentyl 2), 0.836(6)/0.164(6) (3-pentyl 3), 0.63(3)/0.37(3) (*t*-Bu 1), 0.745(4)/0.255(4) (*t*-Bu 2), 0.713(9)/0.287(9) (*i*-Pr) and 0.922(5)/0.078(5) (*n*-pentane), respectively. The hydrogen atom of the bridging hydroxide was placed in the position indicated by a difference electron density map and this position was refined together with an isotropic displacement parameter.

The crystal of compound **4-Yb** suffered from non-merohedral twinning. The contributions of the two twin domains were later on refined to 0.6146(6) and 0.3854(6). The BDI*Mg moiety of the complex and a THF ligand were disordered over two alternative positions and were modeled with similarity restraints (SADI, SIMU) and rigid bond restraints (RIGU).[11] Site occupancy factors of 0.589(4)/0.411(4) (BDI*Mg) and 0.553(19)/0.447(19) (THF) were subsequently determined. The co-crystallized solvent was significantly disordered as well. Although *n*-pentane is likely the predominant occupant of the solvent cavities in the crystal (a mixture of *n*-pentane and THF was used for crystallization), the precise solvent content could not be determined. Therefore, the contribution of the co-crystallized solvent to the structure factors was secured by back-Fourier transformation using the solvent mask routine[12] of the program Olex2.[8] The solvent accessible voids treated this way had a size of 1171.9 $Å^3$ (12.6% of the unit cell) and contained 231.3 electrons.

The data of compound **5-Ca** showed disorder of the reduced ®-stilbene ligand and of one dipp moiety. Substitutional disorder of *n*-pentane and 3-methylpentane (co-crystallized solvents) was also observed. The use of similarity restraints (SADI, SIMU), rigid bond restraints (RIGU)[11] and geometric restraints (DFIX) during refinement ensured reasonable geometries within the disordered parts of the structure. Additionally, a rather tight ISOR restraint was used for C57A. Site occupancy factors of 0.861(3)/0.139(3) (stilbene), 0.741(10)/0.259(10) (DIPP) and 0.564(5)/0.436(5) (3-methylpentane/pentane) were determined for the disordered moieties. In contrast, **5-Yb** was well-ordered and only the co-crystallized *n*-pentane occupied two alternative positions. Refinement with some similarity restraints (SADI) and rigid bond restraints (RIGU)[11] went smoothly and led to site occupancy factors of 0.581(6) and 0.419(6) for the solvent.

The co-crystallized *n*-pentane is also partially disordered in crystals of **6-Ca**. While there was one well-ordered solvent molecule, others moieties occupied an infinite solvent channel and were heavily disordered. Their contribution to the structure factors was secured by back-Fourier transformation using the solvent mask routine[12] of the program Olex2.[8] The solvent accessible voids treated this way had a size of 976.8 Å^3 (11.3% of the unit cell) and contained 126.8 electrons. Since *n*-pentane was the only solvent used, a content of approximately 0.75 *n*-pentane per asymmetric unit (126 electrons per unit cell) in these solvent channels was calculated. Additionally, the crystal of **6-Ca** was a non-merohedral twin and was treated accordingly. The fractional contributions of the two twin domains were 0.6463(6) and 0.3537(6). The hydrogen atoms H73, H75 – H78 of the anthracene ligand were placed in the positions indicated by a difference electron density map and these positions were refined together with isotropic displacement parameters.

The data of the crystal of **6-Yb**, which was isostructural to **6-Ca**, was treated similarly. Hydrogen atoms placement and refinement was done as described for **6-Ca**. However, the crystal of **6-Yb** was not twinned and the solvent channels contained a slightly higher amount of *n*-pentane The solvent accessible voids had a size of 961.5 Å^3 (11.1% of the unit cell) and contained 158.6 electrons, which corresponded better to 1 *n*-pentane per asymmetric unit (168 electrons per unit cell).

In case of compound **7-Ca**, only the co-crystallized *n*-pentane was disordered. This solvent moiety was modeled with some similarity restraints (SADI, SIMU) and gave site occupancy factors of 0.856(4) and 0.144(4). The hydrogen atoms of the amido ligands were placed in the positions indicated by a difference electron density map and these positions were refined together with isotropic displacement parameter.

Table S1. Crystal data and structure refinement for compounds **1**-Yb, **2**-Yb and **3**-Ca.

Compound	Compound **1-Yb**·*n*-pentane	Compound **2-Yb**	Compound **3-Ca**·*n*-pentane
Identification code	hasj250311a	hasj250415a	hasj260319a
Empirical formula	$C_{77}H_{124}N_4MgYb$	$C_{58}H_{86}N_4I_2Yb_2$	$C_{77}H_{124}CaMgN_4O$
Formula weight	1303.14	1439.18	1186.18
Temperature/K	100.01(10)	100.00(10)	100.00(10)
Crystal system	triclinic	monoclinic	triclinic
Space group	P-1	$P2_1/n$	P-1
a/Å	12.1981(3)	23.2609(6)	14.9565(2)
b/Å	24.3626(4)	10.4547(3)	15.2824(2)
c/Å	24.9680(2)	24.2257(7)	19.2339(2)
α/°	86.4690(10)	90	104.9600(10)
β/°	89.8670(10)	97.084(3)	95.4720(10)
γ/°	89.660(2)	90	117.831(2)
Volume/Å^3	7405.7(2)	5846.4(3)	3634.13(10)
Z	4	4	2
ρ_{calc}/g/cm^3	1.169	1.635	1.084
μ/mm^{-1}	2.717	4.273	1.151
F(000)	2784.0	2832.0	1308.0
Crystal size/mm^3	0.309 × 0.149 × 0.037	0.21 × 0.11 × 0.06	0.259 × 0.134 × 0.072
Radiation	Cu Kα (λ = 1.54184)	Mo Kα (λ = 0.71073)	Cu Kα (λ = 1.54184)
2θ range for data collection/°	7.094 to 143.966	4.52 to 58.9	4.916 to 156.684
Index ranges	-13 ≤ h ≤ 15, -29 ≤ k ≤ 29, -30 ≤ l ≤ 30	-31 ≤ h ≤ 31, -13 ≤ k ≤ 14, -33 ≤ l ≤ 33	-14 ≤ h ≤ 18, -19 ≤ k ≤ 19, -24 ≤ l ≤ 23
Reflections collected	108613	54392	55478
Independent reflections	28482 [R_{int} = 0.0499, R_{sigma} = 0.0447]	14438 [R_{int} = 0.0363, R_{sigma} = 0.0406]	14943 [R_{int} = 0.0361, R_{sigma} = 0.0335]
Data/restraints/parameters	28482/501/1656	14438/0/617	14943/1674/1068
Goodness-of-fit on F^2	1.092	1.038	1.037
Final R indexes [I>=2σ (I)]	R_1 = 0.0524, wR_2 = 0.1319	R_1 = 0.0279, wR_2 = 0.0467	R_1 = 0.0671, wR_2 = 0.1873
Final R indexes [all data]	R_1 = 0.0599, wR_2 = 0.1401	R_1 = 0.0399, wR_2 = 0.0502	R_1 = 0.0742, wR_2 = 0.1940
Largest diff. peak/hole / e Å^{-3}	1.69/-3.08	0.92/-0.72	0.66/-0.41
CCDC number	2566191	2566192	2566193

Table S2. Crystal data and structure refinement for compounds **4**-Yb, **5**-Ca and **5**-Yb.

Compound	Compound **4-Yb**·[solvent]	Compound **5-Ca**·(3-methylpentane /*n*-pentane)	Compound **5-Yb**·*n*-pentane
Identification code	hasj250630b_twin1_hklf5	hasj260209a	hasj260127b
Empirical formula	$C_{94}H_{140}MgN_4O_2Yb$ a)	$C_{91.56}H_{137.13}CaMgN_4$	$C_{91}H_{136}MgN_4Yb$
Formula weight	1555.44 a)	1358.34	1483.38
Temperature/K	100.00(10)	100.00(10)	100.00(10)
Crystal system	monoclinic	triclinic	triclinic
Space group	$P2_1/c$	P-1	P-1
a/Å	18.0882(4)	11.1710(2)	11.13576(11)
b/Å	11.3767(2)	18.9830(2)	18.93311(14)
c/Å	45.3491(9)	19.6422(2)	19.62902(13)
α/°	90	90.3190(10)	91.0004(6)
β/°	93.636(2)	96.0550(10)	95.6059(7)
γ/°	90	93.6460(10)	93.2342(7)
Volume/Å^3	9313.3(3)	4133.37(10)	4111.02(6)
Z	4	2	2
ρ_{calc}/g/cm^3	1.109 a)	1.091	1.198
μ/mm^{-1}	2.253 a)	1.061	2.509
F(000)	3320.0 a)	1493.0	1584.0
Crystal size/mm^3	0.433 × 0.316 × 0.041	0.273 × 0.116 × 0.007	0.115 × 0.076 × 0.014
Radiation	Cu Kα (λ = 1.54184)	Cu Kα (λ = 1.54184)	Cu Kα (λ = 1.54184)
2θ range for data collection/°	7.814 to 143.536	6.46 to 157.164	6.432 to 157.178
Index ranges	-22 ≤ h ≤ 22, -14 ≤ k ≤ 14, -53 ≤ l ≤ 55	-14 ≤ h ≤ 13, -20 ≤ k ≤ 24, -23 ≤ l ≤ 24	-14 ≤ h ≤ 14, -23 ≤ k ≤ 24, -24 ≤ l ≤ 24
Reflections collected	30561	59750	79454
Independent reflections	30561 [R_{int} = 0.0639, R_{sigma} = 0.0331]	17117 [R_{int} = 0.0396, R_{sigma} = 0.0384]	17184 [R_{int} = 0.0458, R_{sigma} = 0.0356]
Data/restraints/parameters	30561/6985/1402	17117/2657/1199	17184/49/949
Goodness-of-fit on F^2	1.046	1.055	1.072
Final R indexes [I>=2σ (I)]	R_1 = 0.0447, wR_2 = 0.1242	R_1 = 0.0474, wR_2 = 0.1234	R_1 = 0.0313, wR_2 = 0.0786
Final R indexes [all data]	R_1 = 0.0550, wR_2 = 0.1317	R_1 = 0.0588, wR_2 = 0.1301	R_1 = 0.0353, wR_2 = 0.0804
Largest diff. peak/hole / e Å^{-3}	0.80/-0.87	0.38/-0.39	0.65/-1.13
CCDC number	2566194	2566195	2566196

a) contribution of masked disordered solvent neglected.

Table S3. Crystal data and structure refinement for compounds **6**-Ca, **6**-Yb and **7**-Ca.

Compound	Compound **6-Ca**·*n*-pentane ·0.75[*n*-pentane]	Compound **6-Yb**·*n*-pentane ·[*n*-pentane]	Compound **7-Ca**·*n*-pentane
Identification code	hasj260202a_twin1_hklf5	hasj260120a	hasj260112a
Empirical formula	$C_{94.75}H_{143}CaMgN_4$	$C_{96}H_{146}MgN_4Yb$	$C_{77}H_{128}CaMgN_6$
Formula weight	1402.51	1553.51	1202.24
Temperature/K	100.01(10)	100.01(10)	99.99(10)
Crystal system	monoclinic	monoclinic	monoclinic
Space group	$P2_1/n$	$P2_1/n$	$P2_1/c$
a/Å	19.0084(4)	19.0316(2)	23.8376(3)
b/Å	11.0342(2)	11.00810(10)	12.19094(15)
c/Å	41.6405(7)	41.5380(4)	25.7290(3)
α/°	90	90	90
β/°	97.5269(17)	97.5730(10)	95.4471(10)
γ/°	90	90	90
Volume/Å^3	8658.6(3)	8626.38(15)	7443.15(15)
Z	4	4	4
ρ_{calc}/g/cm^3	1.076	1.196	1.073
μ/mm^{-1}	1.026	2.413	1.126
F(000)	3086.0	3328.0	2656.0
Crystal size/mm^3	0.209 × 0.066 × 0.045	0.135 × 0.12 × 0.014	0.314 × 0.104 × 0.085
Radiation	Cu Kα (λ = 1.54184)	Cu Kα (λ = 1.54184)	Cu Kα (λ = 1.54184)
2θ range for data collection/°	5.404 to 158.22	5.404 to 157.838	6.902 to 154.912
Index ranges	-24 ≤ h ≤ 24, -13 ≤ k ≤ 13, -52 ≤ l ≤ 52	-19 ≤ h ≤ 24, -13 ≤ k ≤ 12, -51 ≤ l ≤ 49	-28 ≤ h ≤ 30, -14 ≤ k ≤ 15, -32 ≤ l ≤ 29
Reflections collected	37948	83262	85206
Independent reflections	37948 [R_{int} = 0.0665, R_{sigma} = 0.0254]	17982 [R_{int} = 0.0514, R_{sigma} = 0.0397]	15536 [R_{int} = 0.0393, R_{sigma} = 0.0290]
Data/restraints/parameters	37948/0/922	17982/0/921	15536/268/857
Goodness-of-fit on F^2	1.052	1.059	1.064
Final R indexes [I>=2σ (I)]	R_1 = 0.0500, wR_2 = 0.1407	R_1 = 0.0401, wR_2 = 0.0834	R_1 = 0.0391, wR_2 = 0.0986
Final R indexes [all data]	R_1 = 0.0645, wR_2 = 0.1499	R_1 = 0.0508, wR_2 = 0.0863	R_1 = 0.0437, wR_2 = 0.1013
Largest diff. peak/hole / e Å^{-3}	0.26/-0.44	0.90/-0.86	0.30/-0.42
CCDC number	2566197	2566198	2566199

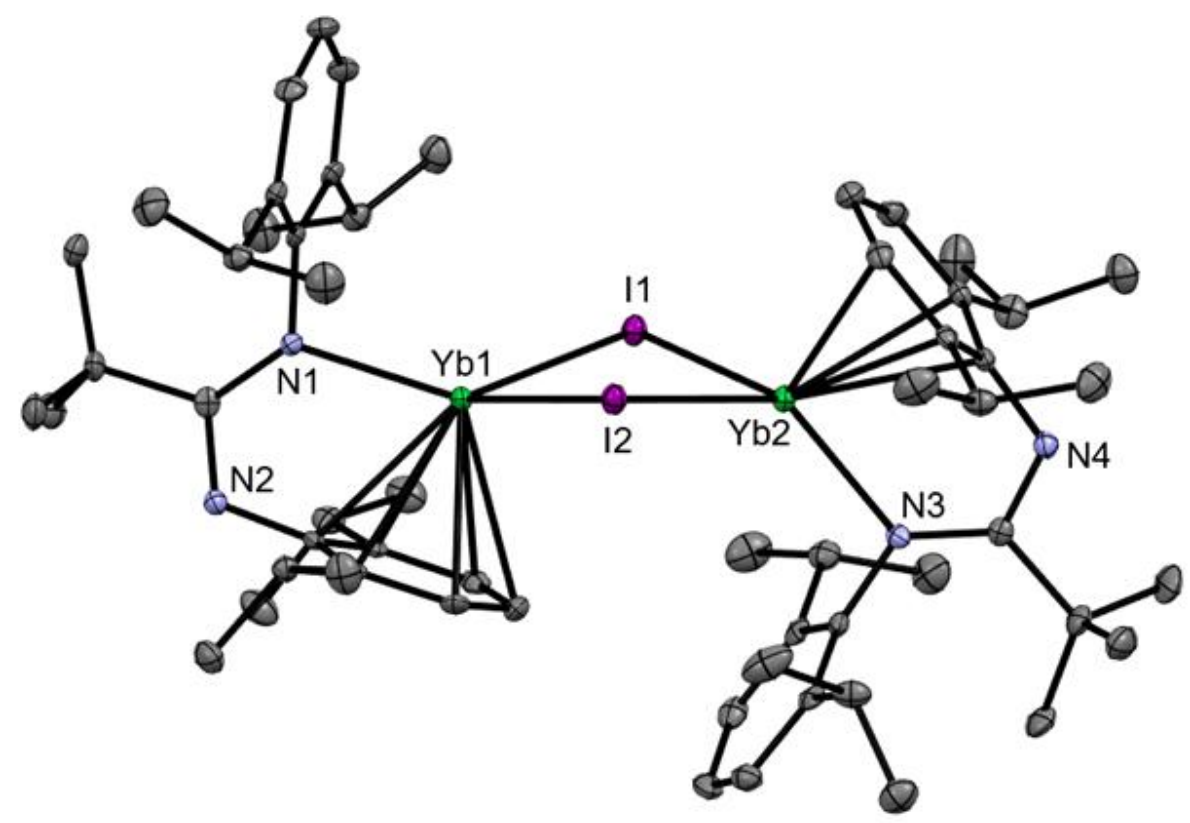

Fig. S57. ORTEP representation of [Am*YbI]$_2$ (**2**-Yb). with atomic displacement ellipsoids set at 50% probability. Hydrogen atoms are omitted for clarity.

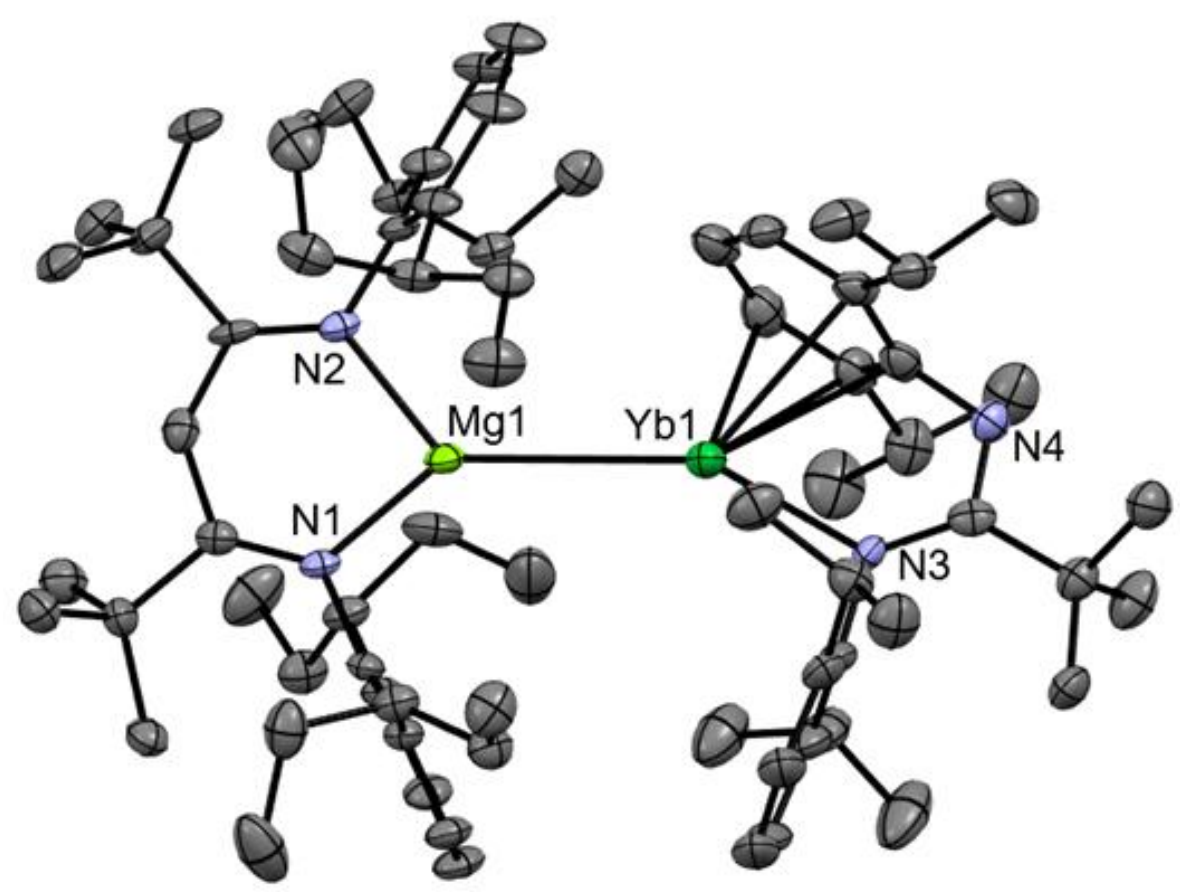

Fig. S58. ORTEP representation of (BDI*)Mg-Yb(Am*) (**1**-Yb) with atomic displacement ellipsoids set at 50% probability. Hydrogen atoms and solvent molecule are omitted for clarity.

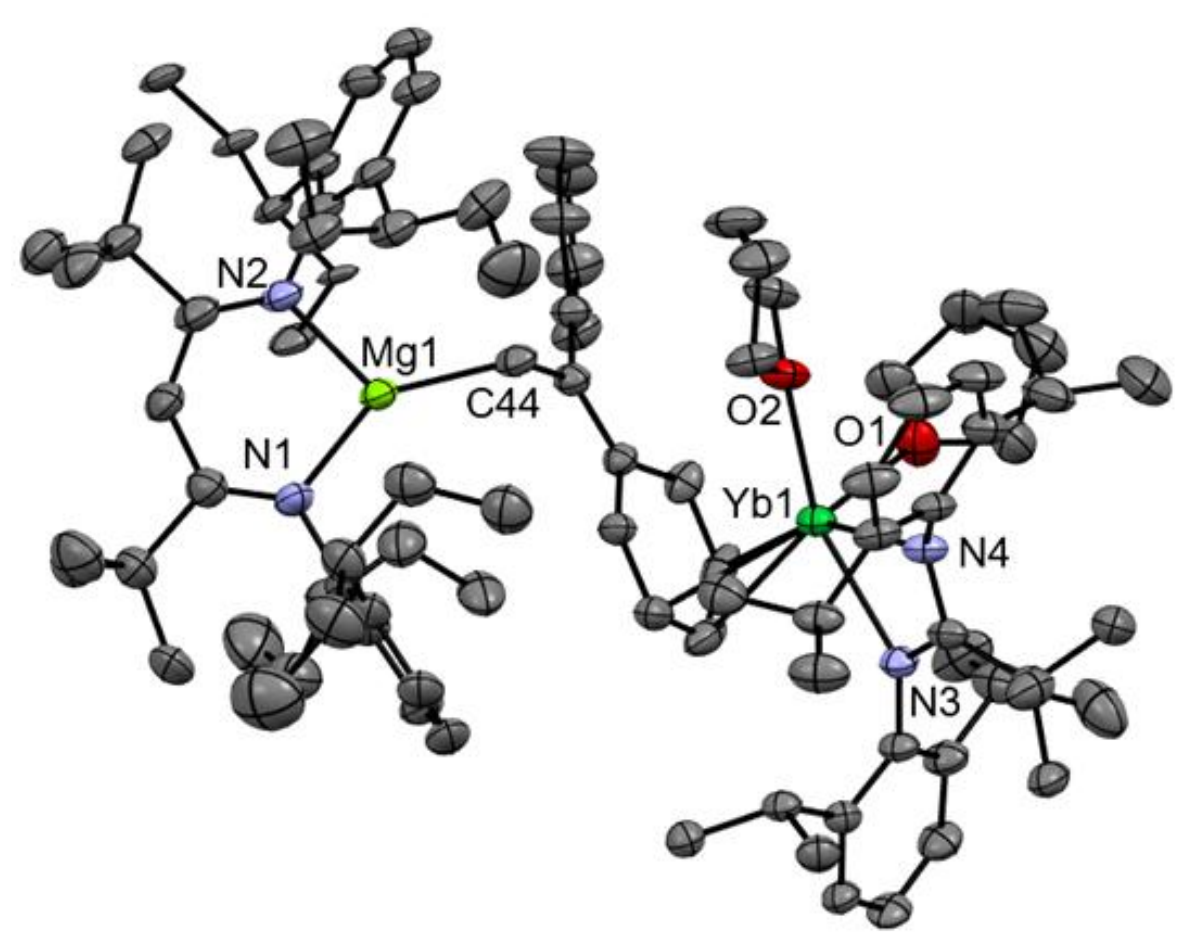

Fig. S59. ORTEP representation of (BDI*)Mg-[H_2CCPh_2]-Yb(Am*) (**4**-Yb) with atomic displacement ellipsoids set at 50% probability. Hydrogen atoms and solvent molecule are omitted for clarity.

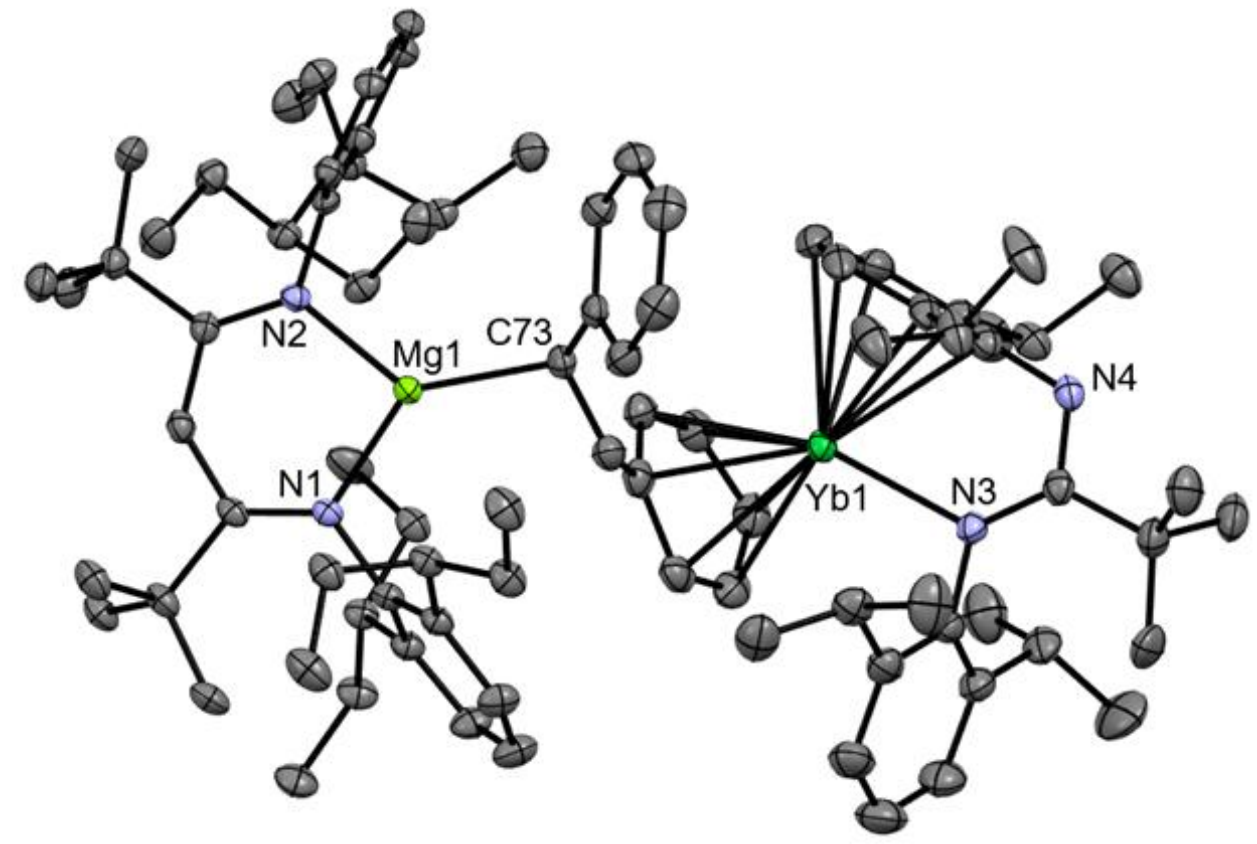

Fig. S60. ORTEP representation of (BDI*)Mg-CH(Ph)(H)C(Ph)-Yb(Am*) (**5**-Yb) with atomic displacement ellipsoids set at 50% probability. Hydrogen atoms and solvent molecule are omitted for clarity.

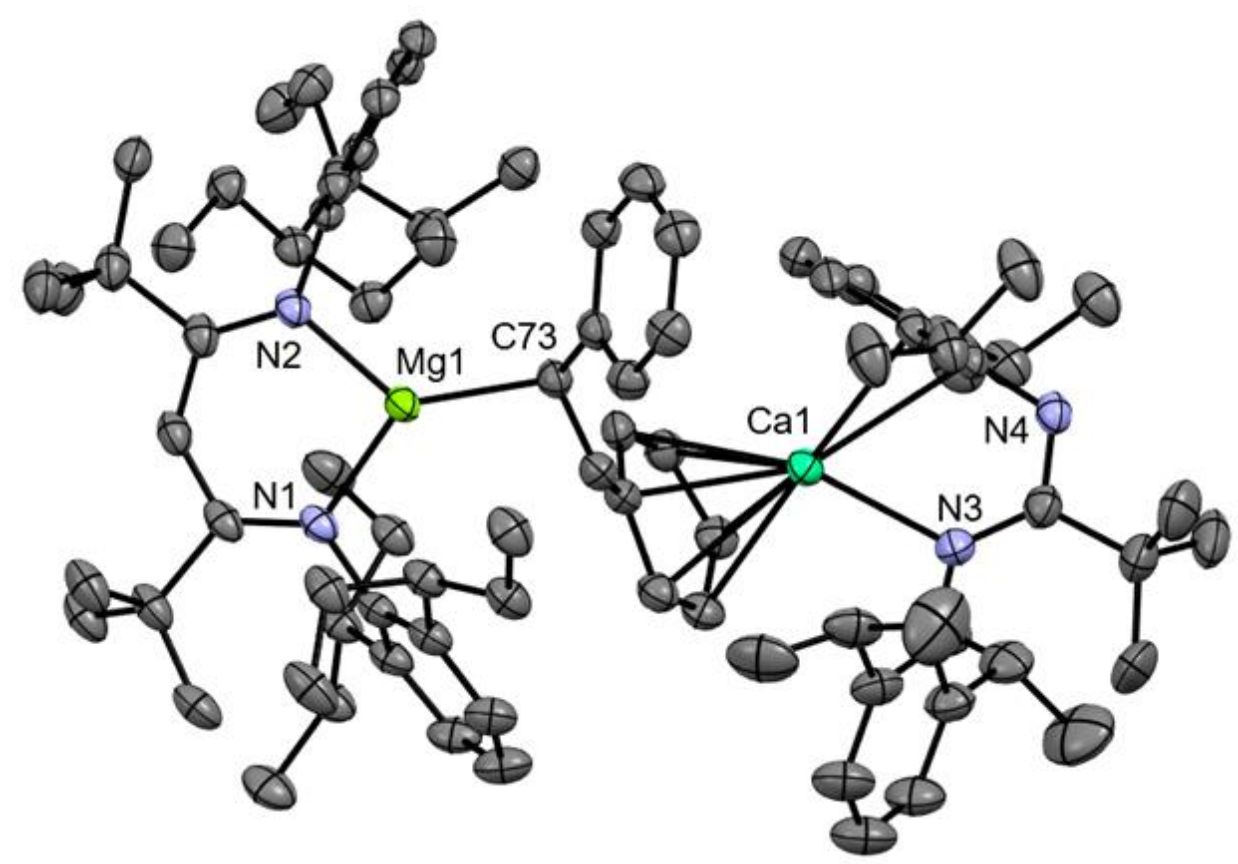

Fig. S61. ORTEP representation of (BDI*)Mg-CH(Ph)(H)C(Ph)-Ca(Am*) (**5**-Ca) with atomic displacement ellipsoids set at 50% probability. Hydrogen atoms and solvent molecule are omitted for clarity.

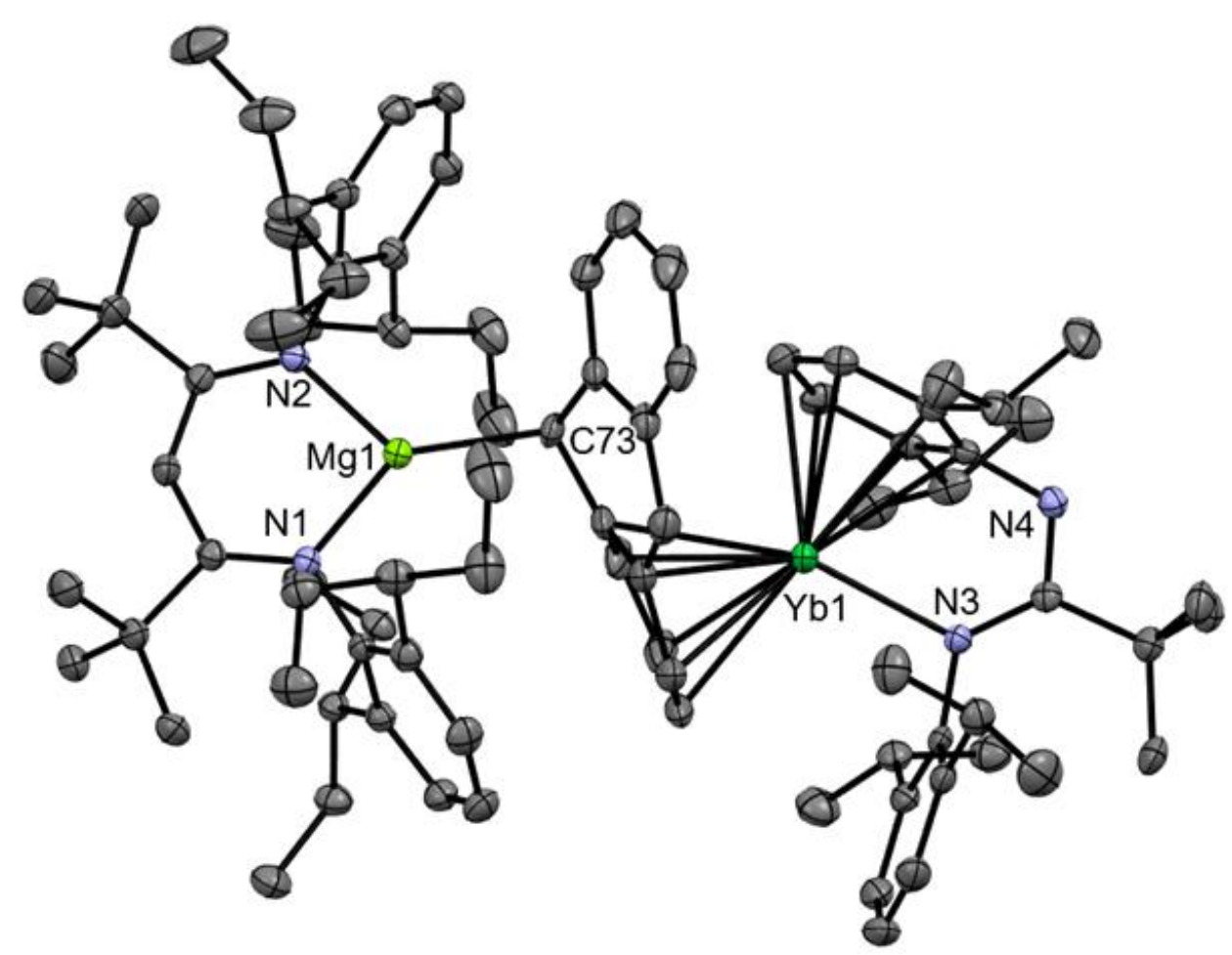

Fig. S62. ORTEP representation of (BDI*)Mg-anthracene-Yb(Am*) (**6**-Yb) with atomic displacement ellipsoids set at 50% probability. Hydrogen atoms and solvent molecule are omitted for clarity.

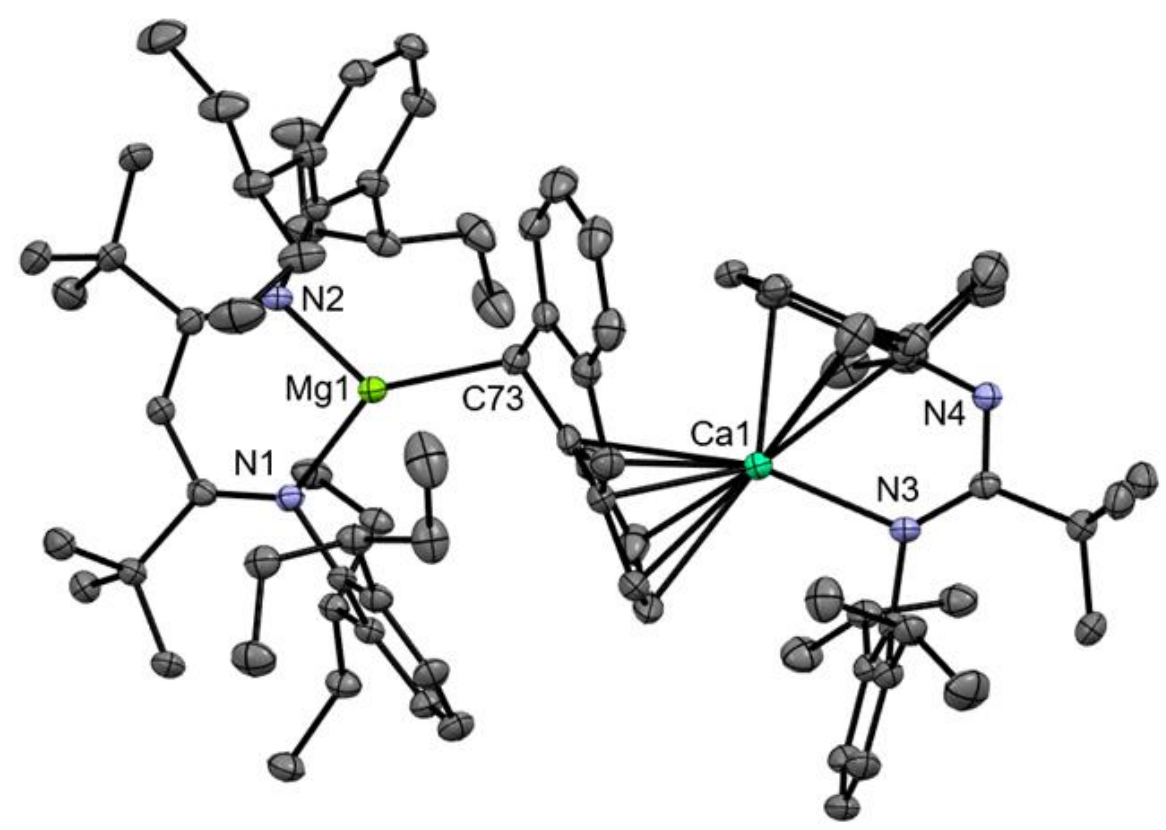

Fig. S63. ORTEP representation of (BDI*)Mg-anthracene-Ca(Am*) (**6**-Ca) with atomic displacement ellipsoids set at 50% probability. Hydrogen atoms and solvent molecule are omitted for clarity.

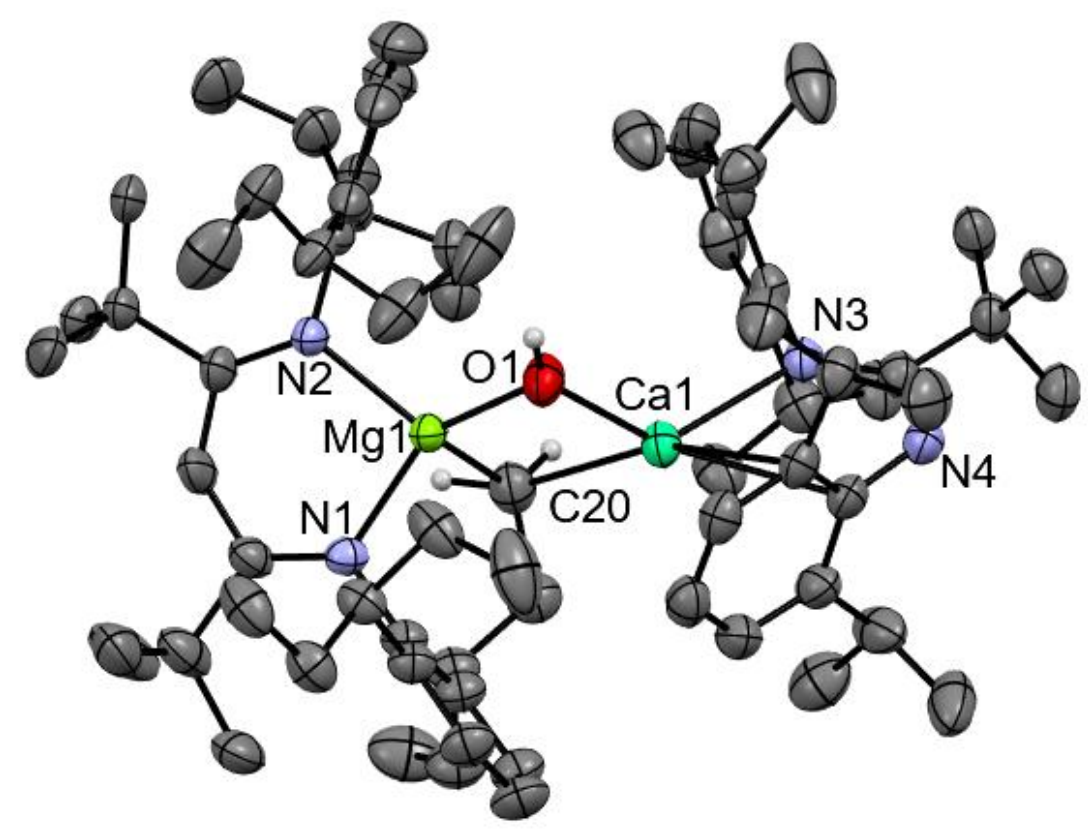

Fig. S64. ORTEP representation of **3**-Ca with atomic displacement ellipsoids set at 50% probability. Hydrogen atoms are omitted for clarity except for OH and C20 atoms.

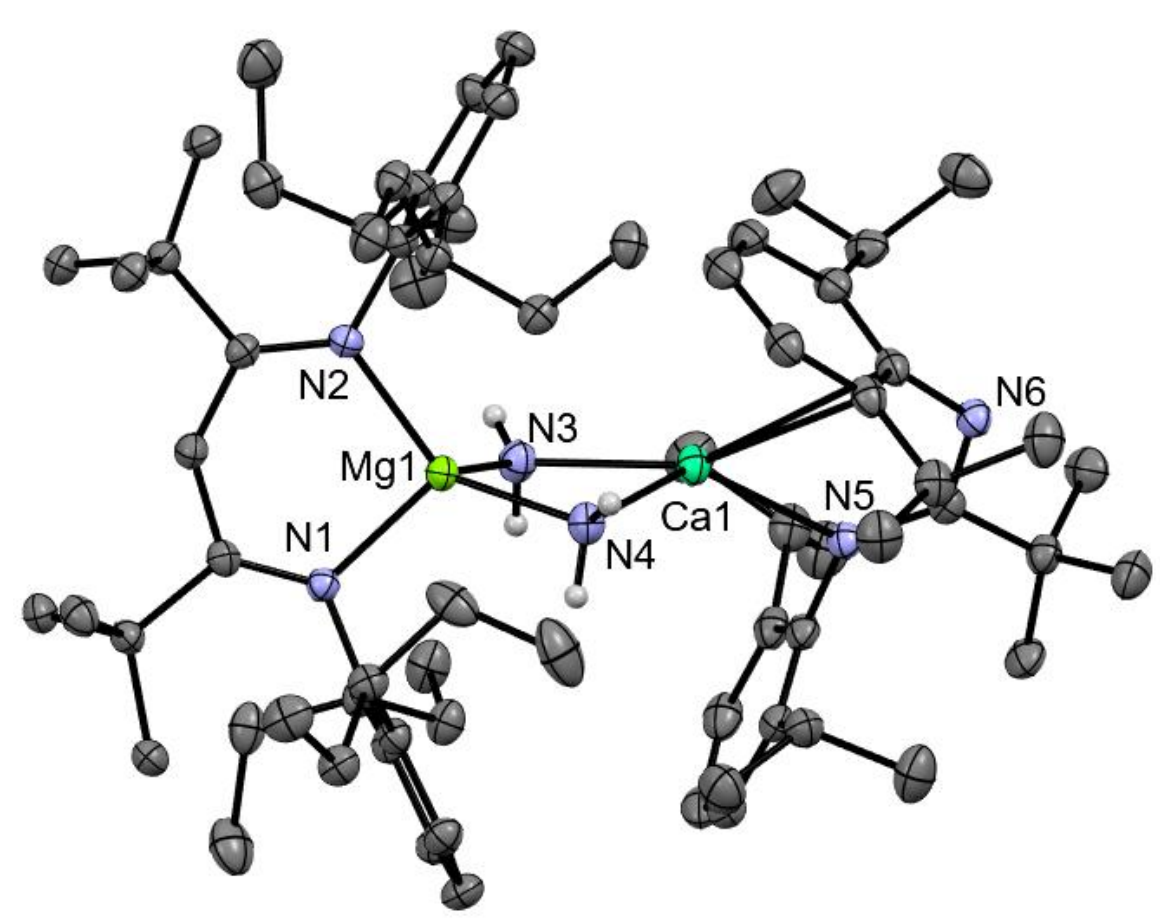

Fig. S65. ORTEP representation of (BDI*)Mg-$(NH_2)_2$-Ca(Am*) (**7**-Ca) with atomic displacement ellipsoids set at 50% probability. Hydrogen atoms except for NH hydrogens and solvent molecule are omitted for clarity.

5. DFT Calculations

General considerations

All geometry optimizations and analytical vibrational frequency calculations were carried out using the Gaussian 16 software package (revision A.03).[13] Geometries we optimized using the corresponding X-ray structures as starting points. The B3PW91 hybrid functional[14] was used together with Ahlrich's def2SVP basis set[15] for all atoms. Dispersion was considered using Grimme's D3 long-range dispersion corrections[16] together with the Becke-Johnson damping function.[17] The wavefunctions used for the QTAIM and EOS analysis (*vide infra*) were obtained with single points calculations of the geometries obtained at the level above using Ahlrich's def2TZVP for all atoms. The coordinates of all the complexes are also provided in XYZ format in a separate file. The visualization and inspection of orbitals was done using Chemcraft (Version 1.8).[18]

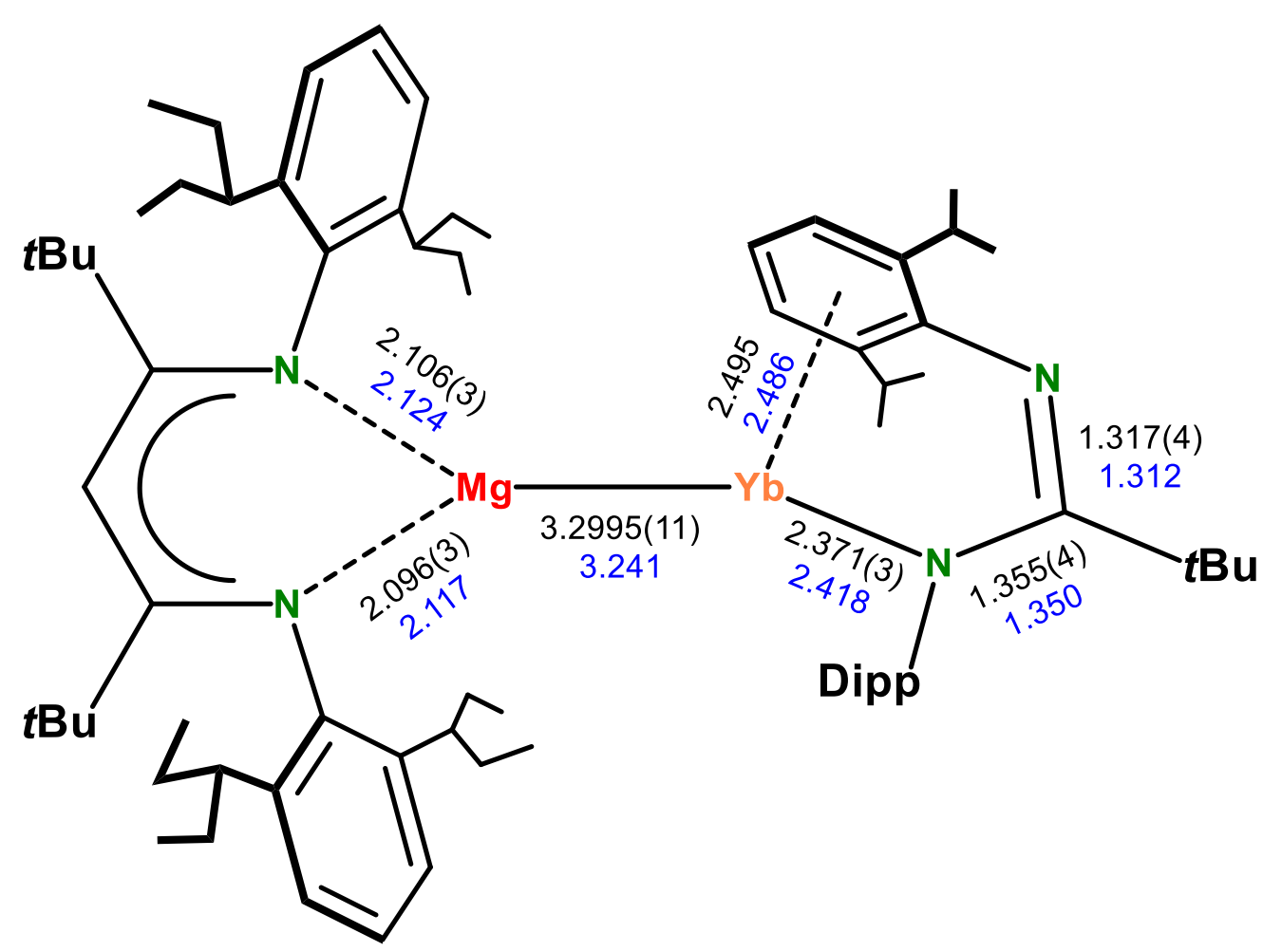

Fig. S66. Comparison of experimental (black) and calculated (blue) selected bond distances in Å. Experimental bond lengths are shown only for one of the two nearly identical molecules in the asymmetric unit.

Atoms-In-Molecules and Natural-Population-Analysis (NPA) analysis

QTAIM (Quantum Theory of Atoms In Molecules) analysis[19] was performed with the *AIMAll* program package[20] using the wavefunctions obtained from the single point calculations at the level: B3PW91(D3BJ)/def2TZVP//B3PW91(D3BJ)/def2SVP. A comparison of NPA and AIM charges is given in Table S4. Selected topological parameters for **1**-Yb are given in Table S5.

Table S4. NPA and AIM charges for **1**-Ca, **1**-Yb.

	**NPA**		**AIM**	
	**1**-Ca	**1**-Yb	**1**-Ca	**1**-Yb
**Mg**	+0.65	+0.89	+0.49	+0.59
**M (Ca or Yb)**	+1.28	+0.83	+1.41	+1.31
**N^{BDI*}**	-0.78	-0.77	-1.35	-1.35
**N^{Am*}**	-0.84	-0.80	-1.30	-1.29
**BDI***	-1.00	-0.97	-1.01	-1.06
**Am***	-0.93	-0.74	-0.90	-0.85
**BDI*+Mg**	-0.34	-0.09	-0.51	-0.46
**Am*+M**	+0.34	0.09	+0.51	+0.46

Table S5. Selected topological parameters for **1**-Yb from AIM analysis

**1**-Y®**(r)/®.**	**V(®a.u.®r)/a.®r)/e B^{-3}**		**H(r)*$\rho(r)^{-1}$/a.u.**		
**Mg–Yb**	0.004	-0.009	-0.0049	0.023	-0.2110
**Mg–N**	0.055	-0.050	0.0047	0.041	0.1150
**Mg–N**	0.056	-0.051	0.0047	0.042	0.1126
**Yb–C^{Ar}**	0.023	-0.023	-0.0005	0.027	-0.0171
**Yb–N**	0.051	-0.057	-0.0063	0.051	-0.1231

Definitions:
G(r): Kinetic energy de®ty at the *bcp*'s
V(r): Potential en®y density at *bcp*'s
H(r): Total ®®®ity at *bcp*'s; H(r)= G(r)+V(r)
®): Electron density at the *bcp*'s
H(r)*$\rho(r)^{-1}$: Total energy density ratio at *bcp*'s

Effective Oxidation State analysis

EOS (Effective Oxidation State) calculations[21] were performed using the APOST-3D software (Version 4)[22] with the wavefunctions obtained from the single point calculations at the level: B3PW91(D3BJ)/def2TZVP//B3PW91(D3BJ)/def2SVP. The topological fuzzy Voronoi cells (TFVC) atomic definition was employed.[23]

Table S6. EOS and reliability indexes for **1**-Yb and **1**-Ca.

	**1**-Yb	**1**-Ca
M	+2	+2
Am*	-1	-1
Mg	0	0
BDI*	-1	-1
R(%)	68.0	69.8

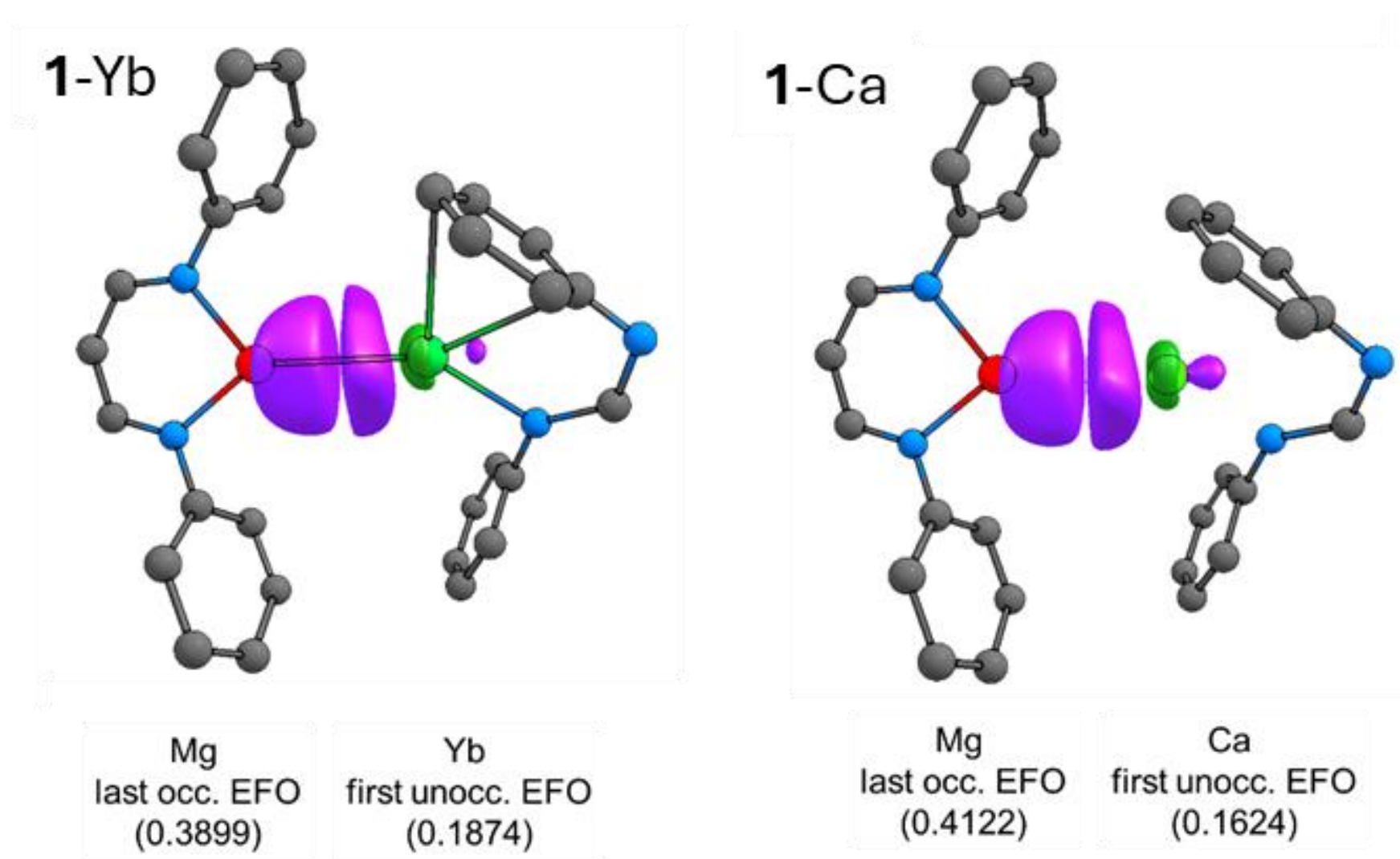

Fig. S67. Interacting Mg-Ca and Mg-Yb Effective Fragment Orbitals (EFOs) of **1**-Yb and **1**-Ca.

Orbital contribution analysis

The orbital contribution of selected MOs (Fig. S68), were calculated with the Gaussian population=orbitals keyword, with the orbital threshold set to 1%. These populations were calculated at the same level of theory as the wavefunctions, as described above. HOMO-1 to HOMO-7 (not depicted) correspond to Yb's *f* orbitals.

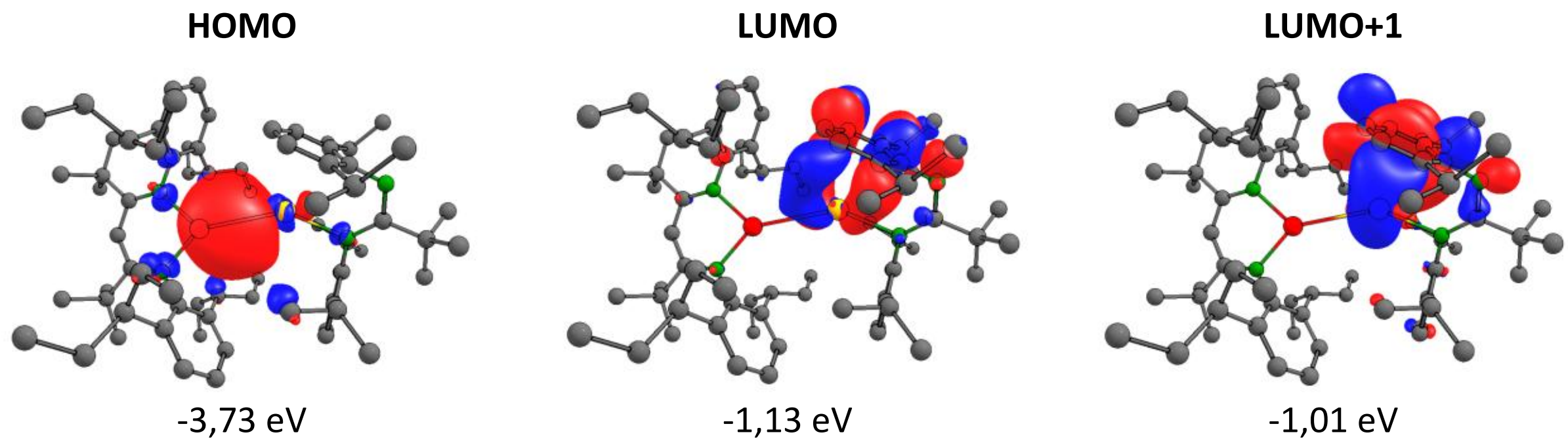

Fig. S68. Representations of the HOMO, LUMO and LUMO+1 for **1**-Yb and their energies. These Corresponding orbital contributions can be found below. Hydrogens omitted for clarity.

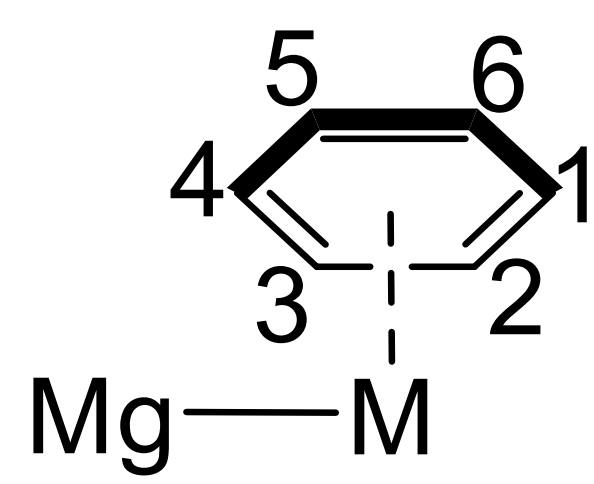

HOMO-7: Yb-f=99%

HOMO-6: Yb-f=99%

HOMO-5: Yb-f=99%

HOMO-4: Yb-f=99%

HOMO-3: Yb-f=98%

HOMO-2: Yb-f=99%

HOMO-1: Yb-f=95%

HOMO: Mg-s=33% Mg-p=20% Yb-s=19% Yb-p=11% Yb-d=5%

LUMO: C3-p=15% C6-p=15% C5-p=15% C2-p=0.14% Yb-d=12% Mg76-p=5%

LUMO+1: 1-p=20% C4-p=19% Yb-d=13% C5-p=7% Yb-s=6% C2-p=5% C3-p=4% C6-p=2%

EDA-NOCV analysis

EDA-NOCV (Energy Decomposition Analysis in combination with Natural Orbitals of Chemical Valence theory)[24] calculations were carried out using the Amsterdam Density Functional (ADF), AMS2024.102 software package[25] using the Gaussian16 optimised geometry. To align with the level of theory used for the optimisation, the functional B3PW91 was used in conjunction with a triple-z quality basis set with two sets of polarisation functions per atom, namely TZ2P.[26] Grimme's D3 long-range dispersion corrections and the Becke-Johnson damping function were also included in these calculations. Scalar relativistic effects were included using the zeroth-order regular approximation (ZORA).[27] Numerical quality was set

to very good in all calculations. EDA-NOCV analysis of **1**-Yb was conducted using the closed shell partitioning to give charged fragments [(BDI*)Mg^{-}] // [(Am*)Yb^{+}], or the open shell partitioning with neutral radical fragments [(BDI*)Mg$^{\bullet}$] // [(Am*)Yb$^{\bullet}$]. However, while we could obtain the EDA-NOCV analysis for the charged fragments (Table S7), all attempts to calculate the EDA-NOCV analysis for the open shell neutral radical fragments failed. Therefore, the system was truncated as follows: The *t*Bu groups in the ligand frameworks have been substituted by Me groups. The *i*Pr substituents in DIPP (Am*) and the Et_2CH-substituents in DIPeP (BDI*) have been trimmed in the outer faces, leaving the inner faces pointing to the metals intact (*i*Pr is now ethyl and 3-pentyl is now *n*-propyl) The truncated systems are abbreviated as **1'**-Yb and **1'**-Ca (Fig. S69). EDA-NOCV analysis on the two truncated systems, **1'**-Yb and **1'**-Ca, could be obtained for both, the closed shell partitioning [(BDI*)Mg^{-}] // [(Am*)Yb^{+}] and the open shell partitioning [(BDI*)Mg$^{\bullet}$] // [(Am*)Yb$^{\bullet}$]. The results are summarized in Table S8. Note that the energies for the closed shell partitioning obtained for the full structures (**1**-Yb and **1**-Ca) compare reasonable well to those for the truncated systems (**1'**-Yb and **1'**-Ca), showing that truncation does not have a major influence on the energy decomposition analysis.

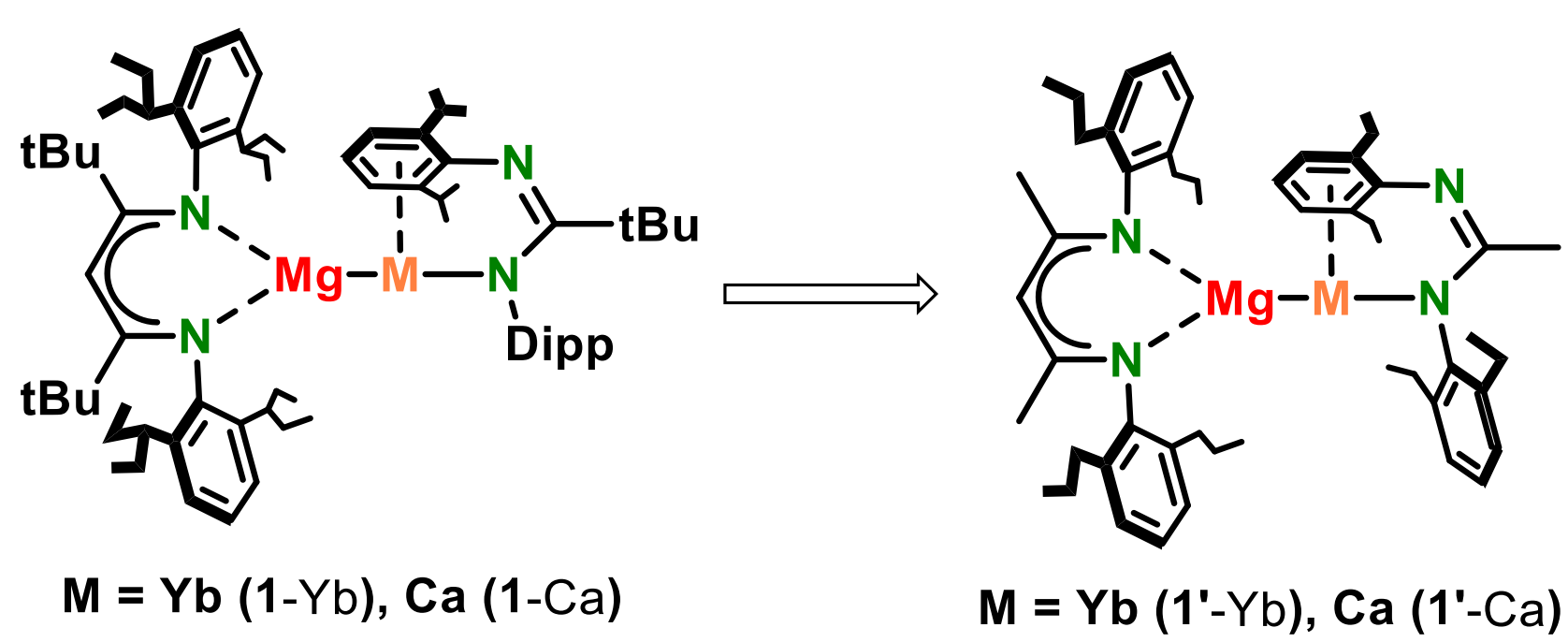

Fig. S69. Truncation of the **1**-Ca and **1**-Yb systems for the EDA-NOCV calculations.

Table S7. Energy decomposition analysis results for **1**-Ca and **1**-Yb (not truncated) using the $[Mg(BDI^*)]^-$ // $[Yb(Am^*)]^+$ fragmentation scheme.

	**Closed shell Charged fragments - M(II), Mg(0)**	
	**1**-Ca	**1**-Yb
ΔE_{Pauli}	91.26	115.32
ΔV_{elstat}	-153.48 (63%)	-176.16 (65%)
ΔE_{orb}	-52.40 (22%)	-55.83 (21%)
ΔE_{disp}	-37.49 (15%)	-39.09 (14%)
ΔE_{int}	-152.11	-155.76
NOCV; Mg→Yb	-33.03	-27.65
|v1|	0.675	0.716

Table S8. Energy decomposition analysis results for **1'**-Ca (M = Ca) and **1'**-Yb (M = Yb) using different fragmentation schemes.

	**Closed shell**				**Open shell**			
	**Charged fragments - M(II), Mg(0)**				**Neutral fragments - M(I), Mg(I)**			
	**1'**-Ca		**1'**-Yb		**1'**-Ca		**1'**-Yb	
$\mathbf{\Delta E_{Pauli}}$	90.3		114.3		80.3		87.4	
$\mathbf{\Delta V_{elstat}}$	-156.2	64%	-178.9	66%	-73.5	52%	-79.1	53%
$\mathbf{\Delta E_{orb}}$	-50.5	21%	-53.7	20%	-32.3	23%	-32.8	22%
$\mathbf{\Delta E_{disp}}$	-35.6	15%	-37.1	14%	-35.6	25%	-37.1	25%
$\mathbf{\Delta E_{int}}$	-151.9		-155.5		-61.0		-61.6	
**NOCV; Mg→Yb**	-27.9 kcal·mol^{-1}		-21.8 kcal·mol^{-1}		-11.6 kcal·mol^{-1}		-8.9 kcal·mol^{-1}	
	|v1| = 0.658		|v1| = 0.694		|v2| = 0.269		|v2| = 0.294	
**Yb→Mg**					-13.0 kcal·mol^{-1}		-20.1 kcal·mol^{-1}	
					|v1| = 0.514		|v1| = 0.517	

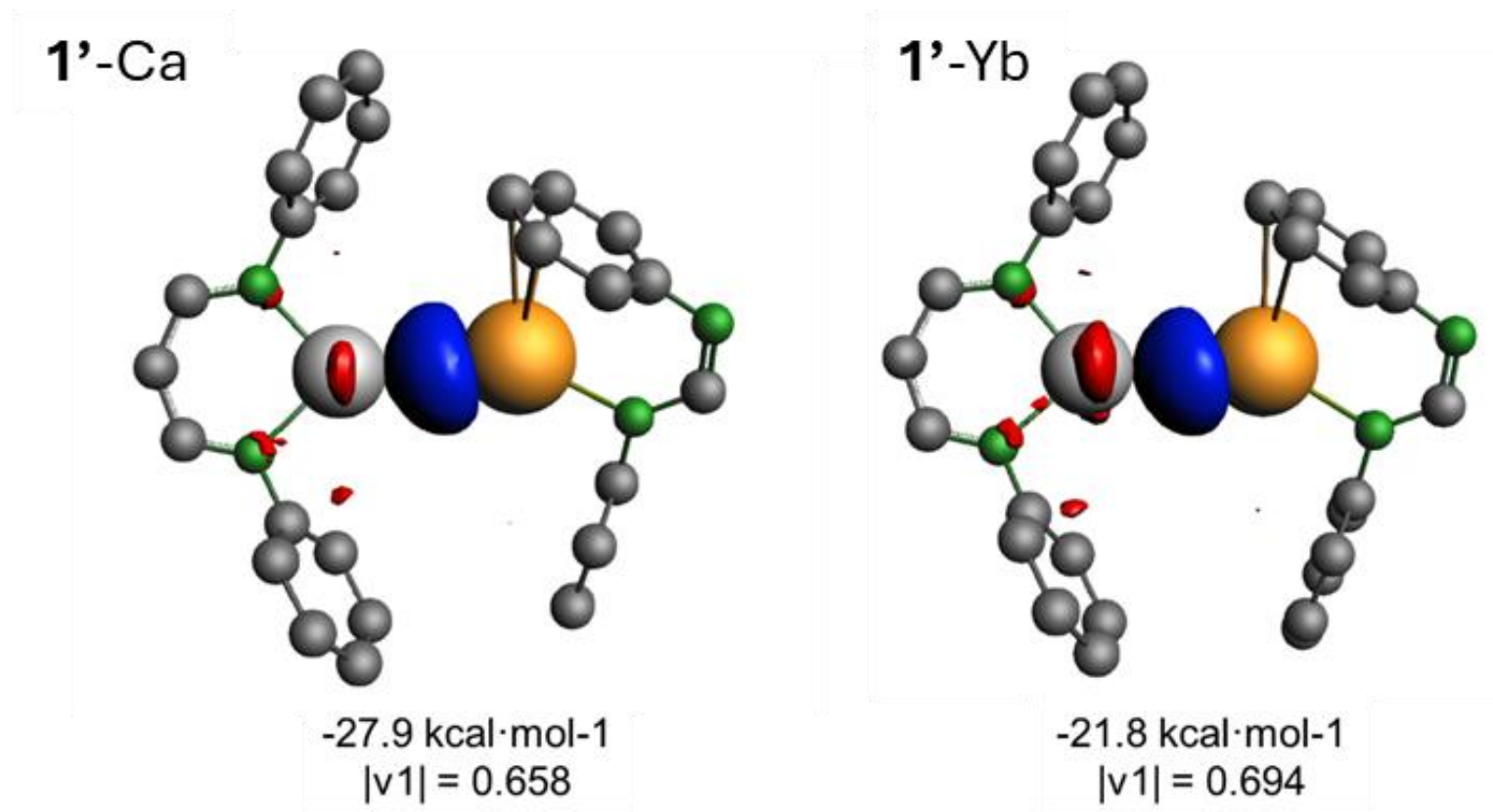

Fig. S70. NOCV deformation densities of **1**′-Ca and **1**′-Yb's major charge transfer interaction in the M(II)/Mg(0) partitioning scheme. CT goes from red to blue. Mg depicted in grey. Ca/Yb depicted in orange. Aliphatic substituents and hydrogens omitted for clarity.

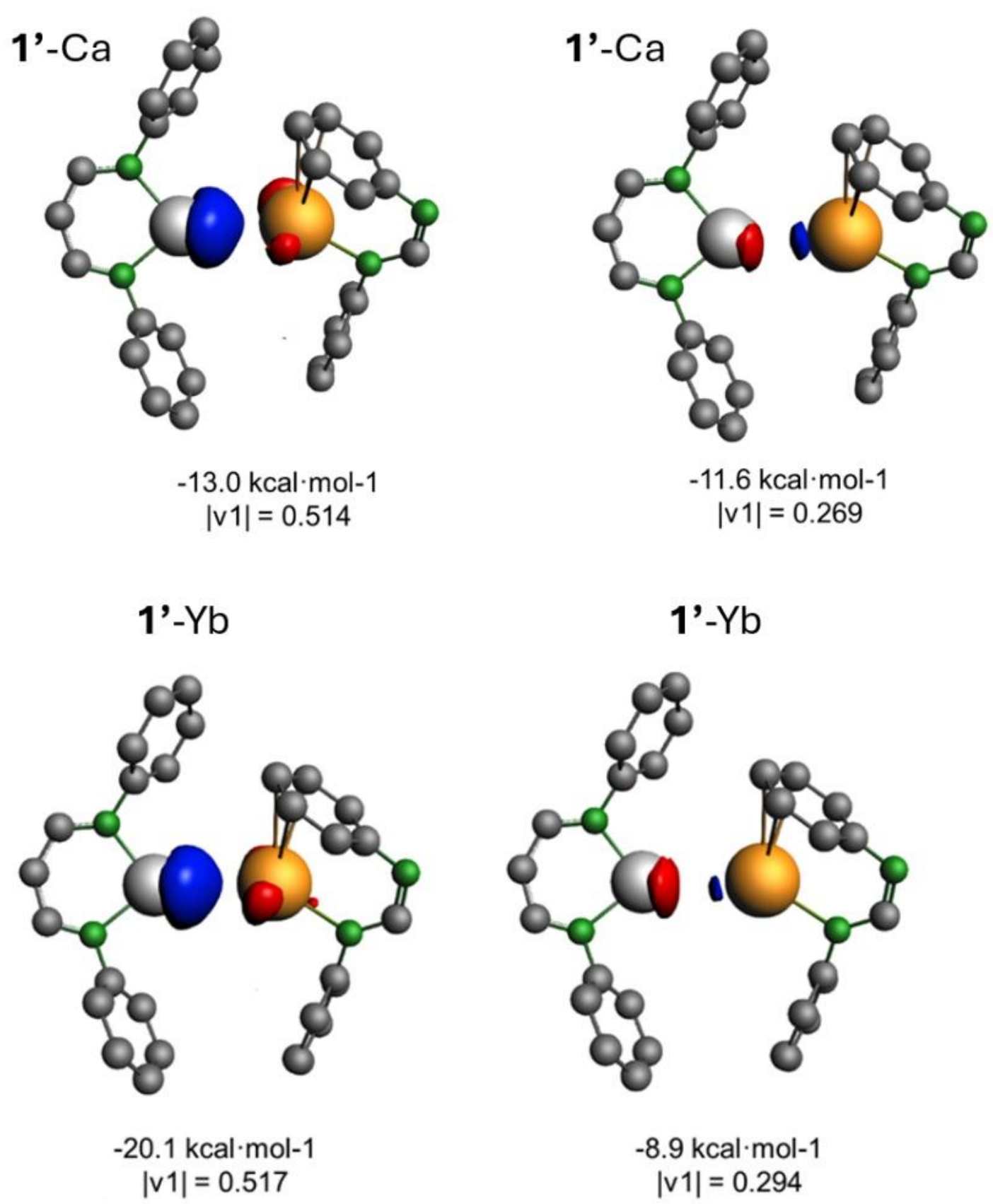

Fig. S71. NOCV deformation densities of **1**′-Ca (top) and **1**′-Yb (bottom) major charge transfer interaction in the M(I)/Mg(I) partitioning scheme. CT goes from red to blue. Mg depicted in grey. Ca/Yb depicted in orange Aliphatic substituents and hydrogens omitted for clarity.

XYZ-coordinates of (BDI*)Mg-Yb(Am*) (**1-**Yb)

190

1-Yb (optimized structure)

Yb	-1.619715117	-0.550189041	0.014796001
N	-3.840206275	0.395004028	0.167315012
N	-4.836673346	-1.431744103	1.168067087
C	-4.922916352	-0.208158015	0.700954053
C	-6.324509464	0.420893030	0.903633067
C	-6.577252456	1.778117129	0.243088017
H	-7.609963554	2.085347148	0.472041034
H	-6.480117474	1.740730127	-0.848786062
H	-5.907917427	2.565404185	0.609189045
C	-6.514064459	0.583172040	2.421987176
H	-6.408597476	-0.384127028	2.930195210
H	-7.513476549	0.992333069	2.639764188
H	-5.769427402	1.277718090	2.840926204
C	-7.366794543	-0.571569040	0.366234027
H	-7.264189508	-1.546191113	0.859143061
H	-7.243574509	-0.722128054	-0.718272050
H	-8.383545602	-0.185213013	0.540059038
C	-3.780644270	1.679332120	-0.397668029
C	-4.077127296	1.864501135	-1.773703130
C	-3.919480282	3.131569225	-2.336836169
H	-4.170769301	3.290222237	-3.387338243
C	-3.451228247	4.204810305	-1.579933116
H	-3.326945239	5.187340376	-2.041066146
C	-3.142058227	4.016056289	-0.237241017
H	-2.774583200	4.856895352	0.355641026
C	-3.309256240	2.769879200	0.374430027
C	-4.588337332	0.692103050	-2.590219185
H	-5.124365368	0.030677002	-1.892270134
C	-5.568982415	1.090063079	-3.690793264
H	-6.390329483	1.705654121	-3.294061238
H	-6.008256418	0.192948014	-4.153672298
H	-5.077944368	1.661460121	-4.494197323
C	-3.433781246	-0.119457009	-3.177646228
H	-2.823986203	0.489085035	-3.858770279
H	-3.801106273	-0.997737069	-3.728633269
H	-2.756635201	-0.497084036	-2.392982172
C	-3.037674221	2.598004187	1.855201136
H	-3.445981248	1.615559117	2.132346152
C	-3.754360269	3.648207265	2.705099195
H	-3.344290240	4.656788335	2.538528183
H	-3.637327264	3.420293248	3.776312271
H	-4.830873346	3.683001267	2.479825180
C	-1.540624110	2.582313187	2.158051154
H	-0.999419070	1.793893131	1.602798114
H	-1.360640096	2.419548177	3.230985231
H	-1.059722077	3.526773252	1.867861135
C	-3.617692259	-2.057699150	1.131422081
C	-3.253410234	-2.877606205	0.019941001
C	-1.983124142	-3.472080248	-0.002821000
H	-1.687580120	-4.093810297	-0.848511059
C	-1.060592076	-3.259664236	1.025857072
H	-0.063159004	-3.693190268	0.965399069
C	-1.437168101	-2.492496181	2.131928155
H	-0.721308051	-2.345077168	2.938118211
C	-2.697077196	-1.888331135	2.209130157
C	-4.276745305	-3.108518222	-1.072927076
H	-4.727436339	-2.125703152	-1.289062094
C	-3.688572265	-3.662928261	-2.365220168
H	-2.854476206	-3.052434218	-2.739531197
H	-4.460332319	-3.693865265	-3.148915227
H	-3.318141237	-4.692352338	-2.235299163
C	-5.397451409	-4.013927287	-0.549260039
H	-5.005589359	-5.017077363	-0.316719023
H	-6.192474449	-4.123335297	-1.303479094
H	-5.839357434	-3.588084257	0.361253026
C	-3.117586224	-1.067790078	3.408765244
H	-3.690755264	-0.213633015	3.016425220
C	-1.946440141	-0.518784037	4.215981305
H	-1.404809103	-1.316417097	4.749079340
H	-2.307571164	0.190519014	4.975542357
H	-1.221784086	0.011895001	3.580109260
C	-4.073398295	-1.885633135	4.282688310
H	-4.924849352	-2.242536159	3.686706264
H	-4.461757319	-1.275553093	5.113269367
H	-3.555838254	-2.759001200	4.710969338
Mg	1.547383111	0.086919006	-0.246835018
N	2.919242209	1.695350124	-0.350266025
N	3.111469227	-1.313725093	-0.571177042
C	4.205525303	1.591328112	-0.661627049
C	4.815455349	0.343576025	-0.913271067
H	5.860767417	0.431845031	-1.177262086
C	4.371092317	-0.994225074	-0.859127064
C	5.204597375	2.788562200	-0.837684061
C	4.642048336	4.197400305	-0.608728042
H	3.761985268	4.414756319	-1.222928088
H	5.423766388	4.924529353	-0.878859062
H	4.367419314	4.380992313	0.435145031
C	6.393838458	2.622067189	0.127495009
H	6.063827438	2.635065189	1.175677084
H	7.098287519	3.457500251	-0.009579001
H	6.946168481	1.687551121	-0.038491003

C	5.743955402	2.755569200	-2.284046167
H	6.292643469	1.832144131	-2.509983182
H	6.431757447	3.601616261	-2.439178176
H	4.930689357	2.844303207	-3.018173219
C	5.530070372	-2.009849144	-1.155084083
C	6.577761472	-1.875077134	-0.033955002
H	7.396761533	-2.593741188	-0.197136014
H	6.134091434	-2.088091149	0.947860066
H	7.012589487	-0.867391061	0.008861001
C	6.192885461	-1.652628121	-2.502689177
H	6.984720526	-2.384744173	-2.724776196
H	6.655508458	-0.657696046	-2.507948178
H	5.465756391	-1.684574123	-3.327124238
C	5.155784372	-3.495261249	-1.252070091
H	4.375848313	-3.691758267	-1.995753142
H	4.817203349	-3.917093280	-0.301407022
H	6.054716435	-4.052029292	-1.559484115
C	2.240835161	2.846913205	0.101491007
C	1.258933091	3.438751247	-0.729042051
C	0.562957041	4.553752328	-0.247829018
H	-0.184262013	5.032251362	-0.881517061
C	0.803621059	5.062458366	1.024210072
H	0.260158019	5.942994417	1.375186099
C	1.715992122	4.423596321	1.861826133
H	1.873620134	4.807018348	2.871287209
C	2.431204173	3.304534238	1.430568102
C	1.011814073	2.903718207	-2.126951153
H	1.206231085	1.814628132	-2.088168149
C	2.024751145	3.463735250	-3.138983226
H	3.030332220	3.214973232	-2.774728200
H	1.917128140	2.917073212	-4.090071294
C	1.925360138	4.960854355	-3.389809242
H	2.038962148	5.532962378	-2.455636176
H	0.954315070	5.239095377	-3.828840273
H	2.709565197	5.297934389	-4.085206293
C	-0.442913032	3.055386217	-2.570633187
H	-1.083701077	2.586705189	-1.804280129
H	-0.739488052	4.115099299	-2.588422184
C	-0.737716054	2.443385177	-3.929173285
H	-0.201988014	2.954552212	-4.743302343
H	-1.811907129	2.502691178	-4.154804298
H	-0.446051032	1.381557102	-3.956507285
C	4.081833296	3.471736252	3.371330245
C	5.155927372	2.760430201	4.183798301
H	5.695526423	3.465816250	4.833965350
H	5.899067414	2.275499165	3.530052256
H	4.730835340	1.978100144	4.831075349
C	2.601592188	-2.619048190	-0.407516029
C	1.809440131	-3.184209227	-1.438315103
C	1.353928096	-4.499428325	-1.289491093
H	0.775837057	-4.960980357	-2.091840152
C	1.636480119	-5.238386374	-0.144052011
H	1.297502095	-6.273252457	-0.058429004
C	2.316448166	-4.632564331	0.910300065
H	2.500686178	-5.200412372	1.823950129
C	2.773796202	-3.313892240	0.816929057
C	1.490487106	-2.386731170	-2.688415194
H	1.509684106	-1.322312095	-2.389466170
C	0.068431005	-2.648944191	-3.192312228
H	-0.059663004	-3.701400268	-3.493420252
H	-0.609760043	-2.504646181	-2.332102165
C	-0.362618026	-1.743602125	-4.334087312
H	-0.246790018	-0.683882048	-4.058463294
H	-1.419115103	-1.906580135	-4.593877331
H	0.228334016	-1.917615137	-5.245612380
C	2.570169183	-2.526141181	-3.773145272
H	2.360970171	-1.798429127	-4.574286332
H	3.524735253	-2.204589160	-3.333308240
C	2.720741198	-3.918076284	-4.368185314
H	3.532202256	-3.942373283	-5.111704368
H	1.801023127	-4.249315307	-4.875977351
H	2.956967213	-4.664882334	-3.594238260
C	3.415192244	-2.632722188	2.008035145
H	3.915098281	-1.728987123	1.628611115
C	4.490145323	-3.487063251	2.691905192
H	4.041550292	-4.395535316	3.125912225
H	5.189782375	-3.848693278	1.921005136
C	5.263164379	-2.740245195	3.770357270
H	5.707151389	-1.813325128	3.371651244
H	6.081892438	-3.355345240	4.173914302
H	4.618687335	-2.455744176	4.616191333
C	2.333007170	-2.124735154	2.986757215
H	2.795847203	-1.389978100	3.662674262
H	1.587457112	-1.556933110	2.400083175
C	1.629511119	-3.183067231	3.823027277
H	0.899159063	-2.715733197	4.501636323
H	2.334979170	-3.745097270	4.454547321
H	1.087031079	-3.912024280	3.203720231
H	4.530068328	4.323420310	2.831822202
H	3.344236243	3.913561281	4.061100290
C	3.387227243	2.568069185	2.348198171
H	4.180291300	2.148336153	1.714645123
C	2.698497195	1.347652096	2.983667216
H	2.227499159	0.763005054	2.174037154
H	3.470848250	0.686010050	3.407217247
C	1.646734118	1.662314118	4.032632290
H	2.083497149	2.129203151	4.929294356
H	1.131774079	0.745294052	4.357020314

H	0.885704065	2.347956168	3.633367261

127

Truncated **1'**-Yb (non-optimized)

Yb	-1.619715000	-0.550189000	0.014796000
N	-3.840206000	0.395004000	0.167315000
N	-4.836673000	-1.431744000	1.168067000
C	-4.922916000	-0.208158000	0.700954000
C	-6.324509000	0.420893000	0.903633000
C	-3.780644000	1.679332000	-0.397668000
C	-4.077127000	1.864501000	-1.773703000
C	-3.919480000	3.131569000	-2.336836000
H	-4.170769000	3.290222000	-3.387338000
C	-3.451228000	4.204810000	-1.579933000
H	-3.326945000	5.187340000	-2.041066000
C	-3.142058000	4.016056000	-0.237241000
H	-2.774583000	4.856895000	0.355641000
C	-3.309256000	2.769879000	0.374430000
C	-4.588337000	0.692103000	-2.590219000
H	-5.124365000	0.030677000	-1.892270000
C	-3.433781000	-0.119457000	-3.177646000
H	-2.823986000	0.489085000	-3.858770000
H	-3.801106000	-0.997737000	-3.728633000
H	-2.756635000	-0.497084000	-2.392982000
C	-3.037674000	2.598004000	1.855201000
H	-3.445981000	1.615559000	2.132346000
C	-1.540624000	2.582313000	2.158051000
H	-0.999419000	1.793893000	1.602798000
H	-1.360640000	2.419548000	3.230985000
H	-1.059722000	3.526773000	1.867861000
C	-3.617692000	-2.057699000	1.131422000
C	-3.253410000	-2.877606000	0.019941000
C	-1.983124000	-3.472080000	-0.002821000
H	-1.687580000	-4.093810000	-0.848511000
C	-1.060592000	-3.259664000	1.025857000
H	-0.063159000	-3.693190000	0.965399000
C	-1.437168000	-2.492496000	2.131928000
H	-0.721308000	-2.345077000	2.938118000
C	-2.697077000	-1.888331000	2.209130000
C	-4.276745000	-3.108518000	-1.072927000
H	-4.727436000	-2.125703000	-1.289062000
C	-3.688572000	-3.662928000	-2.365220000
H	-2.854476000	-3.052434000	-2.739531000
H	-4.460332000	-3.693865000	-3.148915000
H	-3.318141000	-4.692352000	-2.235299000
C	-3.117586000	-1.067790000	3.408765000
H	-3.690755000	-0.213633000	3.016425000
C	-1.946440000	-0.518784000	4.215981000
H	-1.404809000	-1.316417000	4.749079000
H	-2.307571000	0.190519000	4.975542000
H	-1.221784000	0.011895000	3.580109000
H	-5.207147136	1.059225623	-3.395635039
H	-4.984306721	-3.845788579	-0.723372277
H	-3.681509463	-1.704888618	4.073968487
H	-3.482586224	3.416859415	2.401029657
H	-7.024584120	-0.245300888	0.421470651
H	-6.466341740	1.398540498	0.467182723
H	-6.459958412	0.548338840	1.967499070
Mg	1.547383000	0.086919000	-0.246835000
N	2.919242000	1.695350000	-0.350266000
N	3.111469000	-1.313725000	-0.571177000
C	4.205525000	1.591328000	-0.661627000
C	4.815455000	0.343576000	-0.913271000
H	5.860767000	0.431845000	-1.177262000
C	4.371092000	-0.994225000	-0.859127000
C	5.204597000	2.788562000	-0.837684000
C	5.530070000	-2.009849000	-1.155084000
C	2.240835000	2.846913000	0.101491000
C	1.258933000	3.438751000	-0.729042000
C	0.562957000	4.553752000	-0.247829000
H	-0.184262000	5.032251000	-0.881517000
C	0.803621000	5.062458000	1.024210000
H	0.260158000	5.942994000	1.375186000
C	1.715992000	4.423596000	1.861826000
H	1.873620000	4.807018000	2.871287000
C	2.431204000	3.304534000	1.430568000
C	1.011814000	2.903718000	-2.126951000
H	1.206231000	1.814628000	-2.088168000
C	-0.442913000	3.055386000	-2.570633000
H	-1.083701000	2.586705000	-1.804280000
H	-0.739488000	4.115099000	-2.588422000
C	-0.737716000	2.443385000	-3.929173000
H	-0.201988000	2.954552000	-4.743302000
H	-1.811907000	2.502691000	-4.154804000
H	-0.446051000	1.381557000	-3.956507000
C	2.601592000	-2.619048000	-0.407516000
C	1.809440000	-3.184209000	-1.438315000
C	1.353928000	-4.499428000	-1.289491000
H	0.775837000	-4.960980000	-2.091840000
C	1.636480000	-5.238386000	-0.144052000
H	1.297502000	-6.273252000	-0.058429000
C	2.316448000	-4.632564000	0.910300000
H	2.500686000	-5.200412000	1.823950000
C	2.773796000	-3.313892000	0.816929000
C	1.490487000	-2.386731000	-2.688415000
H	1.509684000	-1.322312000	-2.389466000
C	0.068431000	-2.648944000	-3.192312000
H	-0.059663000	-3.701400000	-3.493420000

H	-0.609760000	-2.504646000	-2.332102000
C	-0.362618000	-1.743602000	-4.334087000
H	-0.246790000	-0.683882000	-4.058463000
H	-1.419115000	-1.906580000	-4.593877000
H	0.228334000	-1.917615000	-5.245612000
C	3.415192000	-2.632722000	2.008035000
H	3.915098000	-1.728987000	1.628611000
C	2.333007000	-2.124735000	2.986757000
H	2.795847000	-1.389978000	3.662674000
H	1.587457000	-1.556933000	2.400083000
C	1.629511000	-3.183067000	3.823027000
H	0.899159000	-2.715733000	4.501636000
H	2.334979000	-3.745097000	4.454547000
H	1.087031000	-3.912024000	3.203720000
C	3.387227000	2.568069000	2.348198000
H	4.180291000	2.148336000	1.714645000
C	2.698497000	1.347652000	2.983667000
H	2.227499000	0.763005000	2.174037000
H	3.470848000	0.686010000	3.407217000
C	1.646734000	1.662314000	4.032632000
H	2.083497000	2.129203000	4.929294000
H	1.131774000	0.745294000	4.357020000
H	0.885704000	2.347956000	3.633367000
H	5.476629449	2.732971138	-1.881383192
H	5.879336438	-1.740606808	-2.140944841
H	1.642492483	3.450750554	-2.812078751
H	2.197105947	-2.654439548	-3.460050793
H	4.078998200	-3.327143521	2.501532802
H	3.739321663	3.249619004	3.108407800
H	5.988278521	2.680218580	-0.102493564
H	6.221585129	-1.935119806	-0.328874357
H	4.833307000	3.784975716	-0.648730453
H	5.285116713	-3.060766879	-1.199463024

190

1-Ca (optimized structure)

Ca	-1.760533000	-0.560453000	0.050704000
Mg	1.393108000	0.055109000	-0.253864000
N	2.747138000	1.688323000	-0.352585000
N	2.991456000	-1.317442000	-0.568820000
N	-3.968726000	0.290982000	0.170847000
N	-4.940187000	-1.552173000	1.165438000
C	4.035576000	1.605631000	-0.661618000
C	4.666556000	0.368623000	-0.912881000
H	5.710178000	0.474452000	-1.176929000
C	4.245011000	-0.976439000	-0.857836000
C	2.051503000	2.829369000	0.098398000
C	2.507371000	-2.632199000	-0.403518000
C	2.233800000	3.290718000	1.427490000
C	2.695028000	-3.323026000	0.821024000
C	1.063292000	3.408008000	-0.734150000
C	3.329685000	-2.630877000	2.009612000
H	3.813978000	-1.719709000	1.627718000
C	5.015925000	2.819019000	-0.836428000
C	0.830110000	2.872640000	-2.134320000
H	1.045839000	1.787685000	-2.098208000
C	-5.044825000	-0.330896000	0.698567000
C	-3.928030000	1.576123000	-0.394972000
C	1.500897000	4.398688000	1.857781000
H	1.651915000	4.784938000	2.867179000
C	1.725983000	-3.213476000	-1.433545000
C	4.432164000	4.219068000	-0.606252000
H	3.548695000	4.423869000	-1.219805000
H	5.202809000	4.958086000	-0.876017000
H	4.155243000	4.397744000	0.437854000
C	5.421741000	-1.971415000	-1.155584000
C	0.349533000	4.512049000	-0.253726000
H	-0.402678000	4.980340000	-0.889173000
C	2.244308000	-2.138971000	2.992800000
H	2.700002000	-1.399919000	3.669008000
H	1.490022000	-1.579130000	2.410112000
C	2.261732000	-4.649684000	0.916151000
H	2.458184000	-5.213540000	1.829737000
C	-3.712398000	-2.161827000	1.131427000
C	4.420409000	-3.468096000	2.689930000
H	3.987791000	-4.383594000	3.125458000
H	5.123108000	-3.818647000	1.916705000
C	3.882598000	3.490058000	3.366433000
H	4.314764000	4.349076000	2.825409000
H	3.137759000	3.919451000	4.056317000
C	3.203668000	2.572403000	2.345185000
H	4.003549000	2.166981000	1.710965000
C	1.395519000	-2.425070000	-2.686466000
H	1.410038000	-1.359009000	-2.394372000
C	-4.224078000	1.755554000	-1.771651000
C	-6.456546000	0.277027000	0.896684000
C	0.579791000	5.023584000	1.019128000
H	0.022744000	5.895917000	1.369361000
C	-3.479824000	2.674498000	0.379437000
C	-3.334368000	-2.974359000	0.019704000
C	-0.626009000	2.997595000	-2.581550000
H	-1.259098000	2.511720000	-1.819583000
H	-0.943759000	4.051218000	-2.594033000
C	1.294699000	-4.536621000	-1.282847000
H	0.725141000	-5.009776000	-2.084589000
C	-4.709921000	0.574012000	-2.590243000
H	-5.249055000	-0.090711000	-1.897564000

C	5.075649000	-3.463918000	-1.249760000
H	4.747898000	-3.891006000	-0.297658000
H	5.984387000	-4.003570000	-1.558939000
H	4.297609000	-3.676668000	-1.990917000
C	5.556161000	2.795434000	-2.282703000
H	6.120683000	1.881551000	-2.508400000
H	6.229488000	3.653116000	-2.437575000
H	4.741924000	2.870092000	-3.017260000
C	6.470520000	-1.816165000	-0.038099000
H	6.887244000	-0.800774000	0.002187000
H	7.301897000	-2.520047000	-0.203158000
H	6.034083000	-2.035941000	0.945434000
C	-2.796384000	-1.980207000	2.210703000
C	1.591240000	-5.268785000	-0.136585000
H	1.271286000	-6.309574000	-0.049446000
C	1.556989000	-3.208315000	3.828511000
H	1.023355000	-3.943272000	3.208564000
H	0.821678000	-2.752443000	4.509639000
H	2.271335000	-3.762171000	4.457277000
C	-1.143403000	-3.338765000	1.035921000
H	-0.140370000	-3.759747000	0.977331000
C	6.073514000	-1.603181000	-2.505671000
H	5.344178000	-1.648218000	-3.327536000
H	6.877503000	-2.321176000	-2.730282000
H	6.518221000	-0.600132000	-2.512871000
C	6.208027000	2.670700000	0.128243000
H	5.878531000	2.678760000	1.176612000
H	6.899534000	3.516824000	-0.009246000
H	6.774477000	1.744729000	-0.037944000
C	-0.027922000	-2.698705000	-3.180709000
H	-0.153708000	-3.755795000	-3.466123000
H	-0.701599000	-2.541952000	-2.319563000
C	-2.058044000	-3.556038000	0.001647000
H	-1.751724000	-4.170563000	-0.845453000
C	-1.530586000	-2.573605000	2.139104000
H	-0.818644000	-2.417070000	2.947164000
C	-6.730703000	1.626597000	0.228806000
H	-6.075514000	2.427033000	0.591933000
H	-7.768818000	1.917517000	0.454655000
H	-6.631582000	1.585111000	-0.862798000
C	5.185249000	-2.709426000	3.765958000
H	6.015814000	-3.311166000	4.165480000
H	4.539634000	-2.436623000	4.614776000
H	5.612038000	-1.774802000	3.366451000
C	-7.483105000	-0.734483000	0.364571000
H	-7.357907000	-0.888674000	-0.719174000
H	-8.505647000	-0.363100000	0.536922000
H	-7.365155000	-1.704850000	0.862333000
C	-4.352128000	-3.217788000	-1.075576000

H	-4.813818000	-2.240532000	-1.293834000
C	-4.089672000	3.025829000	-2.333167000
H	-4.341091000	3.180768000	-3.384079000
C	-3.212231000	2.507938000	1.861607000
H	-3.608808000	1.520594000	2.138618000
C	1.834047000	3.456217000	-3.142069000
H	2.843494000	3.225856000	-2.776655000
H	1.739146000	2.910978000	-4.095323000
C	-3.643600000	4.107107000	-1.574423000
H	-3.536869000	5.092055000	-2.034777000
C	2.470793000	-2.564310000	-3.775817000
H	2.251464000	-1.844024000	-4.580938000
H	3.425276000	-2.232148000	-3.343700000
C	-0.904764000	2.387689000	-3.944568000
H	-0.591104000	1.332259000	-3.977673000
H	-0.377541000	2.914768000	-4.754096000
H	-1.979295000	2.426474000	-4.173558000
C	-3.333735000	3.923769000	-0.231441000
H	-2.983671000	4.771190000	0.362487000
C	-3.230024000	-1.164395000	3.409052000
H	-3.812498000	-0.316936000	3.015517000
C	-0.466513000	-1.811541000	-4.333987000
H	-1.523117000	-1.982907000	-4.587852000
H	0.121925000	-1.995977000	-5.245155000
H	-0.355035000	-0.747371000	-4.073985000
C	2.630064000	-3.958932000	-4.362230000
H	1.708259000	-4.304069000	-4.856883000
H	2.883649000	-4.697256000	-3.585690000
H	3.433188000	-3.979714000	-5.114885000
C	4.970308000	2.799873000	4.179013000
H	4.560343000	2.010876000	4.827957000
H	5.497464000	3.516134000	4.827519000
H	5.721633000	2.327629000	3.525355000
C	-3.537114000	-0.226618000	-3.156352000
H	-2.916460000	0.389678000	-3.820109000
H	-3.886855000	-1.103502000	-3.720647000
H	-2.877241000	-0.606257000	-2.359092000
C	-6.649383000	0.444078000	2.414231000
H	-6.529316000	-0.518934000	2.927381000
H	-7.655228000	0.838610000	2.629271000
H	-5.916086000	1.152261000	2.830235000
C	-3.755412000	-3.767893000	-2.365757000
H	-2.926430000	-3.150258000	-2.739269000
H	-4.525291000	-3.808012000	-3.150915000
H	-3.375550000	-4.793563000	-2.233054000
C	-5.678147000	0.956323000	-3.707253000
H	-6.511583000	1.565728000	-3.326433000
H	-6.101331000	0.052291000	-4.171530000
H	-5.180830000	1.528012000	-4.506581000

C	1.706447000	4.952020000	-3.388003000
H	0.730465000	5.213423000	-3.826505000
H	2.484384000	5.306039000	-4.082034000
H	1.809068000	5.523160000	-2.451976000
C	-1.716562000	2.514430000	2.172277000
H	-1.157934000	1.734627000	1.621806000
H	-1.539659000	2.355588000	3.246250000
H	-1.248300000	3.465484000	1.883195000
C	2.539496000	1.339713000	2.982709000
H	2.078425000	0.745921000	2.174204000
H	3.325215000	0.693306000	3.405212000
C	-3.947547000	3.549315000	2.706772000
H	-3.549823000	4.563053000	2.541855000
H	-3.833594000	3.323764000	3.778769000
H	-5.023088000	3.569677000	2.475241000
C	-5.463748000	-4.135707000	-0.553574000
H	-5.060760000	-5.134086000	-0.319526000
H	-6.255409000	-4.255002000	-1.309826000
H	-5.913216000	-3.715018000	0.355614000
C	-2.068408000	-0.601820000	4.220653000
H	-1.517895000	-1.393564000	4.753458000
H	-2.441097000	0.100668000	4.980978000
H	-1.348263000	-0.059699000	3.589100000
C	-4.178894000	-1.993825000	4.279828000
H	-5.024107000	-2.360912000	3.681159000
H	-4.577045000	-1.388689000	5.109386000
H	-3.652183000	-2.861033000	4.709433000
C	1.484804000	1.635441000	4.034242000
H	0.987978000	0.709254000	4.360884000
H	0.710139000	2.306734000	3.636822000
H	1.915119000	2.111112000	4.929419000

127

Truncated **1**′-Ca (non-optimized)

Ca	-1.760533000	-0.560453000	0.050704000
N	-3.968726000	0.290982000	0.170847000
N	-4.940187000	-1.552173000	1.165438000
C	-5.044825000	-0.330896000	0.698567000
C	-3.928030000	1.576123000	-0.394972000
C	-3.712398000	-2.161827000	1.131427000
C	-4.224078000	1.755554000	-1.771651000
C	-6.456546000	0.277027000	0.896684000
C	-3.479824000	2.674498000	0.379437000
C	-3.334368000	-2.974359000	0.019704000
C	-4.709921000	0.574012000	-2.590243000
H	-5.249055000	-0.090711000	-1.897564000
C	-2.796384000	-1.980207000	2.210703000
C	-1.143403000	-3.338765000	1.035921000
H	-0.140370000	-3.759747000	0.977331000
C	-2.058044000	-3.556038000	0.001647000
H	-1.751724000	-4.170563000	-0.845453000
C	-1.530586000	-2.573605000	2.139104000
H	-0.818644000	-2.417070000	2.947164000
C	-4.352128000	-3.217788000	-1.075576000
H	-4.813818000	-2.240532000	-1.293834000
C	-4.089672000	3.025829000	-2.333167000
H	-4.341091000	3.180768000	-3.384079000
C	-3.212231000	2.507938000	1.861607000
H	-3.608808000	1.520594000	2.138618000
C	-3.643600000	4.107107000	-1.574423000
H	-3.536869000	5.092055000	-2.034777000
C	-3.333735000	3.923769000	-0.231441000
H	-2.983671000	4.771190000	0.362487000
C	-3.230024000	-1.164395000	3.409052000
H	-3.812498000	-0.316936000	3.015517000
C	-3.537114000	-0.226618000	-3.156352000
H	-2.916460000	0.389678000	-3.820109000
H	-3.886855000	-1.103502000	-3.720647000
H	-2.877241000	-0.606257000	-2.359092000
C	-3.755412000	-3.767893000	-2.365757000
H	-2.926430000	-3.150258000	-2.739269000
H	-4.525291000	-3.808012000	-3.150915000
H	-3.375550000	-4.793563000	-2.233054000
C	-1.716562000	2.514430000	2.172277000
H	-1.157934000	1.734627000	1.621806000
H	-1.539659000	2.355588000	3.246250000
H	-1.248300000	3.465484000	1.883195000
C	-2.068408000	-0.601820000	4.220653000
H	-1.517895000	-1.393564000	4.753458000
H	-2.441097000	0.100668000	4.980978000
H	-1.348263000	-0.059699000	3.589100000
H	-3.788255894	-1.808657002	4.072153520
H	-5.052012774	-3.962657695	-0.726677104
H	-5.320959870	0.930496161	-3.406300928
H	-3.670746761	3.321427805	2.404191218
H	-6.574088529	1.220334723	0.384088357
H	-7.205262453	-0.375381253	0.472199437
H	-6.573043182	0.408598769	1.962290530
Mg	1.393108000	0.055109000	-0.253864000
N	2.747138000	1.688323000	-0.352585000
N	2.991456000	-1.317442000	-0.568820000
C	4.035576000	1.605631000	-0.661618000
C	4.666556000	0.368623000	-0.912881000
H	5.710178000	0.474452000	-1.176929000
C	4.245011000	-0.976439000	-0.857836000
C	2.051503000	2.829369000	0.098398000
C	2.507371000	-2.632199000	-0.403518000

C	2.233800000	3.290718000	1.427490000
C	2.695028000	-3.323026000	0.821024000
C	1.063292000	3.408008000	-0.734150000
C	3.329685000	-2.630877000	2.009612000
H	3.813978000	-1.719709000	1.627718000
C	5.015925000	2.819019000	-0.836428000
C	0.830110000	2.872640000	-2.134320000
H	1.045839000	1.787685000	-2.098208000
C	1.500897000	4.398688000	1.857781000
H	1.651915000	4.784938000	2.867179000
C	1.725983000	-3.213476000	-1.433545000
C	5.421741000	-1.971415000	-1.155584000
C	0.349533000	4.512049000	-0.253726000
H	-0.402678000	4.980340000	-0.889173000
C	2.244308000	-2.138971000	2.992800000
H	2.700002000	-1.399919000	3.669008000
H	1.490022000	-1.579130000	2.410112000
C	2.261732000	-4.649684000	0.916151000
H	2.458184000	-5.213540000	1.829737000
C	3.203668000	2.572403000	2.345185000
H	4.003549000	2.166981000	1.710965000
C	1.395519000	-2.425070000	-2.686466000
H	1.410038000	-1.359009000	-2.394372000
C	0.579791000	5.023584000	1.019128000
H	0.022744000	5.895917000	1.369361000
C	-0.626009000	2.997595000	-2.581550000
H	-1.259098000	2.511720000	-1.819583000
H	-0.943759000	4.051218000	-2.594033000
C	1.294699000	-4.536621000	-1.282847000
H	0.725141000	-5.009776000	-2.084589000
C	1.591240000	-5.268785000	-0.136585000
H	1.271286000	-6.309574000	-0.049446000
C	1.556989000	-3.208315000	3.828511000
H	1.023355000	-3.943272000	3.208564000
H	0.821678000	-2.752443000	4.509639000
H	2.271335000	-3.762171000	4.457277000
C	-0.027922000	-2.698705000	-3.180709000
H	-0.153708000	-3.755795000	-3.466123000
H	-0.701599000	-2.541952000	-2.319563000
C	-0.904764000	2.387689000	-3.944568000
H	-0.591104000	1.332259000	-3.977673000
H	-0.377541000	2.914768000	-4.754096000
H	-1.979295000	2.426474000	-4.173558000
C	-0.466513000	-1.811541000	-4.333987000
H	-1.523117000	-1.982907000	-4.587852000
H	0.121925000	-1.995977000	-5.245155000
H	-0.355035000	-0.747371000	-4.073985000
C	2.539496000	1.339713000	2.982709000
H	2.078425000	0.745921000	2.174204000
H	3.325215000	0.693306000	3.405212000
C	1.484804000	1.635441000	4.034242000
H	0.987978000	0.709254000	4.360884000
H	0.710139000	2.306734000	3.636822000
H	1.915119000	2.111112000	4.929419000
H	3.542914232	3.261080087	3.104813767
H	4.005641384	-3.314998727	2.500998231
H	2.099656926	-2.693130384	-3.460244658
H	1.451571454	3.433937073	-2.816325320
H	5.761489478	2.677618682	-0.067962282
H	6.061109109	-1.855986729	-0.292864616
H	5.823707285	-1.728590458	-2.128137005
H	5.348286906	2.809934129	-1.863975093
H	5.188525104	-3.021581338	-1.251297154
H	4.610152122	3.810106944	-0.696810454

6. References

(1) B. Rösch, T. X. Gentner, J. Eyselein, J. Langer, H. Elsen, S. Harder, "Strongly reducing magnesium(0) complexes" *Nature,* 2021, ***592***, 717–721.

(2) C. Loh, S. Seupel, H. Görls, S. Krieck, M. Westerhausen, "*N*, *N'*-Bis(2,6-diisopropylphenyl) benzamidinates and -pivalamidinates of the s-Block Metals Lithium, Potassium, and Calcium" *Eur J Inorg Chem* 2014, **2014**, 1312–1321.

(3) R. E. H. Kuveke, L. Barwise, Y. Van Ingen, K. Vashisth, N. Roberts, S. S. Chitnis, J. L. Dutton, C. D. Martin and R. L. Melen, *ACS Cent. Sci.*, 2022, **8**, 855–863.

(4) A. Causero, G. Ballmann, J. Pahl, H. Zijlstra, C. Färber and S. Harder, *Organometallics*, 2016, **35**, 3350–3360.

(5) I. V. Basalov, D. M. Lyubov, G. K. Fukin, A. S. Shavyrin and A. A. Trifonov, *Angew. Chem. Int. Ed.*, 2012, **51**, 3444–3447.

(6) a) Rigaku Oxford Diffraction, **2024**, *CrysAlisPro Software system*, version 1.171.43.106a, Rigaku Corporation, Wroclaw, Poland (compounds **1-Yb**, **2-Yb** and **4-Yb**); b) Rigaku Oxford Diffraction, **2024**, CrysAlisPro Software system, version 1.171.44.85, Rigaku Corporation, Wroclaw, Poland (all other compounds).

(7) R. C. Clark, J. S. Reid, *Acta Crystallogr., Sect. A: Found. Crystallogr.* 1995, ***51***, 887-897.

(8) O. V. Dolomanov, L. J. Bourhis, R.J. Gildea, J. A. K. Howard, H. Puschmann, *J. Appl. Cryst.* 2009, **42**, 339-341.

(9) G. M. Sheldrick, *Acta Crystallogr., Sect. A: Found. Adv.* 2015, **71**, 3-8.

(10) G. M. Sheldrick, *Acta Crystallogr., Sect. C: Struct. Chem.* 2015, **71**, 3-8.

(11) A. Thorn, B. Dittrich and G. M. Sheldrick, *Acta Crystallogr., Sect. A: Found. Crystallogr.* 2012, **68**, 448–451.

(12) P. van der Sluis, A. L. Spek, *Acta Crystallogr., Sect. A: Found. Crystallogr.* 1990, **46**, 194–201.

(13) M. J. Frisch, G. W. Trucks, H. B. Schlegel, G. E. Scuseria, M. A. Robb, J. R. Cheeseman, G. Scalmani, V. Barone, G. A. Petersson, H. Nakatsuji, X. Li, M. Caricato, A. V. Marenich, J. Bloino, B. G. Janesko, R. Gomperts, B. Mennucci, H. P. Hratchian, J. V. Ortiz, A. F. Izmaylov, J. L. Sonnenberg, Williams, F. Ding, F. Lipparini, F. Egidi, J. Goings, B. Peng, A. Petrone, T. Henderson, D. Ranasinghe, V. G. Zakrzewski, J. Gao, N. Rega, G. Zheng, W. Liang, M. Hada, M. Ehara, K. Toyota, R. Fukuda, J. Hasegawa, M. Ishida, T. Nakajima, Y. Honda, O. Kitao, H. Nakai, T. Vreven, K. Throssell, J. A. Montgomery Jr., J. E. Peralta, F. Ogliaro, M. J. Bearpark, J. J. Heyd, E. N. Brothers, K. N. Kudin, V. N. Staroverov, T. A. Keith, R. Kobayashi, J. Normand, K. Raghavachari, A. P. Rendell, J. C. Burant, S. S. Iyengar, J. Tomasi, M. Cossi, J. M. Millam, M. Klene, C. Adamo, R. Cammi, J. W. Ochterski, R. L. Martin, K. Morokuma, O. Farkas, J. B. Foresman, D. J. Fox, *Gaussian 16*, Revision A.03; Gaussian, Inc.: Wallingford CT, 2016.

(14) (a) A. D. Becke, Density-Functional Exchange-Energy Approximation with Correct Asymptotic Behavior. *Phys. Rev. A* 1988, **38**, 3098-3100; (b) A. D. Becke, Density-functional thermochemistry. III. The role of exact exchange. *J. Chem. Phys.* 1993, **98**, 5648–5652.

(15) F. Weigend, R. Ahlrichs, Balanced Basis Sets of Split Valence, Triple Zeta Valence and Quadruple Zeta Valence Quality for H to Rn: Design and Assessment of Accuracy. *Phys. Chem. Chem. Phys.* 2005, **7**, 3297–3305.

(16)S. Grimme, J. Antony, S. Ehrlich, H. Krieg, A Consistend and Accurate Ab Initio Parametrization of Density Functional Dispersion Correction (DFT-D) for the 94 Elements H-Pu. *J. Chem. Phys.* 2010, **132**, 154104.
(17)S. Grimme, S. Ehrlich, L. Goerigk, Effect of the Damping Function in Dispersion Corrected Density Functional Theory. *J. Comput. Chem.* 2011, **32**, 1456-1465.
*(18)*Program Chemcraft - graphical software for visualization of quantum chemistry computations. Version 1.8, build 682. https://www.chemcraftprog.com.
(19)R. F. W. Bader, A Quantum Theory of Molecular Structure and its Applications. *Chem. Rev*. 1991, 893–928.
(20)T. A. Keith, Program AIMAll. Version 19.10.12, 2019, TK Gristmill Software, Overland Park KS, USA (aim.tkgristmill.com).
(21) V. Postils, C. Delgado-Alonso, J. M. Luis, P. Salvador, "An Objective Alternative to IUPAC's Approach To Assign Oxidation States" *Angew Chem Int Ed,* 2018, **57**, 10525-10529. (b) E. Ramos-Cordoba, V. Postils, P. Salvador, Oxidation States from Wave Function Analysis. *J. Chem. Theory Comput.* 2015, **11**, 1501–1508.
(22) P. Salvador, E. Ramos-Cordoba, M. Gimferrer, M. Montilla, *Program APOST-3D*, Version 4, Girona, **2020**.
(23) P. Salvador, E. Ramos-Cordoba, An Approximation to Bader's Topological Atom. *J. Chem. Phys.* 2013, **139**, 071103–071104.
(24) (a) K. Morokuma, Molecular Orbital Studies of Hydrogen Bonds. III. C = O···H–O Hydrogen Bond in $H_2CO\cdots H_2O$ and $H_2CO\cdots 2H_2O$. *J. Chem. Phys.* 1971, **55**, 1236–1244; (b) T. Ziegler, A. Rauk, On the Calculation of Bonding Energies by the Hartree Fock Slater Method. *Theor. Chim. Acta* 1977, **46**, 1–10, (c) F. M. Bickelhaupt, E. J. Baerends, Kohn-Sham Density Functional Theory: Predicting and Understanding Chemistry. *Rev. Comput. Chem.* 2000, **15**, 1–86, (d) L. Zhao, M. V. Hopffgarten, D. M. Andrada, G. Frenking, Energy Decomposition Analysis. *Wiley Interdiscip. Rev.: Comput. Mol. Sci.* 2018, **8**, e1345; (e) M. P. Mitoraj, A. Michalak, T. A. Ziegler, Combined Charge and Energy Decomposition Scheme for Bond Analysis. *J. Chem. Theory Comput.* 2009, **5**, 962– 975.
(25) AMS2024.102, SCM, *Theoretical Chemistry*; Vrije Universiteit, Amsterdam, The Netherlands, **2024** http://www.scm.com.
(26) E. Van Lenthe, E. J. Baerends, Optimized Slater-Type Basis Sets for the Elements 1-118. *J. Comput. Chem.* 2003, **24**, 1142–1156.
(27) (a) E. Van Lenthe, E. J. Baerends, J. G. Snijders, Relativistic Total Energy Using Regular Approximations. *J. Chem. Phys.* 1994, **101**, 9783–9792; (b) E. Van Lenthe, E. J. Baerends, J. G. Snijders, Relativistic Regular Two-Component Hamiltonians. *J. Chem. Phys.* 1993, **99**, 4597–4610.
